# Supplementary material for: Photocatalytic Z/E isomerization unlocking the stereodivergent construction of axially chiral alkene frameworks
Source: Nat Commun. 2024 Apr 16;15:3254. doi: 10.1038/s41467-024-47404-3 (PMC11021481; doi:10.1038/s41467-024-47404-3)
Supplement: Supplementary file 1 — Supplementary Information [file 41467_2024_47404_MOESM1_ESM.pdf]

Supplementary Information

Photocatalytic *Z*/*E* isomerization unlocking the stereodivergent construction of axially chiral alkene frameworks

Jie Wang,[+] Jun Gu,[+] Jia-Yu Zou, Meng-Jie Zhang, Rui Shen, Zhiwen Ye, Ping-Xun Xu, and Ying He*

School of Chemistry and Chemical Engineering, Nanjing University of Science and Technology, Nanjing 210094, China

[+] These authors contributed equally.

* E-mail: yhe@njust.edu.cn

Table of contents

General information...3

General procedure **A**: Synthesis of MBH carbonates 4

General procedure **B**: Synthesis of 2-quinolinone derivatives..5

General procedure **C**: Optimized conditions for accessing (*P*, *Z*)-**3** 7

General procedure **D**: One-pot synthesis of axially chiral products (*P*, *Z*)-**3**..8

General procedure **E**: Optimized conditions for accessing (*P*, *E*)-**3** 9

General procedure **F**: Synthesis of axially chiral products (*P*, *E*)-**3**..9

General procedure **G**: One-pot synthesis of axially chiral products (*P*, *E*)-**3** 9

Racemization experiments 40

Large-scale reactions for the synthesis of **3a**...42

Stereodivergent synthesis of axially chiral *N*-vinylquinolinones **3a**/**3s**...42

Stereodivergent transformations of axially chiral *N*-vinylquinolinones **3** 45

Photocatalyzed *Z*/*E* isomerization of non atropisomeric substrates..53

Density functional theory studies 55

X-ray crystal structures...59

NMR spectrum data...64

HPLC spectrum data...132

References..200

General information

Unless otherwise noted, all starting materials were purchased from commercial sources and used without any further purification. The analytical data for the known compounds were found to match with the literature data and stored at -20 ^{o}C under an inert atmosphere. Room temperature = 23-25 ^{o}C. Thin layer chromatograph plates were visualized under UV light (254 nm) or by staining with phosphomolybdic acid or $KMnO_4$ followed by heating. Abbreviations are reported as follows: DCM = dichloromethane, DCE = dichloroethane, THF = tetrahydrofuran, DMF = *N*,*N*-dimethylformamide, DME = 1,2-Dimethoxyethane, PE = petroleum ether, EA = ethyl acetate, TLC = thin layer chromatograph, dr = diastereomeric ratio. Nuclear magnetic resonance (NMR) spectra were recorded using an AVANCE 500 Bruker spectrometer and chemical shifts were reported in ppm. Multiplicities are reported using the following abbreviations: s = singlet, d = doublet, t = triplet, q = quartet, m = multiplet. High resolution mass spectral data were acquired on Agilent Technologies Accurate-Mass Q-TQF LC/MS 6520. Enantiomeric excesses (ee) were determined on a Thermo Ultimate 3000 Chiral HPLC by using AD, OD, IA, IC, and ID columns.

Photoreactions were performed in a foil-wrapped box, which is placed in lab at a constant 25 ^{o}C. The distance between the reaction vessels and the LED bulb (420 nm) was set at approximately 7-8 cm for all reactions which is shown in the picture.

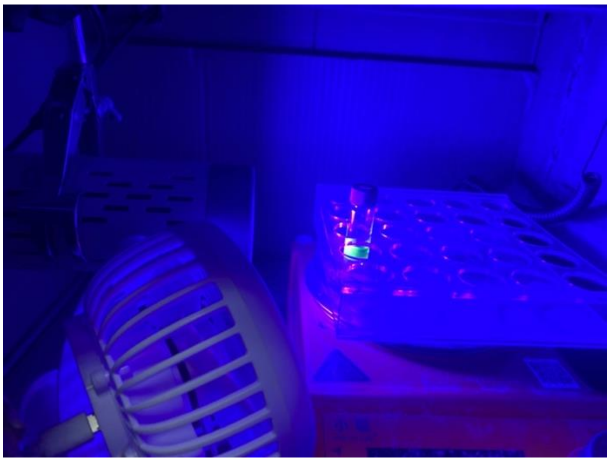

Supplementary Fig. 1. The picture of the photocatalytic reaction.

General procedure A: Synthesis of MBH carbonates

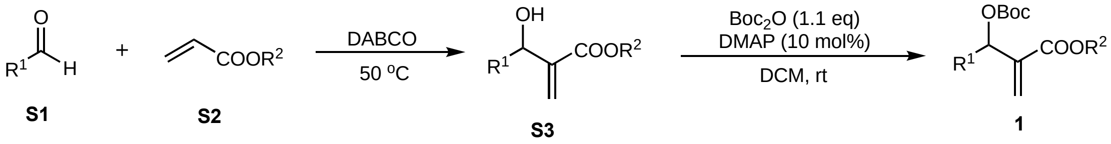

MBH alcohols **S3** were synthesized according to the following procedure. To a round flask equipped with a magnetic stirring bar was added aldehyde **S1** (10 mmol), acrylic ester **S2** (15 mmol) and DABCO (1,4-diazabicyclo[2.2.2]octane) (10 mmol). The reaction mixture was heated to 50 °C and stirred for 1-7 days. The reaction was monitored by TLC. When the reaction was completed, it was diluted with water and extracted with DCM (20 mL×3). The combined organic layers were washed with brine, dried over anhydrous Na_2SO_4, filtered and concentrated in vacuum. The crude product was purified by column chromatography on silica gel with PE/EA as eluent to give the desired alcohols **S3**.

To a round flask equipped with a magnetic stirring bar was added **S3** (10.0 mmol, 1.0 eq), DMAP (1.0 mmol, 0.1 eq) and DCM (50 mL). Boc_2O (11.0 mmol, 1.1 eq) was then added into the mixture at room temperature. The resulting mixture was stirred at room temperature for 0.5-2 hours, and then diluted with water and extracted with DCM (20 mL×3). The combined organic layers were washed with brine, dried over anhydrous Na_2SO_4, filtered and concentrated in vacuum. The crude product was purified by a silica gel flash chromatography (PE/EA) to give compound **1**.

***tert*-butyl 2-(benzo[*b*]thiophen-2-yl((*tert*-butoxycarbonyl)oxy)methyl)acrylate (1m)**

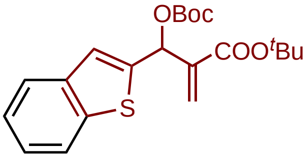

1m

Following the general procedure **A**, **1m** was obtained as white solid. **^{1}H NMR** (500 MHz, Chloroform-*d*) δ 7.84 (d, *J* = 7.4 Hz, 1H), 7.78 (d, *J* = 7.4 Hz, 1H), 7.40-7.35 (m, 3H), 6.80 (s, 1H), 6.46 (s, 1H), 6.05 (s, 1H), 1.54 (s, 9H), 1.47 (s, 9H). **^{13}C NMR** (126 MHz, Chloroform-*d*) δ 163.81, 152.30, 141.71, 140.29, 140.06, 139.24, 125.34, 124.64, 124.36, 123.94, 123.66, 122.41, 83.00, 81.82, 71.63, 27.99, 27.83. HRMS(ESI) m/z:

calculated for $[C_{21}H_{26}O_5S + H]^+$ 391.1574, found 391.1579.

General procedure B: Synthesis of 2-quinolinone derivatives

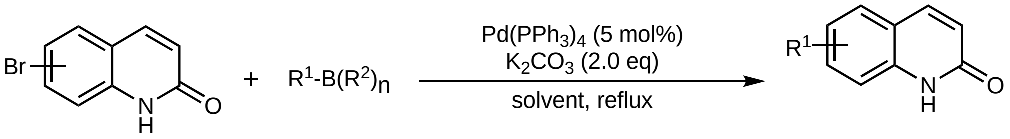

To a flame-dried round-bottom flask filled with argon were added 2-quinolinone (10 mmol, 1.0 eq), potassium methyltrifluoroborate or aryl boric acid ester (20 mmol, 2.0 eq), K_2CO_3 (20 mmol, 2.0 eq) and $Pd(PPh_3)_4$ (0.5 mmol, 0.05 eq). Toluene/MeOH/H_2O (10:3:2, 30 mL) were added before the mixture was heated to 110 °C, and then the mixture was stirred for 12 hours. After cooling down to room temperature, the mixture was filtered through celite and treated with water. Then the solution was separated and extracted with EA (20 mL x 3). The combined organic layers were washed with saturated NaCl aqueous (30 mL), dried over anhydrous Na_2SO_4, filtered and concentrated in vacuum. The crude product was purified by column chromatography over silica gel (PE: EA= 5:1 to 2:1) to afford the desired product.

7-(prop-1-en-2-yl)quinolin-2(1*H*)-one (2x)

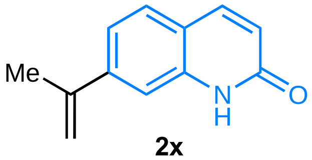

2x

Following the general procedure **B**, **2x** was obtained as white solid. **^{1}H NMR** (500 MHz, DMSO-*d*$_6$) δ 11.69 (s, 1H), 7.88 (d, J = 9.5 Hz, 1H), 7.62 (d, J = 8.2 Hz, 1H), 7.39 – 7.35 (m, 2H), 6.47 (d, J = 9.5 Hz, 1H), 5.52 (s, 1H), 5.23 (s, 1H), 2.13 (s, 3H). **^{13}C NMR** (126 MHz, DMSO-*d*$_6$) δ 161.42, 141.62, 141.33, 139.19, 138.44, 127.14, 121.11, 118.60, 117.89, 113.75, 111.11, 20.74. HRMS(ESI) m/z: calculated for $[C_{12}H_{11}NO + H]^+$ 186.0913, found 186.0919.

7-(4-((((1*S*,2*R*,5*S*)-2-isopropyl-5-methylcyclohexyl)oxy)methyl)phenyl)quinolin-2(1*H*)-one (2y)

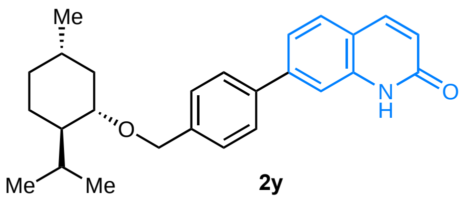

2y

Following the general procedure **B**, **2y** was obtained as white solid. **^{1}H NMR** (500 MHz, Chloroform-*d*) δ 12.36 (s, 1H), 7.88 (d, *J* = 9.4 Hz, 1H), 7.70 – 7.65 (m, 4H), 7.51 (d, *J* = 7.7 Hz, 3H), 6.78 (d, *J* = 9.4 Hz, 1H), 4.78 (d, *J* = 11.5 Hz, 1H), 4.52 (d, *J* = 11.6 Hz, 1H), 3.27 (td, *J* = 10.5, 4.0 Hz, 1H), 2.42 – 2.36 (m, 1H), 2.28 (d, *J* = 12.3 Hz, 1H), 1.82 (s, 1H), 1.74 – 1.68 (m, 2H), 1.38 (t, *J* = 11.3 Hz, 1H), 1.09 – 0.92 (m, 9H), 0.81 (d, *J* = 6.9 Hz, 3H). **^{13}C NMR** (126 MHz, Chloroform-*d*) δ 154.94, 142.61, 139.66, 138.27, 137.92, 127.30, 127.12, 126.37, 121.00, 120.17, 118.03, 113.05, 77.97, 69.02, 47.37, 39.35, 33.58, 30.59, 24.59, 22.30, 21.39, 20.04, 15.15. HRMS(ESI) m/z: calculated for $[C_{26}H_{31}NO_2 + H]^+$ 390.2428, found 390.2433.

6-(4-((((*R*)-2,5,7,8-tetramethyl-2-((4*R*,8*R*)-4,8,12-trimethyltridecyl)chroman-6-yl)oxy)methyl)phenyl)quinolin-2(1*H*)-one (2z)

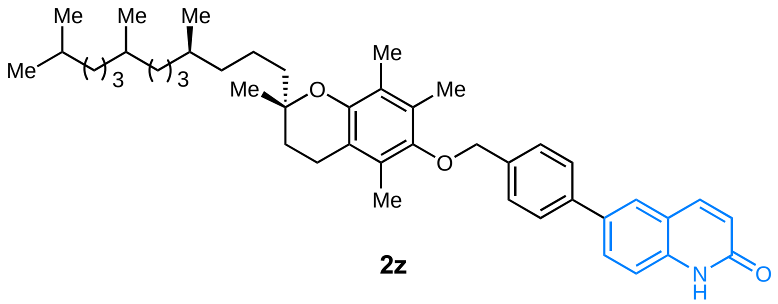

2z

Following the general procedure **B**, **2z** was obtained as white solid. **^{1}H NMR** (500 MHz, Chloroform-*d*) δ 11.68 (s, 1H), 7.95 (d, *J* = 9.5 Hz, 1H), 7.84 (d, *J* = 8.3 Hz, 2H), 7.71 (d, *J* = 7.9 Hz, 2H), 7.66 (d, *J* = 8.0 Hz, 2H), 7.52 (d, *J* = 8.3 Hz, 1H), 6.82 (d, *J* = 9.5 Hz, 1H), 4.81 (s, 2H), 2.66 (t, *J* = 6.8 Hz, 2H), 2.30 (s, 3H), 2.25 (s, 3H), 2.17 (s, 3H), 1.86 (dq, *J* = 20.1, 6.8 Hz, 2H), 1.63 – 1.56 (m, 3H), 1.50 – 1.43 (m, 4H), 1.31 (s, 10H), 1.23 – 1.09 (m, 8H), 0.92 (d, *J* = 6.4 Hz,11H). **^{13}C NMR** (126 MHz,Chloroform-*d*) δ 147.11, 146.97, 140.15, 138.50, 136.75, 136.35, 134.71, 131.13, 131.05, 130.93, 128.86, 127.54, 127.26, 126.89, 126.06, 124.87, 121.98, 120.81, 119.23, 116.64, 115.60, 73.85, 73.32, 39.05, 38.37, 36.49, 36.46, 36.42, 36.29, 31.80, 31.71, 30.33, 26.98, 23.80, 23.45, 22.89, 21.71, 21.62, 20.04, 19.70, 18.75, 18.67, 11.91, 11.04, 10.83. HRMS(ESI) m/z:

calculated for $[C_{45}H_{61}NO_3 + H]^+$ 664.4724, found 664.4730.

General procedure C: Optimized conditions for accessing (*P*, *Z*)-3

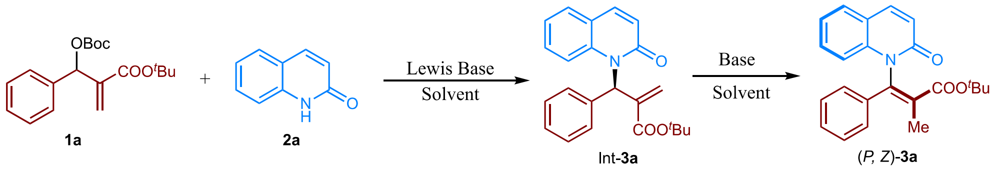

Entry	Lewis base	solvent	yield (%)	ee (%)
1	Quinine	Toluene	69	8
2	Hydroquinine	Toluene	87	13
3	(DHQ)$_2$AQN	Toluene	89	25
4	(DHQ)$_2$PHAL	Toluene	23	8
5	(DHQD)$_2$PYR	Toluene	55	85
6	(DHQD)$_2$PYR	DCE	60	89
7	(DHQD)$_2$PYR	MeCN	75	90
8	(DHQD)$_2$PYR	MTBE	61	92
9	(DHQD)$_2$PYR	DME	83	94

Optimization of the first step conditions: **1a** (0.3 mmol), **2a** (0.1 mmol), Lewis Base (20 mmol%), solvent (1.0 mL), rt, isolated yield, the ee values were calculated by chiral HPLC traces.

Entry	Base	solvent	yield (%)	ee (%)	*Z/E*
1	NaO^{t}Bu	Toluene	51	85	2/1
2	DBU	Toluene	57	73	3/1
3	PhONa	Toluene	94	86	>20/1
4	MeONa	Toluene	91	94	>20/1
5	MeONa	DCE	84	87	12/1
6	MeONa	THF	84	94	13/1
7	MeONa	Dioxane	82	79	6/1

Optimization of the second step conditions: Int-**3a** (0.1 mmol), Base (0.15 mmol), solvent (1.0 mL), rt, isolated yield, the ee values were calculated by chiral HPLC traces. The *Z*/*E* ratio of **3a** was calculated by ^{1}H-NMR.

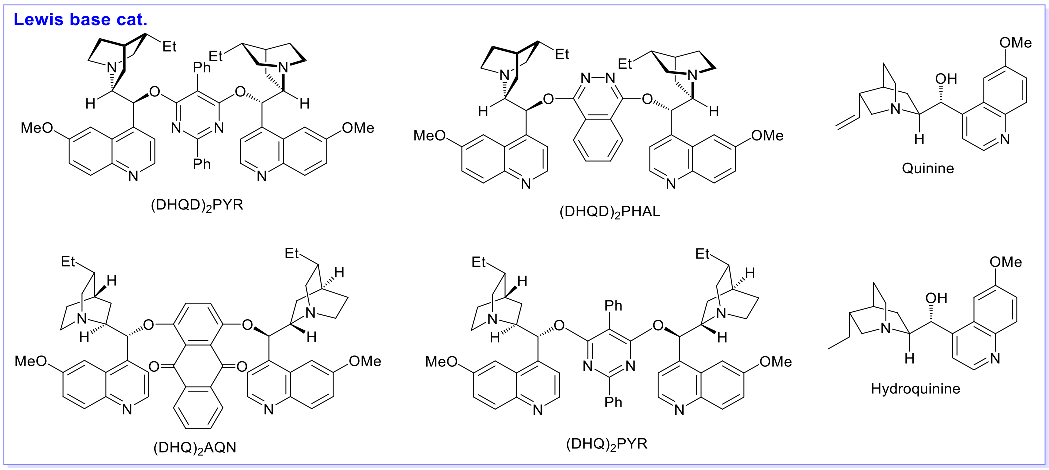

Supplementary Fig. 2. Optimization of the reaction

Compound **1a** (0.3 mmol), **2a** (0.1 mmol) and the Lewis base catalyst (0.02 mmol) were added to a 2 dram scintillation vial equipped with a magnetic stirring bar. The vial was then charged with solvent (1.0 mL) and stirred at room temperature for several days. The reaction was monitored by TLC analyses. After the reaction completed, the residue was purified directly by column chromatography over silica gel (PE: EA = 10:1 to 6:1) to afford the desired centrally chiral product **Int-3a**. The absolute configuration

of Int-**3a** were confirmed by single-crystal X-ray analysis as shown in Supplementary Table 7 (CCDC 2089101).

Compound **Int-3a** (0.1 mmol) and base (0.15 mmol) were added to a 2 dram scintillation vial equipped with a magnetic stirring bar. The vial was then charged with toluene (1.0 mL) and stirred at room temperature for indicated time. The reaction was monitored by TLC analyses. After the reaction completed, the residue was purified directly by column chromatography over silica gel (PE: EA = 10:1 to 5:1) to afford the desired product (*P*, *Z*)-**3a**. The *Z*/*E* ratio was determined by crude ^{1}H-NMR of the mixture.

General procedure D: One-pot synthesis of axially chiral products (*P*, *Z*)-3

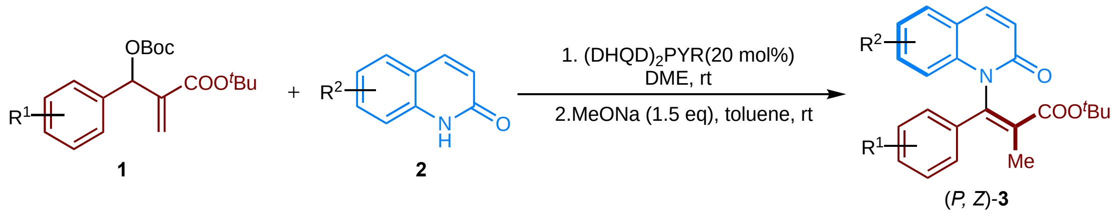

The catalyst (DHQD)$_2$PYR (0.06 mmol, 52.8 mg), **1** (0.9 mmol), **2** (0.3 mmol,) were added to a 2 dram scintillation vial equipped with a magnetic stirring bar. The vial was then charged with DME (1.5 mL) and stirred at room temperature for several days. The reaction was monitored by TLC analyses. When the reaction completed, the solvent in the vial was removed by distillation under reduced pressure. Then MeONa (0.45 mmol, 1.5 eq) was added into the vial. The vial was then charged with toluene (1.0 mL) and stirred at room temperature for about 1 h until the full consumption of the intermediate by TLC monitoring. Then the residue was purified directly by column chromatography over silica gel (PE: EA = 10:1 to 5:1) to afford the desired product (*P*, *Z*)-**3**.

It should be noted that we also tried the reaction of **1a** with 8-bromoquinolin-2(1*H*)-one. However, no desired product **3** was obtained.

General procedure E: Optimized conditions for accessing (*P*, *E*)-3

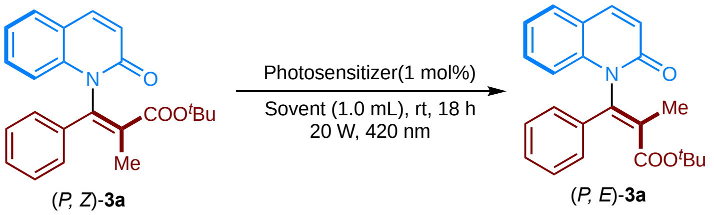

The compound (*P*, *Z*)-**3a** (0.1 mmol, 1.0 eq) and photosensitizer (1 mol%) were weighed out into a 2 dram scintillation vial. The vial was charged with solvent (1.0 mL) and the reaction was stirred at room temperature under visible light irradiation (420 nm) for 18 h. Then the mixture was concentrated under reduced pressure directly. The residue was dissolved in 0.5 mL $CDCl_3$ and then detected by ^{1}H-NMR to afford the *E*/*Z* ratio of **3a**.

General procedure F: Synthesis of axially chiral products (*P*, *E*)-3

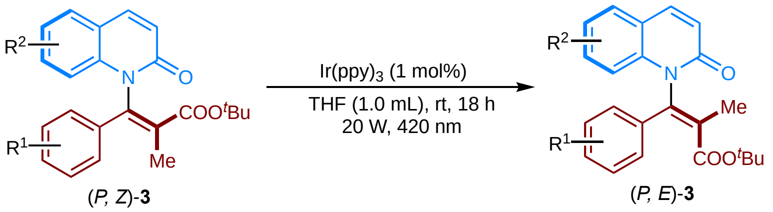

Compound (*P*, *Z*)-**3** (0.1 mmol, 1.0 eq) and Ir(ppy)$_3$ (1 mol%) were weighed out into a 2 dram scintillation vial. The vial was charged with THF (1.0 mL) and the reaction was stirred at room temperature under visible light irradiation (420 nm) for 18 h. Then the mixture was concentrated under reduced pressure and purified directly by column chromatography over silica gel (PE: EA = 50:1 to 10:1) to afford the desired product (*P*, *E*)-**3**. The *Z*/*E* ratio was determined by crude ^{1}H-NMR of the mixture.

General procedure G: One-pot synthesis of axially chiral products (*P*, *E*)-3

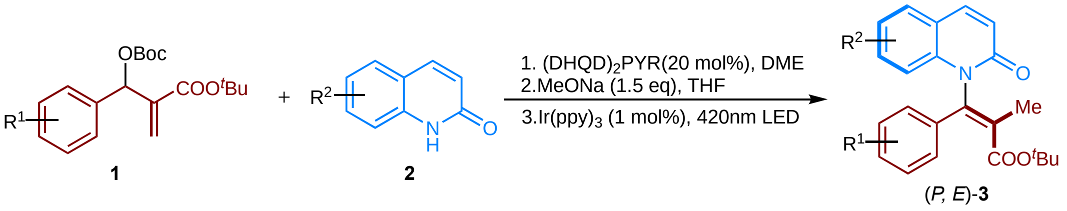

The catalyst $(DHQD)_2PYR$ (0.04 mmol, 35.2 mg), **1a** (0.6 mmol), **2a/2s** (0.2 mmol) were added to a 2 dram scintillation vial equipped with a magnetic stirring bar. The vial was then charged with DME (1.0 mL) and stirred at room temperature for several days. After completion of the reaction detected by TLC, the solvent in the vial was removed by distillation under reduced pressure. Then MeONa (0.3 mmol, 1.5 eq) was added into the vial. The vial was then charged with THF (1.0 mL) and stirred at room temperature for about 1 h until the full consumption of the intermediate by TLC monitoring. Then $Ir(ppy)_3$ (1 mol%) was added into the vial and the reaction was stirred at room temperature under visible light irradiation (420 nm) for 18 h. After that, the mixture was concentrated under reduced pressure and purified directly by column chromatography over silica gel (PE: EA = 50:1 to 10:1) to afford the desired product (*P*, *E*)-**3a**/(*P*, *E*)-**3s**.

***tert*-butyl (*P*, *Z*)-2-methyl-3-(2-oxoquinolin-1(2*H*)-yl)-3-phenylacrylate [(*P*, *Z*)-3a]**

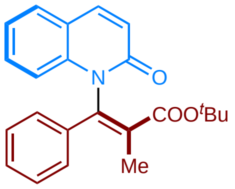

(*P*, *Z*)-**3a**

Following the general procedure of **D**, (*P*, *Z*)-**3a** was obtained as white solid (87% yield). **^{1}H NMR** (500 MHz, Chloroform-*d*) δ 7.71 (d, *J* = 9.5 Hz, 1H), 7.54 (dd, *J* = 7.8, 1.5 Hz, 1H), 7.48 – 7.44 (m, 3H), 7.37 (d, *J* = 8.5 Hz, 1H), 7.34 – 7.29 (m, 3H), 7.18 (td, *J* = 7.5, 1.1 Hz, 1H), 6.69 (d, *J* = 9.5 Hz, 1H), 2.27 (s, 3H), 1.03 (s, 9H). **^{13}C NMR** (126 MHz, Chloroform-*d*) δ 165.76, 160.42, 139.21, 138.77, 136.11, 134.93, 131.59, 129.62, 128.25, 127.84, 127.32, 127.23, 121.42, 121.33, 119.30, 114.78, 80.47, 26.35, 16.51. HRMS(ESI) m/z: calculated for $[C_{23}H_{23}NO_3+H]^+$ 362.1751, found 362.1750. HPLC data (Chiralpak OD column, hexane: isopropanol = 95:5, 1.0 mL/min), tr = 12.4 min (minor), tr = 14.1 min (major), ee = 94%.

***tert*-butyl (*P*, *E*)-2-methyl-3-(2-oxoquinolin-1(2*H*)-yl)-3-phenylacrylate [(*P*, *E*)-3a]**

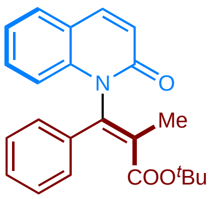

(P, E)-**3a**

Following the general procedure of **F**, (P, E)-**3a** was obtained as white solid (79% yield). E/Z= 8/1. ^{1}**H NMR** (500 MHz, Chloroform-d) δ 7.73 (d, J = 9.5 Hz, 1H), 7.58 (dd, J = 7.8, 1.5 Hz, 1H), 7.51 (ddd, J = 8.6, 7.1, 1.5 Hz, 1H), 7.45 (d, J = 8.4 Hz, 1H), 7.42 – 7.40 (m, 2H), 7.27 – 7.22 (m, 4H), 6.69 (d, J = 9.5 Hz, 1H), 1.77 (s, 3H), 1.32 (s, 9H). ^{13}**C NMR** (126 MHz, Chloroform-d) δ 168.20, 160.80, 140.01, 139.11, 137.73, 136.78, 133.71, 131.03, 128.73, 128.68, 128.59, 128.07, 122.71, 122.46, 120.49, 115.23, 82.00, 27.67, 16.48. HRMS(ESI) m/z: calculated for $[C_{23}H_{23}NO_3+H]^+$ 362.1751, found 362.1749. HPLC data (Chiralpak OD column, hexane: isopropanol = 95:5, 1.0 mL/min), tr = 15.5 min (minor), tr = 23.3 min (minor), ee = 94%.

***tert*-butyl (*P*, *Z*)-3-(4-bromophenyl)-2-methyl-3-(2-oxoquinolin-1(2*H*)-yl)acrylate [(*P*, *Z*)-3b]**

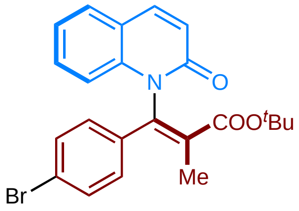

(P, Z)-**3b**

Following the general procedure of **D**, (P, Z)-**3b** was obtained as white solid (84% yield). ^{1}**H NMR** (500 MHz, Chloroform-d) δ 7.72 (d, J = 9.5 Hz, 1H), 7.56 (dd, J = 7.8, 1.5 Hz, 1H), 7.49 – 7.43 (m, 3H), 7.33 (d, J = 8.6 Hz, 3H), 7.22 – 7.18 (m, 1H), 6.68 (d, J = 9.5 Hz, 1H), 2.25 (s, 3H), 1.02 (s, 9H). ^{13}**C NMR** (126 MHz, Chloroform-d) δ 165.43, 160.35, 139.04, 138.91, 135.07, 133.94, 132.19, 130.51, 129.86, 129.73, 127.47, 122.09, 121.51, 121.34, 119.32, 114.51, 80.68, 26.33, 16.47. HRMS(ESI) m/z: calculated for $[C_{23}H_{22}BrNO_3+H]^+$ 440.0856, found 440.0849. HPLC data (Chiralpak AD column, hexane: isopropanol = 88:12, 1.0 mL/min), tr = 10.4 min (minor), tr = 42.8 min (major), ee = 91%.

***tert*-butyl (*P*, *E*)-3-(4-bromophenyl)-2-methyl-3-(2-oxoquinolin-1(2*H*)-yl)acrylate**

[(***P***, ***E***)-3b]

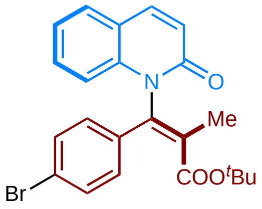

(P, E)-**3b**

Following the general procedure of **F**, (P, E)-**3b** was obtained as white solid (86% yield). E/Z= 12/1. ^{1}**H NMR** (500 MHz, Chloroform-d) δ 7.74 (d, J = 9.5 Hz, 1H), 7.59 (dd, J = 7.8, 1.5 Hz, 1H), 7.53 (ddd, J = 8.7, 7.3, 1.5 Hz, 1H), 7.40 – 7.38 (m, 3H), 7.29 – 7.23 (m, 3H), 6.68 (d, J = 9.5 Hz, 1H), 1.76 (s, 3H), 1.37 (s, 9H). ^{13}**C NMR** (126 MHz, Chloroform-d) δ 167.82, 160.78, 140.22, 138.92, 136.69, 135.78, 134.40, 131.28, 131.20, 130.20, 128.92, 122.93, 122.90, 122.34, 120.51, 114.93, 82.36, 27.74, 16.53. HRMS(ESI) m/z: calculated for $[C_{23}H_{22}BrNO_3+H]^+$ 440.0856, found 440.0847. HPLC data (Chiralpak AD column, hexane: isopropanol = 95:5, 1.0 mL/min), tr = 20.0 min (major), tr = 33.0 min (minor), ee = 92%.

***tert*-butyl (*P*, *Z*)-3-(4-chlorophenyl)-2-methyl-3-(2-oxoquinolin-1(2*H*)-yl)acrylate [(*P*, *Z*)-3c]**

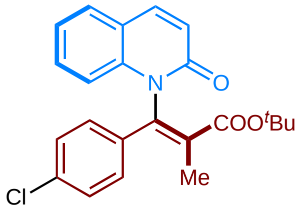

(P, Z)-**3c**

Following the general procedure of **D**, (P, Z)-**3c** was obtained as white solid (92% yield). ^{1}**H NMR** (500 MHz, Chloroform-d) δ 7.77 (d, J = 9.6 Hz, 1H), 7.61 (d, J = 7.7 Hz, 1H), 7.53 (t, J = 7.9 Hz, 1H), 7.44 – 7.42 (m, 2H), 7.38 (d, J = 8.5 Hz, 1H), 7.34 (d, J = 8.6 Hz, 2H), 7.26 (t, J = 7.5 Hz, 1H), 6.73 (d, J = 9.5 Hz, 1H), 2.30 (s, 3H), 1.07 (s, 9H). ^{13}**C NMR** (126 MHz, Chloroform-d) δ 164.22, 161.59, 139.62, 138.56, 138.50, 136.03, 133.39, 128.61, 128.12, 127.71, 127.21, 125.46, 125.28, 121.01, 120.34, 116.11, 80.43, 26.71, 20.05. HRMS(ESI) m/z: calculated for $[C_{23}H_{22}ClNO_3+H]^+$ 396.1361, found 396.1361. HPLC data (Chiralpak AD column, hexane: isopropanol = 88:12, 1.0 mL/min), tr = 9.5 min (minor), tr = 37.8 min (major), ee =91%.

***tert*-butyl (*P*, *E*)-3-(4-chlorophenyl)-2-methyl-3-(2-oxoquinolin-1(2*H*)-yl)acrylate [(*P*, *E*)-3c]**

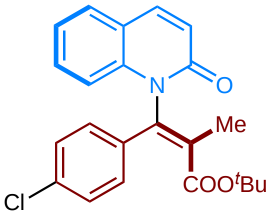

(*P*, *E*)-**3c**

Following the general procedure of **F**, (*P*, *E*)-**3c** was obtained as white solid (83% yield). *E*/*Z*= 11/1. ^{1}**H NMR** (500 MHz, Chloroform-*d*) δ 7.74 (d, *J* = 9.4 Hz, 1H), 7.60 (dd, *J* = 7.8, 1.5 Hz, 1H), 7.53 (ddd, *J* = 8.6, 7.2, 1.5 Hz, 1H), 7.40 (d, *J* = 8.4 Hz, 1H), 7.35 – 7.33 (m, 2H), 7.27 – 7.22 (m, 3H), 6.68 (d, *J* = 9.5 Hz, 1H), 1.77 (s, 3H), 1.36 (s, 9H). ^{13}**C NMR** (126 MHz, Chloroform-*d*) δ 167.84, 160.77, 140.19, 138.94, 136.63, 135.31, 134.63, 134.36, 131.18, 129.94, 128.90, 128.32, 122.90, 122.35, 120.51, 114.94, 82.32, 27.74, 16.50. HRMS(ESI) m/z: calculated for $[C_{23}H_{22}ClNO_3+H]^+$ 396.1361, found 396.1361. HPLC data (Chiralpak AD column, hexane: isopropanol = 90:10, 1.0 mL/min), tr = 10.2 min (major), tr = 14.7 min (minor), ee =91%.

***tert*-butyl (*P*, *Z*)-2-methyl-3-(2-oxoquinolin-1(2*H*)-yl)-3-(*p*-tolyl)acrylate [(*P*, *Z*)-3d]**

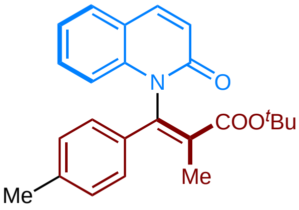

(*P*, *Z*)-**3d**

Following the general procedure of **D**, (*P*, *Z*)-**3d** was obtained as white solid (85% yield). ^{1}**H NMR** (500 MHz, Chloroform-*d*) δ 7.70 (d, *J* = 9.5 Hz, 1H), 7.54 (dd, *J* = 7.8, 1.5 Hz, 1H), 7.45 (ddd, *J* = 8.6, 7.2, 1.5 Hz, 1H), 7.34 (dd, *J* = 11.9, 8.3 Hz, 3H), 7.18 (td, *J* = 7.5, 1.1 Hz, 1H), 7.12 (d, *J* = 8.0 Hz, 2H), 6.69 (d, *J* = 9.5 Hz, 1H), 2.31 (s, 3H), 2.28 (s, 3H), 1.02 (s, 9H). ^{13}**C NMR** (126 MHz, Chloroform-*d*) δ 165.85, 160.39, 139.26, 138.67, 137.88, 136.28, 132.06, 130.95, 129.57, 128.16, 127.94, 127.26, 121.47, 121.25, 119.28, 114.82, 80.34, 26.36, 20.30, 16.54. HRMS(ESI) m/z: calculated for $[C_{24}H_{25}NO_3+H]^+$ 376.1907, found 376.1901. HPLC data (Chiralpak AD column, hexane: isopropanol = 80:20, 1.0 mL/min), tr = 6.0 min (minor), tr = 14.5 min

(major), ee = 95%.

***tert*-butyl (*P*, *E*)-2-methyl-3-(2-oxoquinolin-1(2*H*)-yl)-3-(*p*-tolyl)acrylate [(*P*, *E*)-3d]**

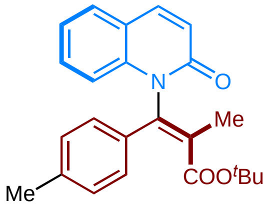

(*P*, *E*)-**3d**

Following the general procedure of **F**, (*P*, *E*)-**3d** was obtained as white solid (72% yield). *E*/*Z*= 5/1. ^{1}**H NMR** (500 MHz, Chloroform-*d*) δ 7.72 (d, *J* = 9.6 Hz, 1H), 7.57 (dd, *J* = 7.7, 1.5 Hz, 1H), 7.50 (ddd, *J* = 8.7, 7.1, 1.5 Hz, 1H), 7.43 (d, *J* = 8.4 Hz, 1H), 7.29 – 7.26 (m, 2H), 7.24 – 7.21 (m, 1H), 7.06 (d, *J* = 8.0 Hz, 2H), 6.69 (d, *J* = 9.5 Hz, 1H), 2.29 (s, 3H), 1.75 (s, 3H), 1.36 (s, 9H). ^{13}**C NMR** (126 MHz, Chloroform-*d*) δ 168.36, 160.79, 139.92, 139.17, 138.63, 137.75, 133.80, 132.97, 130.99, 128.77, 128.65, 128.41, 122.64, 122.49, 120.47, 115.29, 81.92, 27.73, 21.31, 16.47. HRMS(ESI) m/z: calculated for $[C_{24}H_{25}NO_3+H]^+$ 376.1907, found 376.1903. HPLC data (Chiralpak AD column, hexane: isopropanol = 90:10, 1.0 mL/min), tr = 10.8 min (major), tr = 12.2 min (minor), ee = 94%.

***tert*-butyl (*P*, *Z*)-3-(4-(*tert*-butyl)phenyl)-2-methyl-3-(2-oxoquinolin-1(2*H*)-yl)acrylate [(*P*, *Z*)-3e]**

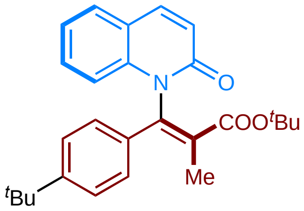

(*P*, *Z*)-**3e**

Following the general procedure of **D**, (*P*, *Z*)-**3e** was obtained as white solid (88% yield). ^{1}**H NMR** (500 MHz, Chloroform-*d*) δ 7.76 (d, *J* = 9.5 Hz, 1H), 7.59 (dd, *J* = 7.7, 1.5 Hz, 1H), 7.51 (ddd, *J* = 8.6, 7.1, 1.6 Hz, 1H), 7.43 – 7.40 (m, 3H), 7.37 (d, *J* = 8.6 Hz, 2H), 7.23 (td, *J* = 7.4, 1.1 Hz, 1H), 6.75 (d, *J* = 9.5 Hz, 1H), 2.34 (s, 3H), 1.32 (s, 9H), 1.07 (s, 9H). ^{13}**C NMR** (126 MHz, Chloroform-*d*) δ 166.92, 161.44, 151.89, 140.38, 139.71, 137.34, 132.98, 132.00, 130.61, 128.96, 128.29, 125.19, 122.51, 122.28, 120.32, 115.91, 81.38, 34.70, 31.21, 27.41, 17.60. HRMS(ESI) m/z: calculated for

$[C_{27}H_{31}NO_3+H]^+$ 418.2377, found 418.2376. HPLC data (Chiralpak AD column, hexane: isopropanol = 85:15, 1.0 mL/min), tr = 6.6 min (minor), tr = 13.1 min (major), ee = 88%.

***tert*-butyl (*P, E*)-3-(4-(*tert*-butyl)phenyl)-2-methyl-3-(2-oxoquinolin-1(2*H*)-yl)acrylate [(*P, E*)-3e]**

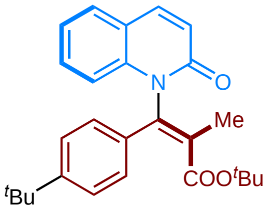

(*P, E*)-**3e**

Following the general procedure of **F**, (*P*, *E*)-**3e** was obtained as white solid (85% yield). *E*/*Z*= 15/1. **^{1}H NMR** (500 MHz, Chloroform-*d*) δ 7.72 (d, *J* = 9.6 Hz, 1H), 7.57 (dd, *J* = 7.9, 1.5 Hz, 1H), 7.51 (ddd, *J* = 8.6, 7.0, 1.5 Hz, 1H), 7.45 (d, *J* = 7.9 Hz, 1H), 7.34 – 7.32 (m, 2H), 7.28 – 7.25 (m, 2H), 7.24 – 7.21 (m, 1H), 6.69 (d, *J* = 9.5 Hz, 1H), 1.76 (s, 3H), 1.32 (s, 9H), 1.25 (s, 9H). **^{13}C NMR** (126 MHz, Chloroform-d) δ 168.35, 160.80, 151.70, 139.95, 139.14, 137.86, 133.80, 133.08, 131.00, 128.68, 128.25, 124.96, 122.66, 122.46, 120.48, 115.30, 81.86, 34.62, 31.25, 27.64, 16.40. HRMS(ESI) m/z: calculated for $[C_{27}H_{31}NO_3+H]^+$ 418.2377, found 418.2370. HPLC data (Chiralpak OD column, hexane: isopropanol = 95:5, 1.0 mL/min), tr = 15.6 min (minor), tr = 18.9 min (major), ee = 89%.

***tert*-butyl (*P, Z*)-3-([1,1'-biphenyl]-4-yl)-2-methyl-3-(2-oxoquinolin-1(2*H*)-yl)acrylate [(*P, Z*)-3f]**

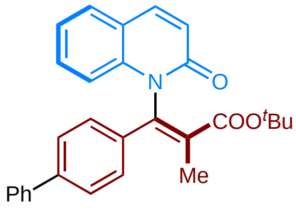

(*P, Z*)-**3f**

Following the general procedure of **D**, (*P*, *Z*)-**3f** was obtained as white solid (81% yield). **^{1}H NMR** (500 MHz, Chloroform-*d*) δ 7.79 (d, *J* = 9.5 Hz, 1H), 7.61 (d, *J* = 8.2 Hz, 2H), 7.59 – 7.56 (m, 5H), 7.53 (d, *J* = 7.3 Hz, 1H), 7.46 (t, *J* = 7.2 Hz, 3H), 7.38 (t, *J* = 7.3 Hz, 1H), 7.26 (t, *J* = 7.4 Hz, 1H), 6.77 (d, *J* = 9.6 Hz, 1H), 2.39 (s, 3H), 1.09 (s, 9H).

^{13}**C NMR** (126 MHz, Chloroform-*d*) δ 166.78, 161.48, 141.66, 140.38, 140.35, 139.84, 136.99, 134.93, 132.62, 130.70, 129.73, 128.83, 128.40, 127.63, 127.11, 126.98, 122.51, 122.41, 120.38, 115.83, 81.52, 27.43, 17.65. HRMS(ESI) m/z: calculated for $[C_{29}H_{27}NO_3+H]^+$ 438.2064, found 438.2063. HPLC data (Chiralpak AD column, hexane: isopropanol = 80:20, 1.0 mL/min), tr = 7.5min (minor), tr = 25.4 min (major), ee = 90%.

***tert*-butyl (*P*, *E*)-3-([1,1'-biphenyl]-4-yl)-2-methyl-3-(2-oxoquinolin-1(2*H*)-yl)acrylate [(*P*, *E*)-3f]**

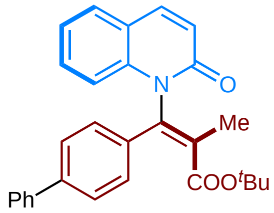

(*P*, *E*)-**3f**

Following the general procedure of **F**, (*P*, *E*)-**3f** was obtained as white solid (79% yield). *E*/*Z*= 10/1. ^{1}**H NMR** (500 MHz, Chloroform-*d*) δ 7.75 (d, *J* = 9.6 Hz, 1H), 7.59 (dd, *J* = 7.8, 1.5 Hz, 1H), 7.53 (dt, *J* = 7.7, 1.8 Hz, 3H), 7.48 (q, *J* = 2.8 Hz, 5H), 7.40 (dd, *J* = 8.4, 6.9 Hz, 2H), 7.34 – 7.30 (m, 1H), 7.26 – 7.23 (m, 1H), 6.71 (d, *J* = 9.5 Hz, 1H), 1.79 (s, 3H), 1.37 (s, 9H). ^{13}**C NMR** (126 MHz, Chloroform-*d*) δ 168.24, 160.87, 141.53, 140.62, 140.10, 139.17, 137.50, 135.71, 133.71, 131.13, 128.98, 128.82, 128.80, 127.53, 127.11, 126.83, 122.80, 122.47, 120.53, 115.22, 82.15, 27.75, 16.57. HRMS(ESI) m/z: calculated for $[C_{29}H_{27}NO_3+H]^+$ 438.2064, found 438.2063. HPLC data (Chiralpak AD column, hexane: isopropanol = 85:15, 1.0 mL/min), tr = 10.2min (major), tr = 13.0 min (minor), ee = 89%.

***tert*-butyl (*P*, *Z*)-2-methyl-3-(2-oxoquinolin-1(2*H*)-yl)-3-(4-(prop-1-en-2-yl)phenyl)acrylate [(*P*, *Z*)-3g]**

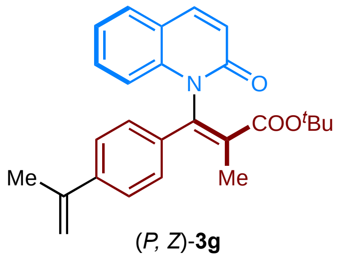

(*P*, *Z*)-**3g**

Following the general procedure of **D**, (*P*, *Z*)-**3g** was obtained as white solid (74%

yield). **^{1}H NMR** (500 MHz, Chloroform-*d*) δ 7.64 (d, *J* = 9.4 Hz, 1H), 7.47 (d, *J* = 7.6 Hz, 1H), 7.39 (t, *J* = 7.8 Hz, 1H), 7.33 (s, 4H), 7.28 (d, *J* = 8.9 Hz, 1H), 7.11 (t, *J* = 7.4 Hz, 1H), 6.62 (d, *J* = 9.4 Hz, 1H), 5.28 (s, 1H), 5.00 (s, 1H), 2.22 (s, 3H), 2.02 (s, 3H), 0.95 (s, 9H).**^{13}C NMR** (126 MHz, Chloroform-*d*) δ 166.79, 161.45, 142.54, 141.63, 140.29, 139.80, 137.00, 134.97, 132.46, 130.65, 129.19, 128.35, 125.36, 122.46, 122.37, 120.35, 115.84, 113.29, 81.48, 27.41, 21.62, 17.58. HRMS(ESI) m/z: calculated for $[C_{26}H_{27}NO_3+H]^+$ 402.2064, found 402.2068. HPLC data (Chiralpak IC column, hexane: isopropanol = 80:20, 1.0 mL/min), tr =23.7 min (minor), tr = 43.4 min (major), ee = 90%.

***tert*-butyl (*P*, *E*)-2-methyl-3-(2-oxoquinolin-1(2*H*)-yl)-3-(4-(prop-1-en-2-yl)phenyl)acrylate [(*P*, *E*)-3g]**

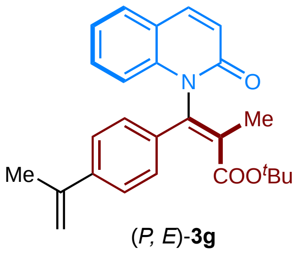

(*P*, *E*)-**3g**

Following the general procedure of **F**, (*P*, *E*)-**3g** was obtained as white solid (64% yield). *E*/*Z*= 5/1. **^{1}H NMR** (500 MHz, Chloroform-*d*) δ 7.74 (d, *J* = 9.5 Hz, 1H), 7.58 (dd, *J* = 7.8, 1.5 Hz, 1H), 7.51 (ddd, *J* = 8.7, 7.2, 1.5 Hz, 1H), 7.44 – 7.43 (m, 1H), 7.35 (s, 4H), 7.25 – 7.22 (m, 1H), 6.70 (d, *J* = 9.5 Hz, 1H), 5.33 – 5.32 (m, 1H), 5.05 (t, *J* = 1.5 Hz, 1H), 2.09 (d, *J* = 0.8 Hz, 3H), 1.77 (s, 3H), 1.36 (s, 9H). **^{13}C NMR** (126 MHz, Chloroform-*d*) δ 168.24, 160.81, 142.74, 141.53, 140.04, 139.13, 137.49, 135.68, 133.45, 131.07, 128.73, 128.39, 125.20, 122.74, 122.44, 120.48, 115.22, 112.95, 82.07, 27.73, 21.71, 16.54. HRMS(ESI) m/z: calculated for $[C_{26}H_{27}NO_3+H]^+$ 402.2064, found 402.2061. HPLC data (Chiralpak AD column, hexane: isopropanol = 95:5, 1.0 mL/min), tr =18.0 min (major), tr = 23.9 min (minor), ee = 89%.

***tert*-butyl (*P*, *Z*)-3-(3-chlorophenyl)-2-methyl-3-(2-oxoquinolin-1(2*H*)-yl)acrylate [(*P*, *Z*)-3h]**

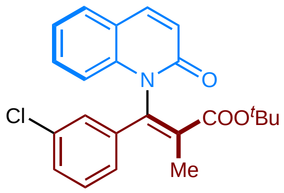

(P, Z)-**3h**

Following the general procedure of **D**, (P, Z)-**3h** was obtained as white solid (81% yield). **^{1}H NMR** (500 MHz, Chloroform-d) δ 7.78 (d, J = 9.5 Hz, 1H), 7.61 (d, J = 7.6 Hz, 1H), 7.54 (t, J = 7.8 Hz, 1H), 7.47 (s, 1H), 7.39 (t, J = 7.3 Hz, 3H), 7.32 (d, J = 3.2 Hz, 2H), 7.26 (t, J = 7.5 Hz, 1H), 6.74 (d, J = 9.5 Hz, 1H), 2.31 (s, 3H), 1.06 (s, 9H). **^{13}C NMR** (126 MHz, Chloroform-d) δ 165.39, 160.36, 139.04, 138.97, 136.75, 134.71, 133.19, 132.70, 129.78, 128.54, 128.12, 128.02, 127.49, 126.57, 121.53, 121.32, 119.34, 114.50, 80.74, 26.32, 16.49. HRMS(ESI) m/z: calculated for $[C_{23}H_{22}ClNO_3+H]^+$ 396.1361, found 396.1362. HPLC data (Chiralpak AD column, hexane: isopropanol = 90: 10, 1.0 mL/min), tr = 9.3 min (minor), tr = 23.0 min (major), ee = 89%.

***tert*-butyl (*P*, *E*)-3-(3-chlorophenyl)-2-methyl-3-(2-oxoquinolin-1(2*H*)-yl)acrylate [(*P*, *E*)-3h]**

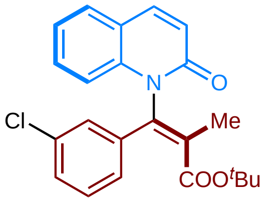

(P, E)-**3h**

Following the general procedure of **F**, (P, E)-**3h** was obtained as white solid (78% yield). E/Z= 6/1. **^{1}H NMR** (500 MHz, Chloroform-d) δ 7.75 (d, J = 9.6 Hz, 1H), 7.60 (dd, J = 7.8, 1.5 Hz, 1H), 7.54 (ddd, J = 8.6, 7.1, 1.5 Hz, 1H), 7.45 (t, J = 2.0 Hz, 1H), 7.40 (d, J = 8.4 Hz, 1H), 7.27 – 7.24 (m, 3H), 7.20 – 7.17 (m, 1H), 6.69 (d, J = 9.5 Hz, 1H), 1.77 (s, 3H), 1.37 (s, 9H). **^{13}C NMR** (126 MHz, Chloroform-d) δ 167.75, 160.78, 140.25, 138.95, 138.55, 136.23, 134.90, 133.94, 131.23, 129.43, 129.43, 128.92, 128.79, 126.51, 122.93, 122.31, 120.51, 114.92, 82.45, 27.71, 16.54. HRMS(ESI) m/z: calculated for $[C_{23}H_{22}ClNO_3+H]^+$ 396.1361, found 396.1362. HPLC data (Chiralpak AD column, hexane: isopropanol = 90:10, 1.0 mL/min), tr = 9.2 min (major), tr = 10.2 min (minor), ee = 89%.

tert-butyl (P, Z)-3-(3-fluorophenyl)-2-methyl-3-(2-oxoquinolin-1(2H)-yl)acrylate [(P, Z)-3i]

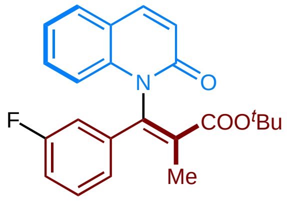

(*P, Z*)-**3i**

Following the general procedure of **D**, (*P*, *Z*)-**3i** was obtained as white solid (74% yield). ^{1}**H NMR** (500 MHz, Chloroform-*d*) δ 7.73 (d, *J* = 9.6 Hz, 1H), 7.56 (dd, *J* = 7.8, 1.5 Hz, 1H), 7.48 (ddd, *J* = 8.7, 7.2, 1.5 Hz, 1H), 7.34 (d, *J* = 8.5 Hz, 1H), 7.31 – 7.28 (m, 1H), 7.26 – 7.24 (m, 1H), 7.21 (td, *J* = 7.5, 1.0 Hz, 1H), 7.17 – 7.14 (m, 1H), 7.00 (tdd, *J* = 8.3, 2.7, 1.2 Hz, 1H), 6.69 (d, *J* = 9.5 Hz, 1H), 2.27 (s, 3H), 1.02 (s, 9H). ^{13}**C NMR** (126 MHz, Chloroform-d) δ 165.47, 161.40 (d, *J* = 254.4 Hz), 160.45, 139.08, 138.95, 137.07 (d, *J* = 7.6 Hz), 134.78, 132.59, 129.75, 128.82 (d, *J* = 8.2 Hz), 127.47, 124.10 (d, *J* = 3.1 Hz), 121.43 (d, *J* = 22.5 Hz), 119.34, 115.32, 115.15, 114.90 (d, *J* = 21.2 Hz), 114.55, 80.73, 26.33, 16.46. ^{19}**F NMR** (470 MHz, Chloroform-d) δ -112.49 (td, *J* = 9.2, 5.8 Hz). HRMS(ESI) m/z: calculated for $[C_{23}H_{22}FNO_3+H]^+$ 380.1656, found 380.1660. HPLC data (Chiralpak AD column, hexane: isopropanol = 90:10, 1.0 mL/min), tr = 10.0 min (minor), tr = 22.9 min (major), ee = 90%.

tert-butyl (P, E)-3-(3-fluorophenyl)-2-methyl-3-(2-oxoquinolin-1(2H)-yl)acrylate [(P, E)-3i]

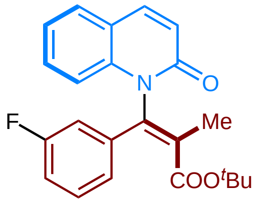

(*P, E*)-**3i**

Following the general procedure of **F**, (*P*, *E*)-**3i** was obtained as white solid (74% yield). *E*/*Z*= 6/1. ^{1}**H NMR** (500 MHz, Chloroform-*d*) δ 7.75 (d, *J* = 9.6 Hz, 1H), 7.60 (dd, *J* = 7.8, 1.6 Hz, 1H), 7.53 (ddd, *J* = 8.7, 7.2, 1.5 Hz, 1H), 7.40 (d, *J* = 8.2 Hz, 1H), 7.27 – 7.18 (m, 3H), 7.13 (ddd, *J* = 9.9, 2.6, 1.6 Hz, 1H), 6.98 (tdd, *J* = 8.2, 2.6, 1.3 Hz, 1H), 6.69 (d, *J* = 9.5 Hz, 1H), 1.77 (s, 3H), 1.36 (s, 9H). ^{13}**C NMR** (126 MHz, Chloroform-

d) δ 167.86, 162.39 (d, *J* = 246.0 Hz), 160.81, 140.22, 138.99, 138.82 (d, *J* = 8.0 Hz), 136.17 (d, *J* = 2.6 Hz), 134.79, 131.20, 129.63 (d, *J* = 8.3 Hz), 128.88, 124.32 (d, *J* = 2.9 Hz), 122.91, 122.33, 115.71 (d, *J* = 5.6 Hz), 115.54 (d, *J* = 7.2 Hz), 114.96, 82.39, 27.68, 16.54. **^{19}F NMR** (470 MHz, Chloroform-*d*) δ -113.27. HRMS(ESI) m/z: calculated for $[C_{23}H_{22}FNO_3+H]^+$ 380.1656, found 380.1652. HPLC data (Chiralpak AD column, hexane: isopropanol = 90:10, 1.0 mL/min), tr = 9.4 min (major), tr = 10.6 min (minor), ee = 90%.

***tert*-butyl (*P, Z*)-2-methyl-3-(2-oxoquinolin-1(2*H*)-yl)-3-(*m*-tolyl)acrylate [(*P, Z*)-3j]**

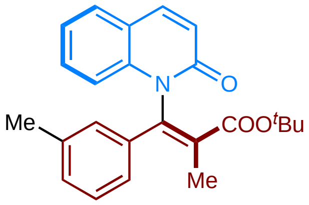

(*P, Z*)-**3j**

Following the general procedure of **D**, (*P, Z*)-**3j** was obtained as white solid (83% yield). **^{1}H NMR** (500 MHz, Chloroform-*d*) δ 7.71 (d, *J* = 9.5 Hz, 1H), 7.54 (dd, *J* = 7.7, 1.5 Hz, 1H), 7.46 (ddd, *J* = 8.6, 7.1, 1.5 Hz, 1H), 7.38 (d, *J* = 8.5 Hz, 1H), 7.27 – 7.16 (m, 4H), 7.10 (d, *J* = 7.5 Hz, 1H), 6.69 (d, *J* = 9.5 Hz, 1H), 2.29 (s, 3H), 2.27 (s, 3H), 1.02 (s, 9H).**^{13}C NMR** (126 MHz, Chloroform-*d*) δ 165.83, 160.42, 139.27, 138.71, 136.88, 136.30, 134.86, 131.40, 129.59, 128.80, 128.69, 127.28, 127.06, 125.35, 121.46, 121.27, 119.30, 114.85, 80.38, 26.36, 20.46, 16.56. HRMS(ESI) m/z: calculated for $[C_{24}H_{25}NO_3+H]^+$ 376.1907, found 376.1911. HPLC data (Chiralpak AD column, hexane: isopropanol = 90:10, 1.0 mL/min), tr =8.7 min (minor), tr = 19.4 min (major), ee = 92 %.

***tert*-butyl (*P, E*)-2-methyl-3-(2-oxoquinolin-1(2*H*)-yl)-3-(*m*-tolyl)acrylate [(*P, E*)-3j]**

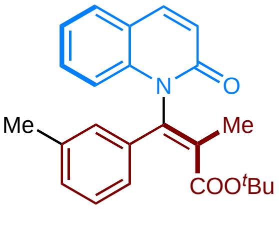

(*P, E*)-**3j**

Following the general procedure of **F**, (*P, E*)-**3j** was obtained as white solid (81% yield). *E*/*Z*= 6/1. **^{1}H NMR** (500 MHz, Chloroform-*d*) δ 7.74 (d, *J* = 9.5 Hz, 1H), 7.59 – 7.57

(m, 1H), 7.51 (ddd, J = 8.6, 7.1, 1.5 Hz, 1H), 7.44 (d, J = 8.5 Hz, 1H), 7.26 – 7.22 (m, 2H), 7.15 – 7.12 (m, 2H), 7.08 – 7.07 (m, 1H), 6.70 (d, J = 9.5 Hz, 1H), 2.28 (s, 3H), 1.76 (s, 3H), 1.34 (s, 9H). **^{13}C NMR** (126 MHz, Chloroform-*d*) δ 168.38, 160.82, 139.97, 139.19, 137.79, 137.54, 136.63, 133.43, 131.03, 129.55, 129.36, 128.69, 128.02, 125.36, 122.67, 122.49, 120.48, 115.30, 81.89, 27.68, 21.41, 16.50. HRMS(ESI) m/z: calculated for $[C_{24}H_{25}NO_3+H]^+$ 376.1907, found 376.1905. HPLC data (Chiralpak OD column, hexane: isopropanol = 90:10, 1.0 mL/min), tr =8.9 min (minor), tr = 9.7 min (major), ee = 92 %.

***tert*-butyl (*P*, *Z*)-2-methyl-3-(naphthalen-2-yl)-3-(2-oxoquinolin-1(2*H*)-yl)acrylate [(*P*, *Z*)-3k]**

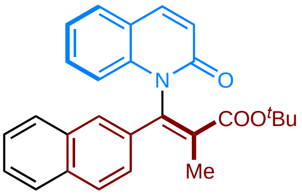

(*P*, *Z*)-**3k**

Following the general procedure of **D**, (*P*, *Z*)-**3k** was obtained as white solid (92% yield). **^{1}H NMR** (500 MHz, Chloroform-*d*) δ 7.98 (d, J = 1.8 Hz, 1H), 7.83 (t, J = 5.9 Hz, 3H), 7.79 (d, J = 9.5 Hz, 1H), 7.63 – 7.60 (m, 2H), 7.53 – 7.49 (m, 4H), 7.24 (ddd, J = 8.0, 6.3, 2.0 Hz, 1H), 6.77 (d, J = 9.5 Hz, 1H), 2.40 (s, 3H), 1.10 (s, 9H). **^{13}C NMR** (126 MHz, Chloroform-*d*) δ 165.76, 160.51, 139.27, 138.85, 136.21, 132.44, 132.19, 131.92, 131.79, 129.66, 128.03, 127.39, 127.36, 126.93, 126.60, 125.83, 125.51, 125.34, 121.43, 121.38, 119.35, 114.86, 80.51, 26.39, 16.63. HRMS(ESI) m/z: calculated for $[C_{27}H_{25}NO_3+H]^+$ 412.1907, found 412.1902. HPLC data (Chiralpak AD column, hexane: isopropanol = 70:30, 1.0 mL/min), tr =5.7 min (minor), tr = 15.8 min (major), ee = 94%.

***tert*-butyl (*P*, *E*)-2-methyl-3-(naphthalen-2-yl)-3-(2-oxoquinolin-1(2*H*)-yl)acrylate [(*P*, *E*)-3k]**

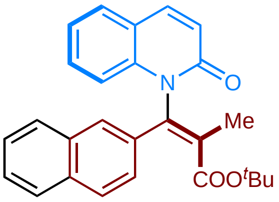

(*P*, *E*)-**3k**

Following the general procedure of **F**, (*P*, *E*)-**3k** was obtained as white solid (67% yield). *E*/*Z*= 3/1. **^{1}H NMR** (500 MHz, Chloroform-*d*) δ 7.88 (d, *J* = 1.9 Hz, 1H), 7.74 (ddd, *J* = 11.3, 8.9, 4.8 Hz, 4H), 7.58 (d, *J* = 7.8 Hz, 1H), 7.51 – 7.48 (m, 3H), 7.43 – 7.41 (m, 2H), 7.23 (dt, *J* = 8.0, 4.1 Hz, 1H), 6.72 (d, *J* = 9.5 Hz, 1H), 1.83 (s, 3H), 1.27 (s, 9H). **^{13}C NMR** (126 MHz, Chloroform-*d*) δ 168.33, 160.91, 140.14, 139.17, 137.68, 134.20, 134.09, 133.28, 132.86, 131.10, 128.78, 128.31, 128.06, 127.78, 127.62, 126.54, 126.26, 126.01, 122.79, 122.44, 120.54, 115.30, 82.10, 27.70, 16.65. HRMS(ESI) m/z: calculated for $[C_{27}H_{25}NO_3+H]^+$ 412.1907, found 412.1907. HPLC data (Chiralpak AD column, hexane: isopropanol = 90:10, 1.0 mL/min), tr =18.4 min (minor), tr = 20.2 min (major), ee = 93%.

***tert*-butyl (*P*, *Z*)-2-methyl-3-(2-oxoquinolin-1(2*H*)-yl)-3-(thiophen-2-yl)acrylate [(*P*, *Z*)-3l]**

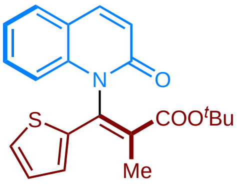

(*P*, *Z*)-**3l**

Following the general procedure of **D**, (*P*, *Z*)-**3l** was obtained as yellow solid (47% yield). **^{1}H NMR** (500 MHz, Chloroform-*d*) δ 7.80 (d, *J* = 9.5 Hz, 1H), 7.61 (d, *J* = 7.7 Hz, 1H), 7.50 – 7.46 (m, 1H), 7.41 – 7.39 (m, 1H), 7.27 – 7.22 (m, 2H), 7.14 (d, *J* = 3.3 Hz, 1H), 7.04 – 7.02 (m, 1H), 6.78 (d, *J* = 9.5 Hz, 1H), 2.50 (s, 3H), 1.08 (s, 9H). **^{13}C NMR** (126 MHz, Chloroform-*d*) δ 166.60, 161.27, 140.02, 138.23, 131.62, 131.39, 130.67, 129.51, 128.29, 128.00, 127.17, 122.48, 122.30, 120.26, 115.78, 81.67, 27.43 , 17.83. HRMS(ESI) m/z: calculated for $[C_{21}H_{21}NO_3S + Na]^+$ 390.1134, found 390.1125. HPLC data (Chiralpak AD column, hexane: isopropanol = 90:10, 1.0 mL/min), tr =12.4 min (minor), tr = 29.8 min (major), ee = 89%.

***tert*-butyl (*P*, *E*)-2-methyl-3-(2-oxoquinolin-1(2*H*)-yl)-3-(thiophen-2-yl)acrylate [(*P*, *E*)-3l]**

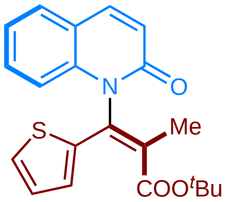

(*P*, *E*)-**3l**

Following the general procedure of **F**, (*P*, *E*)-**3l** was obtained as white solid (65% yield). *E*/*Z*= 5/1. **^{1}H NMR** (500 MHz, Chloroform-*d*) δ 7.74 (d, *J* = 9.6 Hz, 1H), 7.58 (dd, *J* = 7.9, 1.5 Hz, 1H), 7.51 (ddd, *J* = 8.6, 7.2, 1.5 Hz, 1H), 7.39 (d, *J* = 8.5 Hz, 1H), 7.25 – 7.22 (m, 2H), 7.15 (dd, *J* = 3.7, 1.2 Hz, 1H), 6.91 (dd, *J* = 5.1, 3.7 Hz, 1H), 6.72 (d, *J* = 9.6 Hz, 1H), 1.75 (s, 3H), 1.44 (s, 9H). **^{13}C NMR** (126 MHz, Chloroform-*d*) δ 167.94, 160.61, 140.18, 138.63, 137.81, 134.22, 131.09, 130.42, 128.75, 128.52, 127.00, 126.56, 122.85, 122.23, 120.49, 114.99, 82.44, 27.84, 16.39. HRMS(ESI) m/z: calculated for $[C_{21}H_{21}NO_3S + H]^+$ 368.1315, found 368.1317. HPLC data (Chiralpak OD column, hexane: isopropanol = 95:5, 1.0 mL/min), tr =22.2 min (minor), tr = 30.5 min (major), ee = 89%.

***tert*-butyl (*P*, *Z*)-3-(benzo[*b*]thiophen-2-yl)-2-methyl-3-(2-oxoquinolin-1(2*H*)-yl)acrylate [(*P*, *Z*)-3m]**

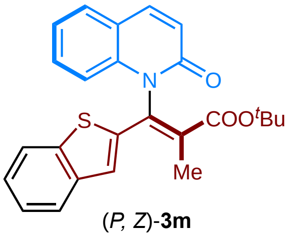

(*P*, *Z*)-**3m**

Following the general procedure of **D**, (*P*, *Z*)-**3m** was obtained as yellow solid (56% yield). **^{1}H NMR** (500 MHz, Chloroform-*d*) δ 7.77 (d, *J* = 9.6 Hz, 1H), 7.74 – 7.72 (m, 1H), 7.70 – 7.68 (m, 1H), 7.57 (dd, *J* = 7.8, 1.5 Hz, 1H), 7.45 (ddd, *J* = 8.6, 7.2, 1.5 Hz, 1H), 7.36 (s, 1H), 7.32 – 7.28 (m, 3H), 7.20 (td, *J* = 7.5, 1.1 Hz, 1H), 6.75 (d, *J* = 9.6 Hz, 1H), 2.52 (s, 3H), 1.05 (s, 9H). **^{13}C NMR** (126 MHz, Chloroform-*d*) δ 165.32, 160.24, 139.35, 139.05, 138.91, 137.88, 137.06, 132.83, 130.46, 129.67, 127.34, 125.33, 124.28, 123.57, 123.17, 121.53, 121.24, 120.97, 119.26, 114.71, 80.85, 26.39,

16.76. HRMS(ESI) m/z: calculated for $[C_{25}H_{23}NO_3S +H]^+$ 418.1471, found 418.1469. HPLC data (Chiralpak AD column, hexane: isopropanol = 85:15, 1.0 mL/min), tr =17.2 min (minor), tr = 31.8 min (major), ee = 91%.

***tert*-butyl (*P*, *E*)-3-(benzo[*b*]thiophen-2-yl)-2-methyl-3-(2-oxoquinolin-1(2*H*)-yl)acrylate [(*P*, *E*)-3m]**

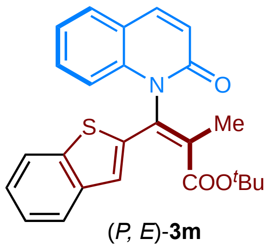

(*P*, *E*)-**3m**

Following the general procedure of **F**, (*P*, *E*)-**3m** was obtained as white solid (61% yield). *E*/*Z*= 4/1. **^{1}H NMR** (500 MHz, Chloroform-*d*) δ 7.76 (d, *J* = 9.6 Hz, 1H), 7.67 (ddd, *J* = 13.0, 6.7, 2.5 Hz, 2H), 7.60 – 7.58 (m, 1H), 7.54 – 7.51 (m, 1H), 7.43 (d, *J* = 8.4 Hz, 1H), 7.39 (s, 1H), 7.30 – 7.23 (m, 3H), 6.74 (d, *J* = 9.5 Hz, 1H), 1.81 (s, 3H), 1.41 (s, 9H). **^{13}C NMR** (126 MHz, Chloroform-*d*) δ 167.77, 160.69, 140.30, 138.89, 138.65, 138.06, 135.97, 131.15, 130.50, 128.83, 125.38, 124.99, 124.99, 124.43, 123.87, 122.96, 122.21, 122.06, 120.53, 114.95, 82.71, 27.82, 16.49. HRMS(ESI) m/z: calculated for $[C_{25}H_{23}NO_3S +H]^+$ 418.1471, found 418.1472. HPLC data (Chiralpak OD column, hexane: isopropanol = 90:10, 1.0 mL/min), tr =12.6 min (minor), tr = 25.1 min (major), ee = 91%.

***tert*-butyl (*P*, *Z*)-2-methyl-3-(4-methyl-2-oxoquinolin-1(2*H*)-yl)-3-phenylacrylate [(*P*, *Z*)-3n]**

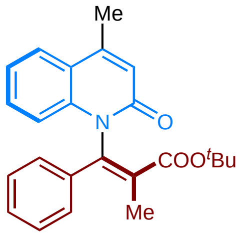

(*P*, *Z*)-**3n**

Following the general procedure of **D**, (*P*, *Z*)-**3n** was obtained as white solid (78% yield). **^{1}H NMR** (500 MHz, Chloroform-*d*) δ 7.68 (dd, *J* = 8.1, 1.4 Hz, 1H), 7.45 (dt, *J* = 7.9, 1.7 Hz, 3H), 7.38 (dd, *J* = 8.5, 1.1 Hz, 1H), 7.31 (s, 1H), 7.30 – 7.27 (m, 2H), 7.21 (ddd, *J* = 8.2, 7.1, 1.2 Hz, 1H), 6.59 (d, *J* = 1.3 Hz, 1H), 2.48 (s, 3H), 2.27 (s, 3H),

1.02 (s, 9H). **^{13}C NMR** (126 MHz, Chloroform-*d*) δ 165.74, 160.13, 146.23, 138.91, 136.23, 135.16, 131.58, 129.37, 128.26, 127.73, 127.18, 123.78, 121.11, 120.65, 120.01, 115.10, 80.34, 26.34, 18.05, 16.47. HRMS(ESI) m/z: calculated for $[C_{24}H_{25}NO_3+H]^+$ 376.1907, found 376.1913. HPLC data (Chiralpak AD column, hexane: isopropanol = 80:20, 1.0 mL/min), tr =5.6 min (minor), tr = 13.1 min (major), ee = 95%.

***tert*-butyl (*P*, *E*)-2-methyl-3-(4-methyl-2-oxoquinolin-1(2*H*)-yl)-3-phenylacrylate [(*P*, *E*)-3n]**

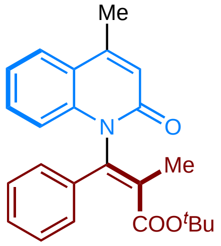

(*P*, *E*)-**3n**

Following the general procedure of **F**, (*P*, *E*)-**3n** was obtained as white solid (80% yield). *E*/*Z*= 9/1. **^{1}H NMR** (500 MHz, Chloroform-*d*) δ 7.71 (dd, *J* = 8.1, 1.4 Hz, 1H), 7.52 – 7.49 (m, 1H), 7.45 (dd, *J* = 8.4, 1.3 Hz, 1H), 7.41 – 7.39 (m, 2H), 7.27 – 7.24 (m, 4H), 6.59 (d, *J* = 1.3 Hz, 1H), 2.48 (s, 3H), 1.77 (s, 3H), 1.32 (s, 9H). **^{13}C NMR** (126 MHz, Chloroform-*d*) δ 168.28, 160.55, 147.65, 138.75, 137.78, 136.94, 133.71, 130.85, 128.60, 128.58, 128.04, 125.26, 122.54, 121.70, 121.22, 115.50, 81.94, 27.66, 19.18, 16.50. HRMS(ESI) m/z: calculated for $[C_{24}H_{25}NO_3+H]^+$ 376.1907, found 376.1904. HPLC data (Chiralpak AD column, hexane: isopropanol = 95:5, 1.0 mL/min), tr =19.6 min (major), tr = 25.4 min (minor), ee = 95%.

***tert*-butyl (*P*, *Z*)-2-methyl-3-(2-oxo-4-((*p*-tolylthio)methyl)quinolin-1(2*H*)-yl)-3-phenylacrylate [(*P*, *Z*)-3o]**

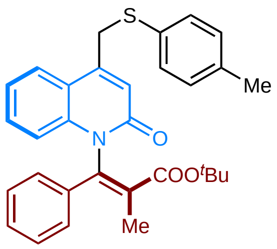

(*P*, *Z*)-**3o**

Following the general procedure of **D**, (*P*, *Z*)-**3o** was obtained as yellow solid (74% yield). **^{1}H NMR** (500 MHz, Chloroform-*d*) δ 7.86 (d, *J* = 8.0 Hz, 1H), 7.53 (t, *J* = 7.9

Hz, 1H), 7.46 (t, J = 7.2 Hz, 3H), 7.37 (q, J = 6.9 Hz, 3H), 7.29 (t, J = 6.7 Hz, 3H), 7.13 (d, J = 7.6 Hz, 2H), 6.58 (s, 1H), 4.23 (s, 2H), 2.36 (s, 3H), 2.32 (s, 3H), 1.07 (s, 9H). **^{13}C NMR** (126 MHz, Chloroform-*d*) δ 166.71, 160.76, 145.99, 140.37, 137.78, 136.92, 135.98, 132.86, 131.83, 131.15, 130.59, 129.97, 129.30, 128.85, 128.25, 124.99, 122.24, 122.10, 119.20, 116.53, 81.47, 37.34, 27.46, 21.14, 17.53. HRMS(ESI) m/z: calculated for $[C_{31}H_{31}NO_3S + H]^+$ 498.2097, found 498.2105. HPLC data (Chiralpak AD column, hexane: isopropanol = 70:30, 1.0 mL/min), tr =5.8 min (minor), tr = 28.4 min (major), ee = 95%.

***tert*-butyl (*P*, *E*)-2-methyl-3-(2-oxo-4-((*p*-tolylthio)methyl)quinolin-1(2*H*)-yl)-3-phenylacrylate [(*P*, *E*)-3o]**

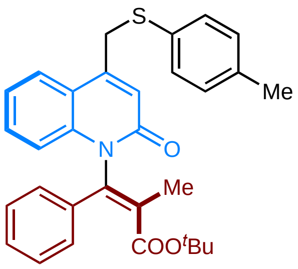

(*P*, *E*)-**3o**

Following the general procedure of **F**, (*P*, *E*)-**3o** was obtained as white solid (69% yield). *E*/*Z*= 6/1. **^{1}H NMR** (500 MHz, Chloroform-*d*) δ 7.86 (d, J = 8.0 Hz, 1H), 7.52 (t, J = 7.6 Hz, 1H), 7.45 (d, J = 8.4 Hz, 1H), 7.37 (dd, J = 7.3, 2.4 Hz, 2H), 7.26 (q, J = 4.5 Hz, 4H), 7.19 (d, J = 7.9 Hz, 2H), 7.05 (d, J = 7.8 Hz, 2H), 6.37 (s, 1H), 4.15 (s, 2H), 2.30 (s, 3H), 1.74 (s, 3H), 1.32 (s, 9H). **^{13}C NMR** (126 MHz, Chloroform-*d*) δ 168.23, 160.05, 146.16, 139.24, 138.25, 137.61, 136.72, 133.73, 132.79, 131.05, 130.42, 129.96, 128.70, 128.55, 128.08, 125.47, 122.62, 122.14, 119.22, 115.79, 82.06, 37.63, 27.67, 21.19, 16.47. HRMS(ESI) m/z: calculated for $[C_{31}H_{31}NO_3S + H]^+$ 498.2097, found 498.2093. HPLC data (Chiralpak AD column, hexane: isopropanol = 85:15, 1.0 mL/min), tr =6.9 min (major), tr = 7.6 min (minor), ee = 89%.

***tert*-butyl (*P*, *Z*)-3-(4-(benzyloxy)-2-oxoquinolin-1(2*H*)-yl)-2-methyl-3-phenylacrylate [(*P*, *Z*)-3p]**

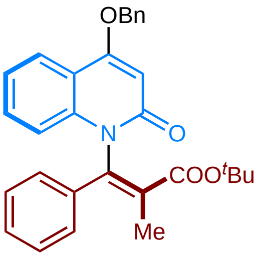

(*P*, *Z*)-**3p**

Following the general procedure of **D**, (*P*, *Z*)-**3p** was obtained as white solid (79% yield). **^{1}H NMR** (500 MHz, Chloroform-*d*) δ 8.01 (dd, *J* = 8.1, 1.6 Hz, 1H), 7.49 – 7.41 (m, 7H), 7.39 – 7.37 (m, 1H), 7.35 – 7.28 (m, 4H), 7.17 (ddd, *J* = 8.1, 7.1, 1.1 Hz, 1H), 6.10 (s, 1H), 5.18 (s, 2H), 2.27 (s, 3H), 1.03 (s, 9H). **^{13}C NMR** (126 MHz, Chloroform-*d*) δ 166.90, 162.83, 162.25, 139.89, 137.12, 136.30, 135.34, 132.83, 131.22, 129.29, 128.80, 128.77, 128.54, 128.23, 127.62, 123.22, 121.95, 116.23, 115.78, 98.05, 81.39, 70.54, 27.45, 17.56. HRMS(ESI) m/z: calculated for $[C_{30}H_{29}NO_4 + Na]^+$ 490.1989, found 490.1980. HPLC data (Chiralpak IA column, hexane: isopropanol = 85:15, 1.0 mL/min), tr = 9.5 min (minor), tr = 25.0 min (major), ee = 94%.

***tert*-butyl (*P*, *E*)-3-(4-(benzyloxy)-2-oxoquinolin-1(2*H*)-yl)-2-methyl-3-phenylacrylate [(*P*, *E*)-3p]**

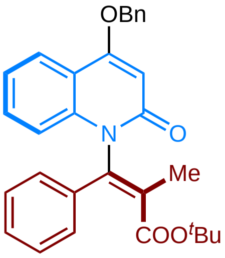

(*P*, *E*)-**3p**

Following the general procedure of **F**, (*P*, *E*)-**3p** was obtained as white solid (82% yield). *E*/*Z*= 8/1. **^{1}H NMR** (500 MHz, Chloroform-*d*) δ 8.03 (dd, *J* = 8.0, 1.5 Hz, 1H), 7.52 (ddd, *J* = 8.7, 7.1, 1.5 Hz, 1H), 7.49 – 7.47 (m, 2H), 7.45 – 7.37 (m, 6H), 7.27 – 7.24 (m, 3H), 7.23 – 7.20 (m, 1H), 6.12 (s, 1H), 5.20 – 5.14 (m, 2H), 1.79 (s, 3H), 1.33 (s, 9H). **^{13}C NMR** (126 MHz, Chloroform-*d*) δ 168.33, 162.45, 162.25, 138.73, 137.68, 137.06, 135.25, 133.92, 131.66, 128.84, 128.63, 128.58, 128.55, 128.04, 127.81, 123.65, 122.31, 115.14, 97.91, 81.95, 70.72, 27.68, 16.52. HRMS(ESI) m/z: calculated for $[C_{30}H_{29}NO_4 + H]^+$ 468.2169, found 468.2165. HPLC data (Chiralpak AD column,

hexane: isopropanol = 90:10, 1.0 mL/min), tr = 15.4 min (major), tr = 29.7 min (minor), ee = 94%.

***tert*-butyl (*P*, *Z*)-2-methyl-3-(2-oxo-4-(piperidin-1-yl)quinolin-1(2*H*)-yl)-3-phenylacrylate [(*P*, *Z*)-3q]**

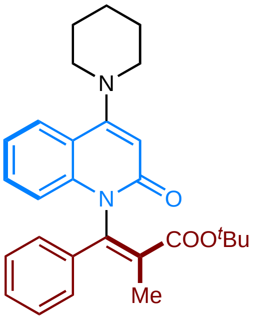

(*P*, *Z*)-**3q**

Following the general procedure of **D**, (*P*, *Z*)-**3q** was obtained as white solid (65% yield). ^{1}**H NMR** (500 MHz, Chloroform-*d*) δ 7.99 (d, *J* = 8.1 Hz, 1H), 7.50 (d, *J* = 7.0 Hz, 3H), 7.44 (d, *J* = 8.6 Hz, 1H), 7.35 (d, *J* = 8.2 Hz, 3H), 7.25 (d, *J* = 7.8 Hz, 1H), 6.84 (s, 1H), 3.90 (q, *J* = 14.7 Hz, 2H), 2.71 (s, 4H), 2.32 (s, 3H), 1.87 (s, 4H), 1.07 (s, 9H). ^{13}**C NMR** (126 MHz, Chloroform-*d*) δ 166.85, 161.41, 148.12, 140.10, 137.12, 136.11, 132.74, 130.27, 129.33, 128.79, 128.23, 125.05, 122.12, 121.21, 120.09, 116.15, 81.38, 57.55, 54.53, 27.41, 23.74, 17.54. HRMS(ESI) m/z: calculated for $[C_{28}H_{32}N_2O_3+H]^+$ 445.2486, found 445.2482. HPLC data (Chiralpak AD column, hexane: isopropanol = 70:30, 1.0 mL/min), tr = 5.5 min (minor), tr = 21.4 min (major), ee = 96%.

***tert*-butyl (*P*, *E*)-2-methyl-3-(2-oxo-4-(piperidin-1-yl)quinolin-1(2*H*)-yl)-3-phenylacrylate [(*P*, *E*)-3q]**

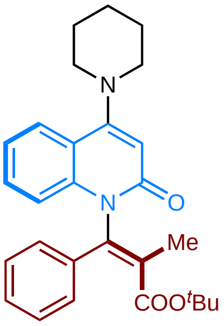

(*P*, *E*)-**3q**

Following the general procedure of **F**, (*P*, *E*)-**3q** was obtained as white solid (69%

yield). *E*/*Z*= 10/1. **^{1}H NMR** (500 MHz, Chloroform-d) δ 7.97 (dd, *J* = 8.1, 1.5 Hz, 1H), 7.47 (dtd, *J* = 16.7, 8.4, 1.4 Hz, 2H), 7.42 – 7.40 (m, 2H), 7.26 – 7.23 (m, 4H), 6.77 (s, 1H), 3.85 – 3.78 (m, 2H), 2.61 (qd, *J* = 6.1, 5.6, 3.3 Hz, 4H), 1.80 (p, *J* = 3.4 Hz, 4H), 1.77 (s, 3H), 1.32 (s, 9H). **^{13}C NMR** (126 MHz, Chloroform-d) δ 168.24, 160.74, 148.35, 138.99, 137.84, 136.92, 133.72, 130.70, 128.63, 128.59, 128.02, 125.48, 122.48, 121.25, 120.24, 115.45, 81.95, 57.56, 54.60, 27.66, 23.74, 16.49. HRMS(ESI) m/z: calculated for $[C_{28}H_{32}N_2O_3+H]^+$ 445.2486, found 445.2466. HPLC data (Chiralpak AD column, hexane: isopropanol = 90:10, 1.0 mL/min), tr = 11.0 min (major), tr = 12.3 min (minor), ee = 95%.

methyl (*P*, *Z*)-1-(3-(*tert*-butoxy)-2-methyl-3-oxo-1-phenylprop-1-en-1-yl)-2-oxo-1,2-dihydroquinoline-4-carboxylate [(*P*, *Z*)-3r]

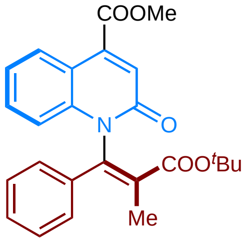

(*P*, *Z*)-**3r**

Following the general procedure of **D**, (*P*, *Z*)-**3r** was obtained as white solid (74% yield). **^{1}H NMR** (500 MHz, Chloroform-*d*) δ 8.30 (dd, *J* = 8.2, 1.5 Hz, 1H), 7.51 (ddd, *J* = 8.6, 7.1, 1.5 Hz, 1H), 7.45 – 7.42 (m, 3H), 7.35 – 7.30 (m, 3H), 7.26 – 7.24 (m, 1H), 7.18 (s, 1H), 3.99 (s, 3H), 2.28 (s, 3H), 1.03 (s, 9H). **^{13}C NMR** (126 MHz, Chloroform-*d*) δ 165.40, 164.78, 159.36, 139.57, 138.39, 135.87, 134.51, 131.86, 130.15, 128.22, 128.03, 127.32, 125.79, 123.90, 121.89, 116.10, 115.18, 80.69, 51.93, 26.38, 16.52. HRMS(ESI) m/z: calculated for $[C_{25}H_{25}NO_5+H]^+$ 420.1805, found 420.1812. HPLC data (Chiralpak AD column, hexane: isopropanol = 70:30, 1.0 mL/min), tr =4.9 min (minor), tr = 27.9 min (major), ee = 92%.

methyl (*P*, *E*)-1-(3-(*tert*-butoxy)-2-methyl-3-oxo-1-phenylprop-1-en-1-yl)-2-oxo-1,2-dihydroquinoline-4-carboxylate [(*P*, *E*)-3r]

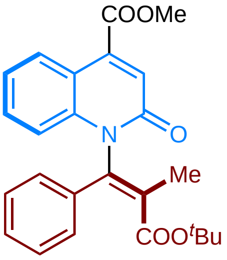

(*P*, *E*)-**3r**

Following the general procedure of **F**, (*P*, *E*)-**3r** was obtained as white solid (74% yield). *E*/*Z*= 8/1. **^{1}H NMR** (500 MHz, Chloroform-*d*) δ 8.33 (dd, *J* = 8.2, 1.4 Hz, 1H), 7.56 (ddd, *J* = 8.5, 7.0, 1.5 Hz, 1H), 7.51 (dd, *J* = 8.6, 1.3 Hz, 1H), 7.39 (dd, *J* = 7.5, 2.0 Hz, 2H), 7.31 – 7.25 (m, 4H), 7.19 (s, 1H), 4.00 (s, 3H), 1.77 (s, 3H), 1.33 (s, 9H). **^{13}C NMR** (126 MHz, Chloroform-*d*) δ 167.99, 165.77, 159.73, 139.60, 139.48, 137.28, 136.32, 134.02, 131.61, 128.87, 128.55, 128.15, 127.21, 125.03, 123.30, 117.30, 115.62, 82.18, 52.99, 27.66, 16.51. HRMS(ESI) m/z: calculated for $[C_{25}H_{25}NO_5+H]^+$ 420.1805, found 420.1801. HPLC data (Chiralpak AD column, hexane: isopropanol = 95:5, 1.0 mL/min), tr =18.2 min (major), tr = 28.4 min (minor), ee = 26%.

***tert*-butyl (*P*, *Z*)-3-(5-bromo-2-oxoquinolin-1(2*H*)-yl)-2-methyl-3-phenylacrylate [(*P*, *Z*)-3s]**

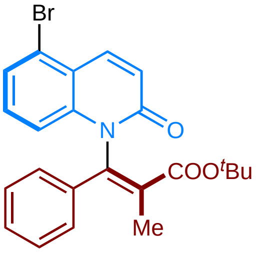

(*P*, *Z*)-**3s**

Following the general procedure of **D**, (*P*, *Z*)-**3s** was obtained as white solid (84% yield). **^{1}H NMR** (500 MHz, Chloroform-*d*) δ 8.21 (d, *J* = 9.9 Hz, 1H), 7.48 – 7.47 (m, 3H), 7.41 – 7.37 (m, 2H), 7.35 (d, *J* = 7.3 Hz, 2H), 7.32 (d, *J* = 7.4 Hz, 1H), 6.82 (d, *J* = 9.9 Hz, 1H), 2.32 (s, 3H), 1.12 (s, 9H). **^{13}C NMR** (126 MHz, Chloroform-*d*) δ 166.54, 160.95, 141.42, 138.40, 136.82, 135.61, 133.09, 130.92, 129.25, 129.04, 128.37, 126.54, 123.60, 123.22, 119.42, 115.67, 81.69, 27.49, 17.57. HRMS(ESI) m/z: calculated for $[C_{23}H_{22}BrNO_3+H]^+$ 440.0856, found 440.0846. HPLC data (Chiralpak AD column, hexane: isopropanol = 85:15, 1.0 mL/min), tr =7.2 min (minor), tr = 14.4

min (major), ee = 96%.

***tert*-butyl (*P, E*)-3-(5-bromo-2-oxoquinolin-1(2*H*)-yl)-2-methyl-3-phenylacrylate [(*P, E*)-3s]**

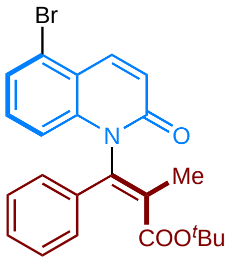

(*P, E*)-**3s**

Following the general procedure of **F**, (*P*, *E*)-**3s** was obtained as white solid (81% yield). *E*/*Z*= 7/1. **^{1}H NMR** (500 MHz, Chloroform-*d*) δ 8.18 (d, *J* = 9.9 Hz, 1H), 7.48 (dd, *J* = 7.8, 1.1 Hz, 1H), 7.42 (d, *J* = 8.5 Hz, 1H), 7.39 – 7.37 (m, 2H), 7.35 – 7.32 (m, 1H), 7.28 – 7.25 (m, 3H), 6.78 (d, *J* = 9.9 Hz, 1H), 1.77 (s, 3H), 1.33 (s, 9H). **^{13}C NMR** (126 MHz, Chloroform-*d*) δ 168.02, 160.26, 140.33, 138.62, 137.40, 136.40, 134.03, 131.41, 128.85, 128.52, 128.16, 126.97, 123.67, 123.60, 119.61, 114.92, 82.18, 27.66, 16.49. HRMS(ESI) m/z: calculated for $[C_{23}H_{22}BrNO_3+H]^+$ 440.0856, found 440.0851. HPLC data (Chiralpak AD column, hexane: isopropanol = 95:5, 1.0 mL/min), tr =17.6 min (major), tr = 19.6 min (minor), ee = 97%.

***tert*-butyl (*P, Z*)-3-(6-bromo-2-oxoquinolin-1(2*H*)-yl)-2-methyl-3-phenylacrylate [(*P, Z*)-3t]**

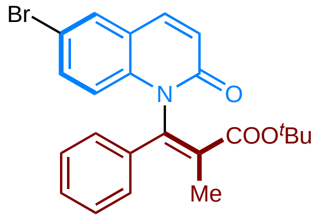

(*P, Z*)-**3t**

Following the general procedure of **D**, (*P*, *Z*)-**3t** was obtained as white solid (78% yield). **^{1}H NMR** (500 MHz, Chloroform-*d*) δ 7.60 (d, *J* = 2.2 Hz, 1H), 7.55 (d, *J* = 9.5 Hz, 1H), 7.45 (dd, *J* = 9.0, 2.2 Hz, 1H), 7.34 (d, *J* = 6.7 Hz, 2H), 7.24 (q, *J* = 6.5, 6.0 Hz, 3H), 7.19 – 7.16 (m, 1H), 6.65 (d, *J* = 9.5 Hz, 1H), 2.20 (s, 3H), 1.01 (s, 9H). **^{13}C NMR** (126 MHz, Chloroform-*d*) δ 166.58, 160.99, 139.15, 138.51, 136.50, 135.56, 133.29, 133.15,

130.45, 129.22, 129.06, 128.39, 123.70, 121.76, 117.70, 115.05, 81.67, 27.51, 17.54. HRMS(ESI) m/z: calculated for $[C_{23}H_{22}BrNO_3+Na]^+$ 462.0675, found 462.0672. HPLC data (Chiralpak AD column, hexane: isopropanol = 90:10, 1.0 mL/min), tr =9.6 min (minor), tr = 15.8 min (major), ee = 96%.

***tert*-butyl (*P, E*)-3-(6-bromo-2-oxoquinolin-1(2*H*)-yl)-2-methyl-3-phenylacrylate [(*P, E*)-3t]**

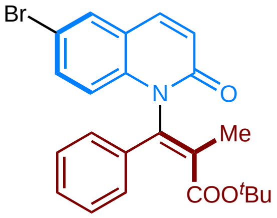

(*P, E*)-**3t**

Following the general procedure of **F**, (*P*, *E*)-**3t** was obtained as white solid (72% yield). *E*/*Z*= 5/1. **^{1}H NMR** (500 MHz, Chloroform-*d*) δ 7.71 (d, *J* = 2.3 Hz, 1H), 7.64 (d, *J* = 9.6 Hz, 1H), 7.58 (dd, *J* = 9.0, 2.3 Hz, 1H), 7.38 – 7.36 (m, 2H), 7.31 (d, *J* = 9.0 Hz, 1H), 7.28 – 7.25 (m, 3H), 6.72 (d, *J* = 9.6 Hz, 1H), 1.77 (s, 3H), 1.32 (s, 9H). **^{13}C NMR** (126 MHz, Chloroform-*d*) δ 168.00, 160.31, 138.75, 138.06, 137.22, 136.39, 134.03, 133.77, 130.89, 128.87, 128.49, 128.18, 123.73, 121.95, 116.99, 115.51, 82.20, 27.66, 16.50. HRMS(ESI) m/z: calculated for $[C_{23}H_{22}BrNO_3+H]^+$ 440.0856, found 440.0849. HPLC data (Chiralpak OD column, hexane: isopropanol =95:5, 1.0 mL/min), tr =24.2 min (minor), tr = 30.4 min (major), ee = 97%.

***tert*-butyl (*P, Z*)-3-(6-methoxy-2-oxoquinolin-1(2*H*)-yl)-2-methyl-3-phenylacrylate [(*P, Z*)-3u]**

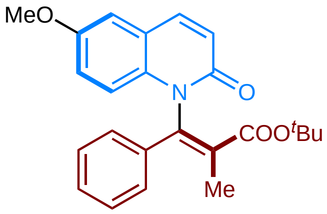

(*P, Z*)-**3u**

Following the general procedure of **D**, (*P*, *Z*)-**3u** was obtained as white solid (56 % yield). **^{1}H NMR** (500 MHz, Chloroform-*d*) δ 7.71 (d, *J* = 9.5 Hz, 1H), 7.48 (d, *J* = 6.7 Hz, 2H), 7.36 (q, *J* = 6.1 Hz, 4H), 7.15 (dd, *J* = 9.2, 2.7 Hz, 1H), 7.04 (d, *J* = 2.7 Hz,

1H), 6.79 (d, *J* = 9.5 Hz, 1H), 3.89 (s, 3H), 2.32 (s, 3H), 1.11 (s, 9H). **^{13}C NMR** (126 MHz, Chloroform-*d*) δ 161.05, 154.91, 139.30, 137.10, 135.98, 134.76, 132.71, 129.28, 128.86, 128.55, 128.27, 122.97, 121.02, 119.22, 117.25, 110.10, 81.50, 55.73, 27.47, 17.50. HRMS(ESI) m/z: calculated for $[C_{24}H_{25}NO_4+H]^+$ 392.1856, found 392.1856. HPLC data (Chiralpak IA column, hexane: isopropanol = 90:10, 1.0 mL/min), tr =14.6 min (minor), tr = 22.6 min (major), ee = 93%.

***tert*-butyl (*P*, *E*)-3-(6-methoxy-2-oxoquinolin-1(2*H*)-yl)-2-methyl-3-phenylacrylate [(*P*, *E*)-3u]**

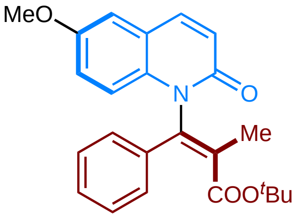

(*P*, *E*)-**3u**

Following the general procedure of **F**, (*P*, *E*)-**3u** was obtained as white solid (67% yield). *E*/*Z*= 5/1. **^{1}H NMR** (500 MHz, Chloroform-*d*) δ 7.67 (d, *J* = 9.6 Hz, 1H), 7.40 – 7.36 (m, 3H), 7.26 (dt, *J* = 5.0, 2.5 Hz, 3H), 7.13 (dd, *J* = 9.2, 2.9 Hz, 1H), 7.02 (d, *J* = 2.9 Hz, 1H), 6.70 (d, *J* = 9.5 Hz, 1H), 3.85 (s, 3H), 1.77 (s, 3H), 1.32 (s, 9H). **^{13}C NMR** (126 MHz, Chloroform-*d*) δ 168.23, 160.39, 155.15, 139.51, 136.80, 133.64, 133.52, 128.67, 128.55, 128.07, 123.02, 121.21, 119.58, 116.59, 110.50, 82.01, 55.74, 27.66, 16.49. HRMS(ESI) m/z: calculated for $[C_{24}H_{25}NO_4+H]^+$ 392.1856, found 392.1855. HPLC data (Chiralpak AD column, hexane: isopropanol = 95:5, 1.0 mL/min), tr =26.5min (major), tr = 30.3min (minor), ee = 94%.

***tert*-butyl (*P*, *Z*)-3-(7-bromo-2-oxoquinolin-1(2*H*)-yl)-2-methyl-3-phenylacrylate [(*P*, *Z*)-3v]**

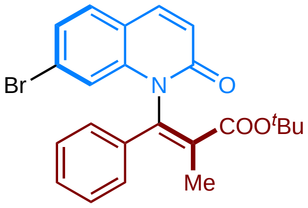

(*P*, *Z*)-**3v**

Following the general procedure of **D**, (*P*, *Z*)-**3v** was obtained as white solid (82 % yield). **^{1}H NMR** (500 MHz, Chloroform-*d*) δ 7.65 (d, *J* = 9.5 Hz, 1H), 7.51 (d, *J* = 1.7

Hz, 1H), 7.45 – 7.43 (m, 2H), 7.38 (d, *J* = 8.2 Hz, 1H), 7.36 – 7.27 (m, 4H), 6.69 (d, *J* = 9.5 Hz, 1H), 2.28 (s, 3H), 1.08 (s, 9H). ^{13}**C NMR** (126 MHz, Chloroform-*d*) δ 166.39, 161.13, 141.06, 139.15, 136.23, 135.52, 133.41, 129.46, 129.23, 129.04, 128.42, 125.58, 124.90, 122.73, 119.13, 118.90, 81.59, 27.47, 17.51. HRMS(ESI) m/z: calculated for $[C_{23}H_{22}BrNO_3+H]^+$ 440.0856, found 440.0860. HPLC data (Chiralpak AD column, hexane: isopropanol = 85:15, 1.0 mL/min), tr =6.6 min (minor), tr = 17.7 min (major), ee = 95%.

***tert*-butyl (*P*, *E*)-3-(7-bromo-2-oxoquinolin-1(2*H*)-yl)-2-methyl-3-phenylacrylate [(*P*, *E*)-3v]**

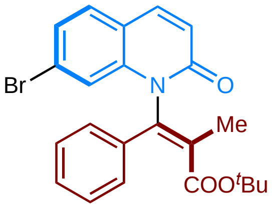

(*P*, *E*)-**3v**

Following the general procedure of **F**, (*P*, *E*)-**3v** was obtained as white solid (84% yield). *E*/*Z*= 9/1. ^{1}**H NMR** (500 MHz, Chloroform-*d*) δ 7.68 (dd, *J* = 9.6, 0.6 Hz, 1H), 7.57 (d, *J* = 1.8 Hz, 1H), 7.42 (d, *J* = 8.3 Hz, 1H), 7.40 – 7.38 (m, 2H), 7.34 (dd, *J* = 8.3, 1.8 Hz, 1H), 7.30 – 7.26 (m, 3H), 6.70 (d, *J* = 9.5 Hz, 1H), 1.79 (s, 3H), 1.32 (s, 9H). ^{13}**C NMR** (126 MHz, Chloroform-*d*) δ 167.92, 160.43, 140.03, 139.39, 137.09, 136.38, 134.24, 129.87, 128.87, 128.52, 128.23, 126.06, 125.46, 122.74, 119.26, 118.11, 82.17, 27.64, 16.51. HRMS(ESI) m/z: calculated for $[C_{23}H_{22}BrNO_3+H]^+$ 440.0856, found 440.0847. HPLC data (Chiralpak AD column, hexane: isopropanol = 95:5, 1.0 mL/min), tr =14.5 min (major), tr = 17.6 min (minor), ee = 95%.

***tert*-butyl (*P*, *Z*)-3-(7-methoxy-2-oxoquinolin-1(2*H*)-yl)-2-methyl-3-phenylacrylate [(*P*, *Z*)-3w]**

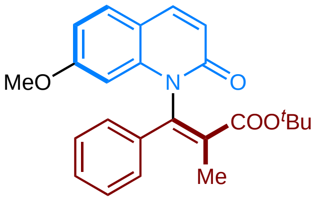

(*P*, *Z*)-**3w**

Following the general procedure of **D**, (*P*, *Z*)-**3w** was obtained as white solid (65%

yield). ^{1}**H NMR** (500 MHz, Chloroform-*d*) δ 7.63 (d, *J* = 9.6 Hz, 1H), 7.47 – 7.43 (m, 3H), 7.34 – 7.29 (m, 3H), 6.82 (d, *J* = 2.4 Hz, 1H), 6.77 (dd, *J* = 8.6, 2.4 Hz, 1H), 6.53 (d, *J* = 9.4 Hz, 1H), 3.81 (s, 3H), 2.28 (s, 3H), 1.06 (s, 9H). ^{13}**C NMR** (126 MHz, Chloroform-*d*) δ 166.82, 161.77, 141.88, 139.56, 137.19, 135.99, 132.63, 129.64, 129.29, 128.86, 128.52, 128.28, 119.22, 114.57, 110.53, 99.82, 81.48, 55.53, 27.43, 17.46. HRMS(ESI) m/z: calculated for $[C_{24}H_{25}NO_4+H]^+$ 392.1856, found 392.1856. HPLC data (Chiralpak AD column, hexane: isopropanol = 85:15, 1.0 mL/min), tr = 7.5 min (minor), tr = 23.9 min (major), ee = 95%.

***tert*-butyl (*P*, *E*)-3-(7-methoxy-2-oxoquinolin-1(2*H*)-yl)-2-methyl-3-phenylacrylate [(*P*, *E*)-3w]**

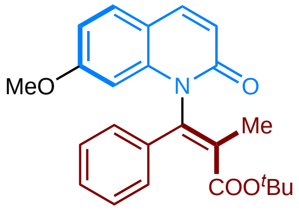

(*P*, *E*)-**3w**

Following the general procedure of **F**, (*P*, *E*)-**3w** was obtained as white solid (71% yield). *E*/*Z*= 7/1. ^{1}**H NMR** (500 MHz, Chloroform-*d*) δ 7.66 (d, *J* = 9.5 Hz, 1H), 7.47 (d, *J* = 8.6 Hz, 1H), 7.43 – 7.41 (m, 2H), 7.27 (dd, *J* = 5.1, 2.0 Hz, 3H), 6.92 (d, *J* = 2.4 Hz, 1H), 6.81 (dd, *J* = 8.6, 2.4 Hz, 1H), 6.53 (d, *J* = 9.5 Hz, 1H), 3.82 (s, 3H), 1.80 (s, 3H), 1.31 (s, 9H). ^{13}**C NMR** (126 MHz, Chloroform-*d*) δ 168.30, 162.12, 161.23, 140.90, 139.81, 137.58, 136.69, 133.72, 129.99, 128.71, 128.53, 128.12, 119.14, 114.61, 110.92, 99.02, 81.96, 55.52, 27.66, 16.55. HRMS(ESI) m/z: calculated for $[C_{24}H_{25}NO_4+H]^+$ 392.1856, found 392.1854. HPLC data (Chiralpak AD column, hexane: isopropanol = 85:15, 1.0 mL/min), tr = 7.0 min (major), tr = 8.5 min (minor), ee = 93%.

***tert*-butyl (*P*, *Z*)-2-methyl-3-(2-oxo-7-(prop-1-en-2-yl)quinolin-1(2*H*)-yl)-3-phenylacrylate [(*P*, *Z*)-3x]**

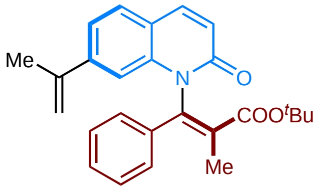

(*P*, *Z*)-**3x**

Following the general procedure of **D**, (*P*, *Z*)-**3x** was obtained as white solid (68% yield). ^{1}**H NMR** (500 MHz, Chloroform-*d*) δ 7.66 (d, *J* = 9.5 Hz, 1H), 7.47 (d, *J* = 7.7 Hz, 1H), 7.40 (t, *J* = 7.9 Hz, 1H), 7.34 (d, *J* = 6.6 Hz, 2H), 7.25 (q, *J* = 8.9, 7.3 Hz, 4H), 7.19 (s, 1H), 7.14 (t, *J* = 7.6 Hz, 1H), 6.62 (d, *J* = 9.5 Hz, 1H), 2.18 (s, 3H), 2.02 (s, 3H), 1.18 (s, 9H). ^{13}**C NMR** (126 MHz, Chloroform-*d*) δ 166.73, 161.62, 143.67, 142.65, 140.19, 139.34, 137.01, 136.10, 132.76, 129.30, 128.86, 128.29, 128.06, 122.06, 119.90, 119.59, 114.64, 113.00, 81.43, 27.39, 21.71, 17.41. HRMS(ESI) m/z: calculated for $[C_{26}H_{27}NO_3+H]^+$ 402.2064, found 402.2063. HPLC data (Chiralpak AD column, hexane: isopropanol = 90:10, 1.0 mL/min), tr = 7.2 min (minor), tr = 21.9 min (major), ee = 97%.

***tert*-butyl (*P*, *E*)-2-methyl-3-(2-oxo-7-(prop-1-en-2-yl)quinolin-1(2*H*)-yl)-3-phenylacrylate [(*P*, *E*)-3x]**

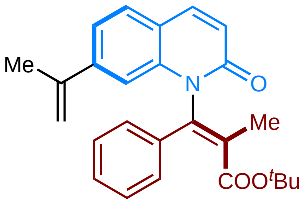

(*P*, *E*)-**3x**

Following the general procedure of **F**, (*P*, *E*)-**3x** was obtained as white solid (41% yield). *E*/*Z*= 1.4/1. ^{1}**H NMR** (500 MHz, Chloroform-d) δ 7.71 (d, *J* = 9.5 Hz, 1H), 7.53 – 7.51 (m, 2H), 7.39 (ddd, *J* = 16.5, 7.1, 2.0 Hz, 4H), 7.27 (d, *J* = 2.6 Hz, 2H), 6.67 (d, *J* = 9.5 Hz, 1H), 5.46 (s, 1H), 5.20 (s, 1H), 2.15 (s, 3H), 1.78 (s, 3H), 1.30 (s, 9H). ^{13}**C NMR** (126 MHz, Chloroform-d) δ 168.29, 160.95, 143.79, 142.36, 139.54, 139.23, 137.36, 133.80, 128.69, 128.54, 128.41, 128.13, 125.37, 122.08, 120.21, 119.69, 114.70, 112.23, 81.92, 27.64, 21.65, 16.58. HRMS(ESI) m/z: calculated for $[C_{26}H_{27}NO_3+H]^+$ 402.2064, found 402.2061. HPLC data (Chiralpak AD column, hexane: isopropanol = 95:5, 1.0 mL/min), tr = 12.5 min (major), tr = 15.8 min (minor), ee = 93%.

tert-butyl (_P, Z_)-3-(7-(4-((((1_S_,2_R_,5_S_)-2-isopropyl-5-methylcyclohexyl)oxy)methyl)phenyl)-2-oxoquinolin-1(2_H_)-yl)-2-methyl-3-phenylacrylate [(_P, Z_)-3y]

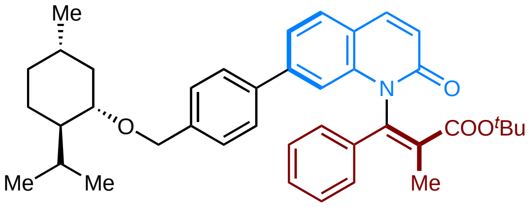

(*P, Z*)-**3y**

Following the general procedure of **D**, (*P, Z*)-**3y** was obtained as white solid with beyond 20/1 dr (72% yield). ^{1}**H NMR** (500 MHz, Chloroform-*d*) δ 7.79 (d, *J* = 9.5 Hz, 1H), 7.64 (d, *J* = 8.1 Hz, 1H), 7.60 – 7.57 (m, 3H), 7.54 – 7.50 (m, 4H), 7.45 (d, *J* = 7.9 Hz, 1H), 7.37 (dt, *J* = 11.8, 6.9 Hz, 3H), 6.74 (d, *J* = 9.5 Hz, 1H), 4.77 (d, *J* = 11.5 Hz, 1H), 4.50 (d, *J* = 11.5 Hz, 1H), 3.27 (td, *J* = 10.5, 4.1 Hz, 1H), 2.41 – 2.37 (m, 1H), 2.35 (s, 3H), 2.28 (d, *J* = 12.3 Hz, 1H), 1.88 (d, *J* = 14.9 Hz, 1H), 1.74 – 1.68 (m, 2H), 1.08 (s, 9H), 0.99 (dd, *J* = 16.0, 6.7 Hz, 10H), 0.82 (d, *J* = 7.0 Hz, 3H). ^{13}**C NMR** (126 MHz, Chloroform-*d*) δ 166.82, 161.59, 143.52, 140.59, 139.58, 139.42, 139.32, 137.01, 136.06, 132.84, 129.32, 128.88, 128.70, 128.42, 128.31, 127.39, 122.18, 121.57, 119.43, 114.45, 79.12 , 70.08, 48.40, 40.40, 34.63, 31.64, 27.41, 25.65, 23.36, 22.42, 21.09, 17.45, 16.21. HRMS(ESI) m/z: calculated for $[C_{40}H_{47}NO_4+H]^+$ 606.3578, found 606.3578.

tert-butyl (_P, E_)-3-(7-(4-((((1_S_,2_R_,5_S_)-2-isopropyl-5-methylcyclohexyl)oxy)methyl)phenyl)-2-oxoquinolin-1(2_H_)-yl)-2-methyl-3-phenylacrylate [(_P, E_)-3y]

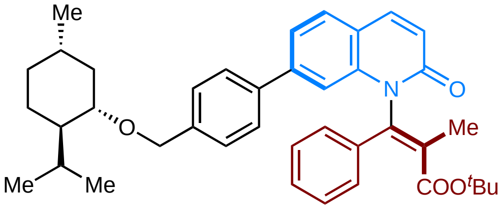

(*P, E*)-**3y**

Following the general procedure of **F**, (*P, E*)-**3y** was obtained as white solid (52% yield) with beyond 20/1 dr. *E*/*Z*= 2/1. ^{1}**H NMR** (500 MHz, Chloroform-d) δ 7.75 (d, *J* = 9.6 Hz, 1H), 7.66 (d, *J* = 1.6 Hz, 1H), 7.63 (d, *J* = 8.0 Hz, 1H), 7.56 (d, *J* = 8.2 Hz, 2H),

7.48 (dd, *J* = 8.1, 1.6 Hz, 1H), 7.44 (dt, *J* = 7.3, 2.6 Hz, 4H), 7.28 (q, *J* = 2.6, 2.0 Hz, 3H), 6.69 (d, *J* = 9.5 Hz, 1H), 4.71 (d, *J* = 11.6 Hz, 1H), 4.45 (d, *J* = 11.6 Hz, 1H), 3.21 (td, *J* = 10.5, 4.1 Hz, 1H), 2.33 (qd, *J* = 7.0, 2.6 Hz, 1H), 2.22 (dq, *J* = 11.7, 3.3, 2.7 Hz, 1H), 1.81 (s, 3H), 1.70 – 1.62 (m, 3H), 1.31 (s, 9H), 1.03 – 0.83 (m, 10H), 0.75 (d, *J* = 6.9 Hz, 3H). **^{13}C NMR** (126 MHz, Chloroform-d) δ 168.19, 160.91, 143.62, 139.65, 139.59, 139.50, 139.08, 137.47, 136.86, 133.90, 129.08, 128.71, 128.59, 128.46, 128.17, 127.22, 122.20, 121.67, 119.57, 113.43, 81.91, 79.14, 70.03, 48.39, 40.39, 34.62, 31.63, 27.66, 25.66, 23.35, 22.41, 21.06, 16.58, 16.20. HRMS(ESI) m/z: calculated for $[C_{40}H_{47}NO_4+H]^+$ 606.3578, found 606.3573.

***tert*-butyl (*P*, *Z*)-2-methyl-3-(2-oxo-6-(4-((((*R*)-2,5,7,8-tetramethyl-2-((4*R*,8*R*)-4,8,12-trimethyltridecyl)chroman-6-yl)oxy)methyl)phenyl)quinolin-1(2*H*)-yl)-3-phenylacrylate [(*P*, *Z*)-3z]**

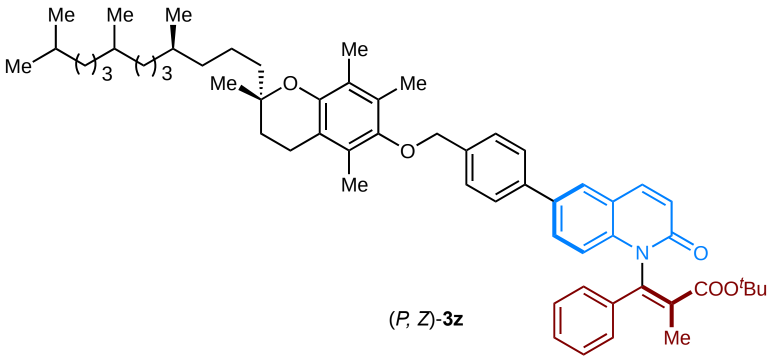

(*P*, *Z*)-**3z**

Following the general procedure of **D**, (*P*, *Z*)-**3z** was obtained as white solid with beyond 20/1 dr (64% yield). **^{1}H NMR** (500 MHz, Chloroform-*d*) δ 7.84 (d, *J* = 10.6 Hz, 2H), 7.79 (d, *J* = 9.5 Hz, 1H), 7.69 (d, *J* = 8.0 Hz, 2H), 7.65 (d, *J* = 8.0 Hz, 2H), 7.55 (d, *J* = 7.4 Hz, 2H), 7.51 (d, *J* = 8.7 Hz, 1H), 7.38 (dd, *J* = 11.3, 7.2 Hz, 3H), 6.80 (d, *J* = 9.5 Hz, 1H), 4.80 (s, 2H), 2.66 (t, *J* = 7.0 Hz, 2H), 2.36 (s, 3H), 2.30 (s, 3H), 2.25 (s, 3H), 2.17 (s, 3H), 1.93 – 1.82 (m, 2H), 1.75 (s, 2H), 1.61 (tt, *J* = 13.7, 6.2 Hz, 3H), 1.45 (d, *J* = 5.7 Hz, 2H), 1.31 (s, 10H), 1.13 (s, 17H), 0.93 (d, *J* = 6.4 Hz, 11H). **^{13}C NMR** (126 MHz, Chloroform-*d*) δ 166.82, 161.59, 143.52, 140.59, 139.63, 139.58, 139.42, 139.32, 137.01, 136.06, 132.84, 129.32, 128.88, 128.70, 128.58, 128.46, 128.42, 128.31, 128.17, 127.39, 127.21, 122.18, 121.56, 119.43, 114.45, 81.49, 79.12, 70.07, 60.40, 53.45, 48.40, 40.40, 34.63, 31.96, 31.64, 31.48, 30.25, 29.73, 29.69, 29.39, 27.66,

27.41, 25.65, 23.36, 22.72, 22.42, 21.09, 17.45, 16.58, 16.21, 14.24, 14.15. HRMS(ESI) m/z: calculated for $[C_{59}H_{77}NO_5+H]^+$ 880.5875, found 880.5871.

***tert*-butyl (*P*, *E*)-2-methyl-3-(2-oxo-6-(4-((((*R*)-2,5,7,8-tetramethyl-2-((4*R*,8*R*)-4,8,12-trimethyltridecyl)chroman-6-yl)oxy)methyl)phenyl)quinolin-1(2*H*)-yl)-3-phenylacrylate [(*P*, *E*)-3z]**

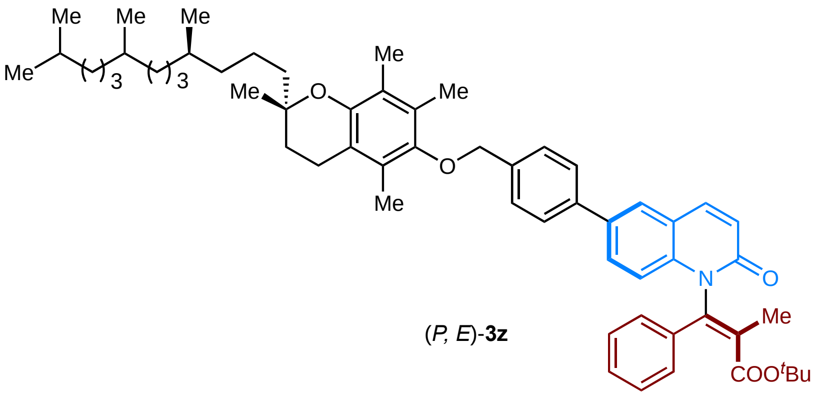

(*P*, *E*)-**3z**

Following the general procedure of **F**, (*P*, *E*)-**3z** was obtained as white solid with beyond 20/1 dr (42% yield). *E*/*Z*= 2/1. **^{1}H NMR** (500 MHz, Chloroform-d) δ 7.77 (d, *J* = 9.4 Hz, 1H), 7.69 (s, 1H), 7.66 – 7.58 (m, 5H), 7.50 (d, *J* = 8.1 Hz, 1H), 7.45 (dd, *J* = 6.7, 2.9 Hz, 2H), 7.30 – 7.28 (m, 3H), 6.71 (d, *J* = 9.5 Hz, 1H), 4.75 (s, 2H), 2.60 (t, *J* = 6.9 Hz, 2H), 2.24 (s, 3H), 2.19 (s, 3H), 2.11 (s, 3H), 1.87 – 1.75 (m, 5H), 1.66 (s, 1H), 1.58 – 1.33 (m, 9H), 1.31 – 1.28 (m, 13H), 1.21 – 0.99 (m, 9H), 0.87 – 0.84 (m, 13H). **^{13}C NMR** (126 MHz, Chloroform-d) δ 168.18, 160.93, 148.14, 148.07, 143.54, 139.67, 139.60, 139.47, 138.36, 137.43, 136.85, 133.95, 129.13, 128.74, 128.62, 128.24, 128.20, 127.90, 127.40, 125.92, 123.07, 122.30, 121.74, 119.66, 117.71, 113.54, 81.95, 74.91, 74.19, 40.10, 39.42, 37.54, 37.51, 37.47, 37.34, 32.85, 32.76, 31.37, 28.03, 27.68, 24.85, 24.49, 23.94, 22.76, 22.67, 21.08, 20.74, 19.80, 19.72, 16.60, 12.92, 12.05, 11.87. HRMS(ESI) m/z: calculated for $[C_{59}H_{77}NO_5+H]^+$ 880.5875, found 880.5879.

Racemization experiments

Compound **(*P*, *Z*)-3a**, **(*P*, *E*)-3a** (0.1 mmol) was dissolved in toluene (1.0 mL) in a sealed tube, respectively. The tube was placed at room temperature. At given interval of time, small samples (5 μL) was removed via syringe and subjected into the HPLC to measure the enantiomeric excess. R = 8.31451 J • K^{-1}• mol^{-1}, h = 6.62608*10^{-34} J • s and k_B = 1.38066*10^{-23} J • K^{-1}• (-ln ee = ln/{(1+***M***/***P***)/(1-***M***/***P***)})

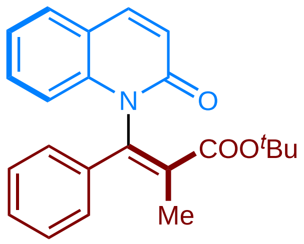

(*P*, *Z*)-**3a**

Time (h)	ee	-ln ee
0	90.34	-4.5035803
2	88.97	-4.4882992
9	86.90	-4.4647081
33	81.98	-4.4065192
68	74.93	-4.3164881
92	69.50	-4.2413268
105	66.69	-4.200105
140	58.93	-4.0763374
164	55.18	-4.0105332

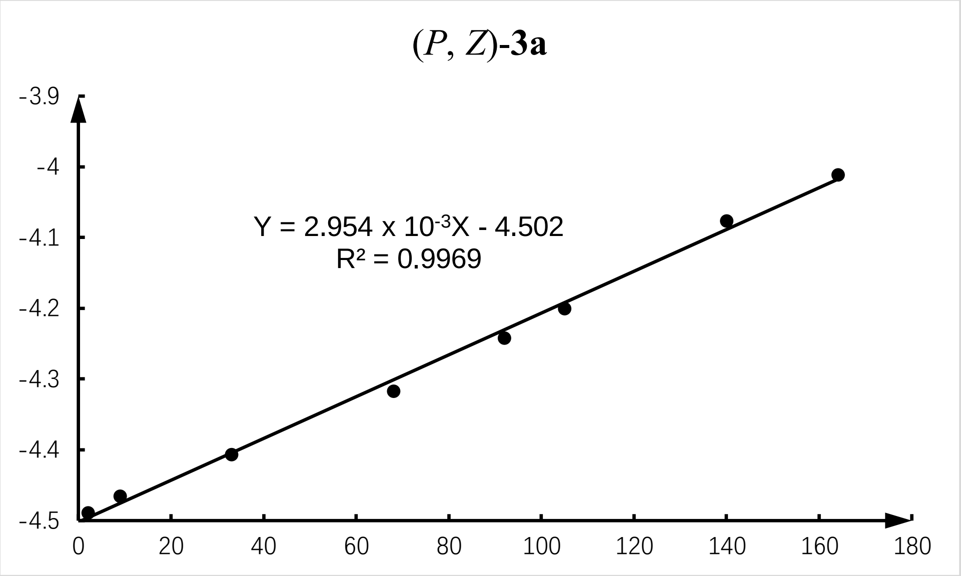

Supplementary Fig. 3. Racemization experiment of (*P*, *Z*)-3a

$$K_{(P,\,Z)\text{-}3a\ \text{racemization}} = 0.002954\ h^{-1} = 8.206*10^{-7}\ s^{-1}$$

$$\text{Half-life time } t_{(P,\,Z)\text{-}3a\ 1/2}^{298K} = 234.65\ h$$

$$\Delta G_{(P,\,Z)\text{-}3a}^{298K} = 107.70\ KJ/mol = 25.73\ kcal/mol$$

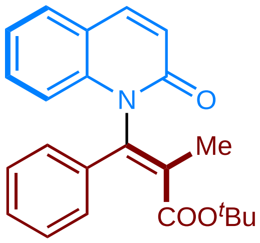

(*P, E*)-**3a**

Time (h)	ee	-ln ee
0	91.2	-4.5130549
6	90.92	-4.50998
12	90.58	-4.5062334
72	88.74	-4.4857107
144	86.8	-4.4636066
312	81.96	-4.4062313
408	80.18	-4.3842741

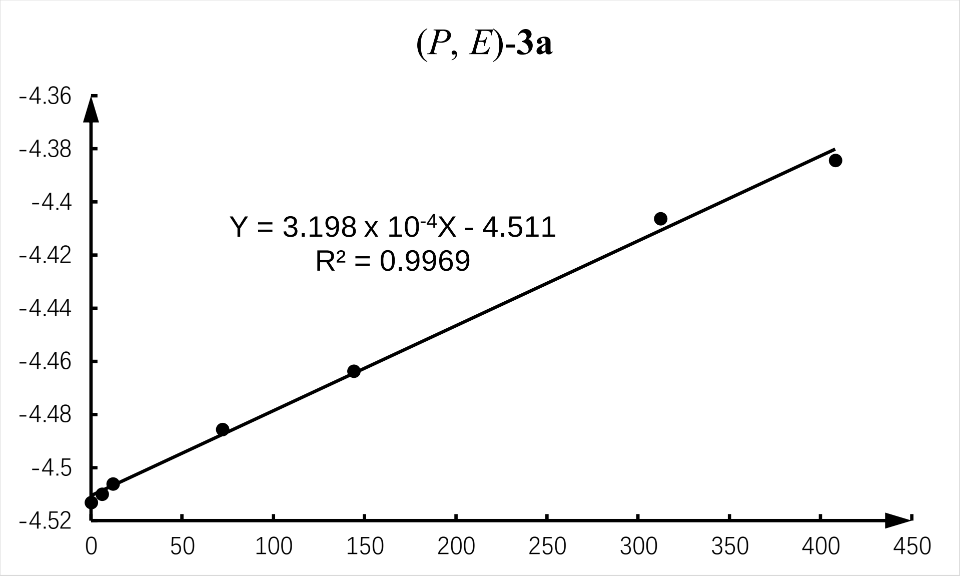

Supplementary Fig. 4. Racemization experiment of (*P*, *E*)-3a

K $_{\mathbf{(P, E)\text{-}3a}\ \text{racemization}}$ = 0.0003198 h^{-1} = 8.883*10^{-8} s^{-1}

Half-life time t $_{\mathbf{(P, E)\text{-}3a}\ 1/2}^{298K}$ =2167.44 h

ΔG $_{\mathbf{(P, E)\text{-}3a}}^{298K}$ = 113.21 KJ/mol = 27.05 kcal/mol

Large-scale reactions for the synthesis of 3a

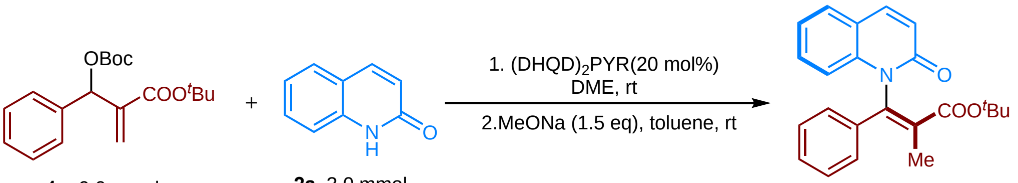

To a flame-dried round-bottom flask equipped with a magnetic stirring bar was added **1a** (9.0 mmol), **2a** (3.0 mmol) and (DHQD)$_2$PYR (0.6 mmol, 528.6 mg). The flask was then charged with DME (20.0 mL) and stirred at room temperature for several days. After the completion of the reaction monitored by TLC, DME was removed by distillation under reduced pressure. Then MeONa (4.5 mmol, 1.5 eq) was added into the flask. The flask was charged with toluene (10 mL) and stirred at room temperature for about 1 h until the full consumption of the intermediate by TLC monitoring. Then the residue was purified directly by column chromatography over silica gel (PE: EA = 20:1 to 5:1) to afford the desired product (*P*, *Z*)-**3a** as white solid (78% yield, 94% ee).

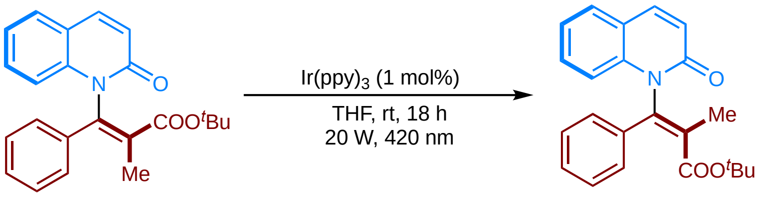

The compound (*P*, *Z*)-**3a** (1.0 mmol, 1.0 eq) and Ir(ppy)$_3$ (1 mol%) were weighed out into a 20 mL scintillation vial. The vial was charged with THF (10 mL) and the reaction was stirred at room temperature under visible light irradiation (420 nm) for 18 h. Then the mixture was concentrated under reduced pressure and purified directly by column chromatography over silica gel (PE: EA = 50:1 to 10:1) to afford the desired product (*P*, *E*)-**3a**. (72% yield, 94% ee)

Stereodivergent synthesis of axially chiral *N*-vinylquinolinones 3a/3s

***tert*-butyl (*P*, *Z*)-2-methyl-3-(2-oxoquinolin-1(2*H*)-yl)-3-phenylacrylate [(*P*, *Z*)-3a]**

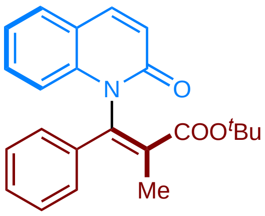

(*P, Z*)-**3a**

Following the general procedure of **D**, (*P*, *Z*)-**3a** was obtained as white solid (87% yield). HPLC data (Chiralpak OD column, hexane: isopropanol = 95:5, 1.0 mL/min), tr = 12.4 min (minor), tr = 14.1 min (major), ee = 94%.

***tert*-butyl (*M, Z*)-2-methyl-3-(2-oxoquinolin-1(2*H*)-yl)-3-phenylacrylate [(*M, Z*)-3a]**

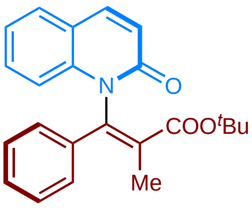

(*M, Z*)-**3a**

Following the general procedure of **D**, using (DHQ)$_2$PYR instead of (DHQD)$_2$PYR, (*M*, *Z*)-**3a** was obtained as white solid (78% yield). HPLC data (Chiralpak OD column, hexane: isopropanol = 95:5, 1.0 mL/min), tr = 11.9 min (major), tr = 14.2 min (minor), ee = 86%.

***tert*-butyl (*P, E*)-2-methyl-3-(2-oxoquinolin-1(2*H*)-yl)-3-phenylacrylate [(*P, E*)-3a]**

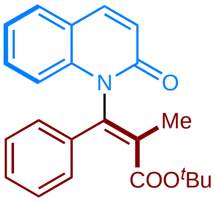

(*P, E*)-**3a**

Following the general procedure of **G**, (*P*, *E*)-**3a** was obtained as white solid (73% yield). HPLC data (Chiralpak OD column, hexane: isopropanol = 95:5, 1.0 mL/min), tr = 15.5 min (minor), tr = 23.3 min (minor), ee = 94%.

***tert*-butyl (*M, E*)-2-methyl-3-(2-oxoquinolin-1(2*H*)-yl)-3-phenylacrylate [(*M, E*)-3a]**

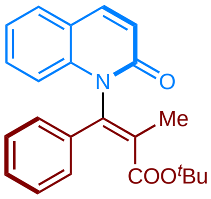

(*M, E*)-**3a**

Following the general procedure of **G**, using (DHQ)2PYR instead of (DHQD)2PYR, (*M*, *E*)-**3a** was obtained as white solid (65% yield). HPLC data (Chiralpak OD column, hexane: isopropanol = 95:5, 1.0 mL/min), tr = 15.4 min (major), tr = 25.9 min (minor), ee = 84%.

***tert*-butyl (*P*, *Z*)-3-(5-bromo-2-oxoquinolin-1(2*H*)-yl)-2-methyl-3-phenylacrylate [(*P*, *Z*)-3s]**

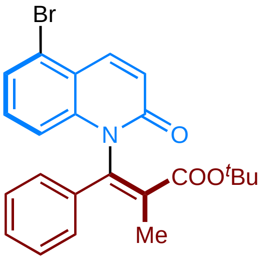

(*P*, *Z*)-**3s**

Following the general procedure of **D**, (*P*, *Z*)-**3s** was obtained as white solid (84% yield). HPLC data (Chiralpak AD column, hexane: isopropanol = 85:15, 1.0 mL/min), tr =7.2 min (minor), tr = 14.4 min (major), ee = 96%.

***tert*-butyl (*M*, *Z*)-3-(5-bromo-2-oxoquinolin-1(2*H*)-yl)-2-methyl-3-phenylacrylate [(*M*, *Z*)-3s]**

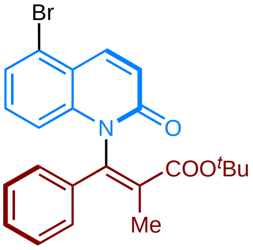

(*M*, *Z*)-**3s**

Following the general procedure of **D**, using (DHQ)2PYR instead of (DHQD)2PYR, (*M*, *Z*)-**3s** was obtained as white solid (78% yield). HPLC data (Chiralpak AD column, hexane: isopropanol = 85:15, 1.0 mL/min), tr =7.5 min (major), tr = 15.3 min (minor), ee = 88%.

***tert*-butyl (*P*, *E*)-3-(5-bromo-2-oxoquinolin-1(2*H*)-yl)-2-methyl-3-phenylacrylate [(*P*, *E*)-3s]**

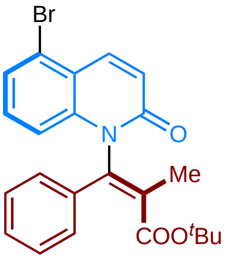

(*P, E*)-**3s**

Following the general procedure of **G**, (*P*, *E*)-**3s** was obtained as white solid (65% yield). HPLC data (Chiralpak AD column, hexane: isopropanol = 95:5, 1.0 mL/min), tr =17.6 min (major), tr = 19.6 min (minor), ee = 97%.

***tert*-butyl (*M, E*)-3-(5-bromo-2-oxoquinolin-1(2*H*)-yl)-2-methyl-3-phenylacrylate [(*M, E*)-3s]**

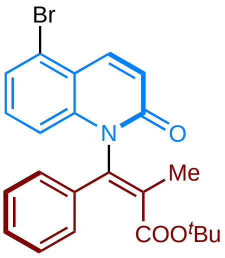

(*M, E*)-**3s**

Following the general procedure of **G**, using $(DHQ)_2PYR$ instead of $(DHQD)_2PYR$, (*M*, *E*)-**3s** was obtained as white solid (61% yield). HPLC data (Chiralpak AD column, hexane: isopropanol = 95:5, 1.0 mL/min), tr =18.4 min (minor), tr = 20.0 min (major), ee = 88%.

Stereodivergent transformations of axially chiral *N*-vinylquinolinones 3

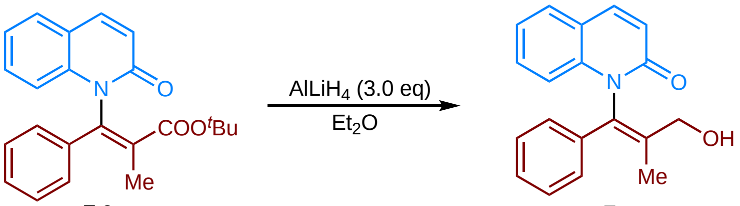

Z-**3a** *Z*-**4**

The reaction was proceeded under argon atmosphere. The compound (*P, Z*)-**3a**/(*M, Z*)-**3a** (0.2 mmol) and $AlLiH_4$ (0.6 mmol) were weighed out into a 10 mL tube. Then the tube was cooled down to -15 °C. Then Et_2O (1.0 mL) was added into the tube. The

reaction was stirred at -15 °C overnight. Then a drop of water was added and the mixture was purified directly by column chromatography over silica gel (PE: EA = 5:1 to 1:1) to afford the desired product (*P*, *Z*)-**4**/(*M, Z*)-**4**.

(*P*, *Z*)-1-(3-hydroxy-2-methyl-1-phenylprop-1-en-1-yl)quinolin-2(1*H*)-one [(*P*, *Z*)-4]

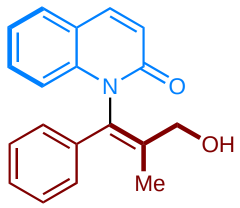

(*P, Z*)-**4**

Colorless oil. (56% yield) ^{1}**H NMR** (500 MHz, Chloroform-d) δ 7.78 (d, *J* = 9.3 Hz, 1H), 7.55 (d, *J* = 8.0 Hz, 1H), 7.39 (dd, *J* = 16.2, 7.9 Hz, 3H), 7.29 (dd, *J* = 8.1, 5.5 Hz, 3H), 7.24 (t, *J* = 7.7 Hz, 1H), 7.18 (t, *J* = 7.5 Hz, 1H), 6.82 (d, *J* = 9.5 Hz, 1H), 4.01 (d, *J* = 11.5 Hz, 1H), 3.79 (d, *J* = 11.6 Hz, 1H), 3.07 (s, 1H), 2.25 (s, 3H). ^{13}**C NMR** (126 MHz, Chloroform-d) δ 163.27, 140.50, 139.49, 138.72, 136.05, 130.74, 130.60, 129.13, 128.60, 128.31, 128.20, 122.91, 121.92, 120.86, 116.46, 63.42, 17.81. HRMS(ESI) m/z: calculated for $[C_{19}H_{17}NO_2+H]^+$ 292.1332, found 292.1334. HPLC data (Chiralpak IA column, hexane: isopropanol = 90:10, 1.0 mL/min), tr =15.3 min (minor), tr = 17.8 min (major), ee = 95%.

(*M*, *Z*)-1-(3-hydroxy-2-methyl-1-phenylprop-1-en-1-yl)quinolin-2(1*H*)-one [(*M*, *Z*)-4]

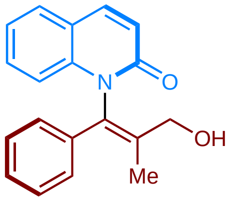

(*M, Z*)-**4**

Colorless oil. (56% yield). HPLC data (Chiralpak IA column, hexane: isopropanol = 90:10, 1.0 mL/min), tr =15.5 min (major), tr = 18.4 min (major), ee = 88%.

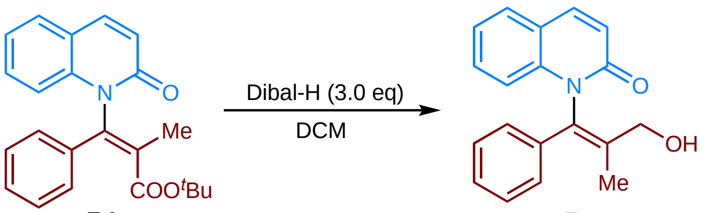

E-**3a** *E*-**4**

The reaction was proceeded under argon atmosphere. The compound (*P, E*)-**3a**/(*M, E*)-**3a** (0.2 mmol) was weighed out into a 10 mL tube. The tube was cooled down to -78 °C before DCM (1.0 mL) was added. Then Dibal-H (2.0 M in toluene, 0.3 mL) was added dropwise and the reaction was stirred at -78 °C for 5 h. Then a drop of water was added to quench the reaction. The reaction mixture was purified directly by column chromatography over silica gel (PE: EA = 5:1 to 1:1) to afford product (*P*, *E*)-**4**/(*M, E*)-**4**.

(*P*, *E*)-1-(3-hydroxy-2-methyl-1-phenylprop-1-en-1-yl)quinolin-2(1*H*)-one [(*P*, *E*)-4]

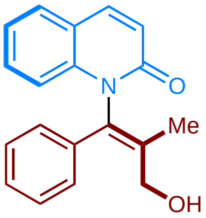

(*P, E*)-**4**

Colorless oil. (65% yield) **^{1}H NMR** (500 MHz, Chloroform-d) δ 7.77 (d, *J* = 9.5 Hz, 1H), 7.57 (dd, *J* = 7.8, 1.5 Hz, 1H), 7.45 (ddd, *J* = 8.6, 7.0, 1.5 Hz, 1H), 7.41 – 7.39 (m, 3H), 7.26 (qd, *J* = 7.7, 6.6, 3.7 Hz, 3H), 7.21 – 7.19 (m, 1H), 6.77 (d, *J* = 9.5 Hz, 1H), 4.53 (d, *J* = 12.1 Hz, 1H), 4.36 (d, *J* = 12.1 Hz, 1H), 3.40 (s, 1H), 1.69 (s, 3H). **^{13}C NMR** (126 MHz, Chloroform-d) δ 161.66, 140.27, 139.01, 138.98, 135.77, 131.82, 130.92, 129.20, 128.59, 128.32, 128.26, 122.74, 122.06, 120.69, 115.77, 63.20, 15.96. HRMS(ESI) m/z: calculated for $[C_{19}H_{17}NO_2+H]^+$ 292.1332, found 292.1336. HPLC data (Chiralpak IA column, hexane: isopropanol = 90:10, 1.0 mL/min), tr =11.9 min (minor), tr = 13.7 min (major), ee = 94%.

(*M*, *E*)-1-(3-hydroxy-2-methyl-1-phenylprop-1-en-1-yl)quinolin-2(1*H*)-one [(*M*, *E*)-4]

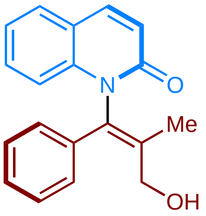

(*M, E*)-**4**

Colorless oil. (59% yield) HPLC data (Chiralpak IA column, hexane: isopropanol = 90:10, 1.0 mL/min), tr =11.8 min (major), tr = 13.8 min (minor), ee = 85%.

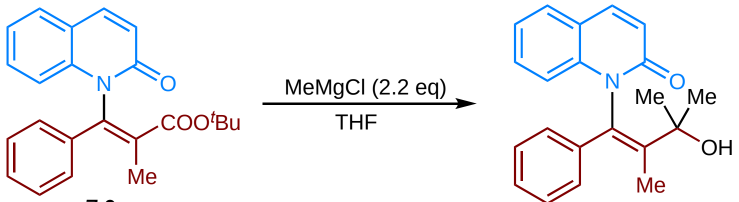

Z-**3a** *Z*-**5**

The reaction was proceeded under argon atmosphere. The Compound (*P, Z*)-**3a**/(*M, Z*)-**3a** (0.2 mmol) was weighed out into a 10ml tube. The tube was cooled down to -78 °C before THF (1.0 mL) was added. Then MeMgCl (3.0 M in THF, 0.15 mL) was added dropwise and the reaction was warmed to room temperature slowly and stirred for 1h. Then drops of water was added and the solvent was removed by distillation under reduced pressure. The crude product was purified by column chromatography over silica gel (PE: EA= 5:1 to 2:1) to afford the desired product (*P*, *Z*)-**5**/(*M, Z*)-**5**.

(*P*, *Z*)-1-(3-hydroxy-2,3-dimethyl-1-phenylbut-1-en-1-yl)quinolin-2(1*H*)-one [(*P*, *Z*)-5]

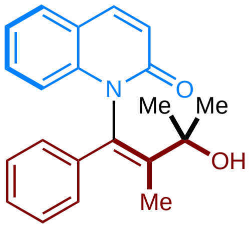

(*P, Z*)-**5**

Colorless oil. (71% yield) ^{1}**H NMR** (500 MHz, Chloroform-d) δ 7.71 (d, *J* = 9.5 Hz, 1H), 7.52 (d, *J* = 8.2 Hz, 2H), 7.50 – 7.48 (m, 1H), 7.46 – 7.44 (m, 2H), 7.27 (t, *J* = 7.5 Hz, 2H), 7.20 (dt, *J* = 15.2, 7.3 Hz, 2H), 6.75 (d, *J* = 9.5 Hz, 1H), 2.41 (s, 1H), 2.06 (s, 3H), 1.41 (s, 3H), 1.23 (s, 3H). ^{13}**C NMR** (126 MHz, Chloroform-d) δ 163.06, 143.95, 140.08, 140.01, 138.91, 130.32, 129.70, 128.58, 128.31, 128.12, 127.85, 122.44,

122.19, 120.81, 116.96, 73.72, 29.31, 28.37, 19.23. HRMS(ESI) m/z: calculated for [$C_{21}H_{21}NO_2$+H]$^+$ 320.1645, found 320.1643. HPLC data (Chiralpak IA column, hexane: isopropanol = 90:10, 1.0 mL/min), tr =10.2 min (minor), tr = 11.9 min (major), ee = 94%.

(*M*, *Z*)-1-(3-hydroxy-2,3-dimethyl-1-phenylbut-1-en-1-yl)quinolin-2(1*H*)-one [(*M*, *Z*)-5]

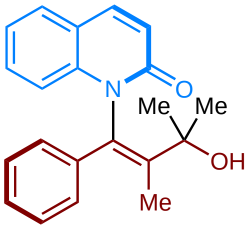

(*M*, *Z*)-**5**

Colorless oil. (66% yield) HPLC data (Chiralpak IA column, hexane: isopropanol = 90:10, 1.0 mL/min), tr = 9.8 min (major), tr = 11.5 min (minor), ee = 86%.

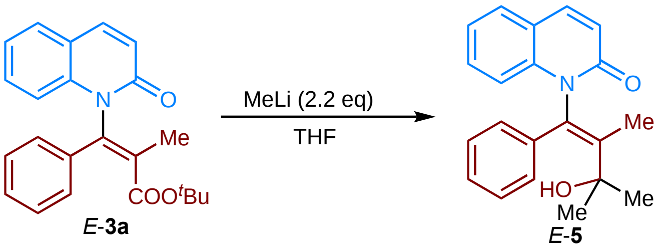

The reaction was proceeded under argon atmosphere. The compound (*P*, *E*)-**3a**/(*M*, *E*)-**3a** (0.2 mmol) was weighed out into a 10 mL tube. The tube was cooled down to -78 °C before THF (1.0 mL) was added. Then MeLi (3.0 M in THF, 0.15 mL) was added dropwise and the reaction was warmed to room temperature slowly and further stirred for 0.5 h. Then drops of water was added and the solvent was removed by distillation under reduced pressure. The crude product was purified by column chromatography over silica gel (PE: EA= 5:1 to 2:1) to afford the desired product (*P*, *E*)-**5**/(*M*, *E*)-**5**.

(*P*, *E*)-1-(3-hydroxy-2,3-dimethyl-1-phenylbut-1-en-1-yl)quinolin-2(1*H*)-one [(*P*, *E*)-5]

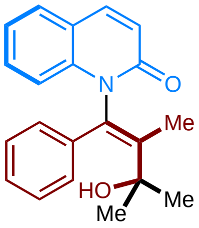

(*P, E*)-**5**

Colorless oil. (49% yield) ^{1}**H NMR** (500 MHz, Chloroform-d) δ 7.68 – 7.65 (m, 2H), 7.61 – 7.59 (m, 2H), 7.57 – 7.53 (m, 2H), 7.26 – 7.20 (m, 4H), 6.67 (d, *J* = 9.5 Hz, 1H), 1.96 (s, 1H), 1.60 (s, 3H), 1.51 (s, 3H), 1.47 (s, 3H). ^{13}**C NMR** (126 MHz, Chloroform-d) δ 161.02, 146.13, 139.56, 138.72, 138.44, 130.68, 129.73, 128.79, 128.30, 128.22, 128.03, 122.62, 122.41, 120.87, 115.37, 74.05, 30.66, 29.79, 16.80. HRMS(ESI) m/z: calculated for $[C_{21}H_{21}NO_2+Na]^+$ 342.1465, found 342.1464. HPLC data (Chiralpak ID column, hexane: isopropanol = 95:5, 1.0 mL/min), tr =35.6 min (minor), tr = 46.4 min (major), ee = 92%.

(*M, E*)-1-(3-hydroxy-2,3-dimethyl-1-phenylbut-1-en-1-yl)quinolin-2(1*H*)-one [(*M, E*)-5]

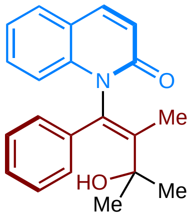

(*M, E*)-**5**

Colorless oil. (42% yield) HPLC data (Chiralpak ID column, hexane: isopropanol = 95:5, 1.0 mL/min), tr =35.6 min (major), tr = 46.8 min (minor), ee = 85%.

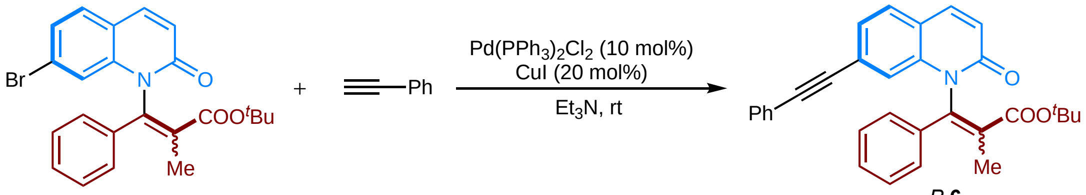

P-**3v** *P*-**6**

The reaction was proceeded under argon atmosphere. The compound (*P, Z*)-**3v**/(*P, E*)-**3v** (0.1 mmol), phenylacetylene (0.2 mmol), $Pd(PPh_3)_2Cl_2$ (10 mol%) and CuI (20

mol%) were weighed out into a 2 dram scintillation vial equipped with a magnetic stirring bar. The vial was then charged with Et_3N (0.75 mL) and stirred at room temperature for overnight. The solvent in the vial was removed by distillation under reduced pressure at room temperature. The crude product was purified by column chromatography over silica gel (PE: EA= 10:1 to 4:1) to afford the desired product (*P*, *Z*)-**6**/(*P, E*)-**6**.

(*P*,*Z*)-*tert*-butyl (*Z*)-2-methyl-3-(2-oxo-7-(phenylethynyl)quinolin-1(2*H*)-yl)-3-phenylacrylate [(*P*, *Z*)-6]

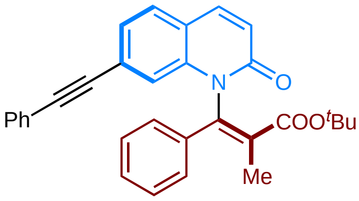

(*P*, *Z*)-**6**

Brown oil. (76% yield). **^{1}H NMR** (500 MHz, Chloroform-*d*) δ 7.68 (d, *J* = 9.5 Hz, 1H), 7.56 (dd, *J* = 6.7, 3.0 Hz, 2H), 7.51 – 7.46 (m, 4H), 7.37 – 7.31 (m, 7H), 6.69 (d, *J* = 9.5 Hz, 1H), 2.31 (s, 3H), 1.08 (s, 9H). **^{13}C NMR** (126 MHz, Chloroform-*d*) δ 166.53, 161.34, 140.23, 139.23, 136.73, 135.82, 133.07, 131.80, 129.32, 128.97, 128.79, 128.46, 128.38, 128.32, 125.65, 125.58, 122.88, 122.75, 120.12, 118.59, 91.71, 89.07, 81.54, 27.50, 17.62. HRMS(ESI) m/z: calculated for $[C_{31}H_{27}NO_3+H]^+$ 462.2064, found 462.2067. HPLC data (Chiralpak IA column, hexane: isopropanol = 80:20, 1.0 mL/min), tr =6.2 min (minor), tr = 17.0 min (major), ee = 87%.

(*P*,*E*)-*tert*-butyl (*E*)-2-methyl-3-(2-oxo-7-(phenylethynyl)quinolin-1(2*H*)-yl)-3-phenylacrylate [(*P*, *E*)-6]

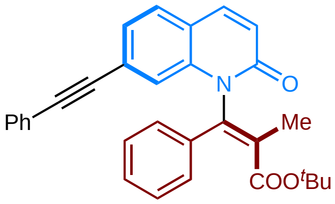

(*P*, *E*)-**6**

Brown oil. (85% yield). **^{1}H NMR** (500 MHz, Chloroform-*d*) δ 7.70 (d, *J* = 9.6 Hz, 1H), 7.57 – 7.53 (m, 4H), 7.44 – 7.42 (m, 2H), 7.37 (dd, *J* = 6.3, 2.4 Hz, 4H), 7.29 – 7.28 (m, 3H), 6.70 (d, *J* = 9.5 Hz, 1H), 1.82 (s, 3H), 1.34 (s, 9H). **^{13}C NMR** (126 MHz,

Chloroform-*d*) δ 168.10, 160.66, 139.46, 139.09, 137.39, 136.56, 134.01, 131.80, 128.84, 128.76, 128.69, 128.56, 128.46, 128.16, 126.02, 126.02, 122.80, 122.62, 120.21, 117.84, 91.98, 88.96, 82.04, 27.66, 16.52. HRMS(ESI) m/z: calculated for $[C_{31}H_{27}NO_3+H]^+$ 462.2064, found 462.2062. HPLC data (Chiralpak AD column, hexane: isopropanol = 85:15, 1.0 mL/min), tr =6.4 min (major), tr = 9.3 min (minor), ee = 92%

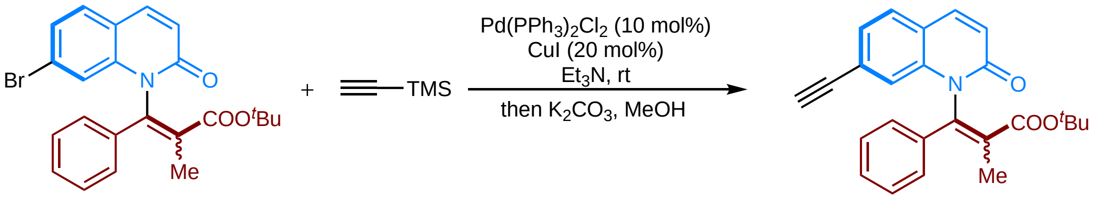

P-**3v** *P*-**7**

The first step of the reaction was proceeded under argon atmosphere. The Compound (*P, Z*)-**3v**/(*P, E*)-**3v** (0.1 mmol), trimethylsilyl acetylene (0.2 mmol), $Pd(PPh_3)_2Cl_2$ (10 mol%) and CuI (20 mol%) were weighed out into a 2 dram scintillation vial equipped with a magnetic stirring bar. The vial was then charged with Et_3N (0.75 mL) and stirred at room temperature for overnight. The solvent in the vial was removed by distillation under reduced pressure at room temperature. Then the crude was dissolved in MeOH (1.0 mL) before K_2CO_3 (0.2 mmol) was added. After 30 minutes, the mixture was concentrated by reduced pressure at room temperature. The crude product was purified by column chromatography over silica gel (PE: EA= 10:1 to 4:1) to afford the desired product (*P, Z*)-**7**/(*P, E*)-**7**.

(*P*, *Z*)-*tert*-butyl (*Z*)-3-(7-ethynyl-2-oxoquinolin-1(2*H*)-yl)-2-methyl-3-phenylacrylate [(*P*, *Z*)-7]

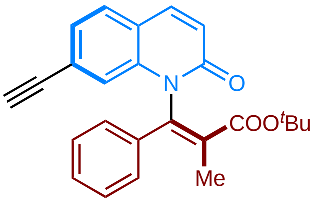

(*P, Z*)-**7**

White solid. (81% yield). **^{1}H NMR** (500 MHz, Chloroform-*d*) δ 7.68 (d, *J* = 9.5 Hz, 1H), 7.49 – 7.44 (m, 4H), 7.33 (qd, *J* = 7.7, 6.7, 3.6 Hz, 3H), 7.29 – 7.26 (m, 1H), 6.71

(d, *J* = 9.5 Hz, 1H), 3.19 (s, 1H), 2.28 (s, 3H), 1.06 (s, 9H). **^{13}C NMR** (126 MHz, Chloroform-*d*) δ 166.46, 161.26, 140.03, 139.15, 136.54, 135.71, 133.17, 129.28, 129.00, 128.38, 128.32, 125.87, 124.23, 123.24, 120.52, 119.45, 83.17, 81.52, 79.50, 27.46, 17.56. HRMS(ESI) m/z: calculated for $[C_{25}H_{23}NO_3+H]^+$ 386.1751, found 386.1734. HPLC data (Chiralpak AD column, hexane: isopropanol = 70:30, 1.0 mL/min), tr =4.9 min (minor), tr = 12.7 min (major), ee = 86%.

(*P*, *E*)- *tert*-butyl (*E*)-3-(7-ethynyl-2-oxoquinolin-1(2*H*)-yl)-2-methyl-3-phenylacrylate [(*P*, *E*)-7]

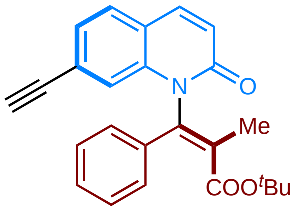

(*P*, *E*)-**7**

White solid. (94% yield).**^{1}H NMR** (500 MHz, Chloroform-*d*) δ 7.70 (d, *J* = 9.6 Hz, 1H), 7.53 – 7.51 (m, 2H), 7.40 (dd, *J* = 6.7, 3.0 Hz, 2H), 7.32 (dd, *J* = 8.0, 1.4 Hz, 1H), 7.27 (dd, *J* = 6.5, 2.5 Hz, 3H), 6.71 (d, *J* = 9.5 Hz, 1H), 3.22 (s, 1H), 1.79 (s, 3H), 1.32 (s, 9H). **^{13}C NMR** (126 MHz, Chloroform-*d*) δ 167.99, 160.58, 139.38, 138.92, 137.29, 136.53, 134.10, 128.79, 128.71, 128.56, 128.18, 126.30, 124.71, 123.21, 120.65, 118.67, 83.05, 82.08, 79.78, 27.65, 16.48. HRMS(ESI) m/z: calculated for $[C_{25}H_{23}NO_3+H]^+$ 386.1751, found 386.1739. HPLC data (Chiralpak AD column, hexane: isopropanol = 95:5, 1.0 mL/min), tr =21.0 min (major), tr = 25.9 min (minor), ee = 93%.

Photocatalyzed *Z*/*E* isomerization of non atropisomeric substrates

In order to show whether the non atropisomeric substrates could be tolerated to the photocatalyzed *Z*/*E* isomerization, we performed the DABCO-catalyzed reaction of MBH carbonates **1a** with 3,4-dihydroquinolin-2(1*H*)-one followed by MeONa-promoted isomerization. Since the non-planarity of *Z*-**S4**, in this case, *Z*-**S4** is non atropisomeric compounds. When *Z*-**S4** was subjected into our photocatalysis system, the reaction proceeded smoothly to afford *E*/*Z* ratio of 7/1. This result indicated that our photocatalysis reaction can be used for the non atropisomeric substrates. More efforts

on the exploration of different kinds of substrates is currently in progress in our laboratory.

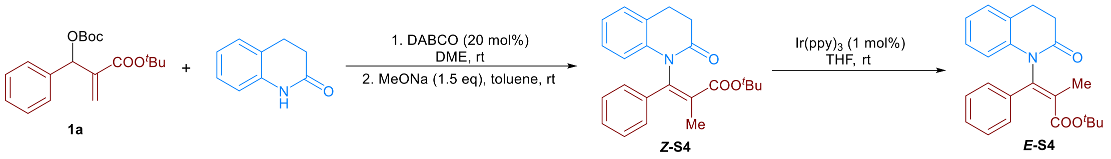

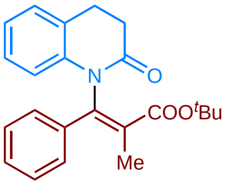

Z-**S4**

Following the general procedure of **D**, *Z*-**S4** was obtained as white solid (86% yield). ^{1}**H NMR** (500 MHz, Chloroform-*d*) δ 7.41 – 7.39 (m, 2H), 7.29 (t, *J* = 7.2 Hz, 2H), 7.27 – 7.24 (m, 1H), 7.14 – 7.09 (m, 2H), 7.00 (d, *J* = 8.1 Hz, 1H), 6.92 (t, *J* = 7.4 Hz, 1H), 2.99 – 2.88 (m, 2H), 2.76 (dt, *J* = 15.6, 5.6 Hz, 1H), 2.65 (ddd, *J* = 15.6, 11.8, 6.3 Hz, 1H), 2.17 (s, 3H), 1.29 (s, 9H). ^{13}**C NMR** (126 MHz, Chloroform-*d*) δ 169.48, 167.09, 139.79, 137.13, 136.37, 132.31, 129.25, 128.59, 128.19, 127.57, 127.21, 126.00, 123.00, 117.44, 81.12, 32.44, 27.87, 25.51, 17.38. HRMS(ESI) m/z: calculated for $[C_{23}H_{25}NO_3+H]^+$ 364.1907, found 364.1905.

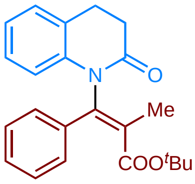

E-**S4**

Following the general procedure of **F**, *E*-**S4** was obtained as white solid (78% yield). ^{1}**H NMR** (500 MHz, Chloroform-*d*) δ 7.36 – 7.34 (m, 2H), 7.26 – 7.24 (m, 3H), 7.20 – 7.16 (m, 2H), 7.02 (d, *J* = 7.9 Hz, 1H), 6.99 (td, *J* = 7.4, 1.2 Hz, 1H), 3.01 – 2.91 (m, 2H), 2.78 – 2.67 (m, 2H), 1.85 (s, 3H), 1.27 (s, 9H). ^{13}**C NMR** (126 MHz, Chloroform-*d*) δ 168.75, 168.40, 139.00, 138.16, 137.42, 133.09, 128.40, 128.38, 128.00, 127.96, 127.71, 125.93, 123.39, 116.43, 81.61, 32.21, 27.57, 25.63, 16.59. HRMS(ESI) m/z: calculated for $[C_{23}H_{25}NO_3+H]^+$ 364.1907, found 364.1907.

Density functional theory studies

The Gaussian 09 program was used for all the density functional theory (DFT) calculations.[1] Geometry optimizations were optimized in toluene (for the isomerization) and tetrahydrofuran (THF, for the photochemical *Z/E* isomerization) with the SMD solvation model, and these calculations were carried out at the M06-2X[2] level of theory with the def2-SVP[3] basis set used for all atoms. Vibrational frequency calculations were performed at the same level to obtain thermal energy correction, and to confirm the stationary point is an energy minimum or a transition state. The single-point energies in THF and toluene were calculated using the SMD solvation model[4] at the M06-2X and def2-TZVPP basis set level of theory. The minimum energy crossing point were calculated with sobMECP program[5] with M06-2X/def2-TZVPP level. The vertical excitation energy was performed with TDDFT method with def2-TZVPP basis set. The spin density plot was generated by the Multiwfn program.[6] Distances are shown in angstroms [Å].

The DFT calculations of MeONa-promoted isomerization of **Int-3** to accessing (*P*, *Z*)-**3a** were shown in Supplementary Fig. 5A. Based on our previous reports on stereospecific isomerization of allylic alkenes (refs 34-38 in manuscript), we proposed that the isomerization was occurred from the conformer **3a1** and **3a2**. With the NaOMe was added in the reaction system, the coordination between amide carbonyl with sodium cation would occur to generate **3a1-1** and **3a2-1**. Our calculation results indicated that the **3a2-1** (-5.9 kcal/mol) was more stable than **3a1-1** (-4.5 kcal/mol). The comparison between **3a1-TS1** and **3a2-TS1** as well as **3aTS-NaOMe** implies that the deprotonation prefers to take place via **3a2-TS1** rather than **3a1-TS1**. Afterward, the reprotonation occurs to afford (*P*, *Z*)-**3a**·NaOMe via **3a2-TS2** (−11.6 kcal/mol).

In addition, we also calculated the energy profiles taken place from the other two conformers **3a3** and **3a4**, respectively (Supplementary Fig. 5B). It can be seen that the energies of **3a3-TS1** and **3a4-TS1** are far higher than that of **3a1-TS1** and **3a2-TS1**. These results indicate that the isomerization of **Int-3** taken place from conformer **3a3** and **3a4** are not likely. Thus, the isomerization affords the axially chiral molecules *P*-

3a as the major product rather than *M*-**3a**. This result is consistent with our experimental outcome illustrated in **Fig.** 2 of manuscript.

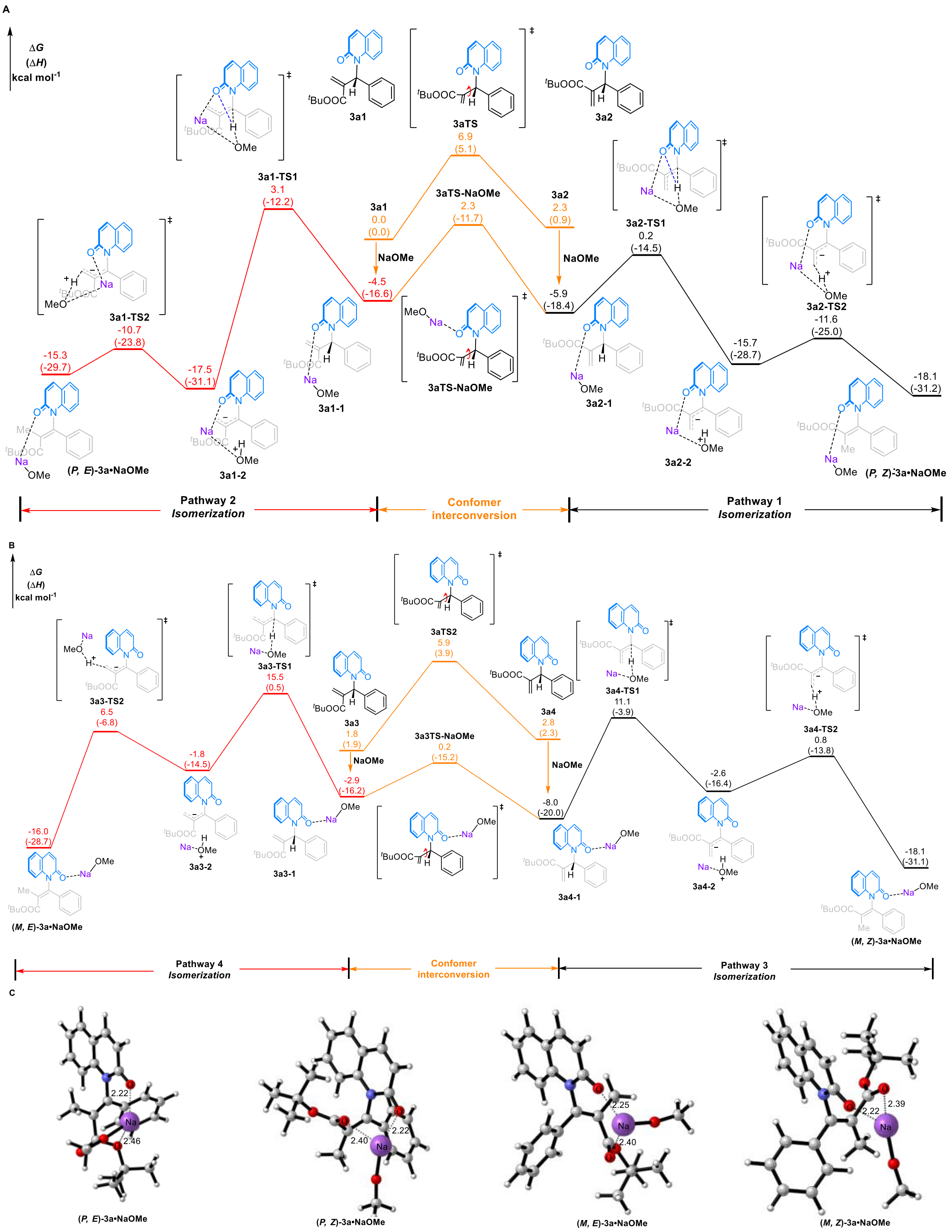

Supplementary Fig. 5. Free energy profiles of MeONa-promoted stereospecific isomerization of Int-3 to accessing (*P*, *Z*)-3a. **A** Isomerization from conformer **3a1** or **3a2**. **B** Isomerization from conformer **3a3** or **3a4**. **C** The key optimized structures.

We then performed the DFT studies of photocatalyzed *Z*/*E* isomerization of (*P, Z*)-**3a**. The results of vertical excitation energy show that the photoexcitation of (*P, Z*)-**3a** is easier than (*P, E*)-**3a**, and T_1 state can be reached after intersystem crossing (ISC). For subsequent relaxation from T_1 to S_0, we calculated the minimum energy intersection (MECP) between singlet and triplet surfaces using the sob-MECP program. For (*P, Z*)-**3a** and (*P, E*)-**3a**, the MECP structure is 5.2 and 4.9 kcal/mol higher than the twisted intermediate (T_1), respectively. From this result in Supplementary Fig. 6, it can be seen that (*P, Z*)-**3a** is more easily excited to the T_1 state and tends to generate a more stable configuration (*P, E*)-**3a** through the crossing point. On the other hand, the spin density plot of **III** shows that the radical electrons are distributed on the aromatic ring.

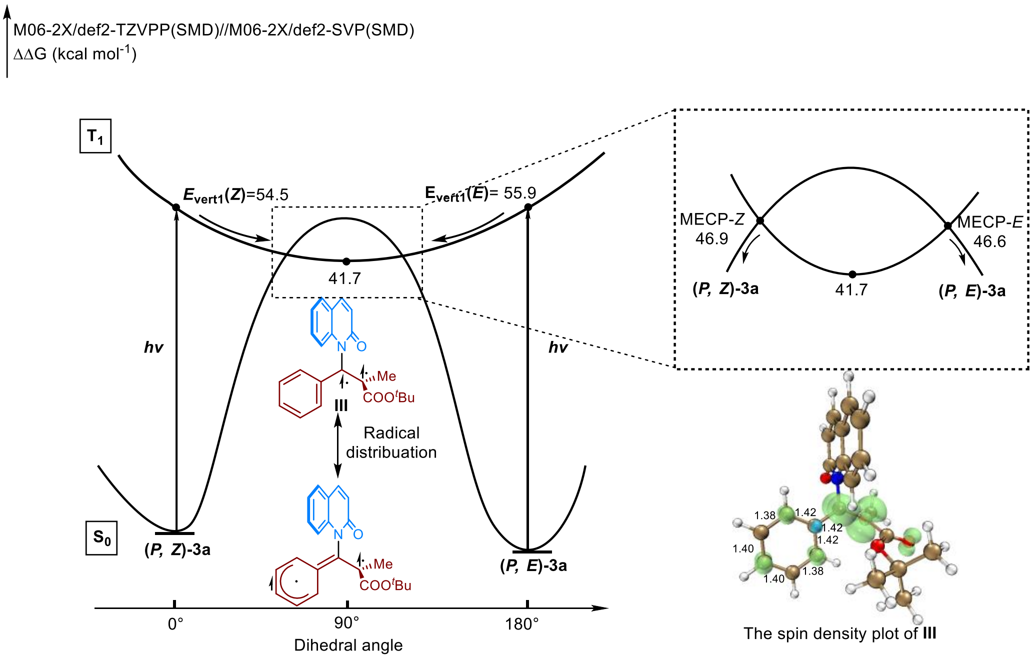

Supplementary Fig. 6. The energy profiles of the photocatalytic reaction.

The explicit solvent effect was calculated by TDDFT method which is shown in Supplementary Table 1. The ratios were consistent with our experimental results illustrated in manuscript.

Supplementary Table 1. Vertical excitation energy of different isomers and solvent molecules (in kcal mol^{-1}).

Entry	$E_{vert}(E)$	$E_{vert}(Z)$
With MeOH	58.4	57.8

With THF	56.9	55.8
With Toluene	55.6	55.2

Supplementary Table 2. Thermal correction of Enthalpy and Gibbs free energy (TCE and TCG, hartree) and total electronic energies (E, hartree) in toluene for all species involved in this study. The intermediate and transition states were calculated at the M06-2X/def2-TZVPP/SMD-(Toluene)//M06-2X/def2-SVP /SMD-(Toluene) level of theory.

Compounds	TCE	TCG	E
**NaOMe**	0.045149	0.012497	-277.4064432
**3a1**	0.438288	0.358787	-1170.731468
**3aTS**	0.437239	0.360602	-1170.722314
**3a2**	0.438364	0.361189	-1170.730117
**3a1-1**	0.485514	0.392652	-1448.166390
**3aTS-NaOMe**	0.484892	0.395100	-1448.157991
**3a2-1**	0.485205	0.392980	-1448.168932
**3a1-TS1**	0.480178	0.392450	-1448.154077
**3a1-2**	0.483529	0.393073	-1448.187525
**3a1-TS2**	0.479115	0.387895	-1448.171556
**(*P*, *E*)-3a·NaOMe**	0.484851	0.395727	-1448.186718
**3a2-TS1**	0.480026	0.391227	-1448.157532
**3a2-2**	0.484928	0.393487	-1448.185078
**3a2-TS2**	0.479195	0.388419	-1448.173537
**(*P*, *Z*)-3a·NaOMe**	0.484847	0.393615	-1448.189089
**3a3**	0.438122	0.358344	-1170.728184
**3aTS2**	0.437635	0.361372	-1170.724575
**3a4**	0.438023	0.359198	-1170.727415
**3a3-1**	0.485654	0.394729	-1448.165981
**3a3TS-NaOMe**	0.484555	0.397043	-1448.163278
**3a4-1**	0.485634	0.392467	-1448.171902
**3a3-TS1**	0.480375	0.392067	-1448.134042
**3a3-2**	0.485527	0.393580	-1448.163125
**3a3-TS2**	0.479335	0.388389	-1448.144719
**(*M*, *E*)-3a·NaOMe**	0.484709	0.392777	-1448.184928
**3a4-TS1**	0.480084	0.391947	-1448.140819
**3a4-2**	0.485241	0.395111	-1448.165887
**3a4-TS2**	0.478226	0.389346	-1448.154768
**(*M*, *Z*)-3a·NaOMe**	0.484811	0.393241	-1448.188844

X-ray crystal structures

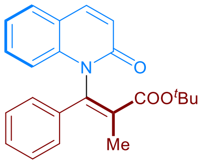

(*P*,*Z*)-**3a**

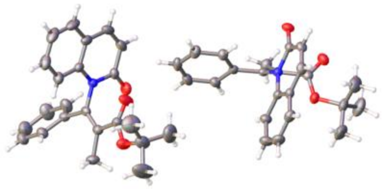

CCDC 2082917

Supplementary Table 3. Crystal data and structure refinement for 2082917.

Identification code	2082917
Empirical formula	$C_{23}H_{23}NO_3$
Formula weight	361.42
Temperature/K	273.15
Crystal system	orthorhombic
Space group	$P2_12_12_1$
a/Å	9.2486(8)
b/Å	13.0118(12)
c/Å	33.021(3)
α/°	90
β/°	90
γ/°	90
Volume/Å^3	3973.8(6)
Z	8
ρ_{calc}g/cm^3	1.208
μ/mm^{-1}	0.638
F(000)	1536.0
Crystal size/mm^3	0.12 × 0.1 × 0.1
Radiation	CuKα (λ = 1.54178)
2Θ range for data collection/°	7.302 to 138.084
Index ranges	$-10 \leq h \leq 11$, $-15 \leq k \leq 12$, $-35 \leq l \leq 39$
Reflections collected	17077
Independent reflections	6919 [R_{int} = 0.0291, R_{sigma} = 0.0295]
Data/restraints/parameters	6919/6/495
Goodness-of-fit on F^2	1.027
Final R indexes [I>=2σ (I)]	R_1 = 0.0331, wR_2 = 0.0825
Final R indexes [all data]	R_1 = 0.0393, wR_2 = 0.0857
Largest diff. peak/hole / e Å^{-3}	0.14/-0.19
Flack parameter	-0.04(7)

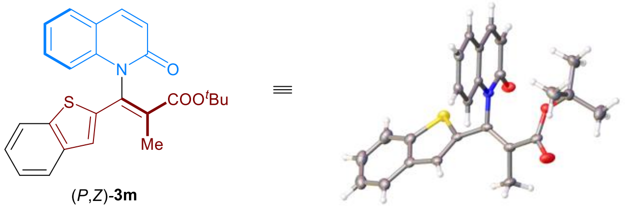

(*P*,*Z*)-**3m**

CCDC 2096051

Supplementary Table 4. Crystal data and structure refinement for 2096051.

Identification code	2096051
Empirical formula	$C_{25}H_{23}NO_3S$
Formula weight	417.50
Temperature/K	100.15
Crystal system	hexagonal
Space group	$P6_5$
a/Å	20.77790(10)
b/Å	20.77790(10)
c/Å	9.16930(10)
α/°	90
β/°	90
γ/°	120
Volume/$Å^3$	3428.23(5)
Z	6
ρ_{calc}g/cm^3	1.213
μ/mm^{-1}	1.456
F(000)	1320.0
Crystal size/mm^3	0.05 × 0.05 × 0.05
Radiation	CuKα (λ = 1.54184)
2Θ range for data collection/°	4.91 to 133.124
Index ranges	$-24 \leq h \leq 24$, $-24 \leq k \leq 24$, $-10 \leq l \leq 8$
Reflections collected	35983
Independent reflections	3724 [R_{int} = 0.0351, R_{sigma} = 0.0166]
Data/restraints/parameters	3724/1/275
Goodness-of-fit on F^2	1.042
Final R indexes [I>=2σ (I)]	R_1 = 0.0225, wR_2 = 0.0602
Final R indexes [all data]	R_1 = 0.0228, wR_2 = 0.0605
Largest diff. peak/hole / e $Å^{-3}$	0.15/-0.15
Flack parameter	0.004(4)

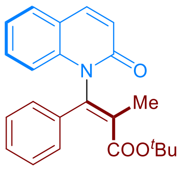

(*P*,*E*)-**3a**

≡

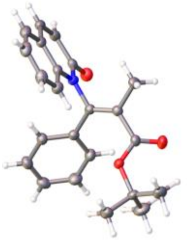

CCDC 2252177

Supplementary Table 5. Crystal data and structure refinement for 2252177.

Identification code	2252177
Empirical formula	$C_{23}H_{23}NO_3$
Formula weight	361.42
Temperature/K	193.15
Crystal system	orthorhombic
Space group	$P2_12_12_1$
a/Å	8.743
b/Å	9.380
c/Å	23.078
α/°	90
β/°	90
γ/°	90
Volume/Å^3	1892.6
Z	4
ρ_{calc}g/cm^3	1.268
μ/mm^{-1}	0.670
F(000)	768.0
Crystal size/mm^3	0.13 × 0.12 × 0.11
Radiation	CuKα (λ = 1.54184)
2Θ range for data collection/°	7.662 to 136.456
Index ranges	$-10 \leq h \leq 10$, $-11 \leq k \leq 11$, $-27 \leq l \leq 27$
Reflections collected	41759
Independent reflections	3461 [R_{int} = 0.0288, R_{sigma} = 0.0180]
Data/restraints/parameters	3461/0/248
Goodness-of-fit on F^2	1.144
Final R indexes [I>=2σ (I)]	R_1 = 0.0307, wR_2 = 0.0771
Final R indexes [all data]	R_1 = 0.0308, wR_2 = 0.0772
Largest diff. peak/hole / e Å^{-3}	0.17/-0.25
Flack parameter	0.02(2)

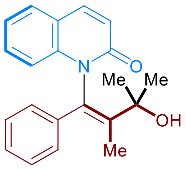

(*P*,*Z*)-**5**

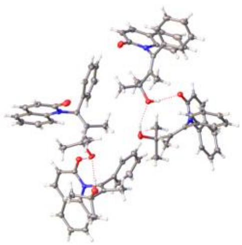

CCDC 2286999

Supplementary Table 6. Crystal data and structure refinement for 2286999.

Identification code	2286999
Empirical formula	$C_{84}H_{84}N_4O_8$
Formula weight	1277.55
Temperature/K	100.01(10)
Crystal system	orthorhombic
Space group	$P2_12_12_1$
a/Å	27.8477(3)
b/Å	18.7127(2)
c/Å	13.05830(10)
α/°	90
β/°	90
γ/°	90
Volume/$Å^3$	6804.76(12)
Z	4
ρ_{calc}g/cm^3	1.247
μ/mm^{-1}	0.631
F(000)	2720.0
Crystal size/mm^3	0.02 × 0.02 × 0.02
Radiation	CuKα (λ = 1.54184)
2Θ range for data collection/°	5.69 to 148.156
Index ranges	$-34 \leq h \leq 34$, $-19 \leq k \leq 23$, $-16 \leq l \leq 16$
Reflections collected	48017
Independent reflections	13620 [R_{int} = 0.0651, R_{sigma} = 0.0462]
Data/restraints/parameters	13620/0/881
Goodness-of-fit on F^2	1.077
Final R indexes [I>=2σ (I)]	R_1 = 0.0551, wR_2 = 0.1392
Final R indexes [all data]	R_1 = 0.0623, wR_2 = 0.1424
Largest diff. peak/hole / e Å^{-3}	0.29/-0.28
Flack parameter	-0.09(12)

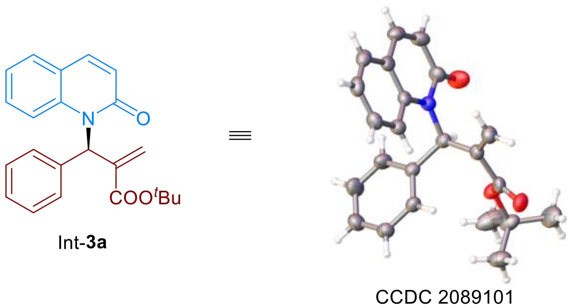

Int-**3a**

CCDC 2089101

Supplementary Table 7. Crystal data and structure refinement for 2089101.

Identification code	2089101
Empirical formula	$C_{23}H_{23}NO_3$
Formula weight	361.42
Temperature/K	273(2)
Crystal system	orthorhombic
Space group	$P2_12_12_1$
a/Å	10.2957(12)
b/Å	12.117(2)
c/Å	16.365(3)
α/°	90
β/°	90
γ/°	90
Volume/Å^3	2041.6(6)
Z	4
ρ_{calc}g/cm^3	1.176
μ/mm^{-1}	0.621
F(000)	768.0
Crystal size/mm^3	0.12 × 0.11 × 0.08
Radiation	CuKα (λ = 1.54178)
2Θ range for data collection/°	9.08 to 136.812
Index ranges	$-12 \le h \le 12$, $-14 \le k \le 14$, $-19 \le l \le 19$
Reflections collected	15331
Independent reflections	3690 [R_{int} = 0.0312, R_{sigma} = 0.0215]
Data/restraints/parameters	3690/0/248
Goodness-of-fit on F^2	1.050
Final R indexes [I>=2σ (I)]	R_1 = 0.0262, wR_2 = 0.0703
Final R indexes [all data]	R_1 = 0.0264, wR_2 = 0.0705
Largest diff. peak/hole / e Å^{-3}	0.12/-0.12
Flack parameter	0.03(4)

NMR spectrum data

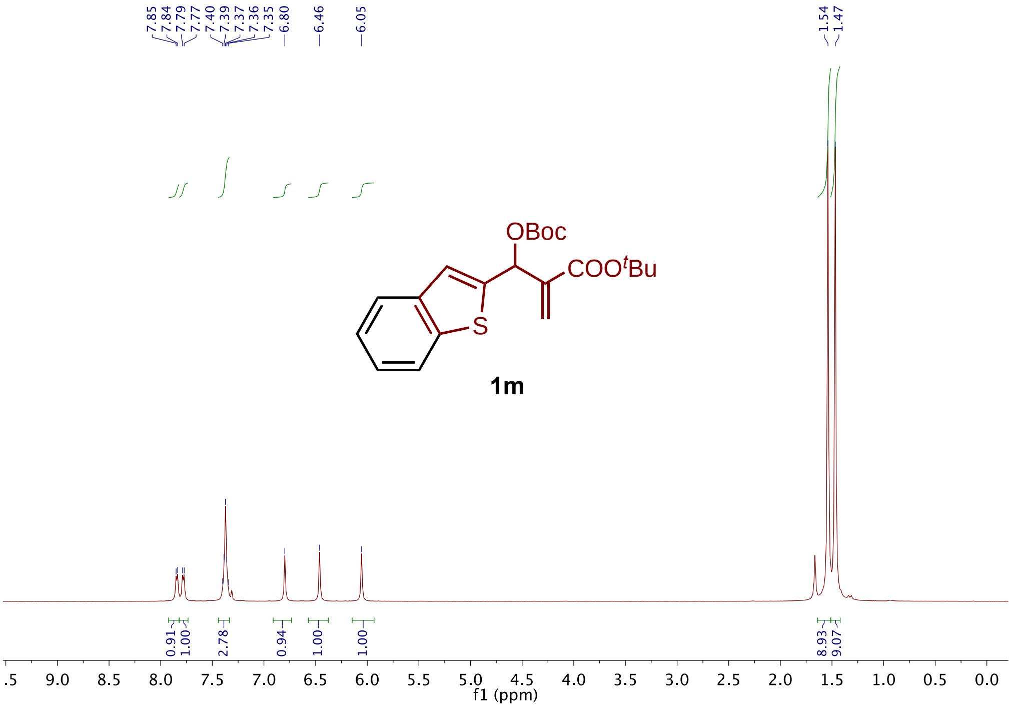

Supplementary Fig. 7. ^{1}H NMR spectrum of 1m.

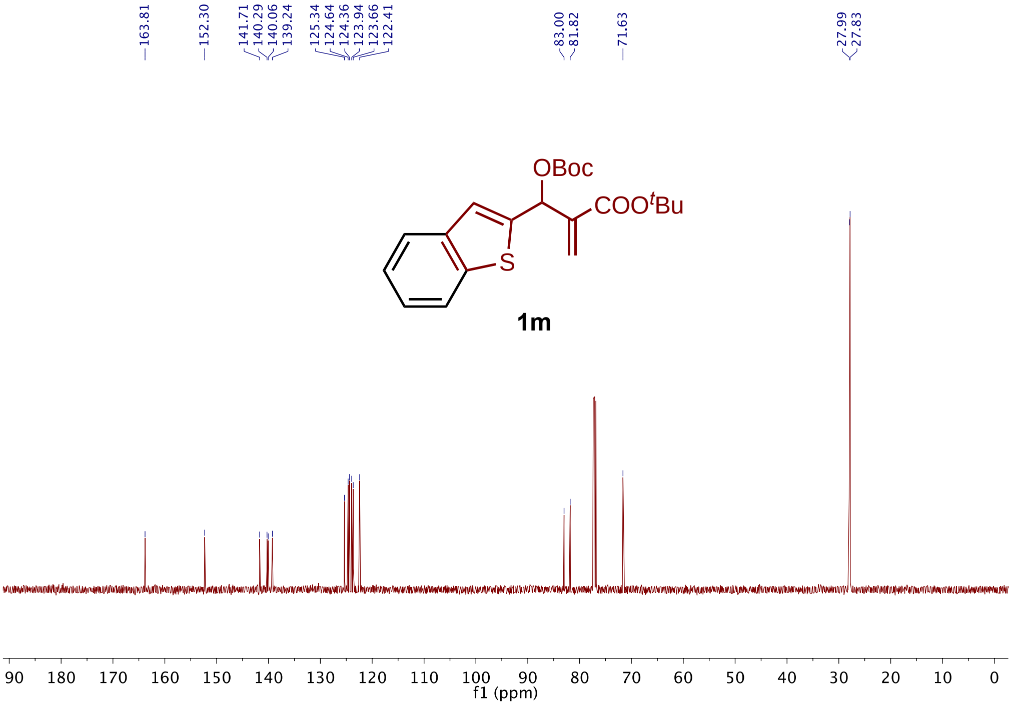

Supplementary Fig. 8. ^{13}C NMR spectrum of 1m.

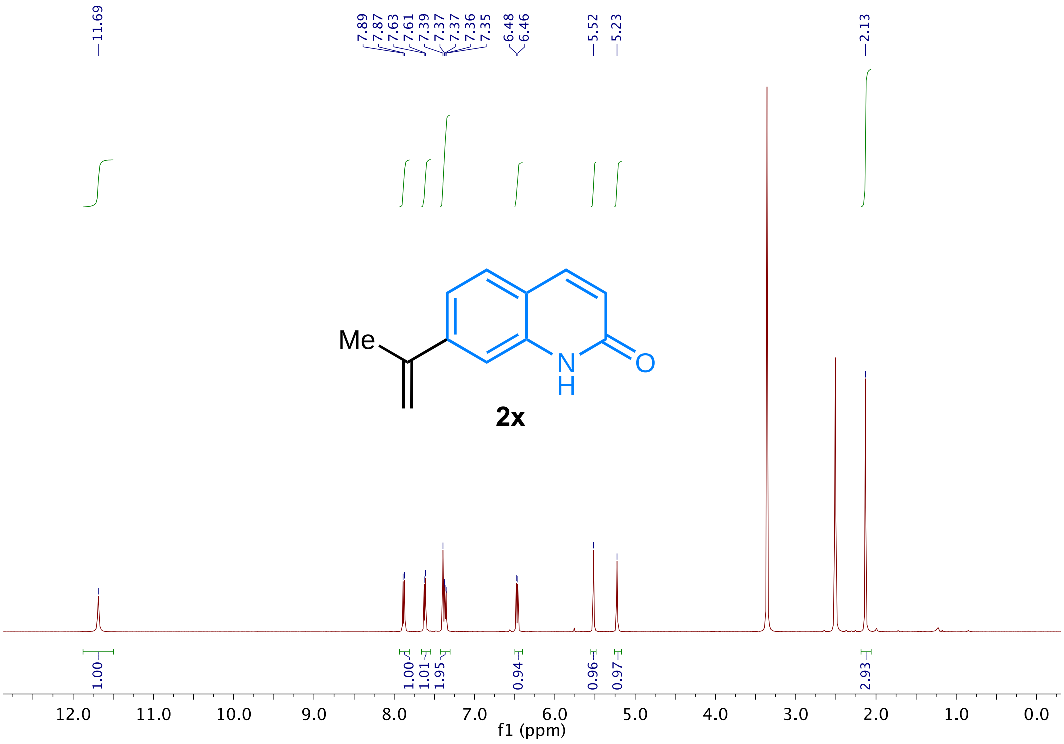

Supplementary Fig. 9. ^{1}**H NMR spectrum of 2x.**

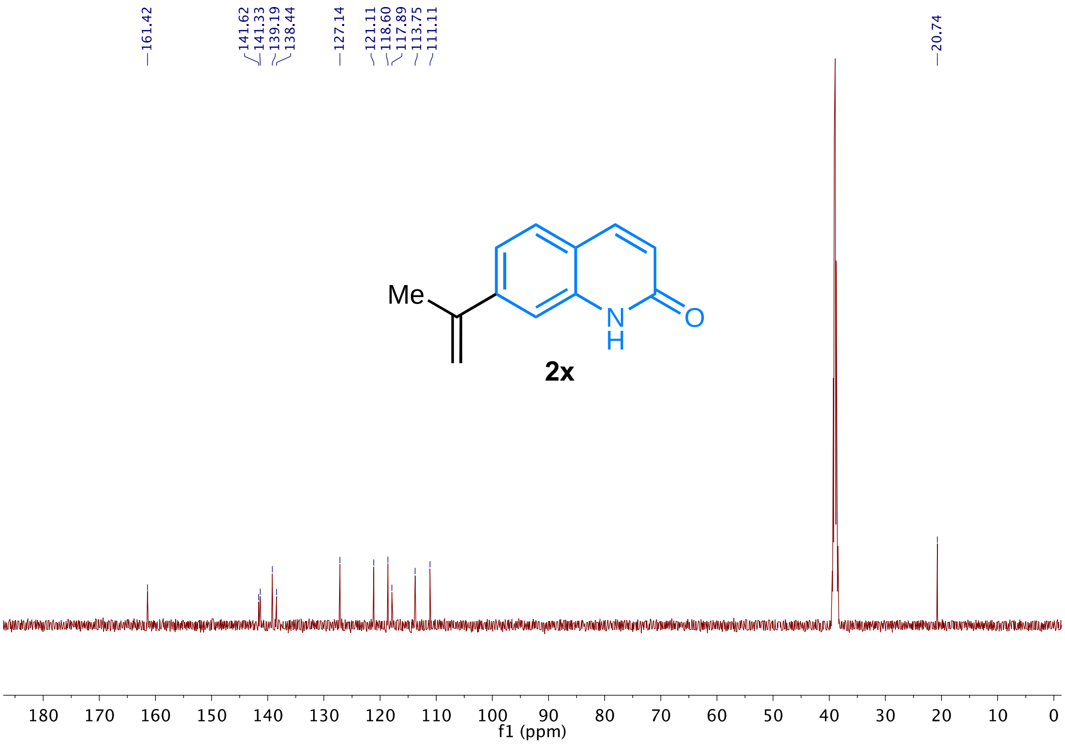

Supplementary Fig. 10. ^{13}**C NMR spectrum of 2x.**

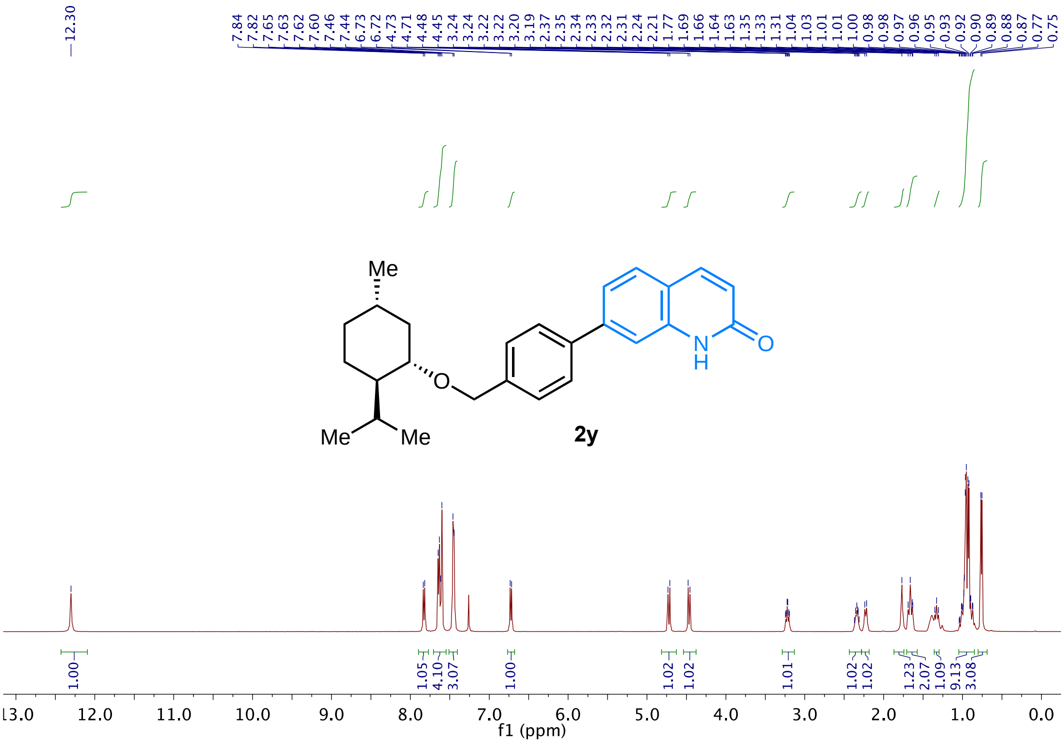

Supplementary Fig. 11. ^{1}**H NMR spectrum of 2y.**

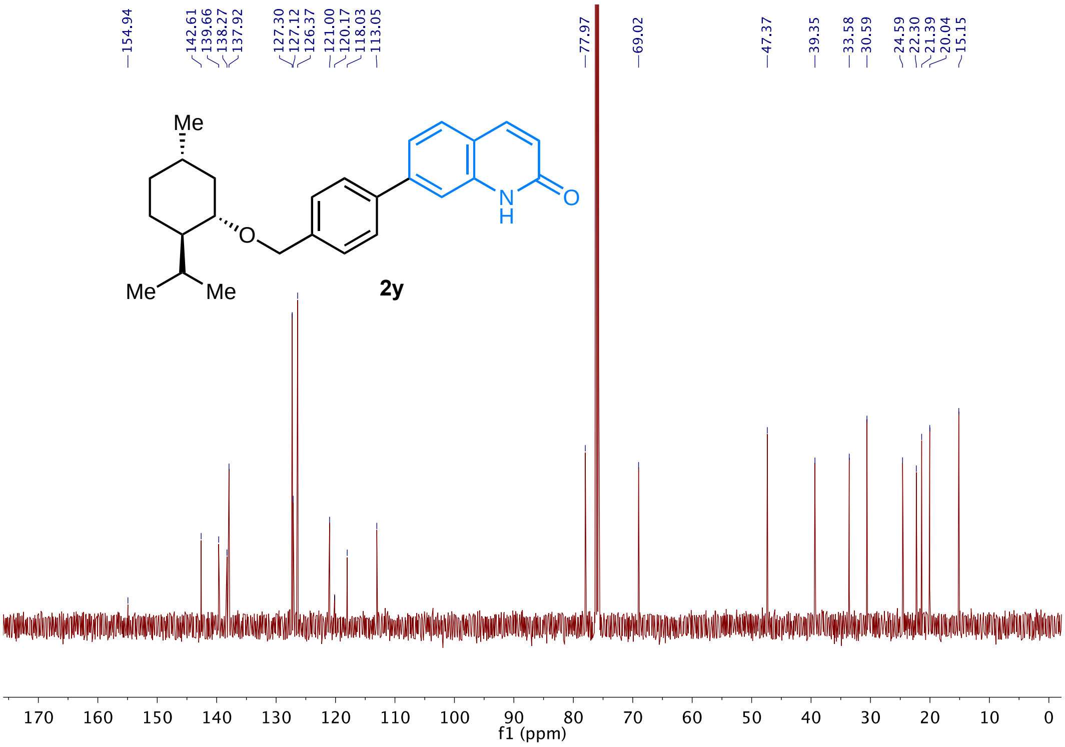

Supplementary Fig. 12. ^{13}**C NMR spectrum of 2y.**

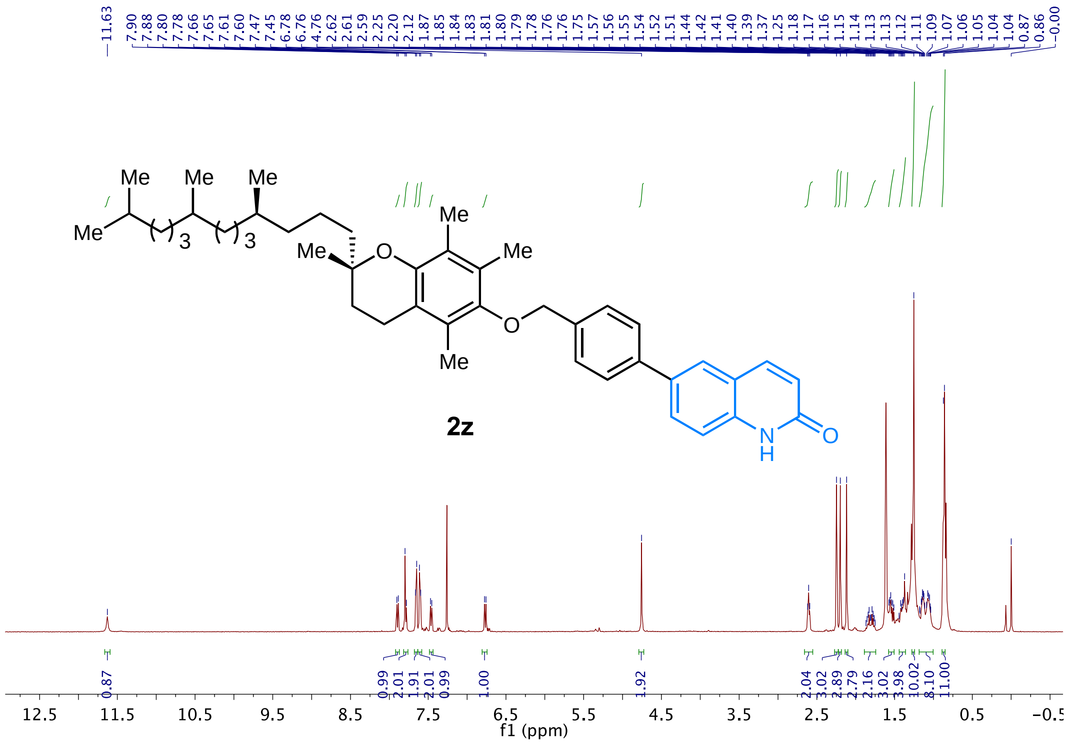

Supplementary Fig. 13. ^{1}H NMR spectrum of 2z.

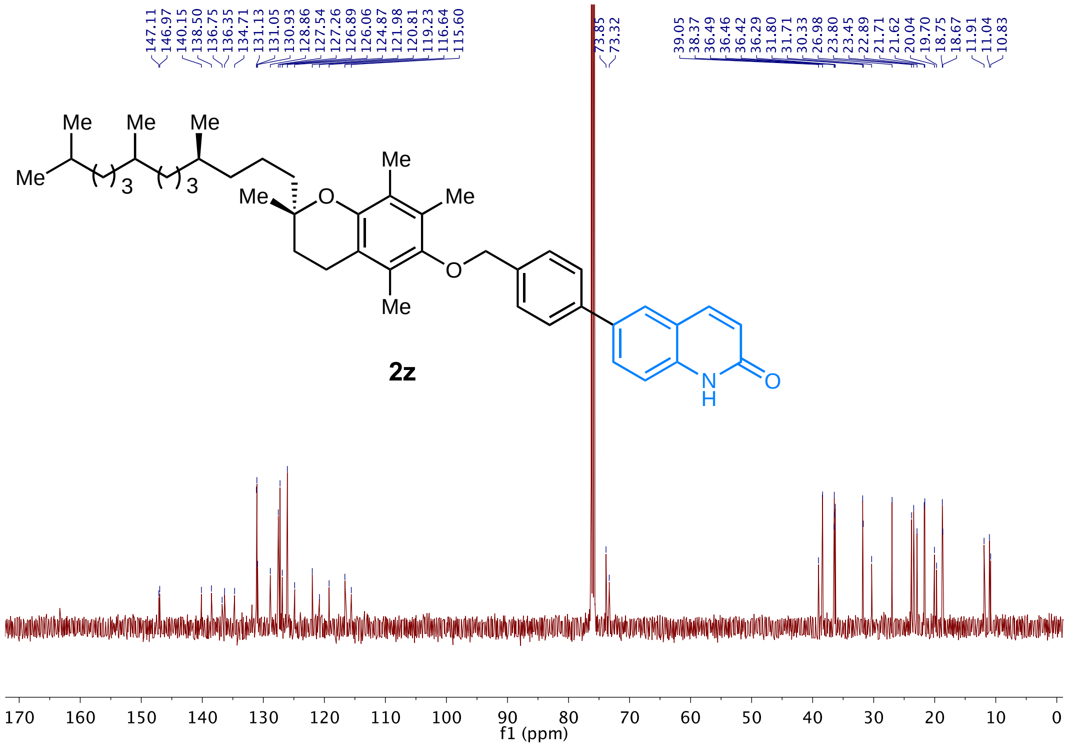

Supplementary Fig. 14. ^{13}C NMR spectrum of 2z.

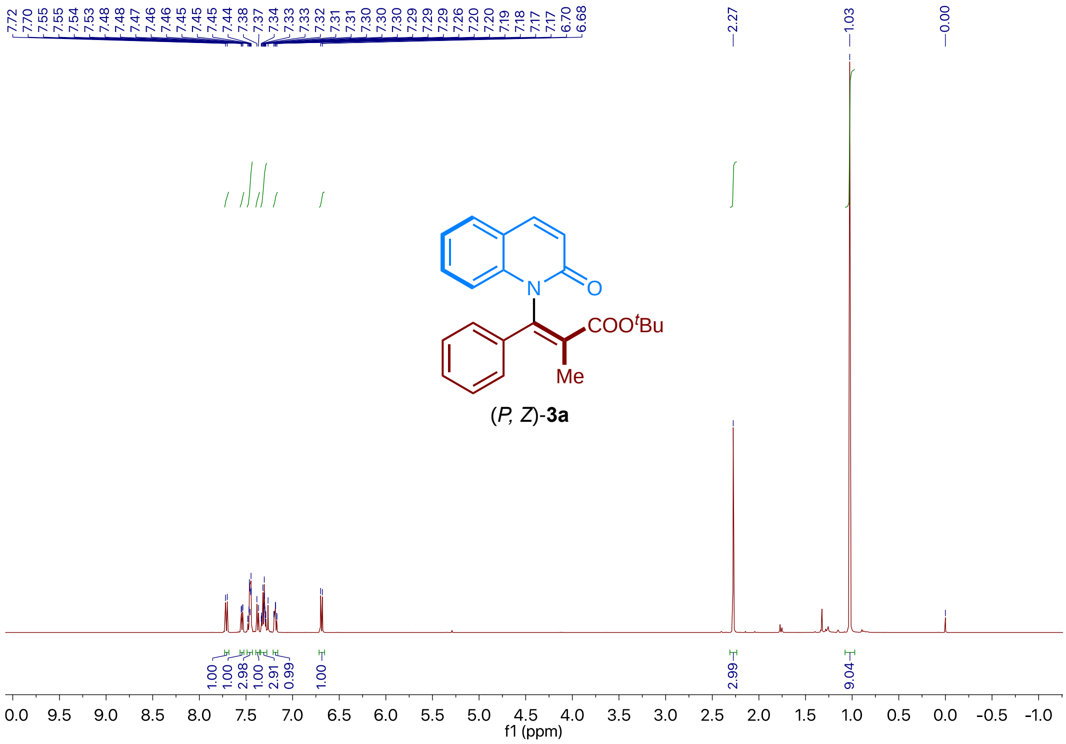

Supplementary Fig. 15. ^{1}H NMR spectrum of (*P*, *Z*)-3a.

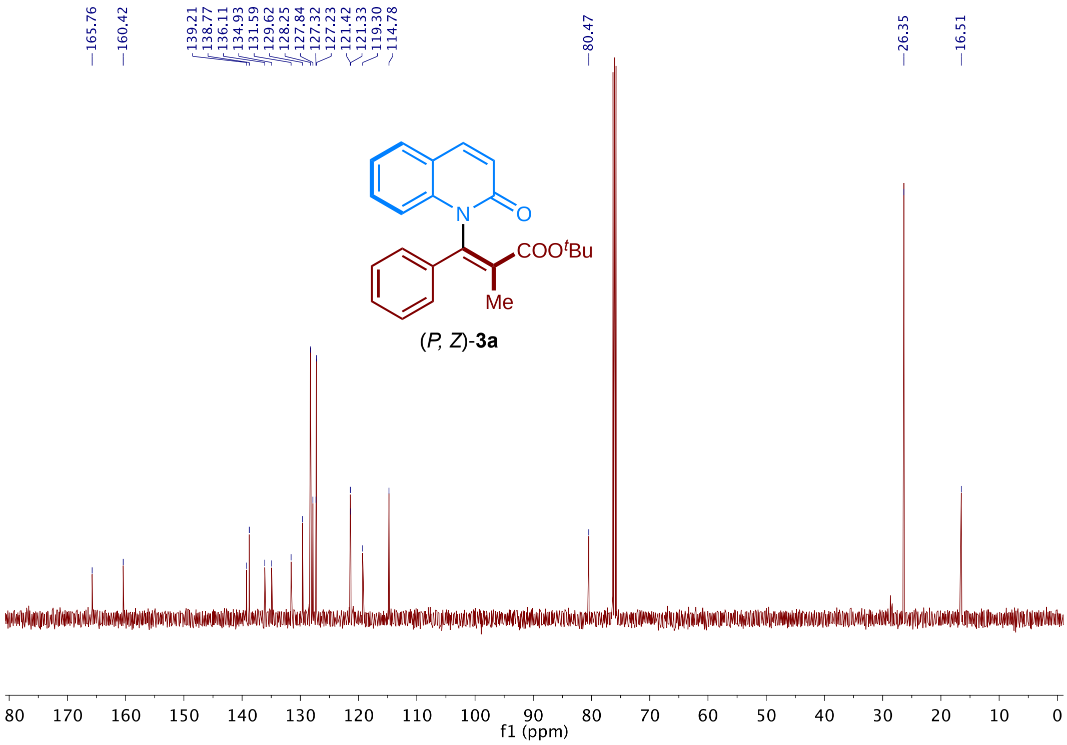

Supplementary Fig. 16. ^{13}C NMR spectrum of (*P*, *Z*)-3a.

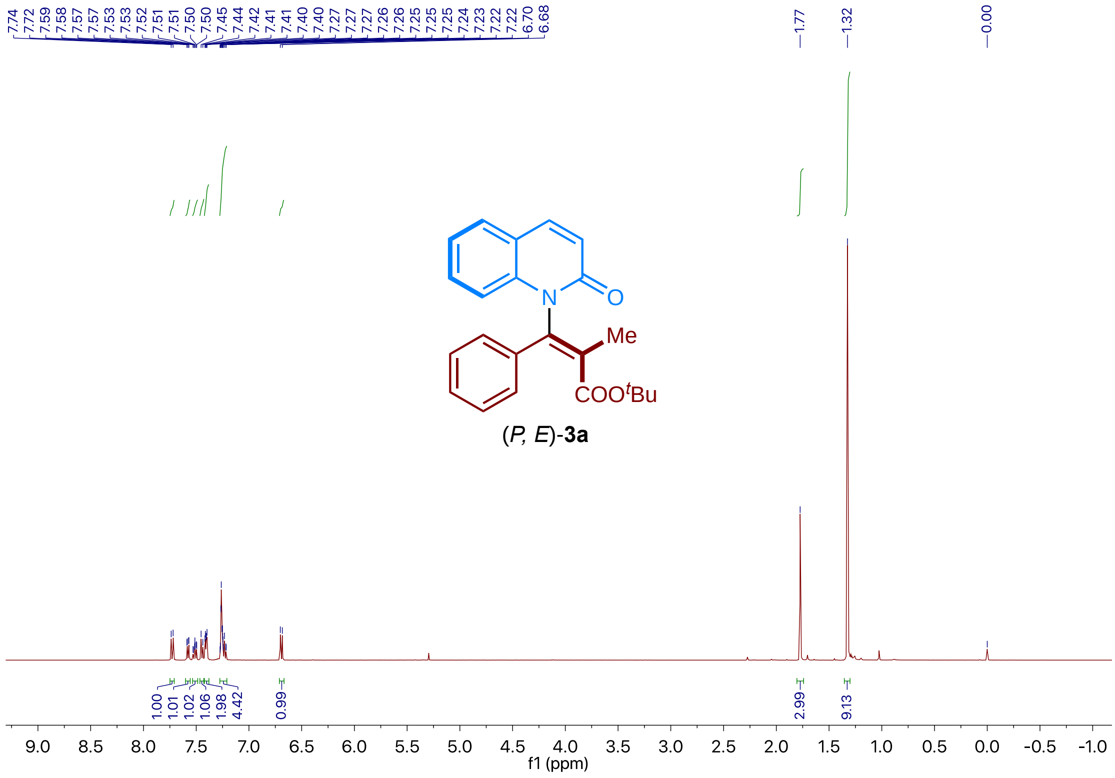

Supplementary Fig. 17. ^{1}**H NMR spectrum of (*P*, *E*)-3a.**

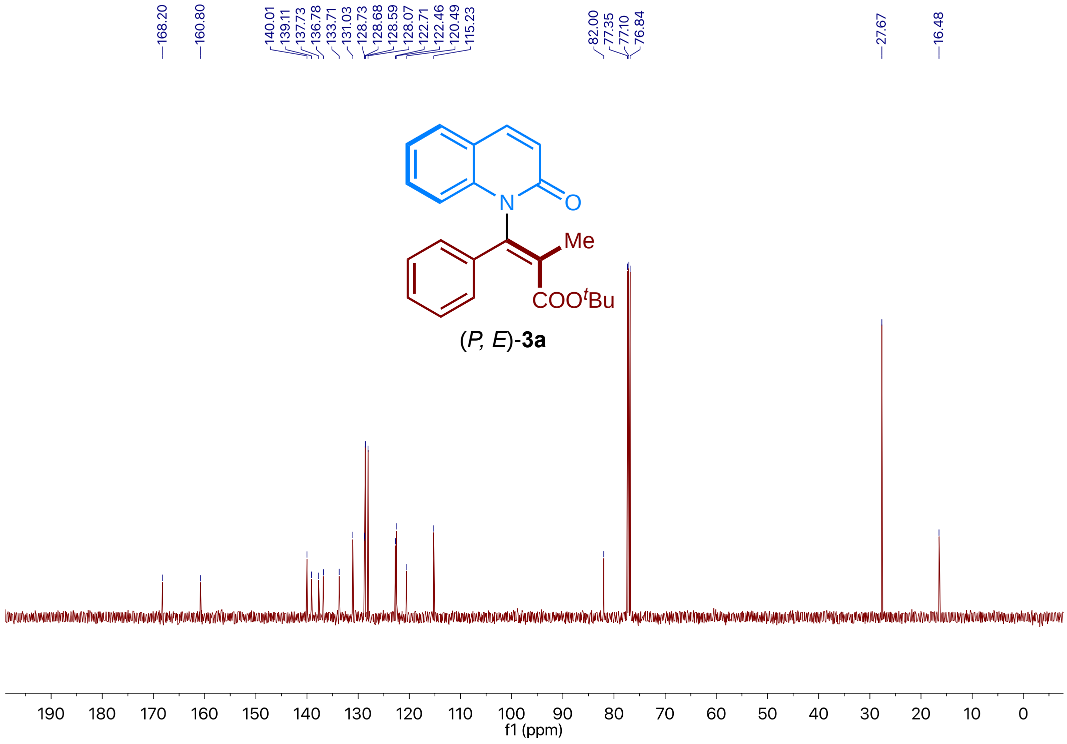

Supplementary Fig. 18. ^{13}**C NMR spectrum of (*P*, *E*)-3a.**

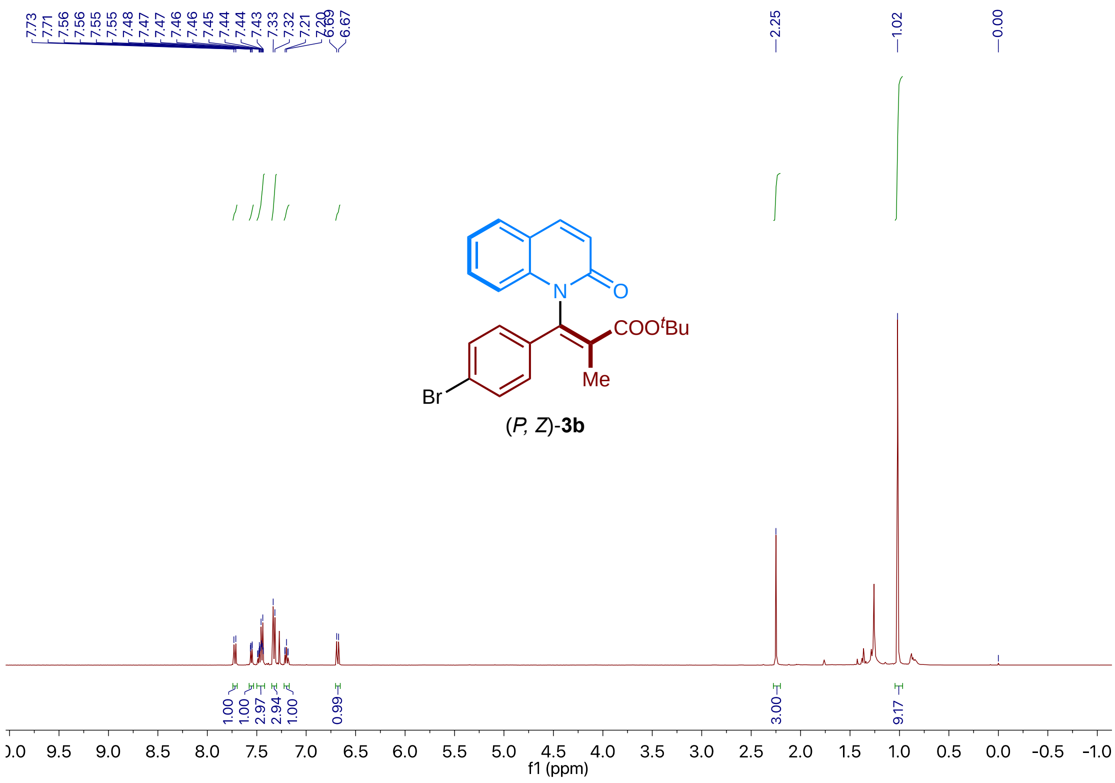

Supplementary Fig. 19. ^{1}H NMR spectrum of (*P*, *Z*)-3b

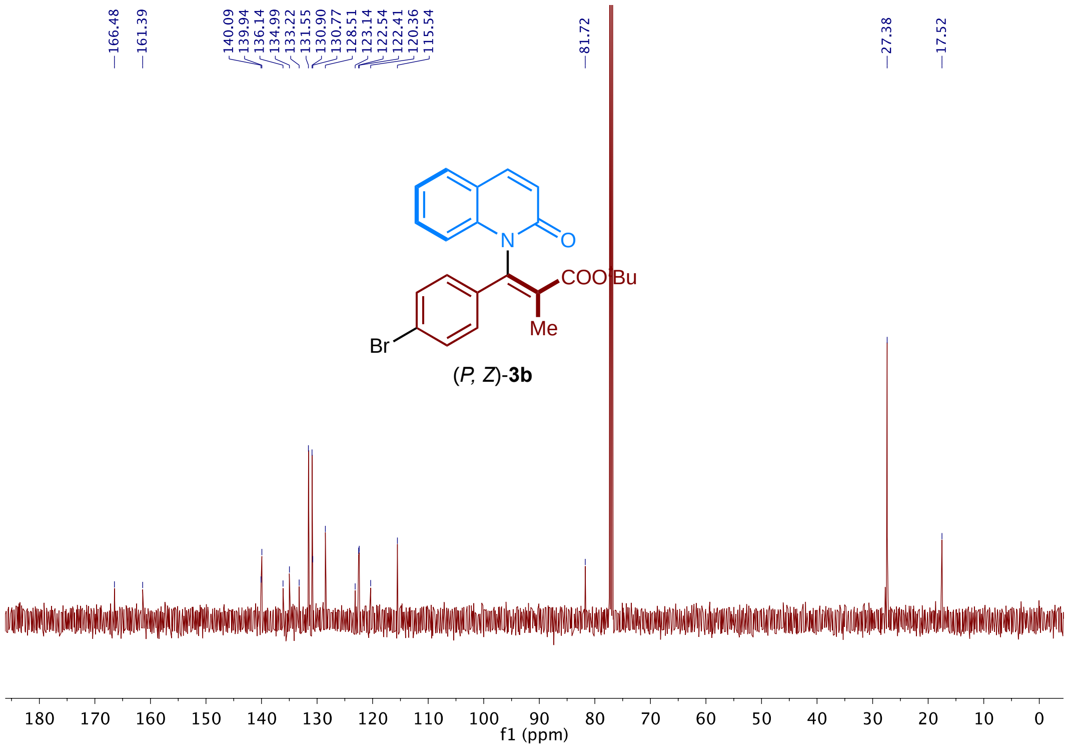

Supplementary Fig. 20. ^{13}C NMR spectrum of (*P*, *Z*)-3b.

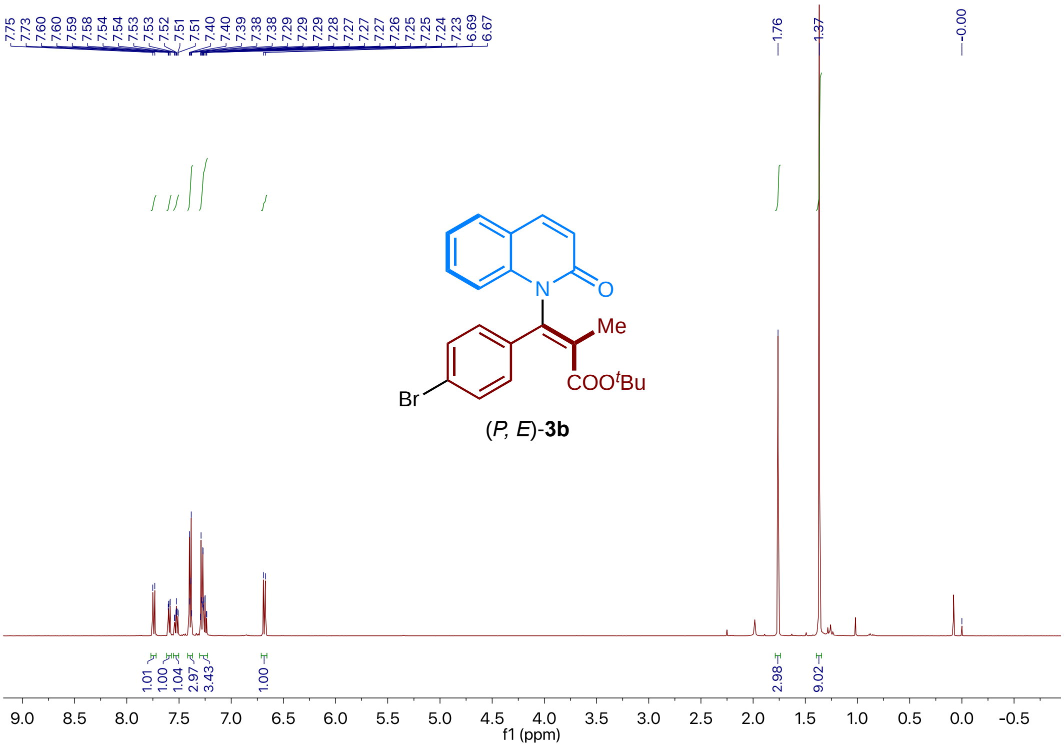

Supplementary Fig. 21. ^{1}H NMR spectrum of (*P*, *E*)-3b

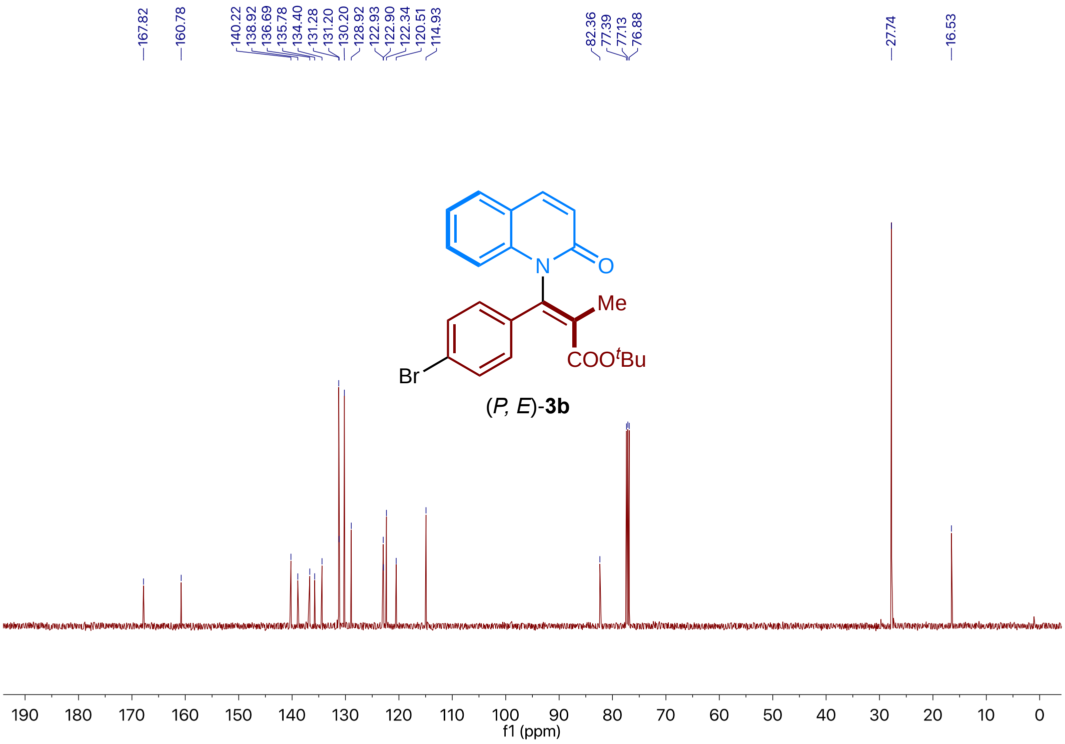

Supplementary Fig. 22. ^{13}C NMR spectrum of (*P*, *E*)-3b.

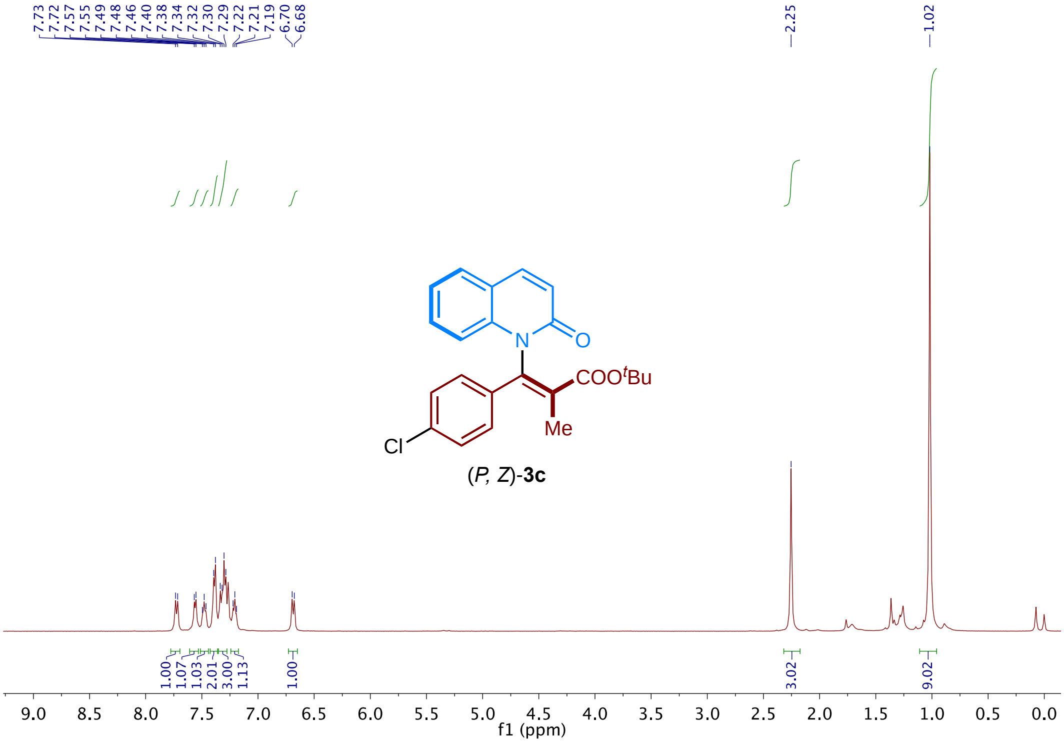

Supplementary Fig. 23. ^{1}H NMR spectrum of (*P*, *Z*)-3c

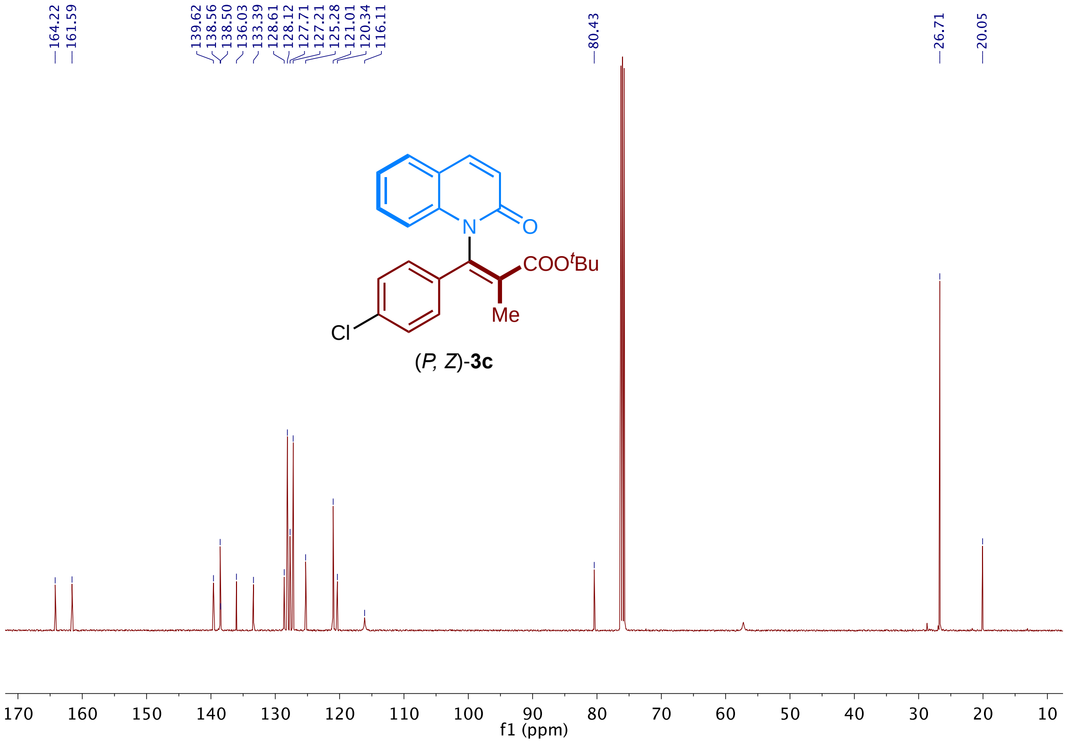

Supplementary Fig. 24. ^{13}C NMR spectrum of (*P*, *Z*)-3c.

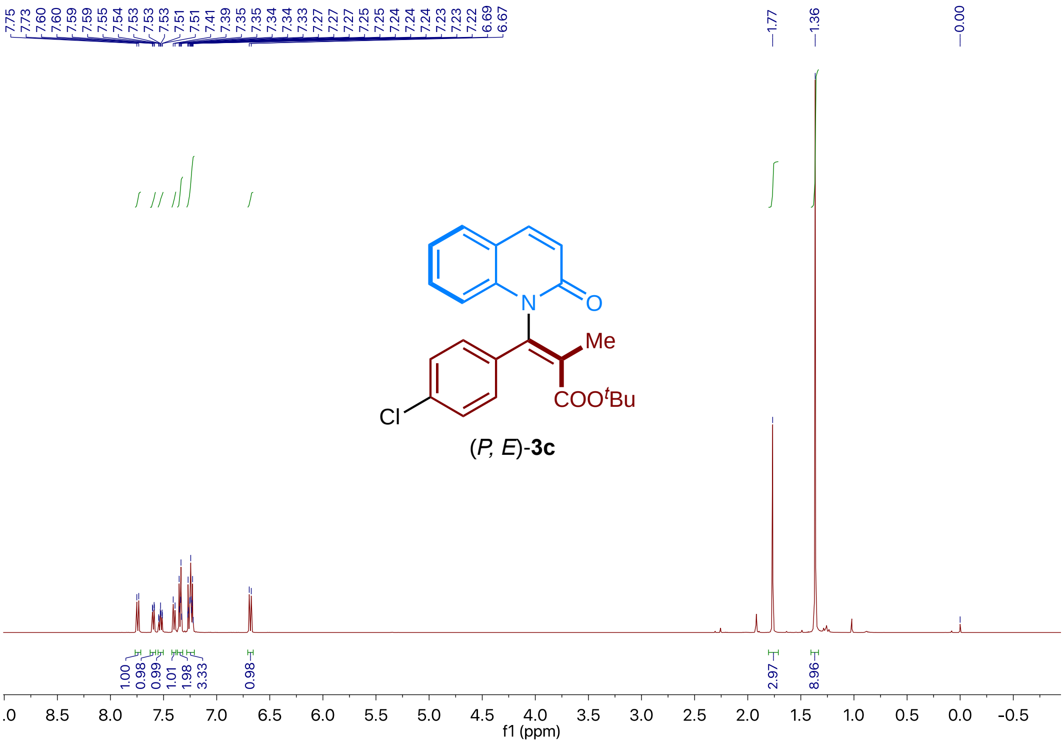

Supplementary Fig. 25. ^{1}H NMR spectrum of (*P*, *E*)-3c

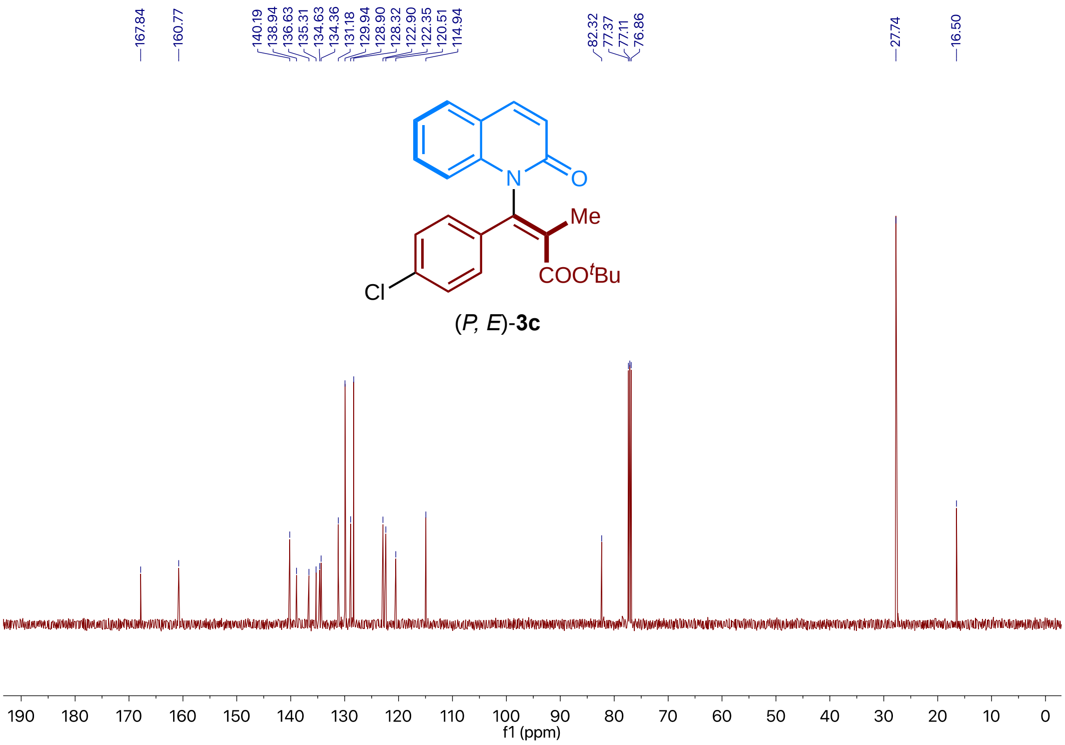

Supplementary Fig. 26. ^{13}C NMR spectrum of (*P*, *E*)-3c.

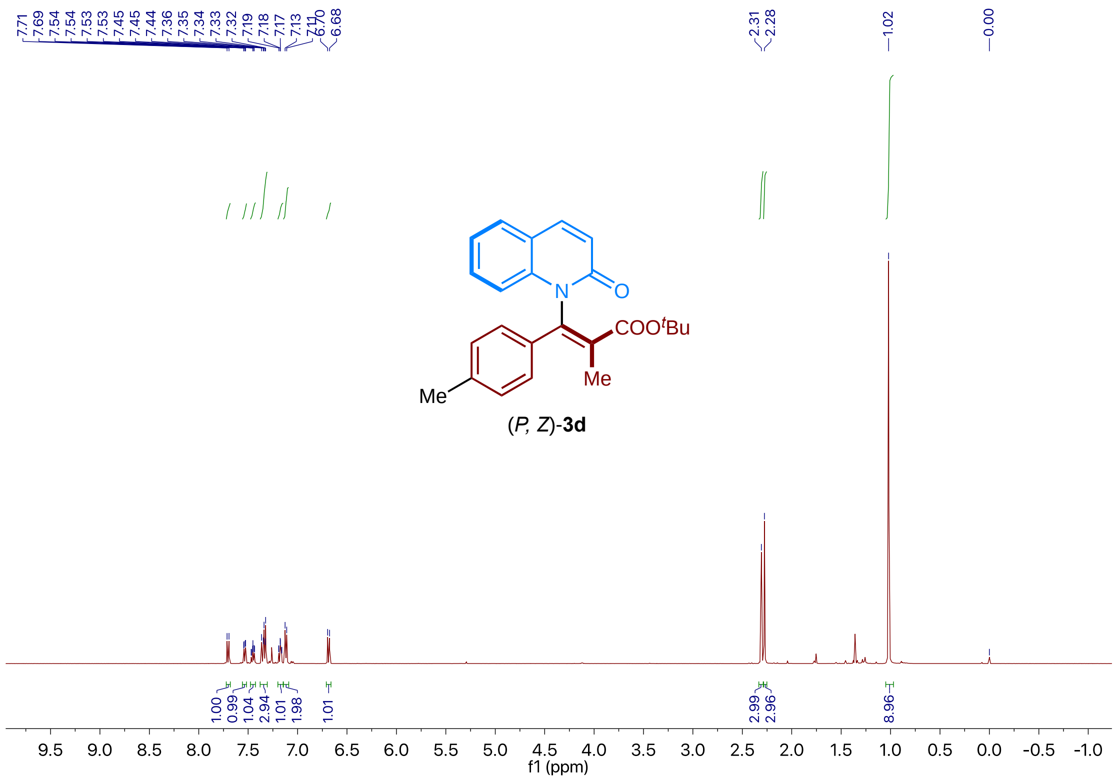

Supplementary Fig. 27. ^{1}H NMR spectrum of (*P*, *Z*)-3d

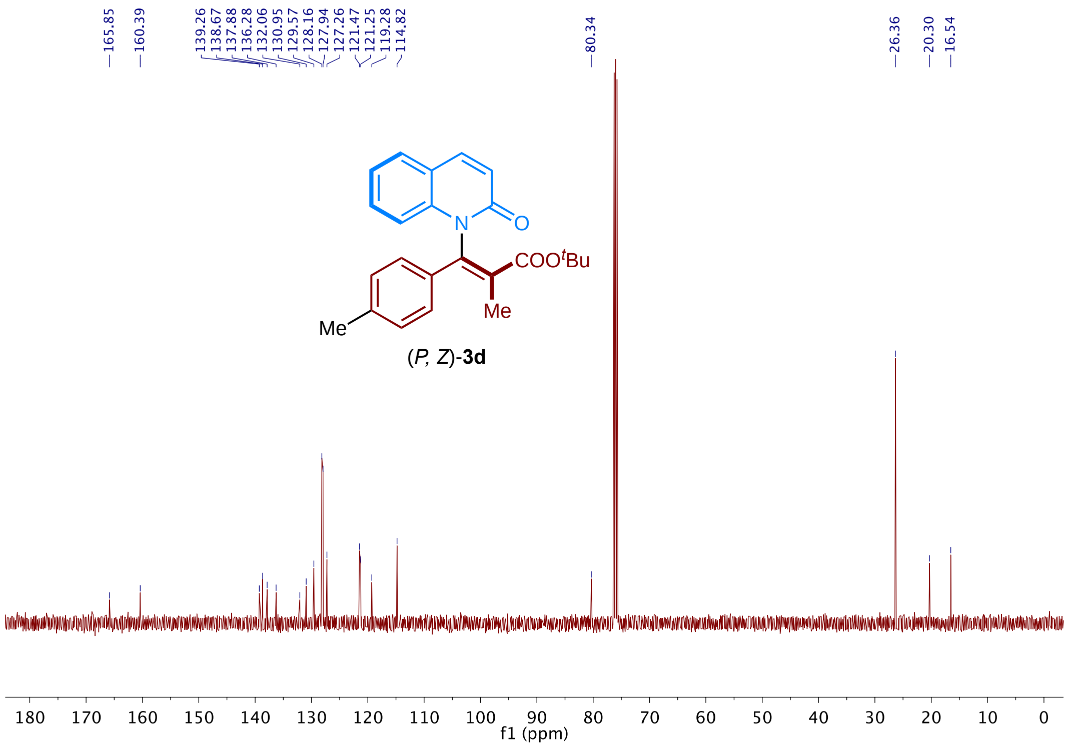

Supplementary Fig. 28. ^{13}C NMR spectrum of (*P*, *Z*)-3d.

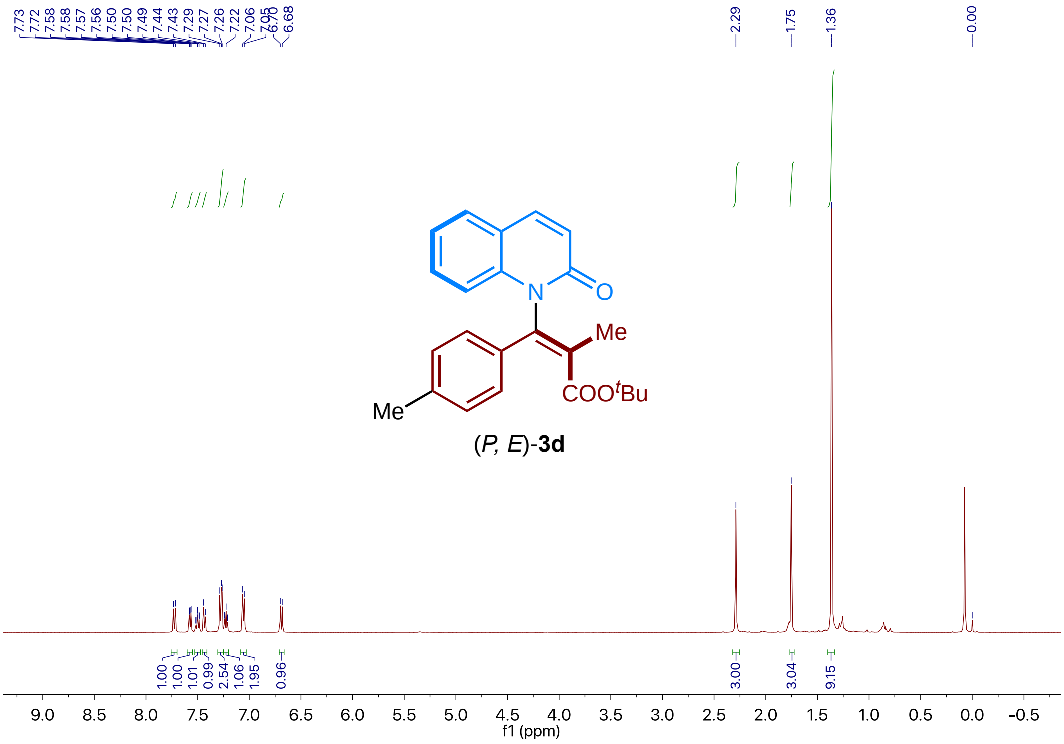

Supplementary Fig. 29. ^{1}H NMR spectrum of (*P*, *E*)-3d

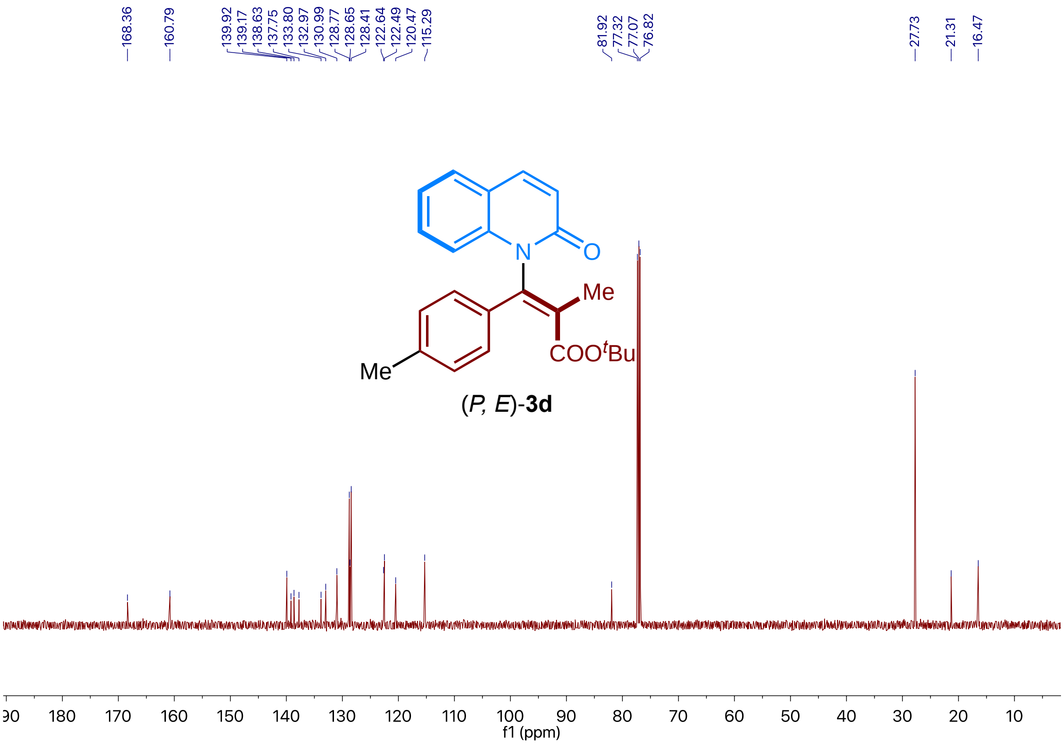

Supplementary Fig. 30. ^{13}C NMR spectrum of (*P*, *E*)-3d.

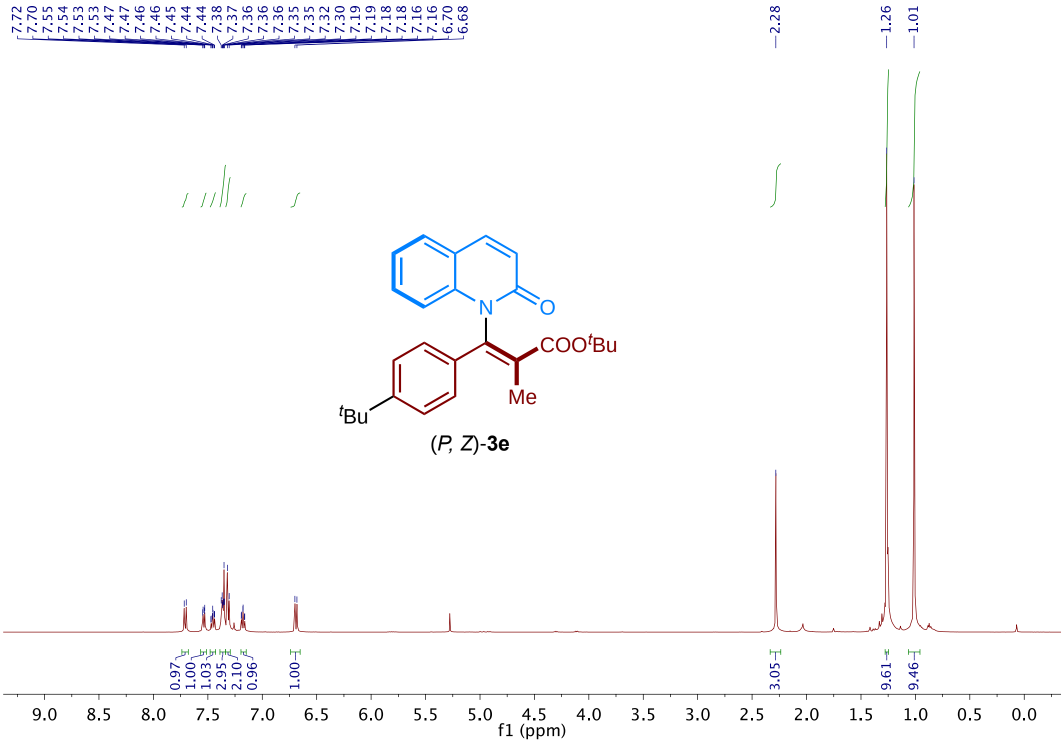

Supplementary Fig. 31. ^{1}H NMR spectrum of (*P*, *Z*)-3e

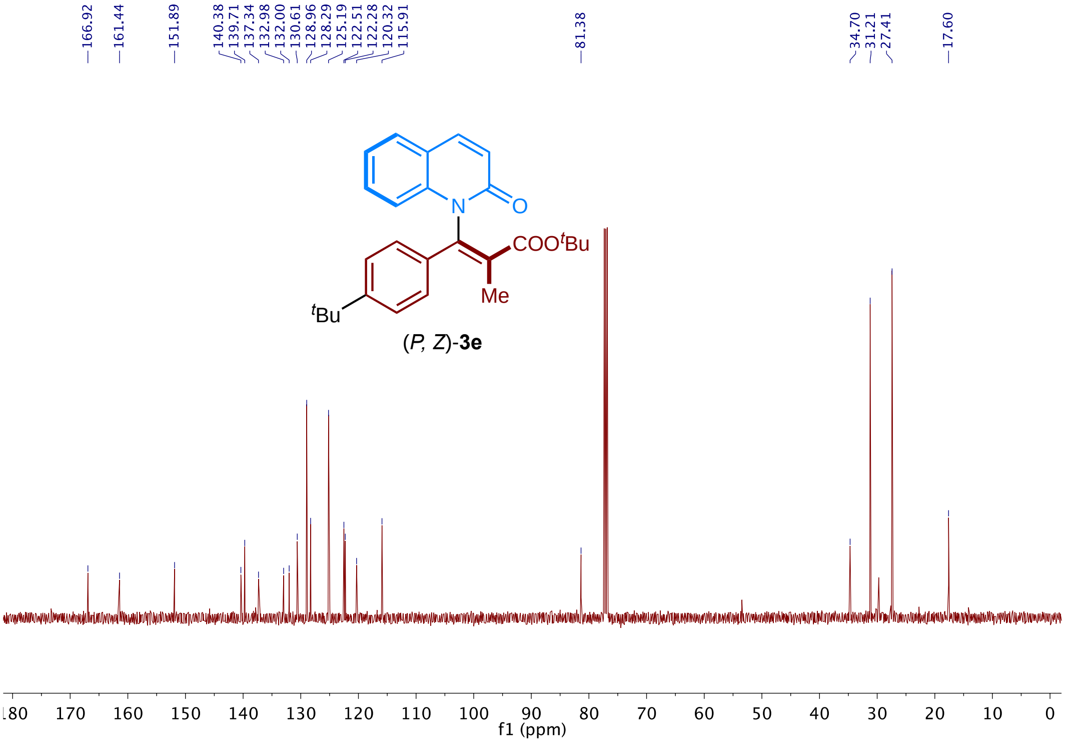

Supplementary Fig. 32. ^{13}C NMR spectrum of (*P*, *Z*)-3e.

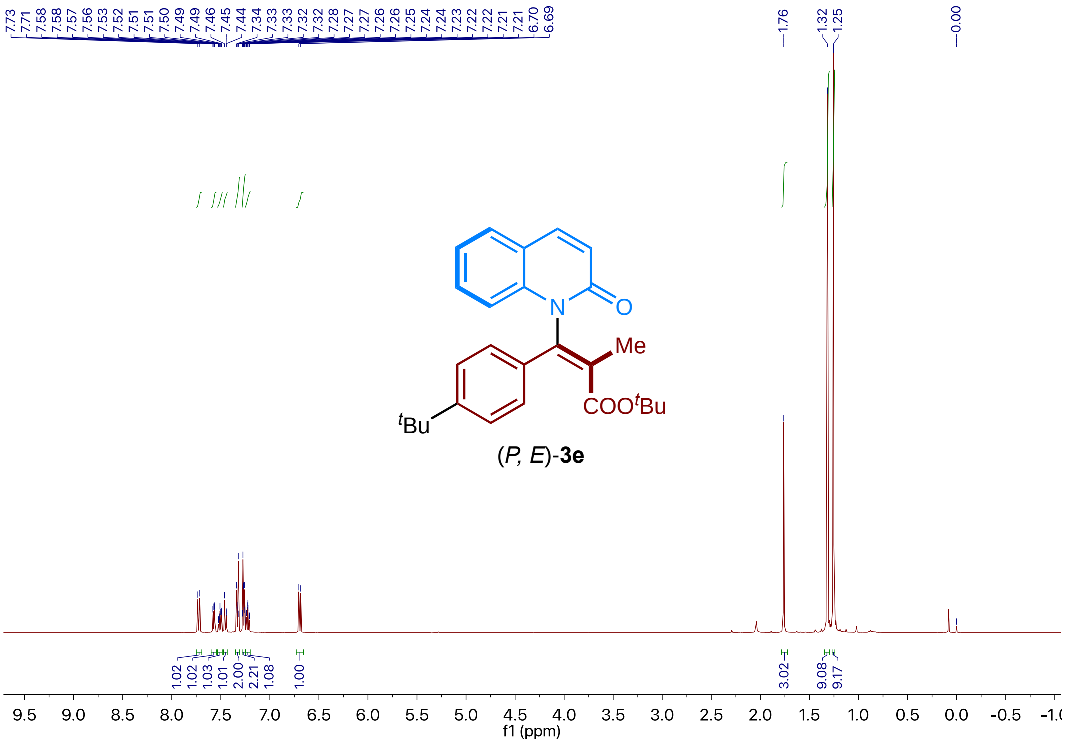

Supplementary Fig. 33. ^{1}**H NMR spectrum of (*P*, *E*)-3e**

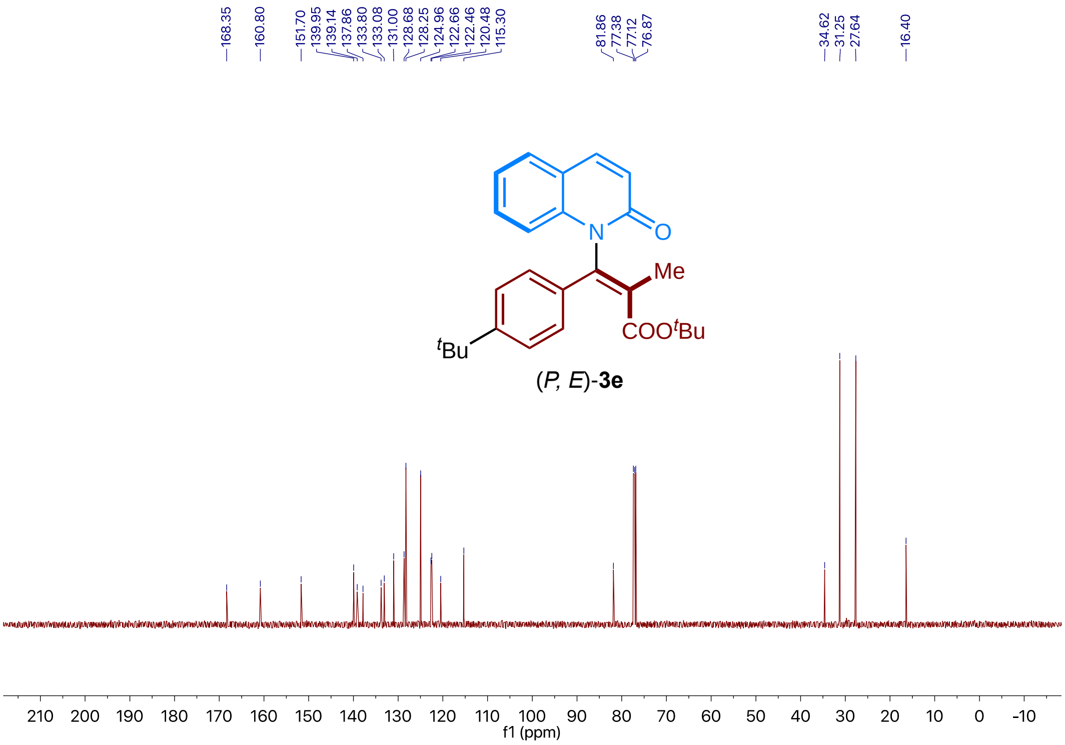

Supplementary Fig. 34. ^{13}**C NMR spectrum of (*P*, *E*)-3e.**

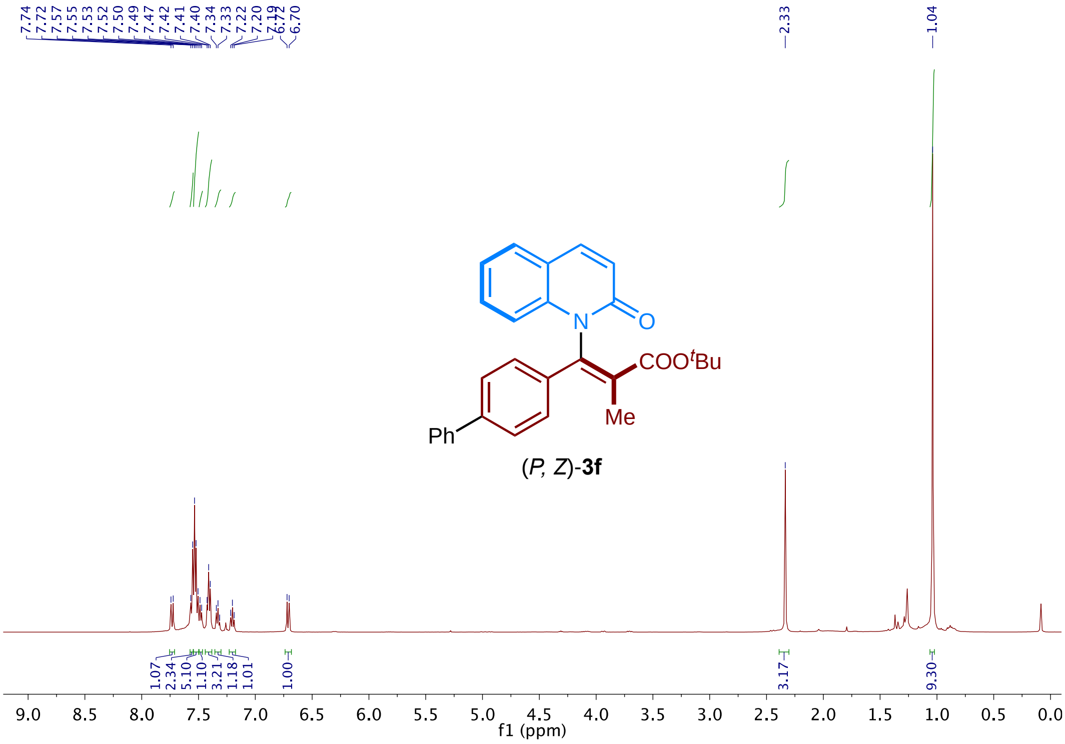

Supplementary Fig. 35. ^{1}H NMR spectrum of (*P*, *Z*)-3f

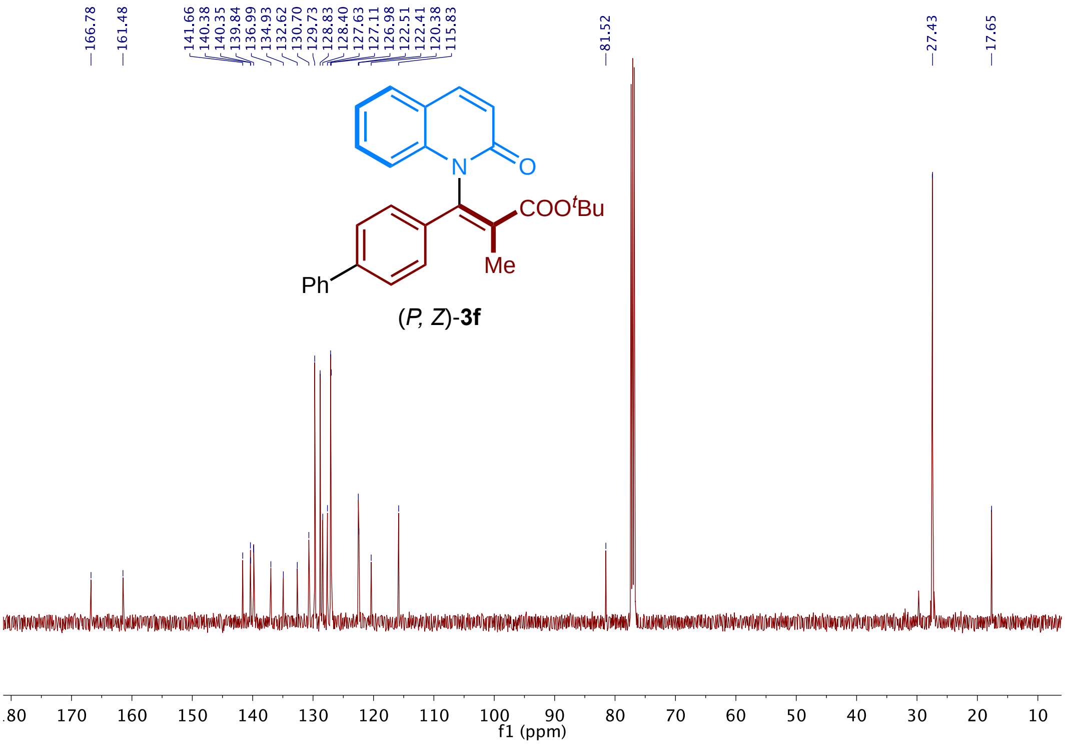

Supplementary Fig. 36. ^{13}C NMR spectrum of (*P*, *Z*)-3f.

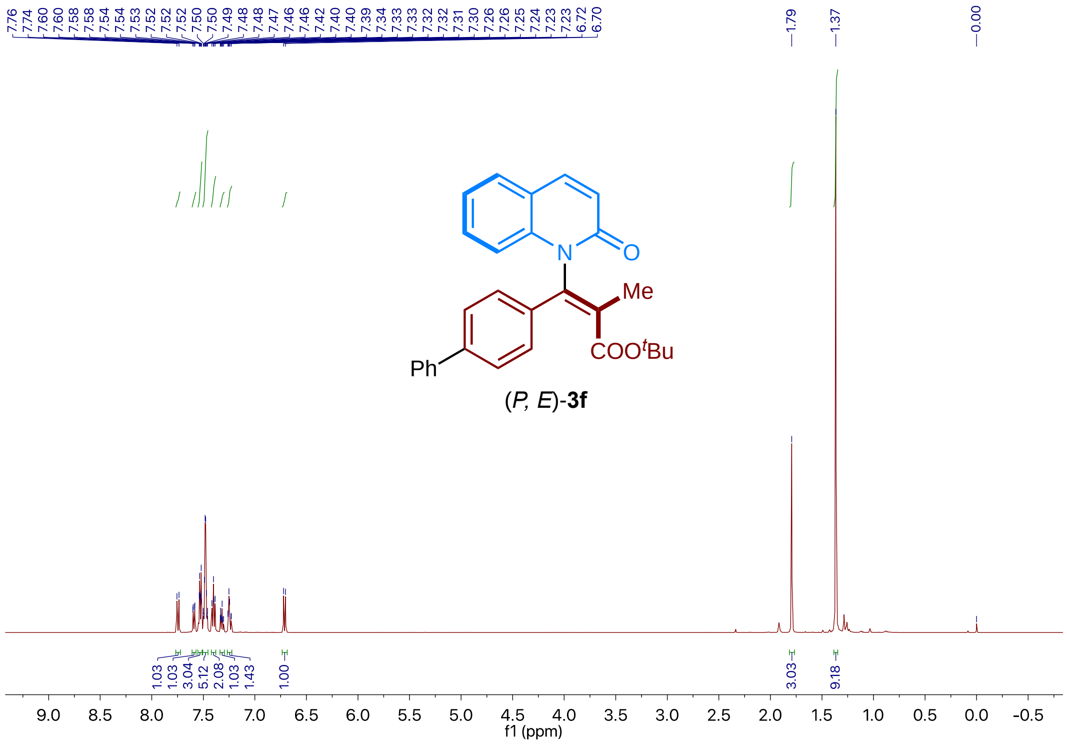

Supplementary Fig. 37. ^{1}H NMR spectrum of (*P*, *E*)-3f

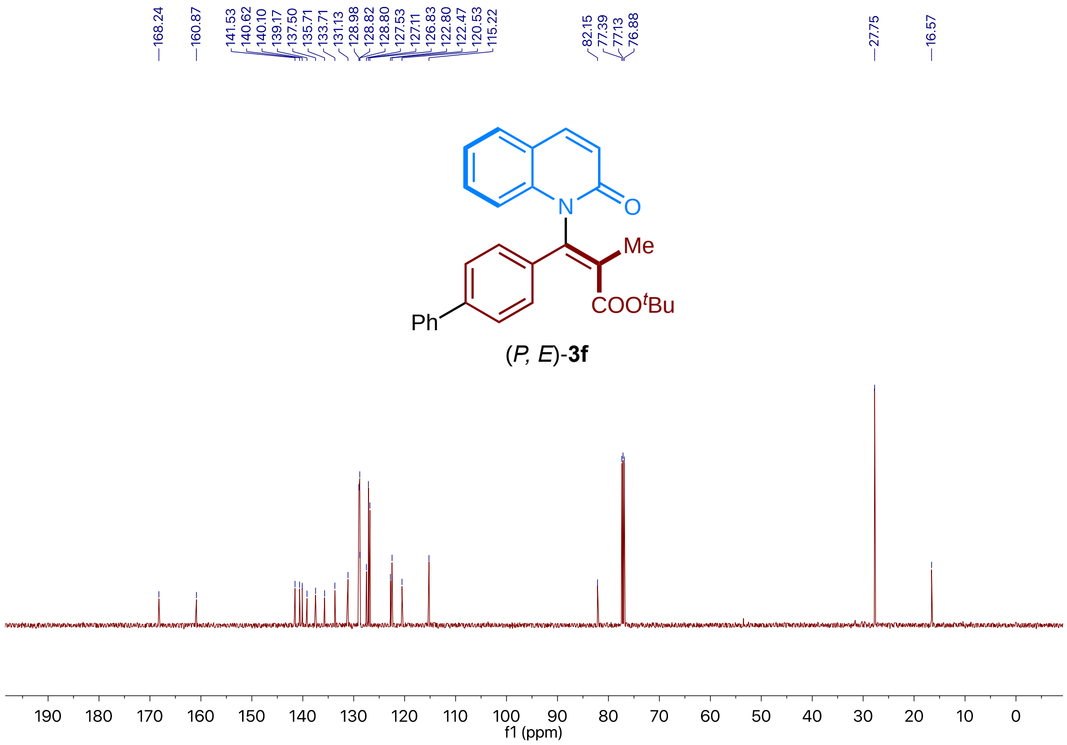

Supplementary Fig. 38. ^{13}C NMR spectrum of (*P*, *E*)-3f.

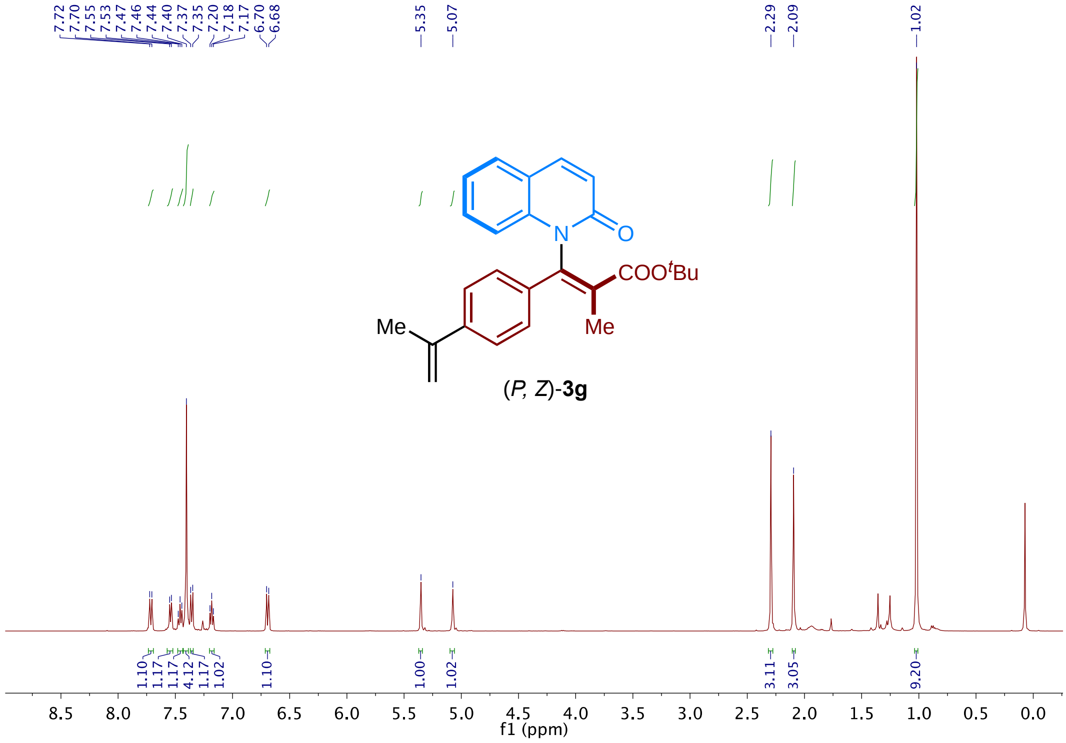

Supplementary Fig. 39. ^{1}H NMR spectrum of (*P*, *Z*)-3g

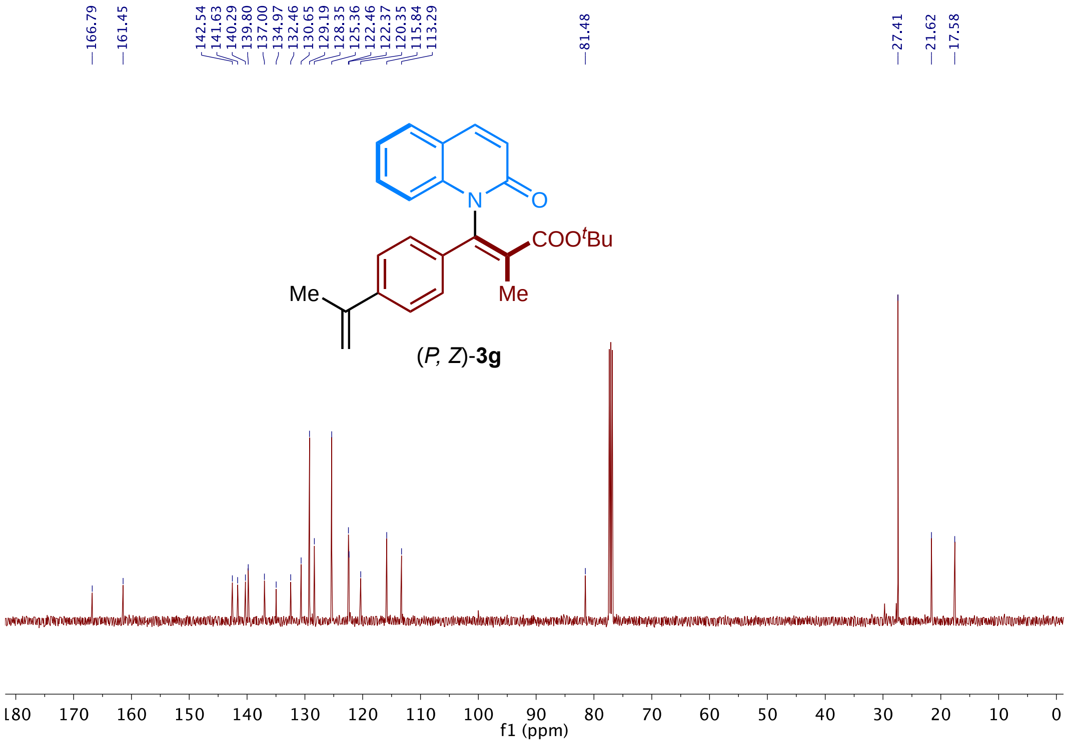

Supplementary Fig. 40. ^{13}C NMR spectrum of (*P*, *Z*)-3g.

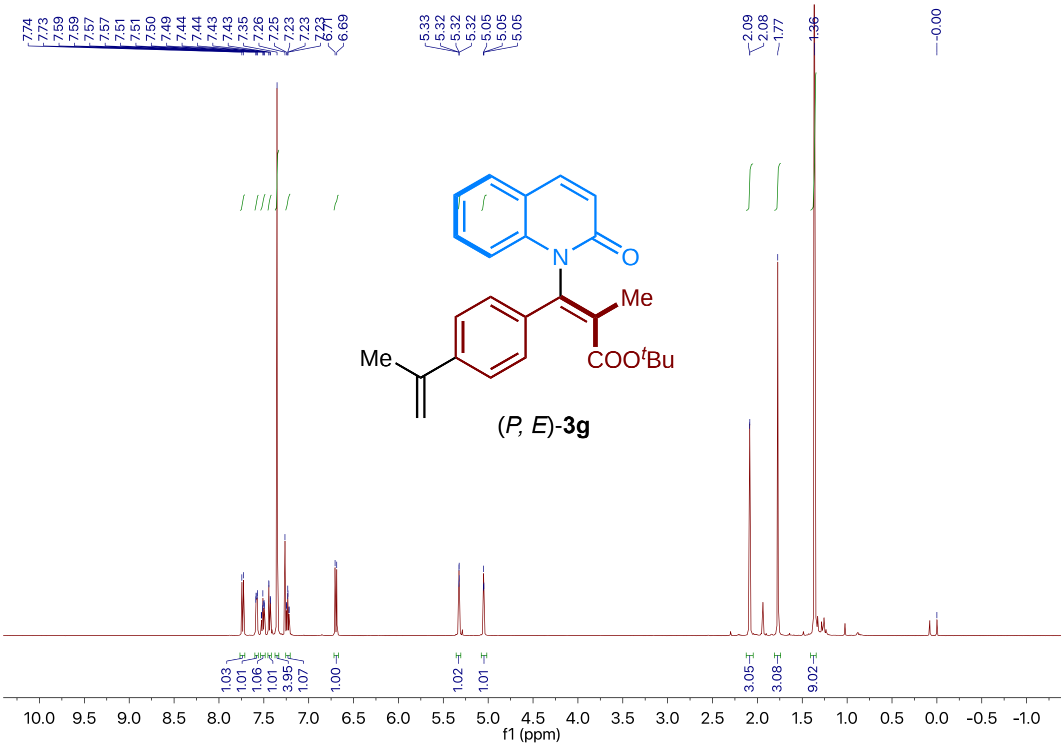

Supplementary Fig. 41. ^{1}**H NMR spectrum of (*P*, *E*)-3g**

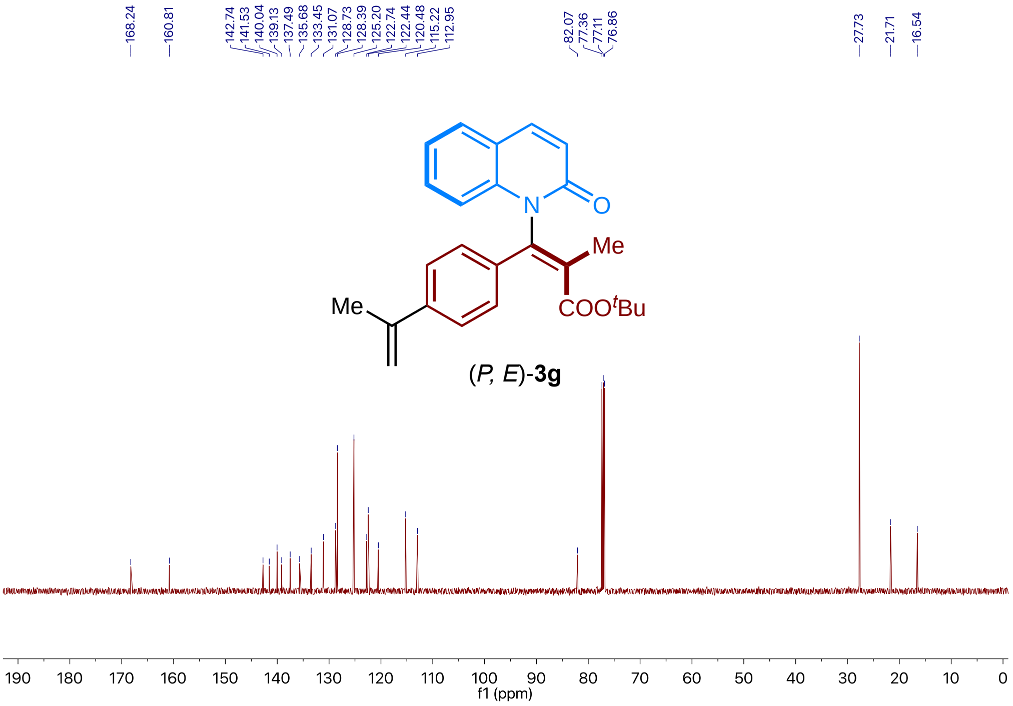

Supplementary Fig. 42. ^{13}**C NMR spectrum of (*P*, *E*)-3g.**

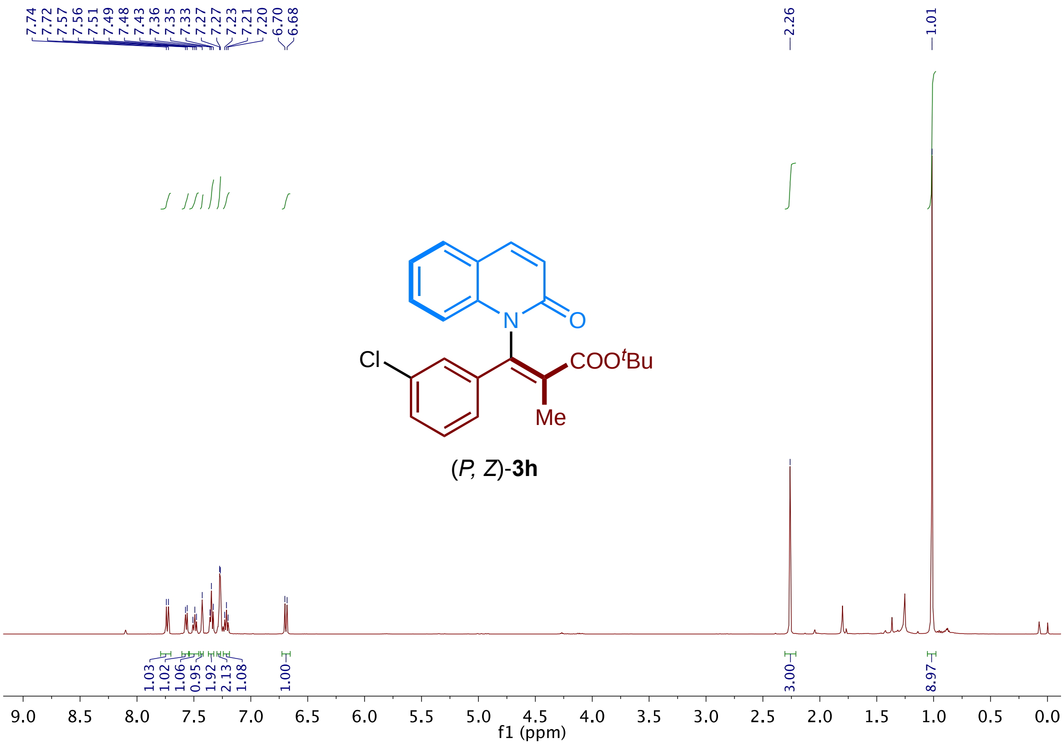

Supplementary Fig. 43. ^{1}H NMR spectrum of (*P*, *Z*)-3h

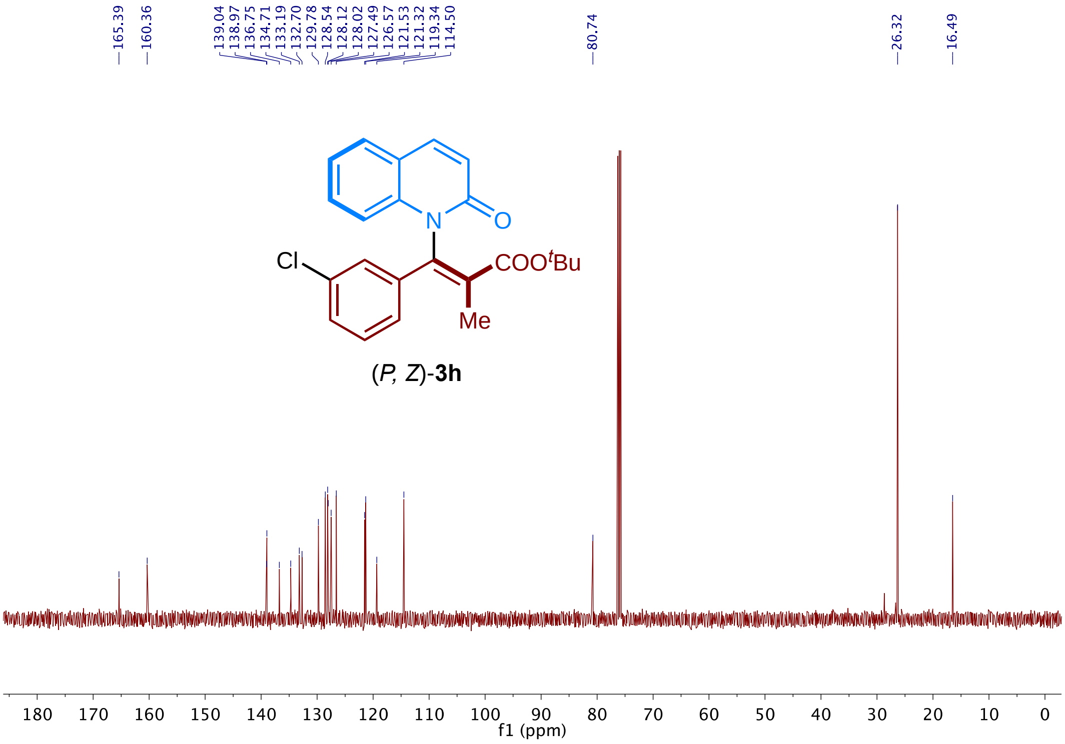

Supplementary Fig. 44. ^{13}C NMR spectrum of (*P*, *Z*)-3h.

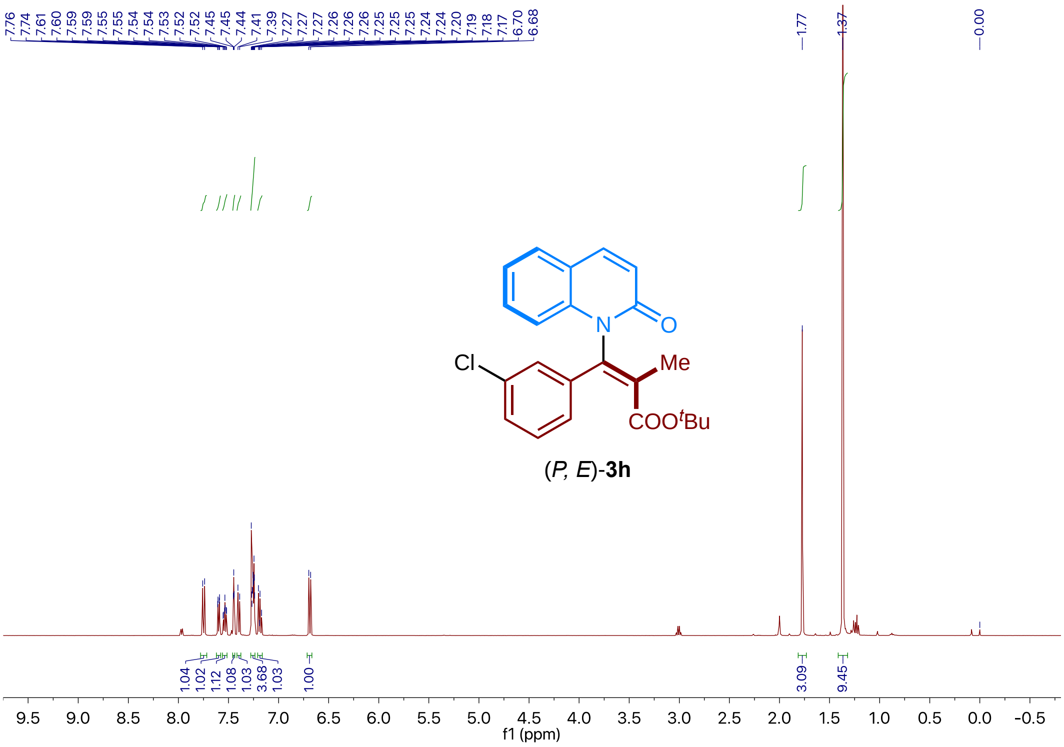

Supplementary Fig. 45. ^{1}H NMR spectrum of (*P*, *E*)-3h

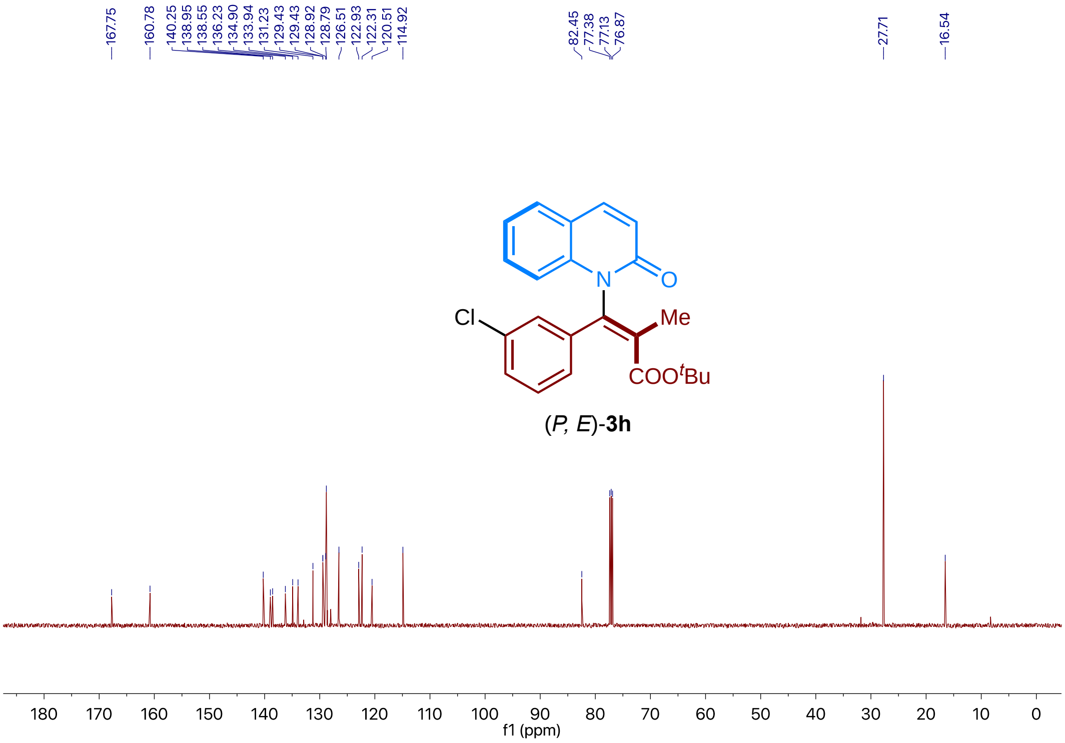

Supplementary Fig. 46. ^{13}C NMR spectrum of (*P*, *E*)-3h.

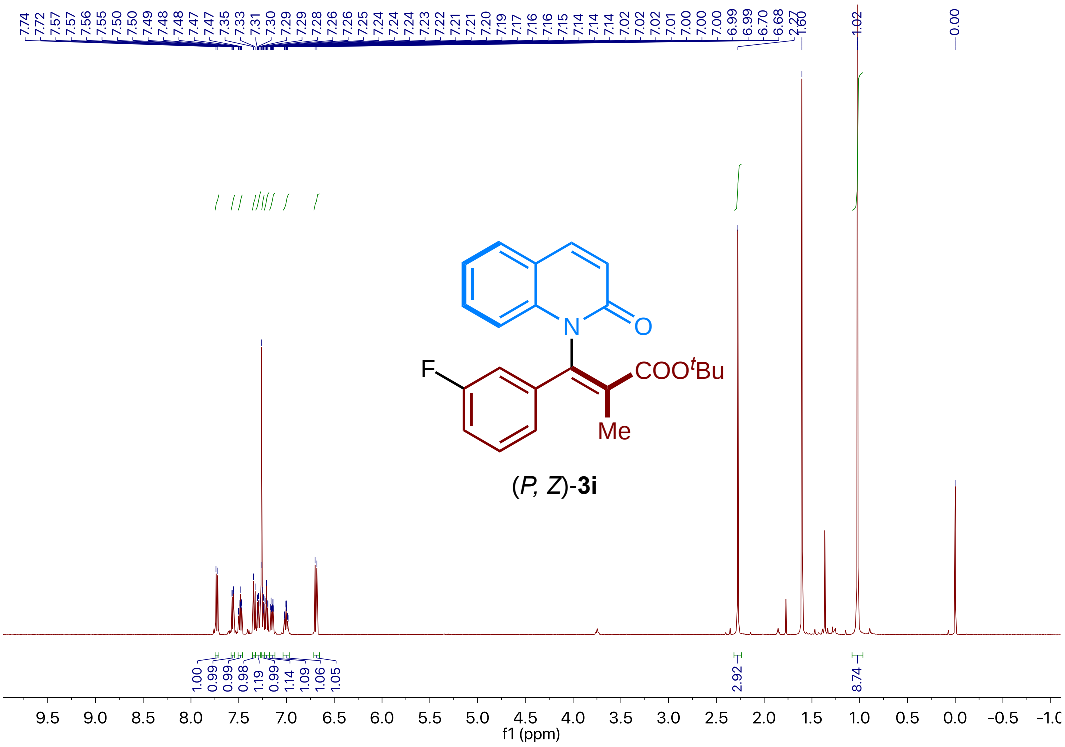

Supplementary Fig. 47. ^{1}H NMR spectrum of (*P*, *Z*)-3i

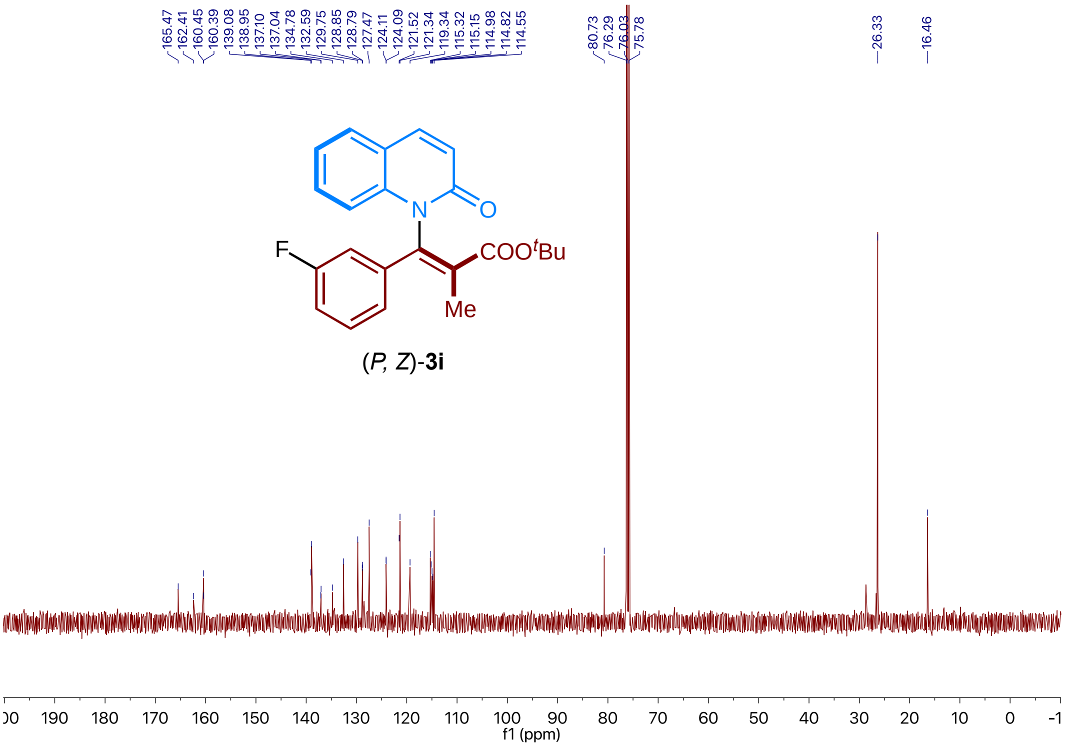

Supplementary Fig. 48. ^{13}C NMR spectrum of (*P*, *Z*)-3i.

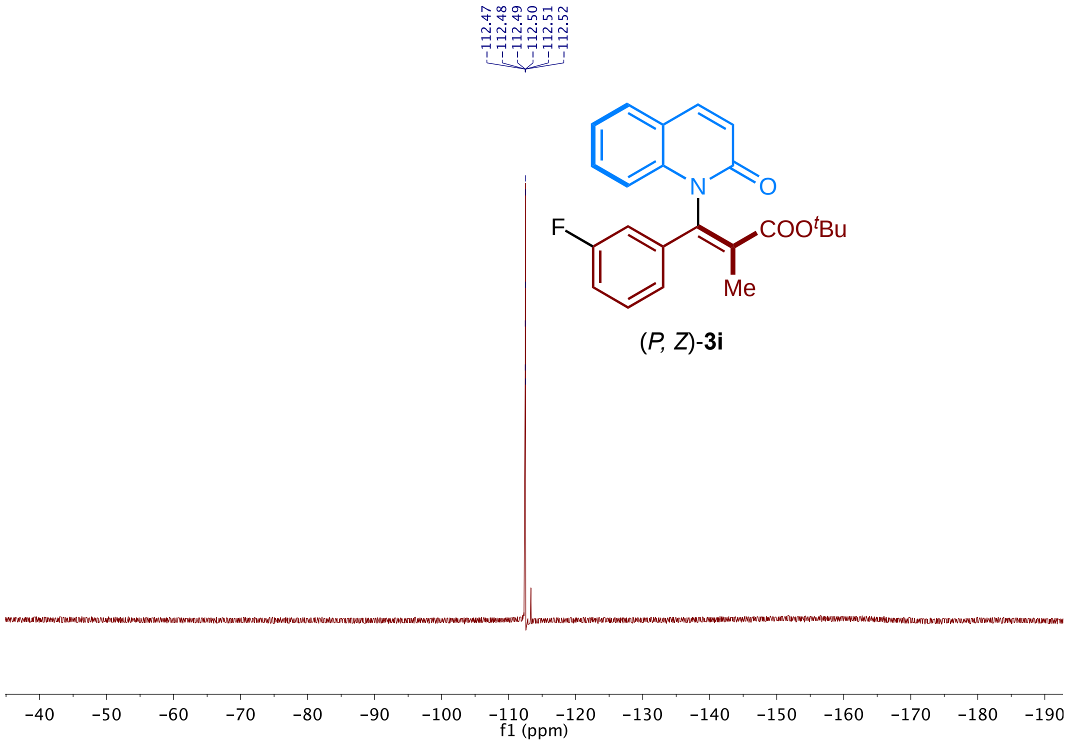

Supplementary Fig. 49. ^{19}F NMR spectrum of (*P*, *Z*)-3i.

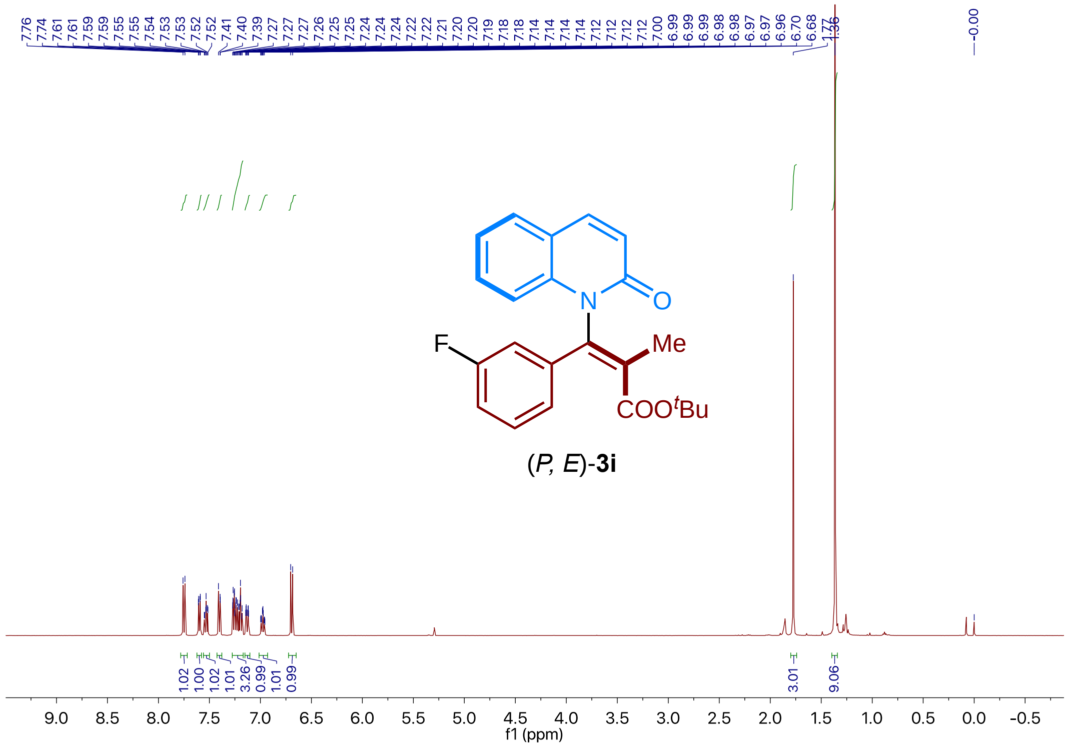

Supplementary Fig. 50. ^{1}H NMR spectrum of (*P*, *E*)-3i

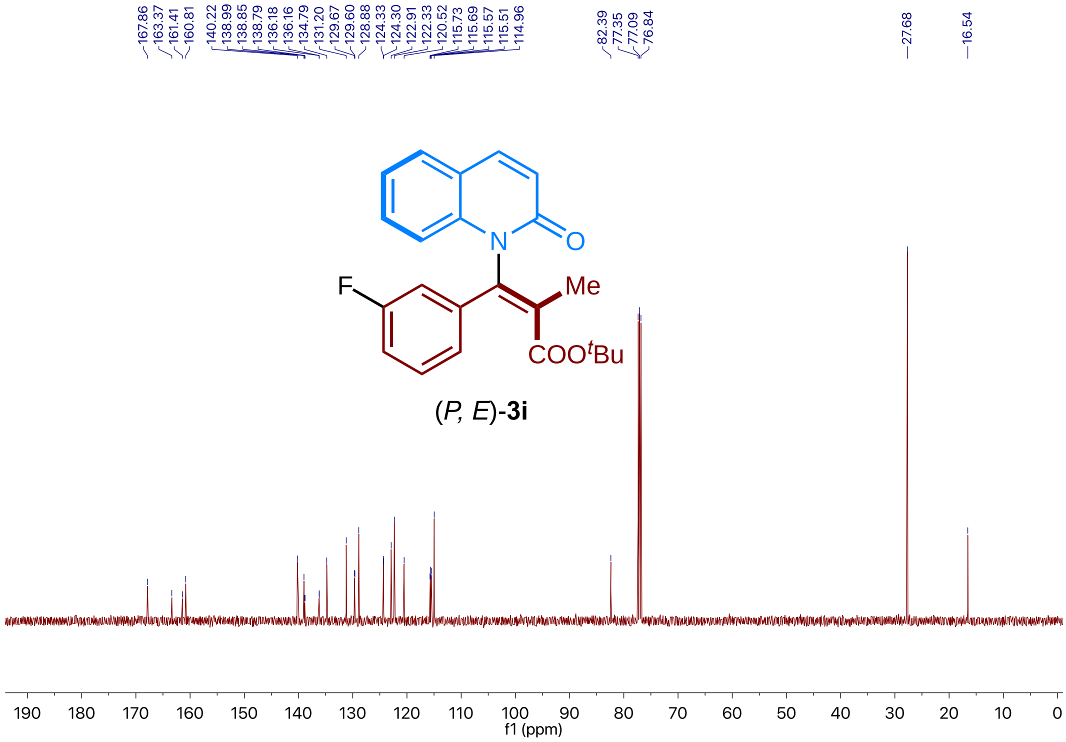

Supplementary Fig. 51. ^{13}C NMR spectrum of (*P*, *E*)-3i.

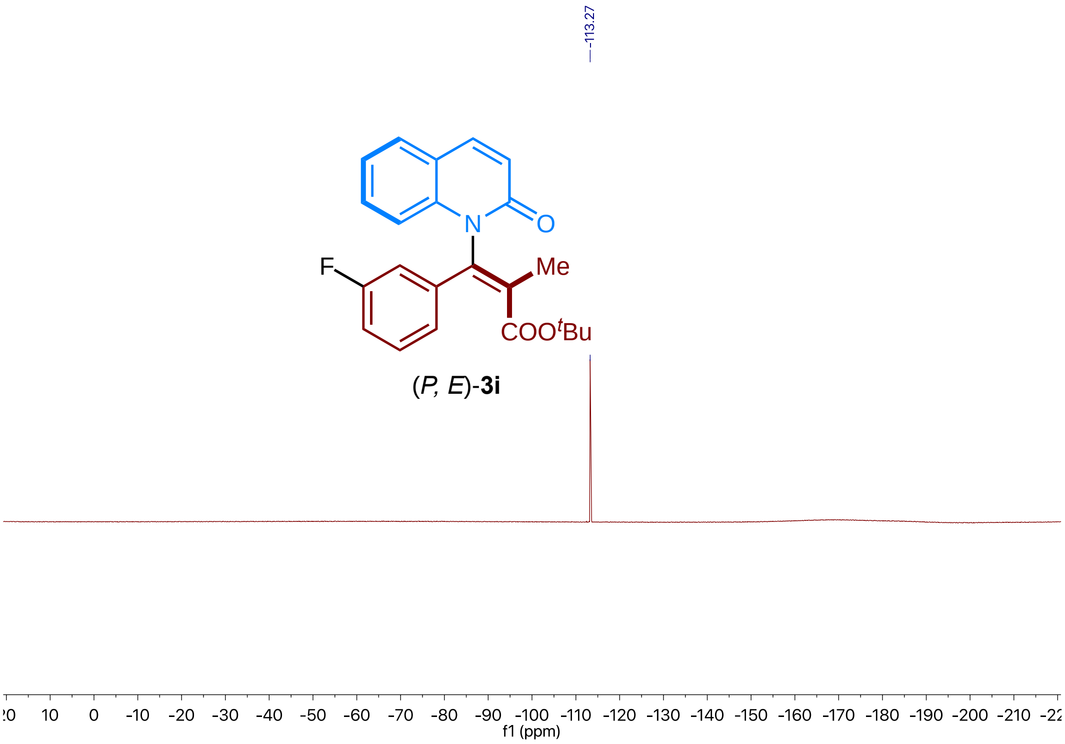

Supplementary Fig. 52. ^{19}F NMR spectrum of (*P*, *E*)-3i.

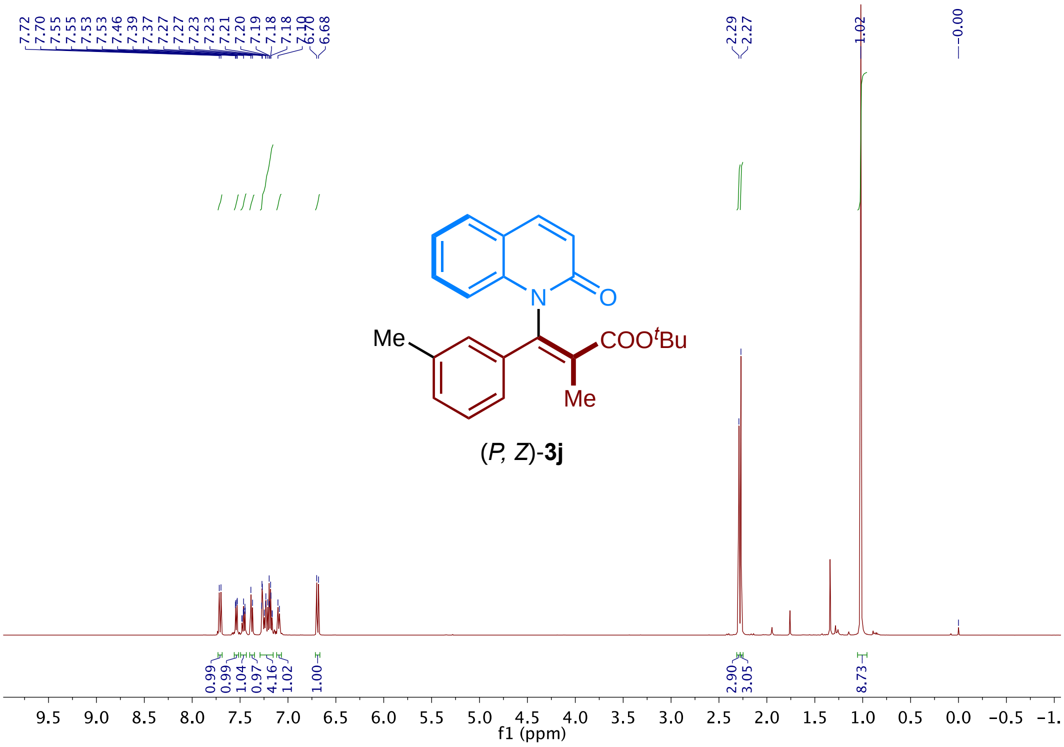

Supplementary Fig. 53. ^{1}H NMR spectrum of (*P*, *Z*)-3j

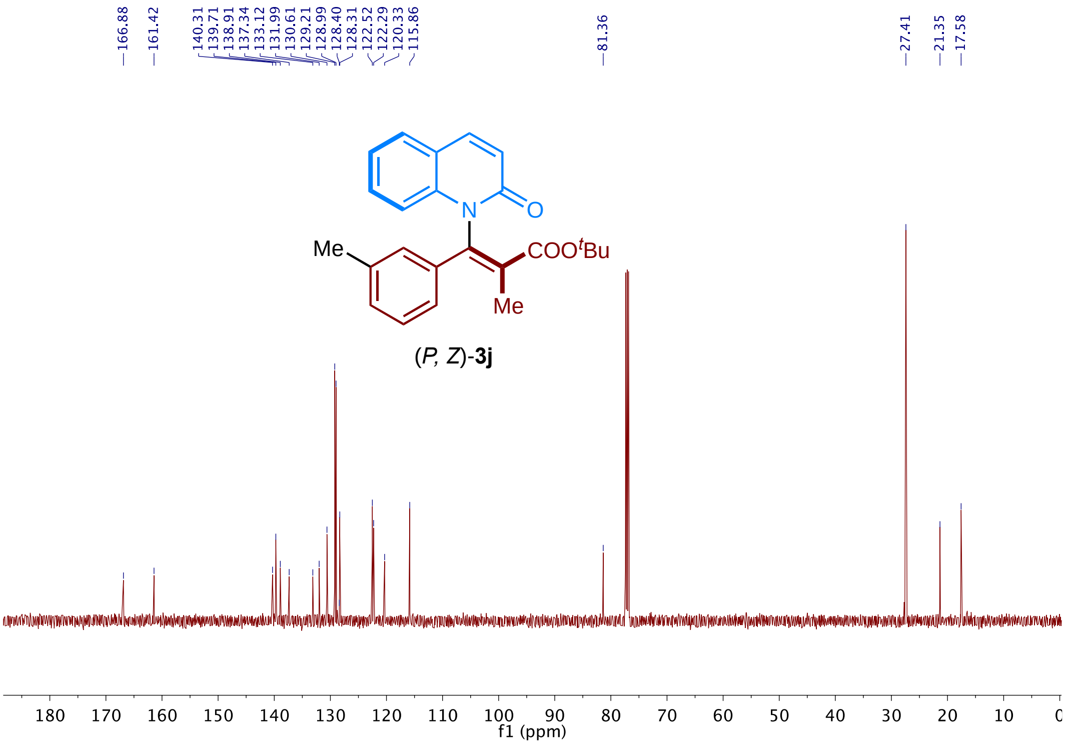

Supplementary Fig. 54. ^{13}C NMR spectrum of (*P*, *Z*)-3j.

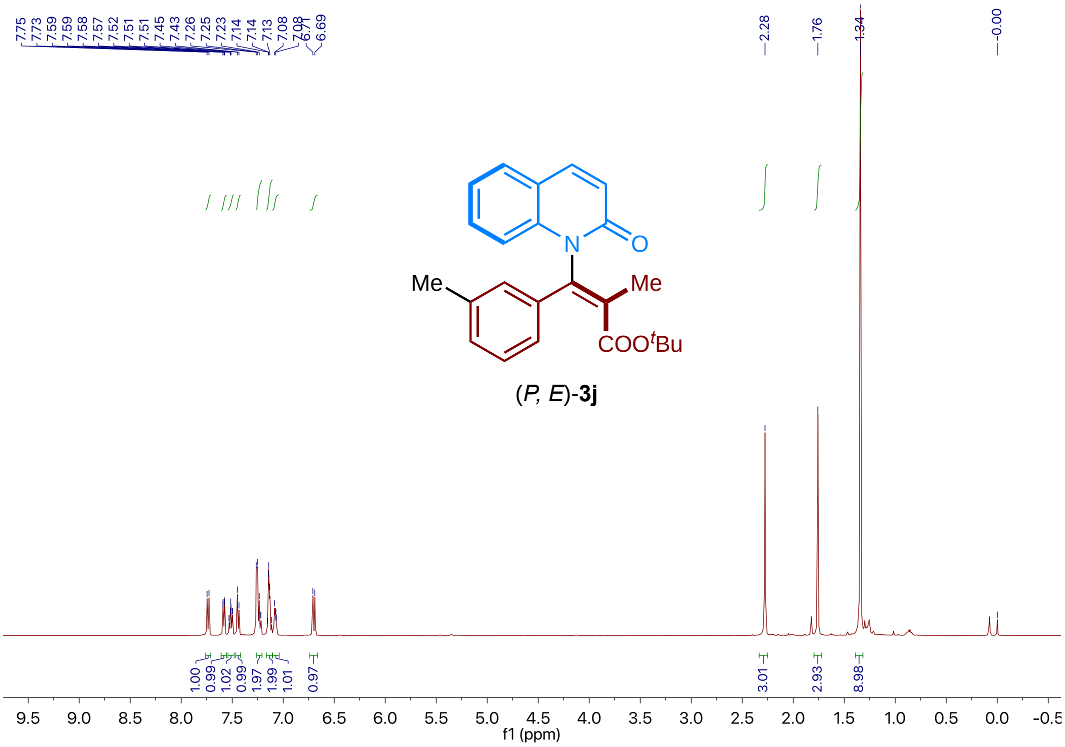

Supplementary Fig. 55. ^{1}H NMR spectrum of (*P*, *E*)-3j

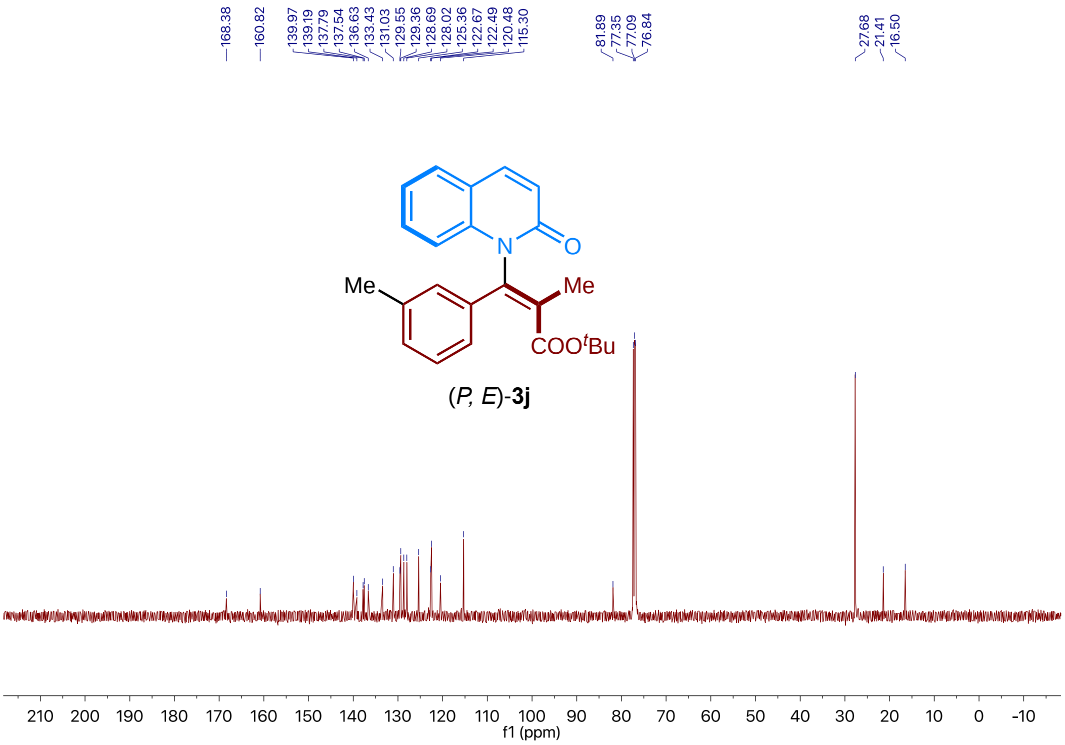

Supplementary Fig. 56. ^{13}C NMR spectrum of (*P*, *E*)-3j.

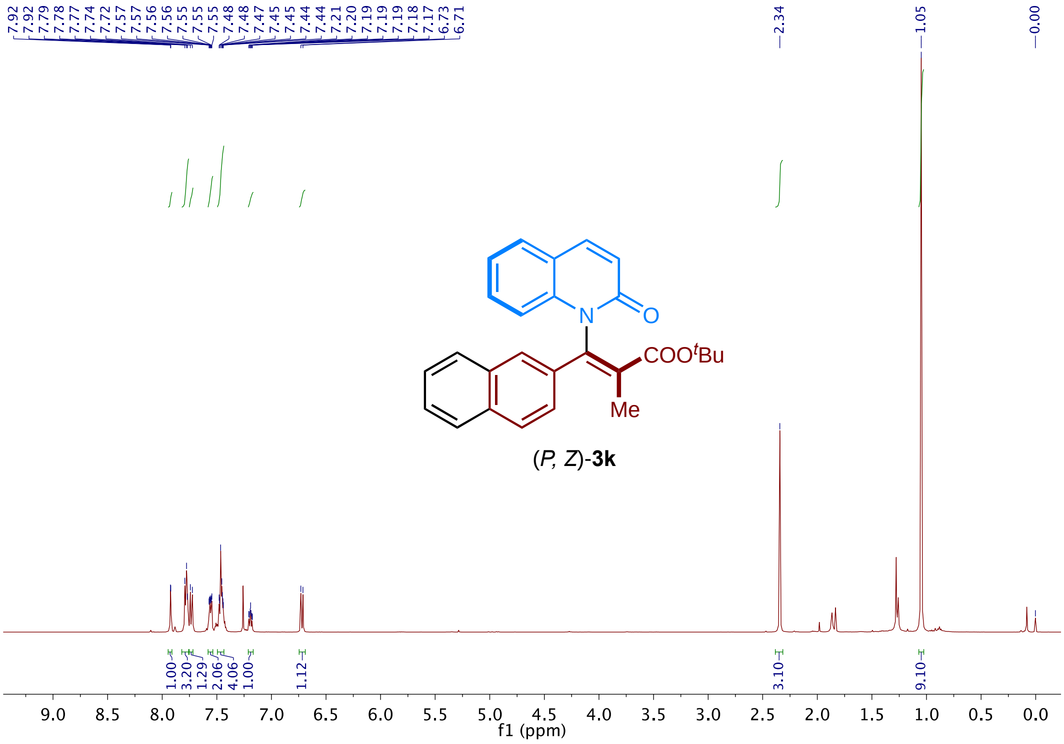

Supplementary Fig. 57. ^{1}H NMR spectrum of (*P*, *Z*)-3k

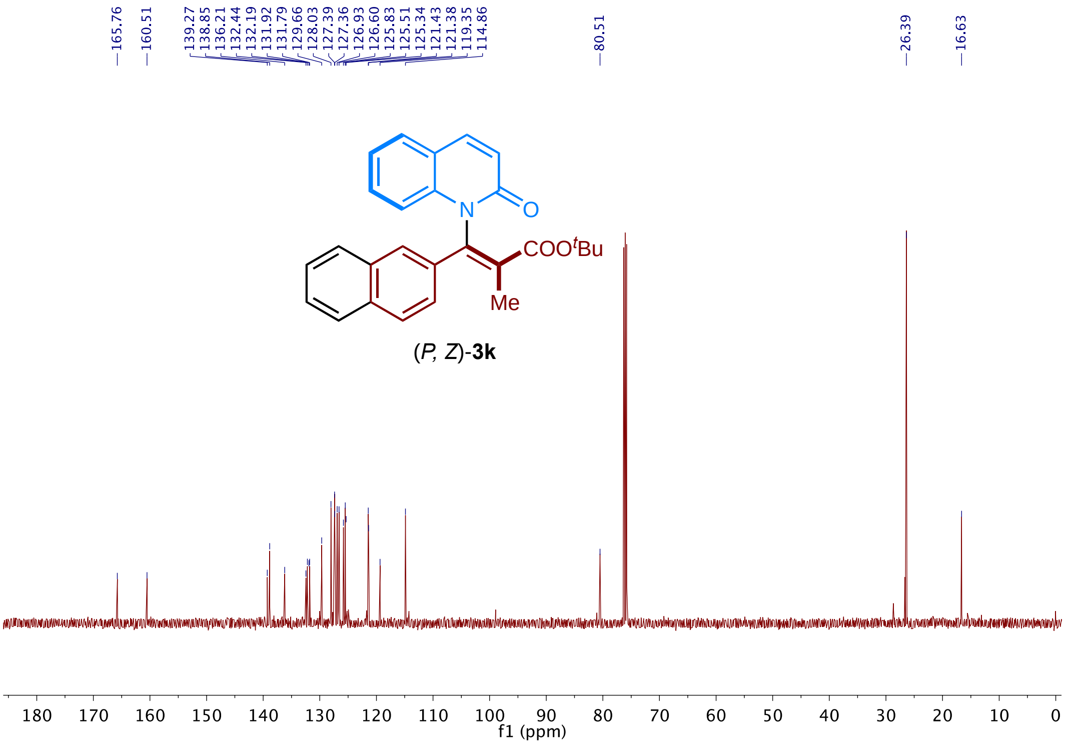

Supplementary Fig. 58. ^{13}C NMR spectrum of (*P*, *Z*)-3k.

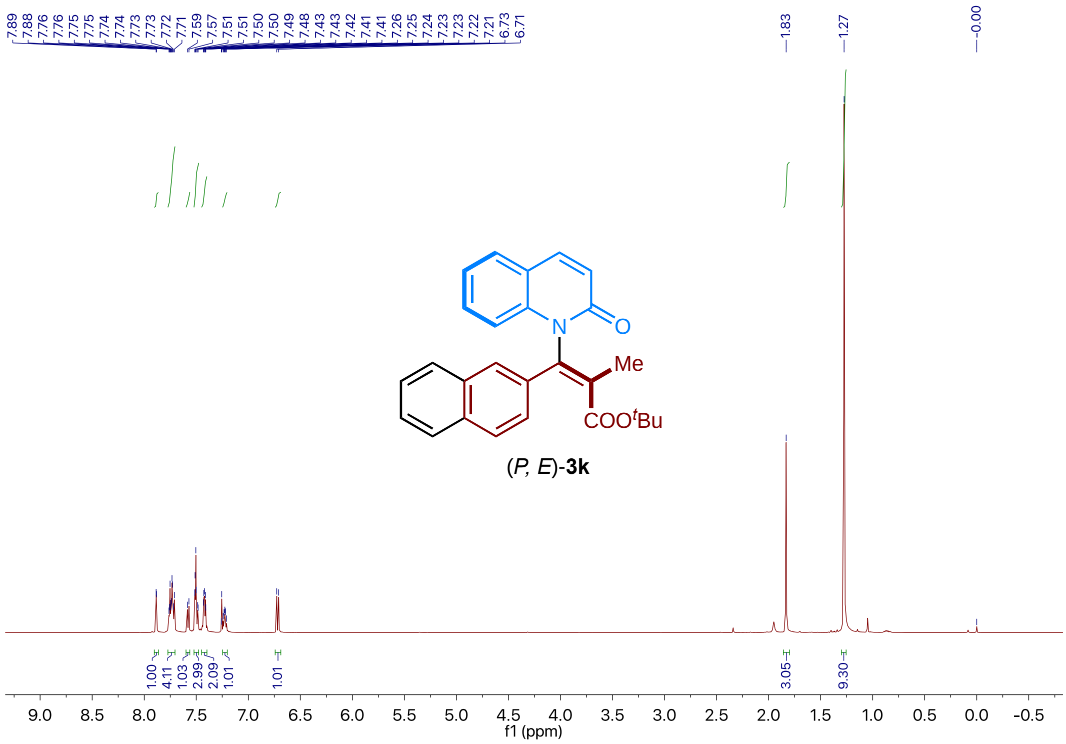

Supplementary Fig. 59. ^{1}H NMR spectrum of (*P*, *E*)-3k

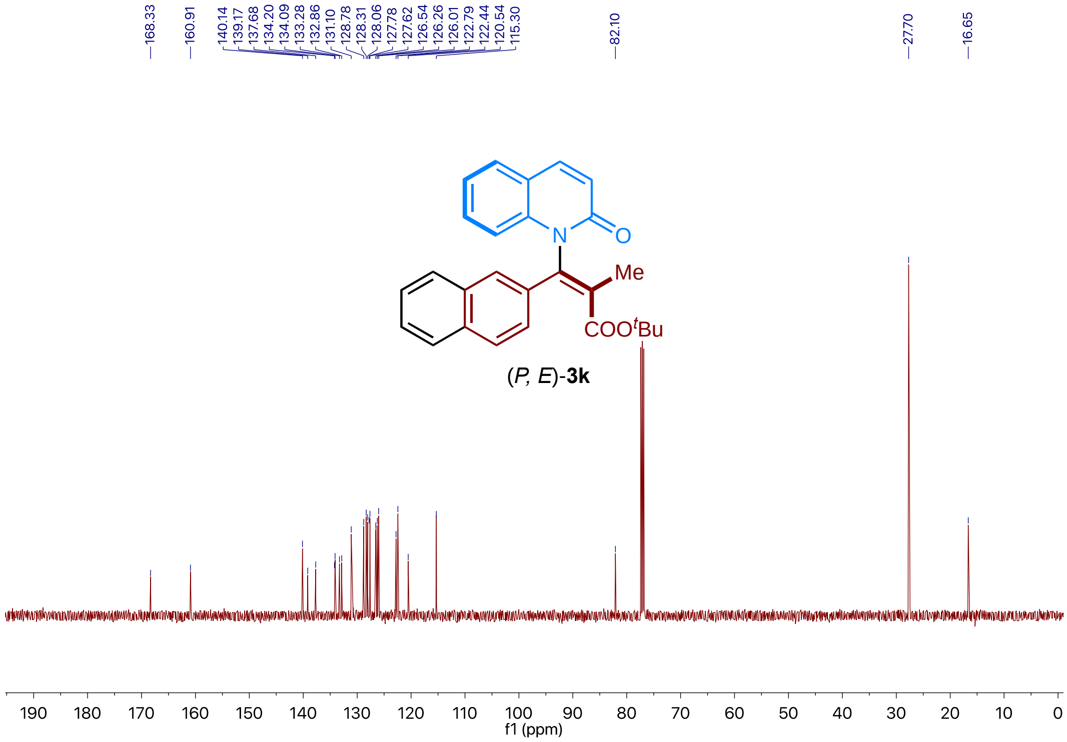

Supplementary Fig. 60. ^{13}C NMR spectrum of (*P*, E)-3k.

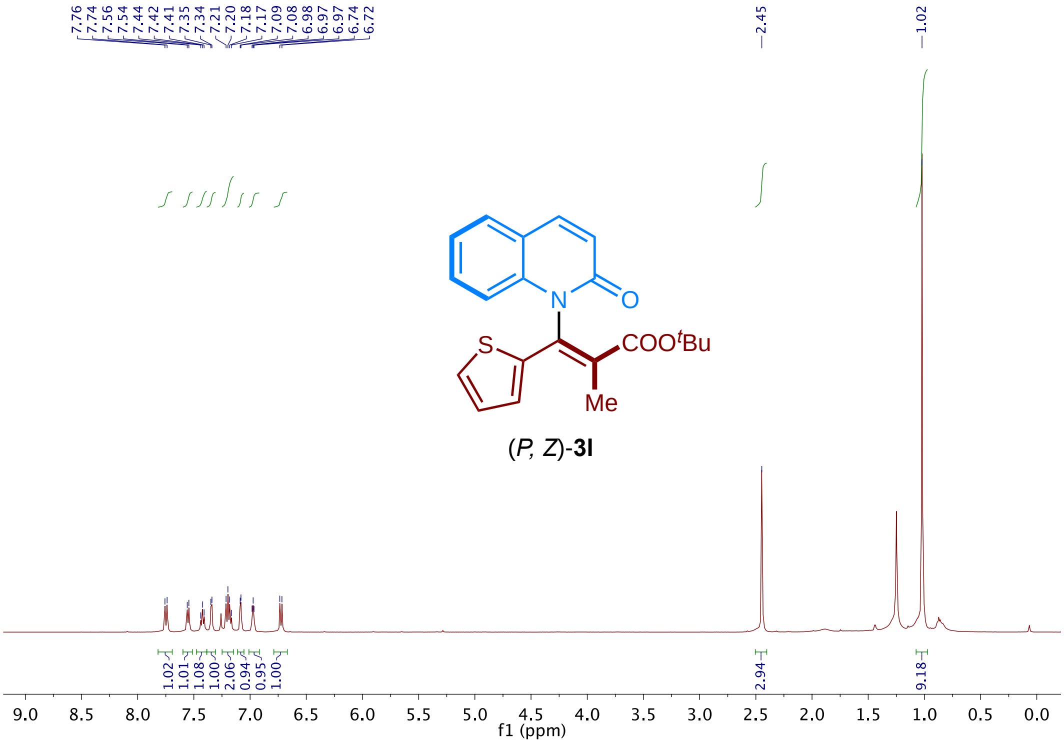

Supplementary Fig. 61. ^{1}H NMR spectrum of (*P*, *Z*)-3l

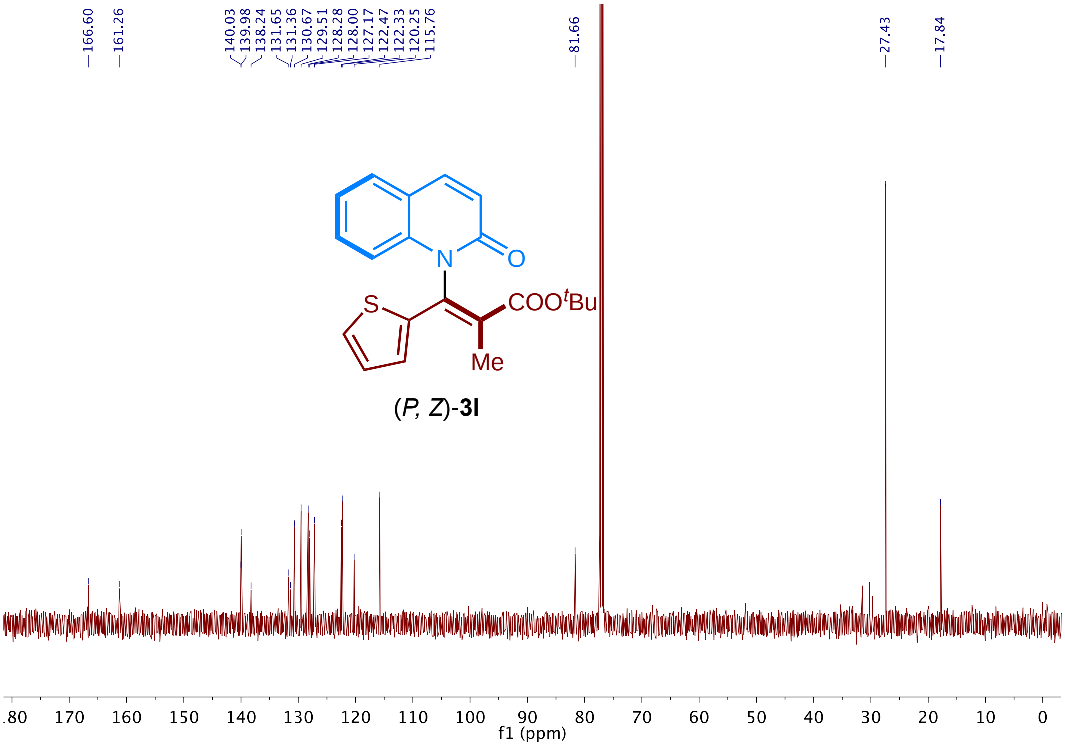

Supplementary Fig. 62. ^{13}C NMR spectrum of (*P*, *Z*)-3l.

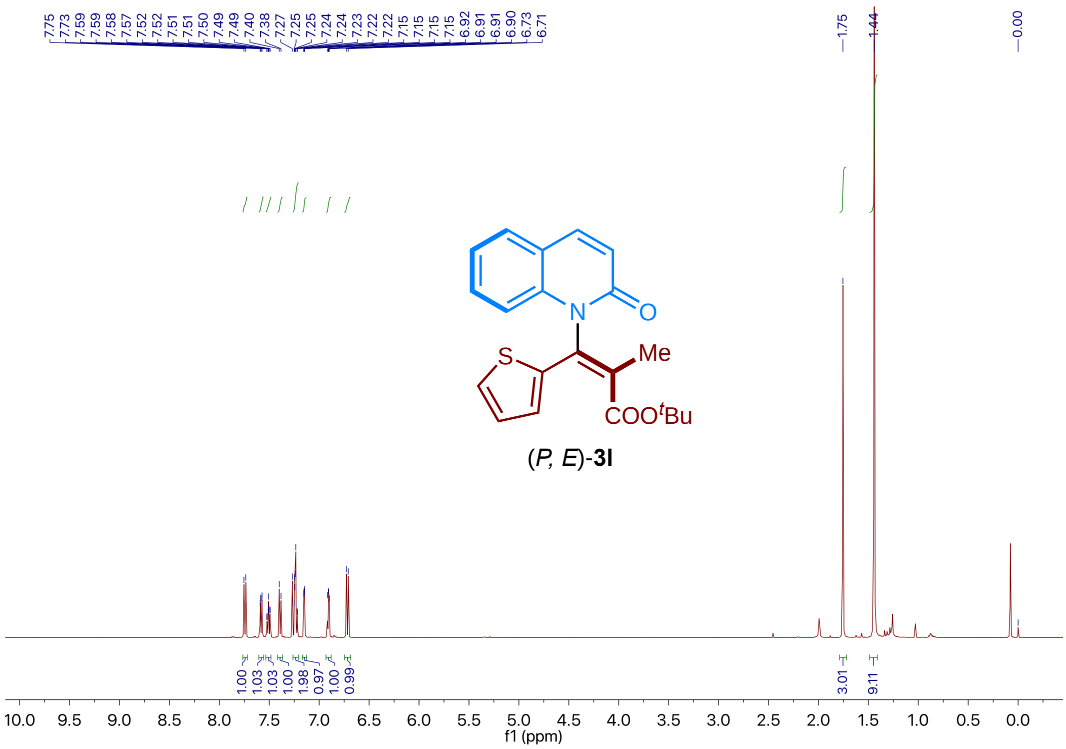

Supplementary Fig. 63. ^{1}H NMR spectrum of (*P*, *E*)-3l

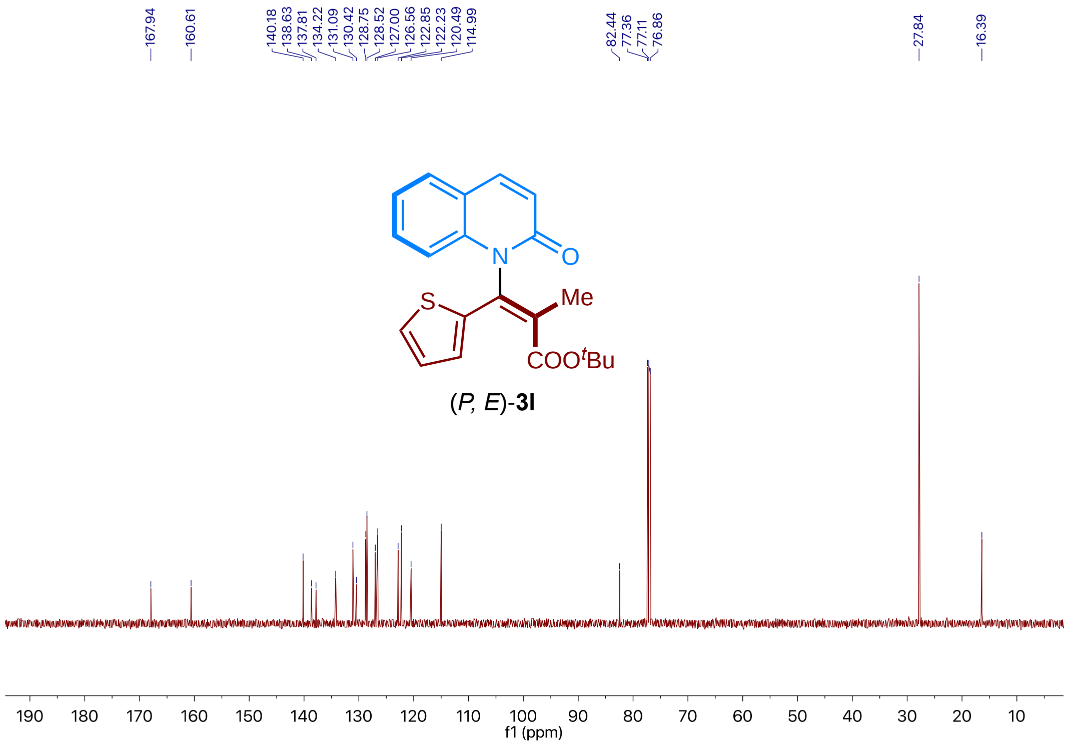

Supplementary Fig. 64. ^{13}C NMR spectrum of (*P*, *E*)-3l.

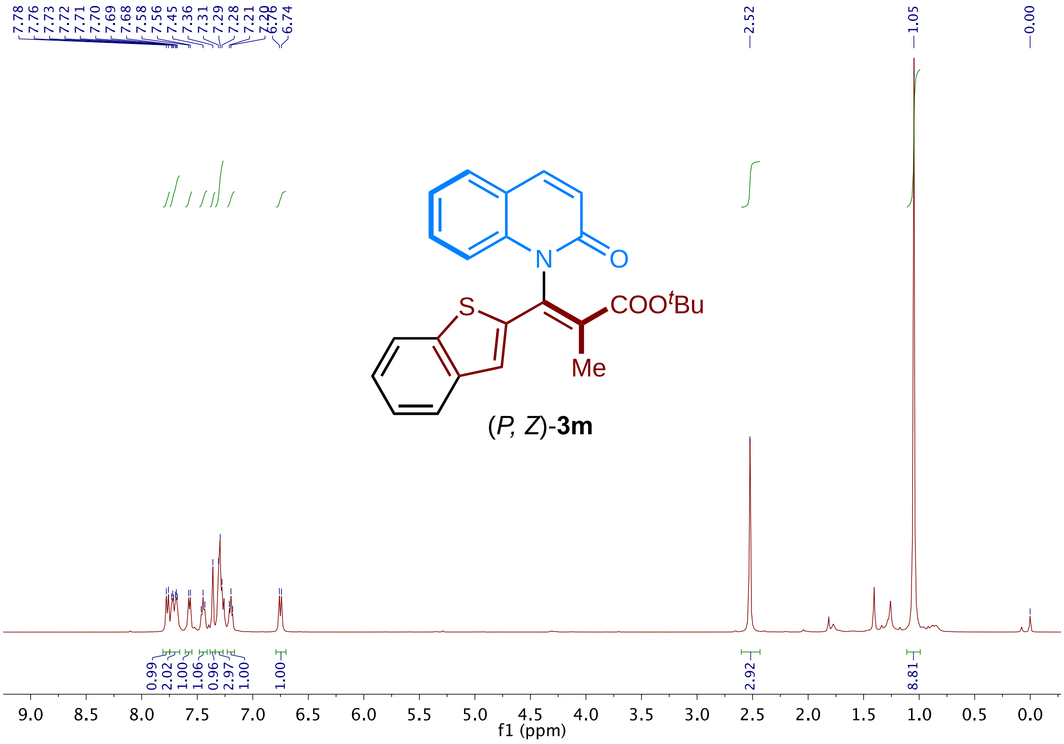

Supplementary Fig. 65. ^{1}**H NMR spectrum of (*P*, *Z*)-3m**

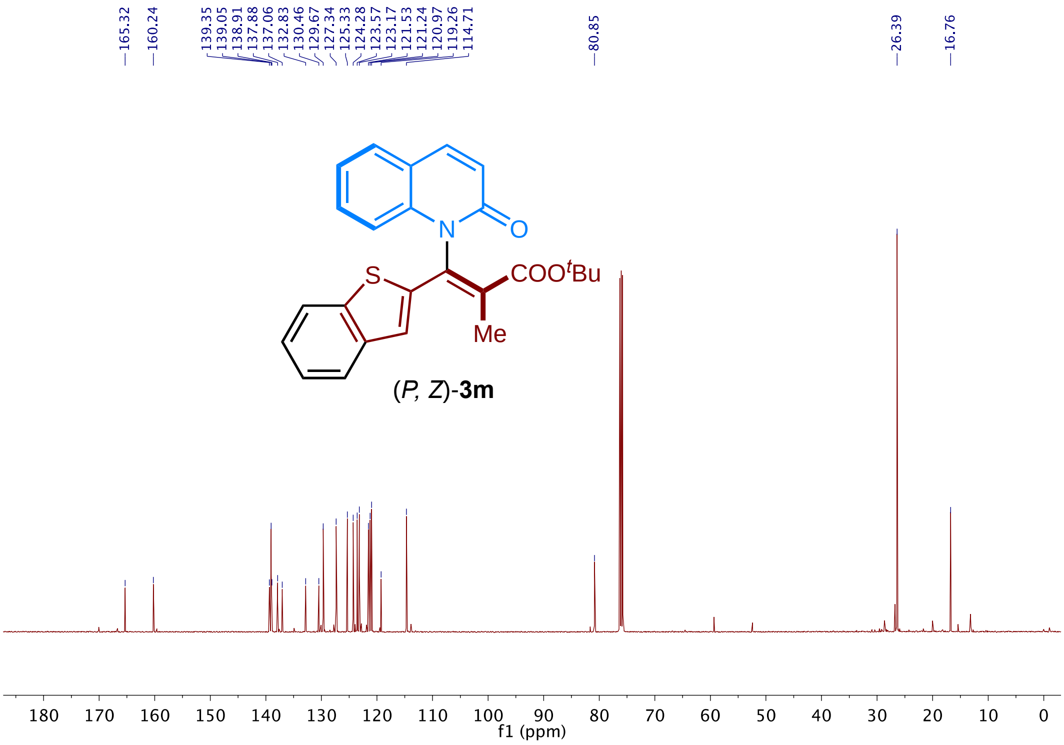

Supplementary Fig. 66. ^{13}**C NMR spectrum of (*P*, *Z*)-3m.**

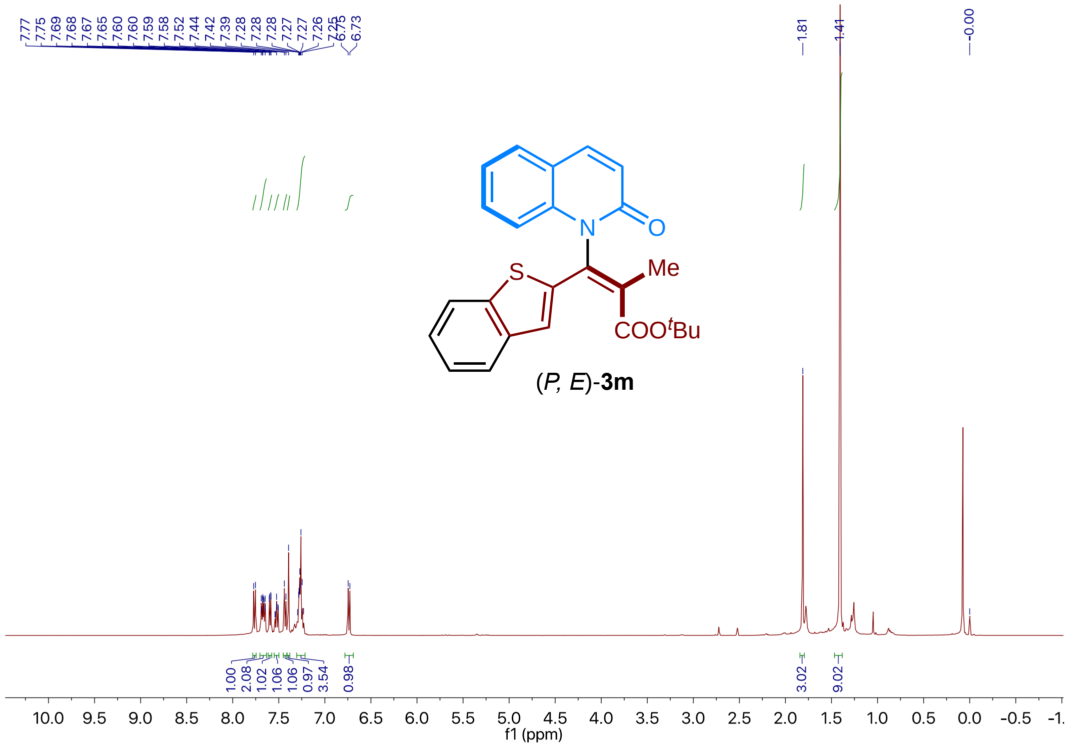

Supplementary Fig. 67. ¹H NMR spectrum of (*P*, *E*)-3m

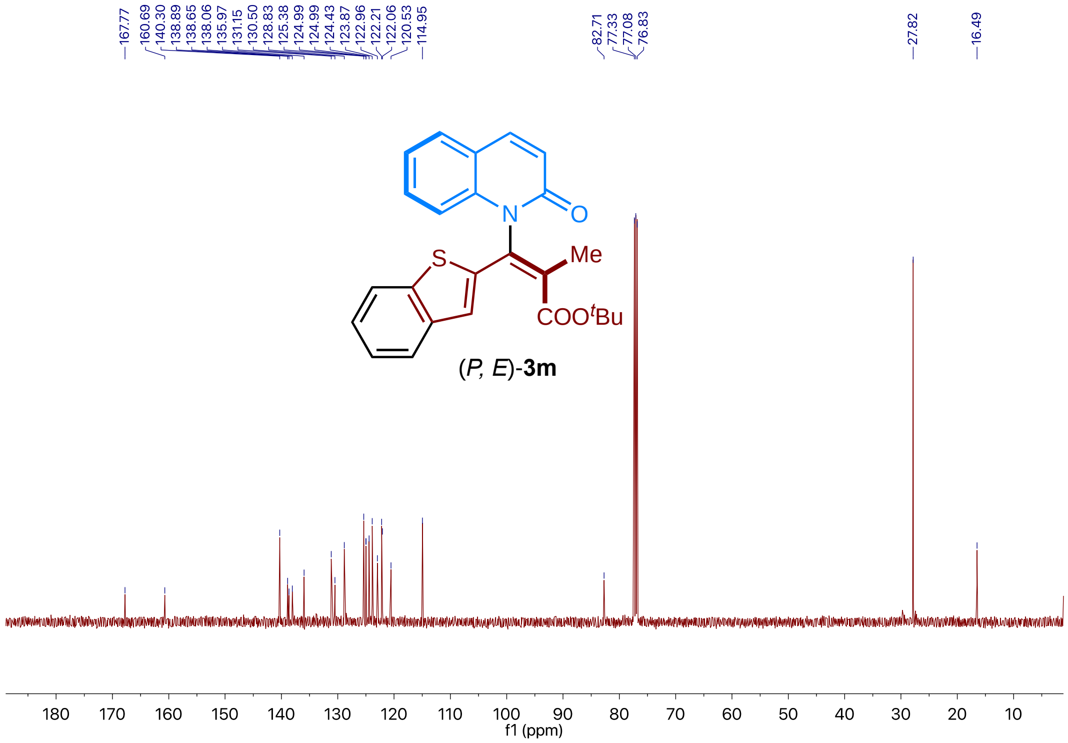

Supplementary Fig. 68. ¹³C NMR spectrum of (*P*, *E*)-3m.

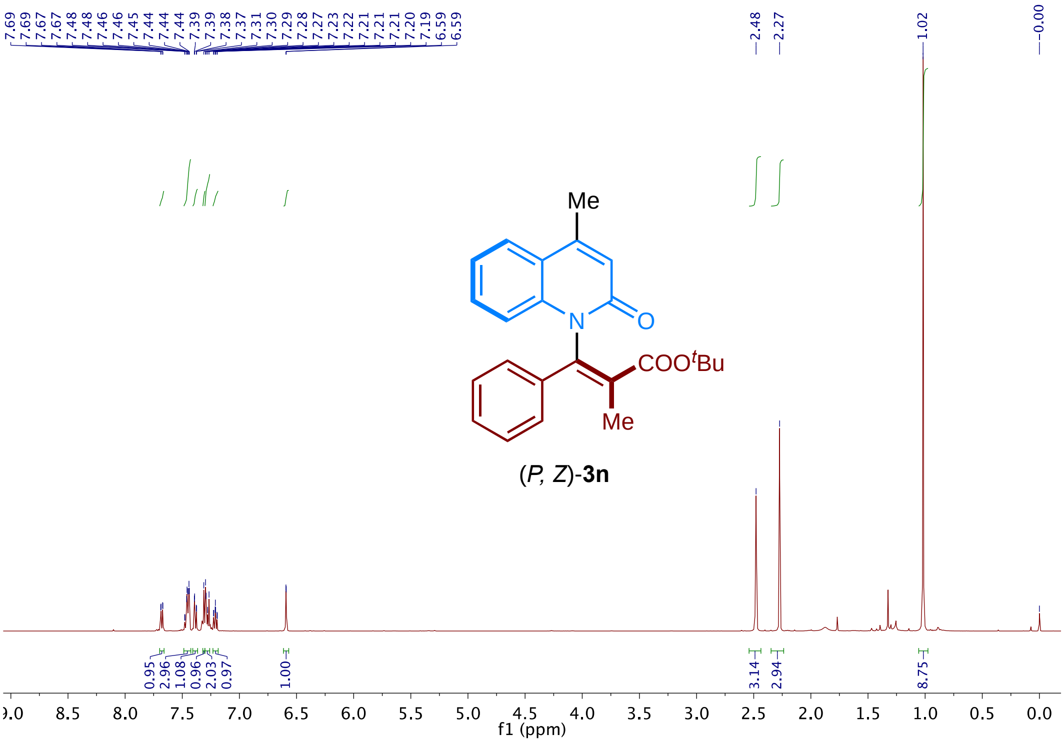

Supplementary Fig. 69. ¹H NMR spectrum of (*P*, *Z*)-3n

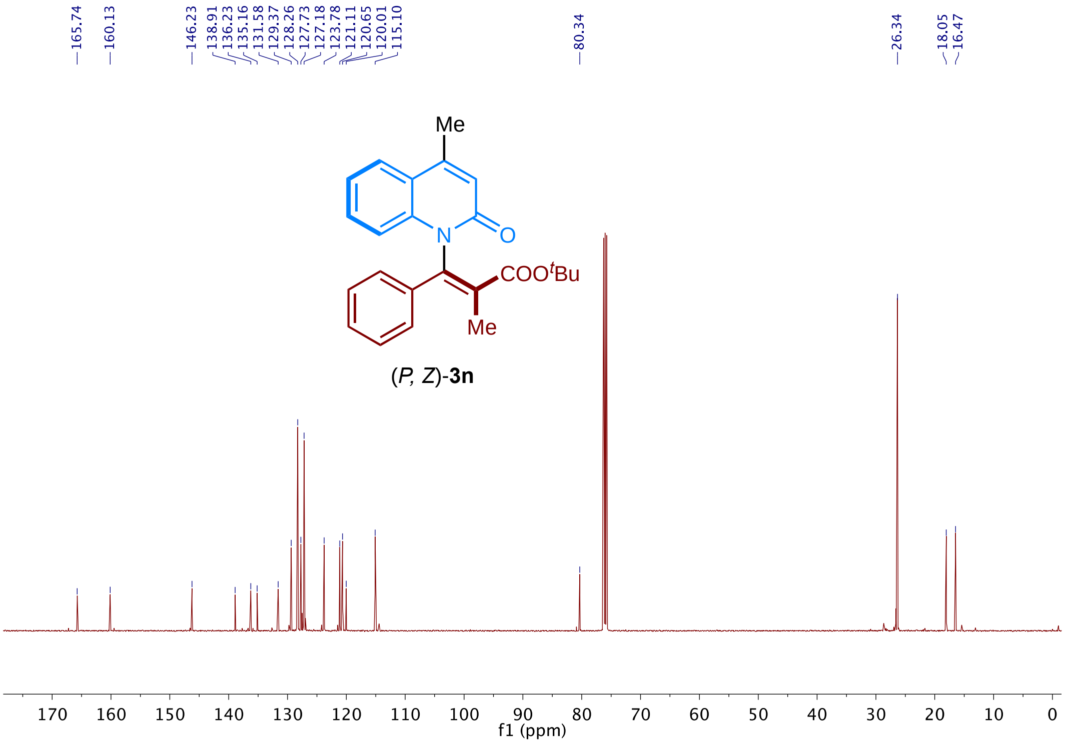

Supplementary Fig. 70. ¹³C NMR spectrum of (*P*, *Z*)-3n

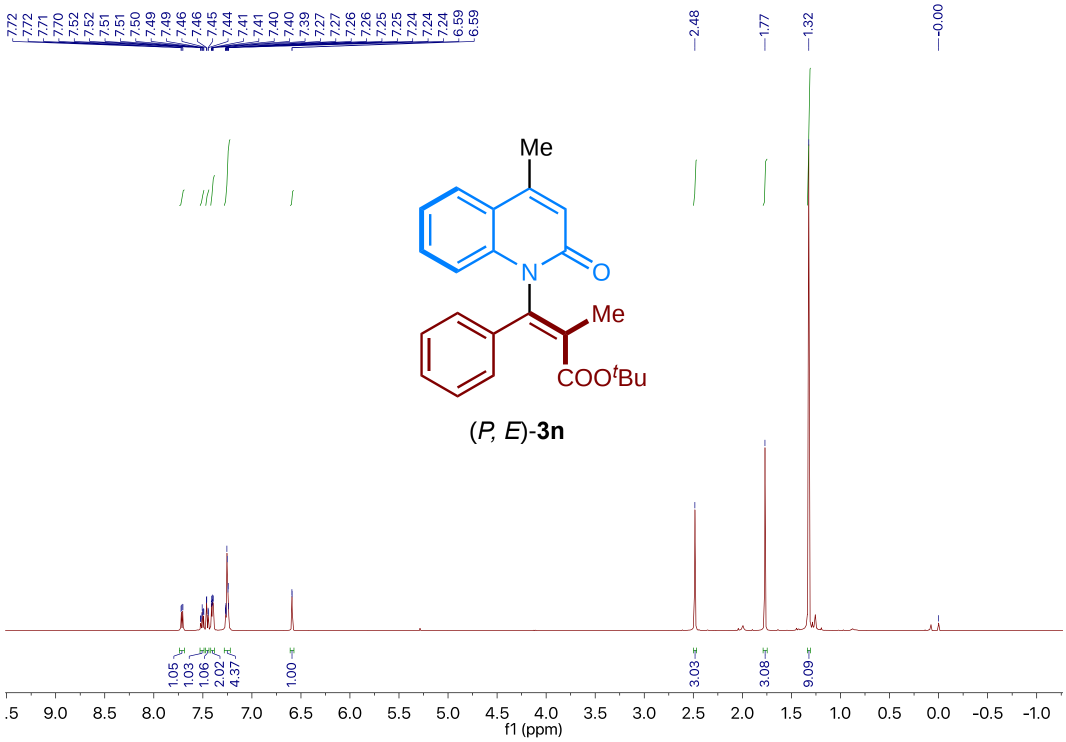

Supplementary Fig. 71. ^{1}H NMR spectrum of (*P*, *E*)-3n

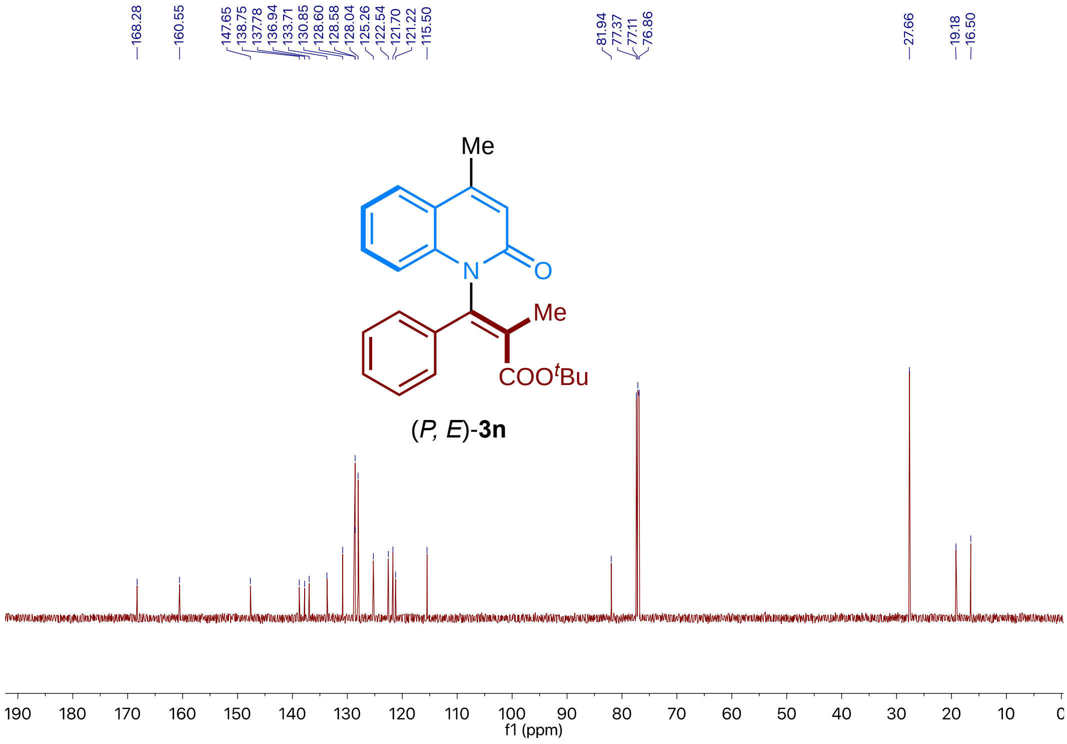

Supplementary Fig. 72. ^{13}C NMR spectrum of (*P*, *E*)-3n

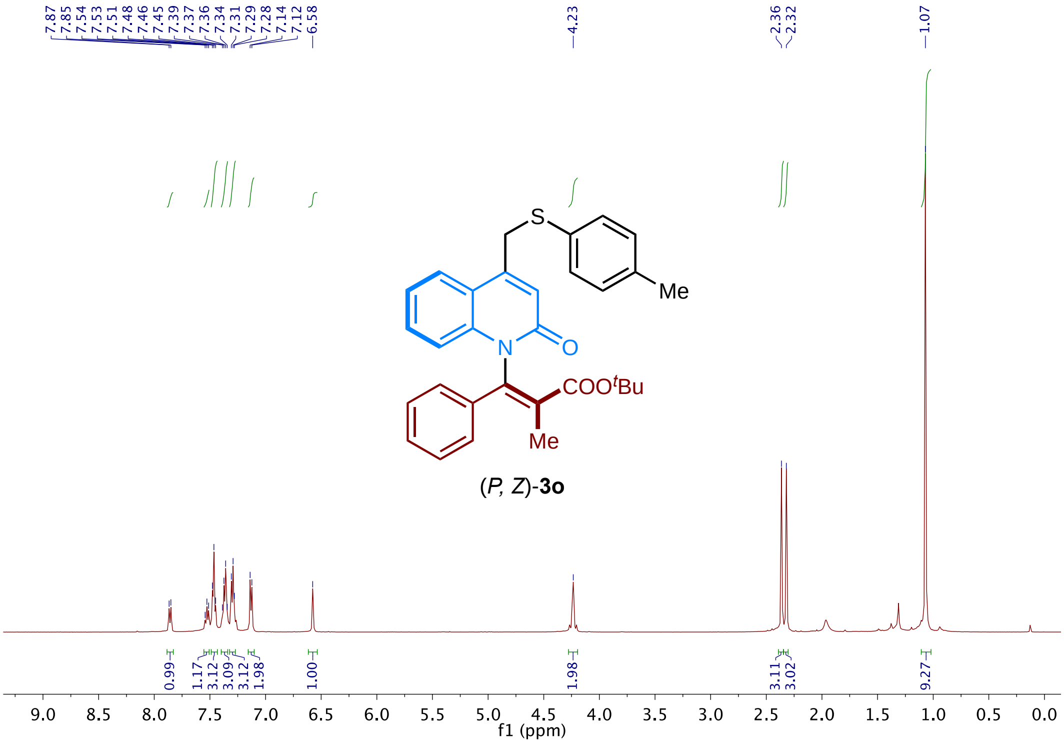

Supplementary Fig. 73. ^{1}H NMR spectrum of (*P*, *Z*)-3o

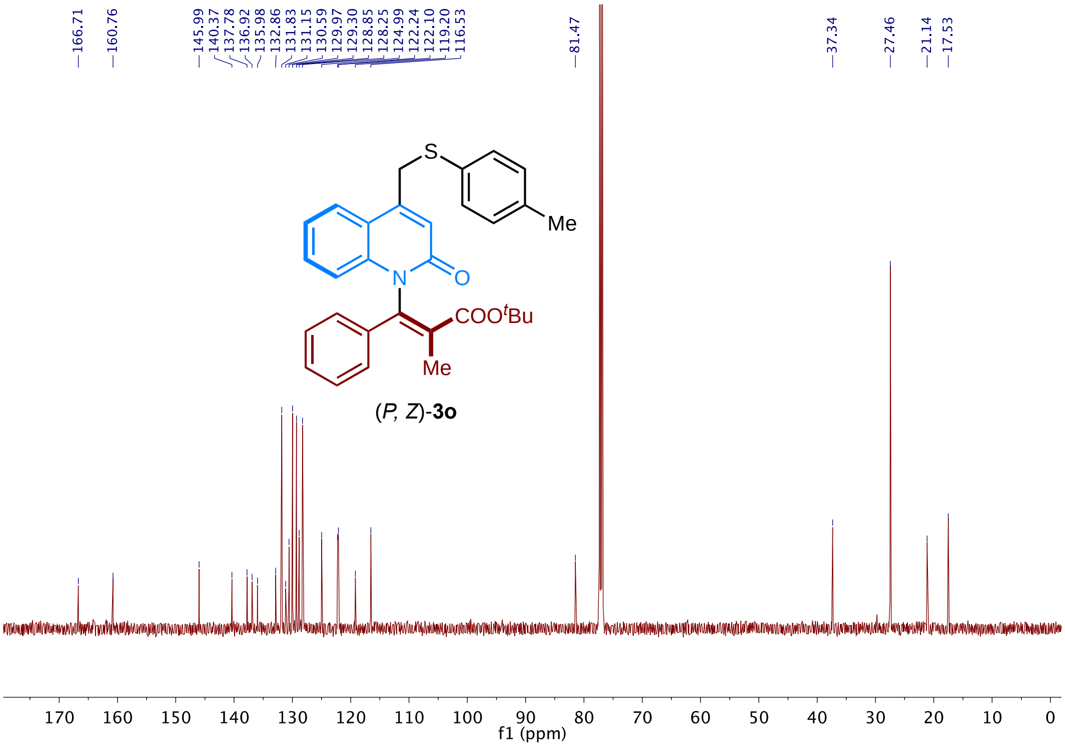

Supplementary Fig. 74. ^{13}C NMR spectrum of (*P*, *Z*)-3o

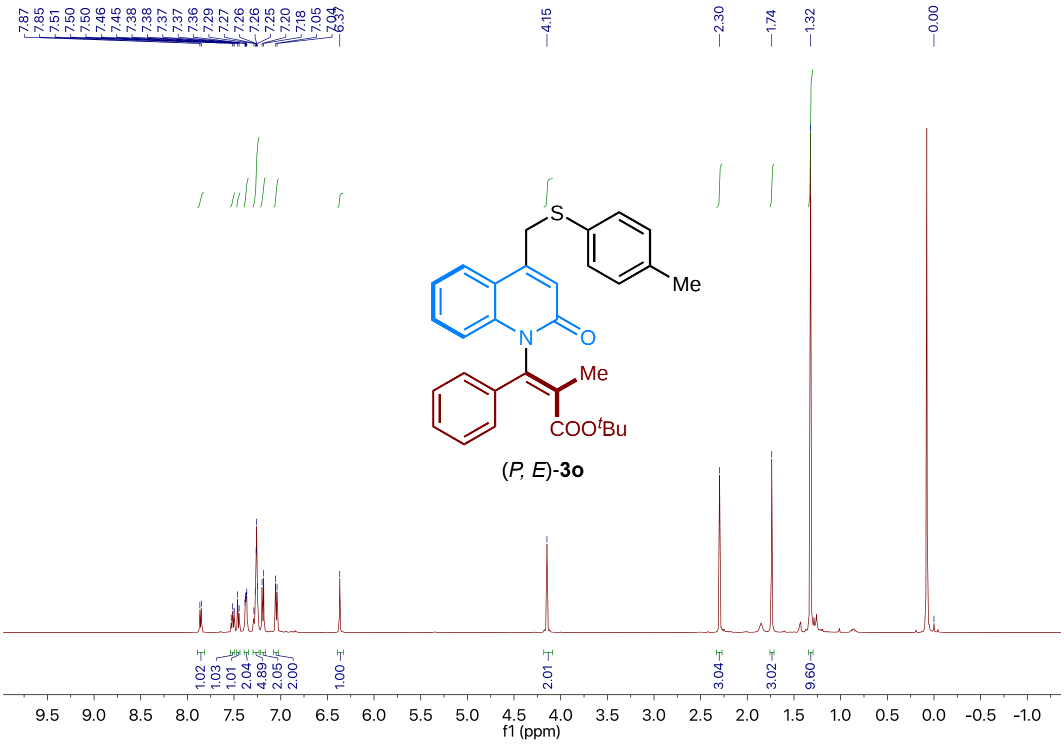

Supplementary Fig. 75. ^{1}**H NMR spectrum of (*P*, *E*)-3o**

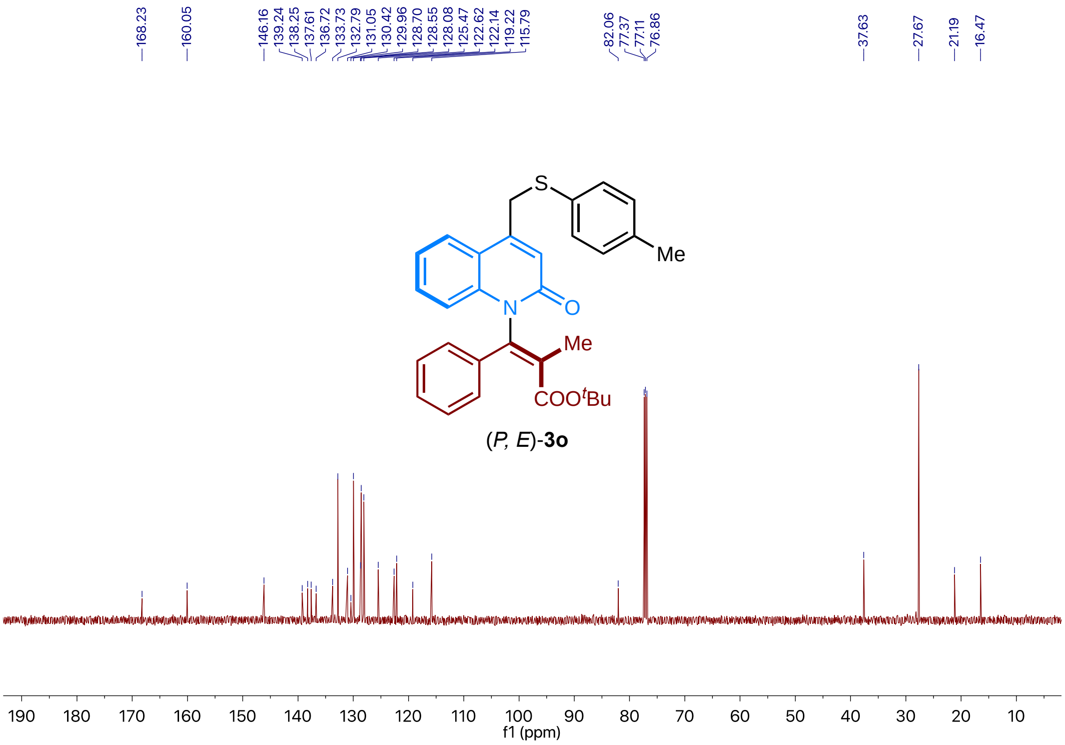

Supplementary Fig. 76. ^{13}**C NMR spectrum of (*P*, *E*)-3n**

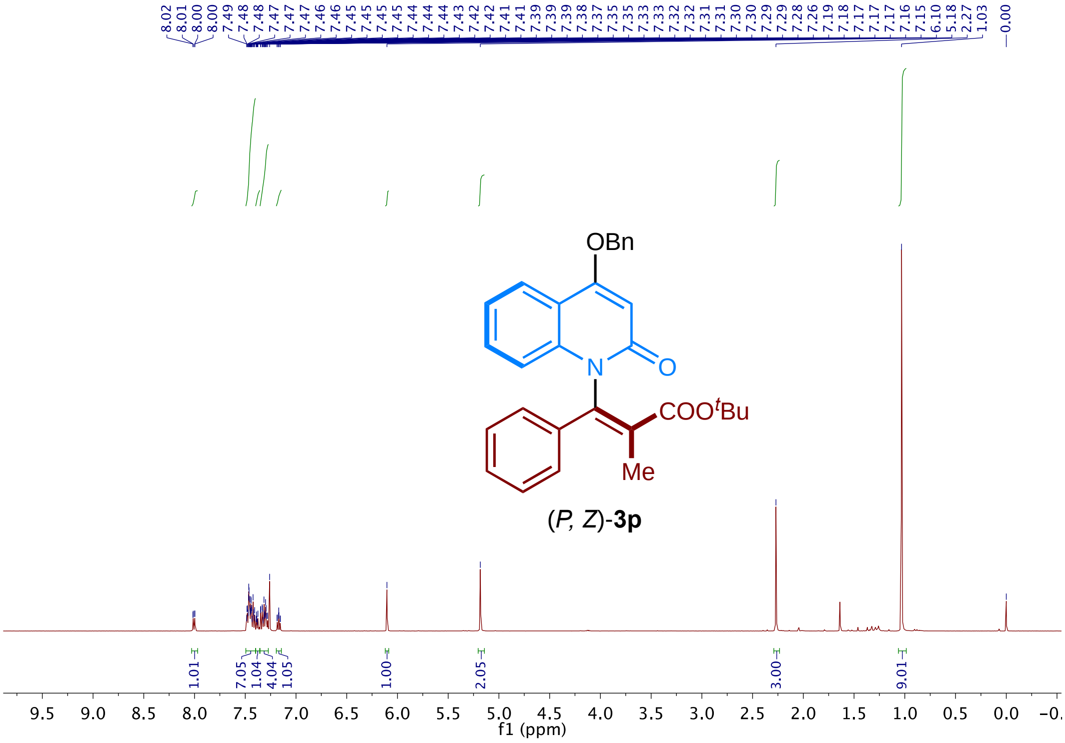

Supplementary Fig. 77. ^{1}H NMR spectrum of (*P*, *Z*)-3p

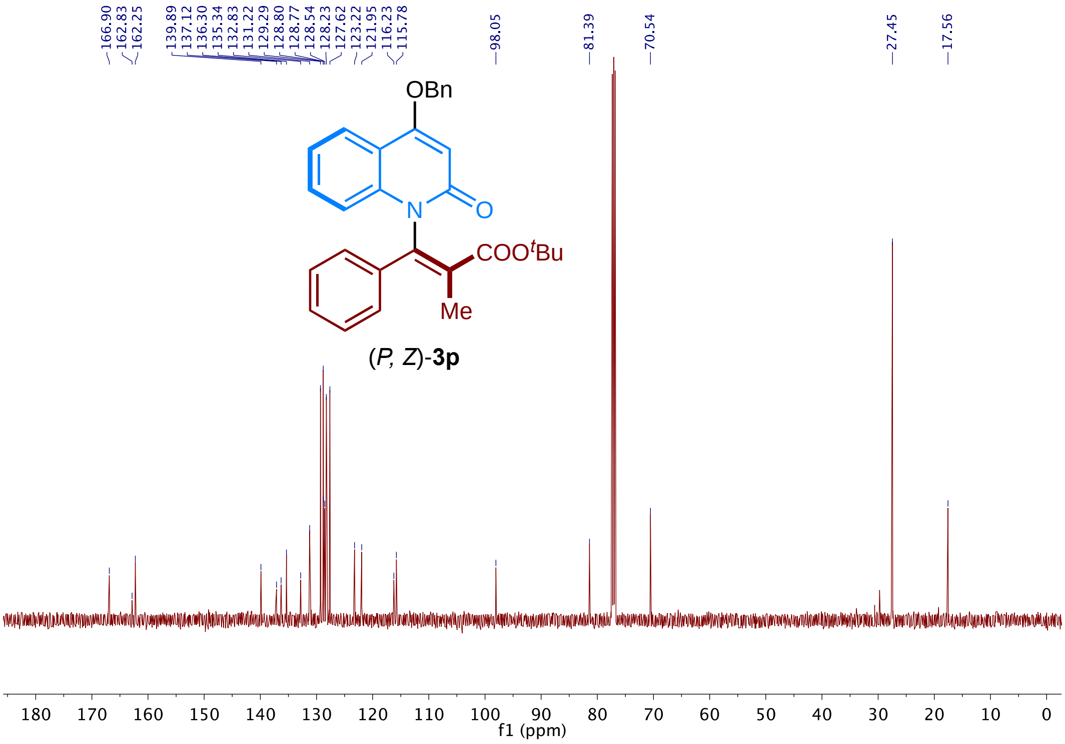

Supplementary Fig. 78. ^{13}C NMR spectrum of (*P*, *Z*)-3p

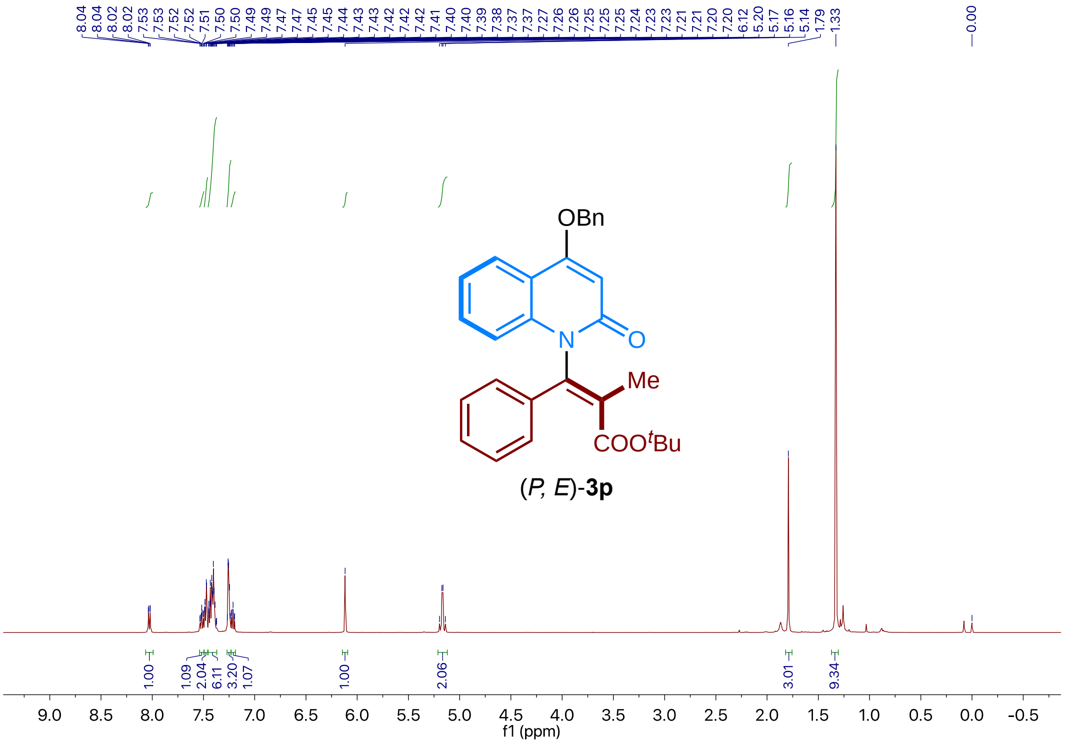

Supplementary Fig. 79. ^{1}**H NMR spectrum of (*P*, *E*)-3p**

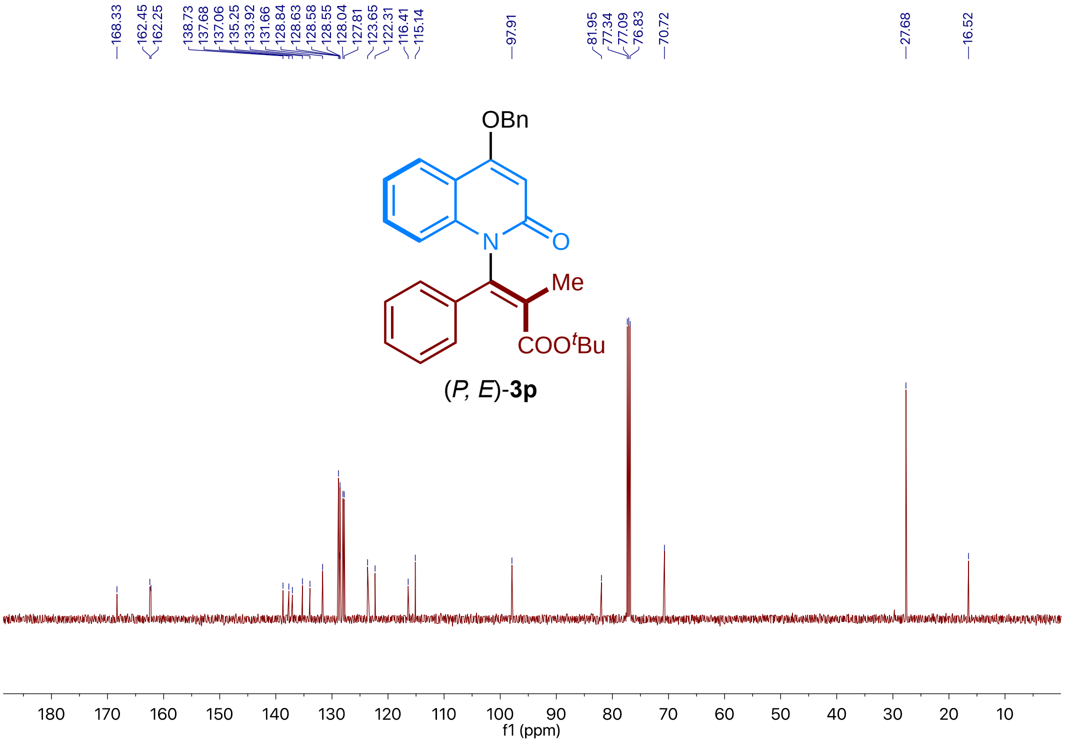

Supplementary Fig. 80. ^{13}**C NMR spectrum of (*P*, *E*)-3p**

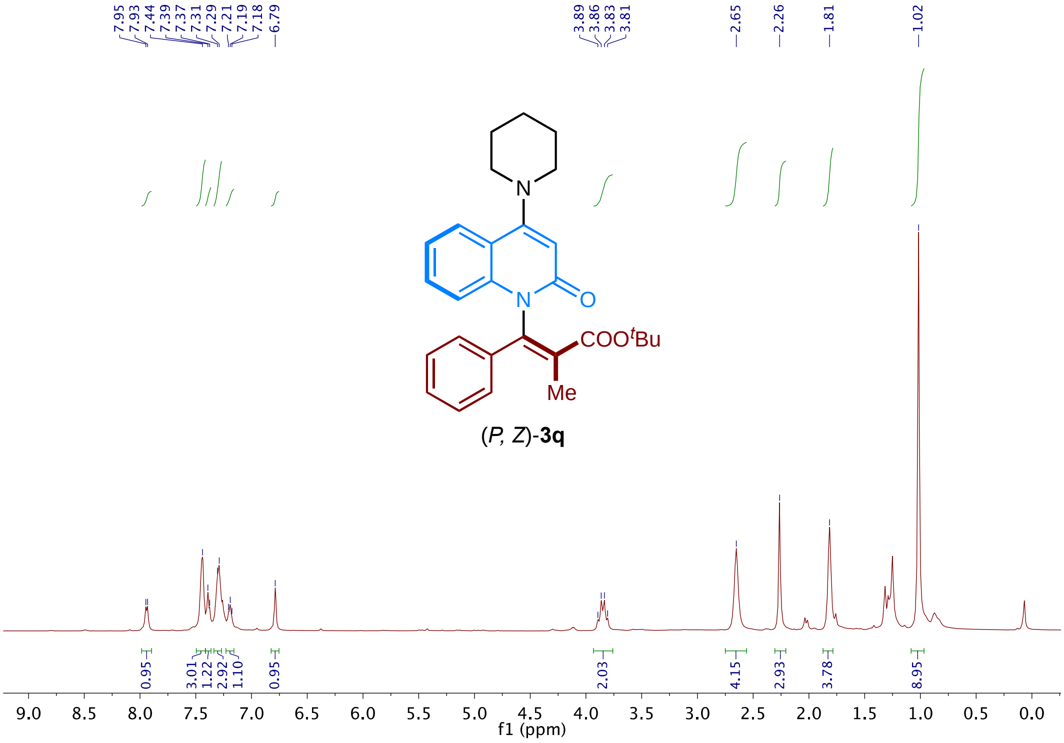

Supplementary Fig. 81. ^{1}**H NMR spectrum of (*P*, *Z*)-3q**

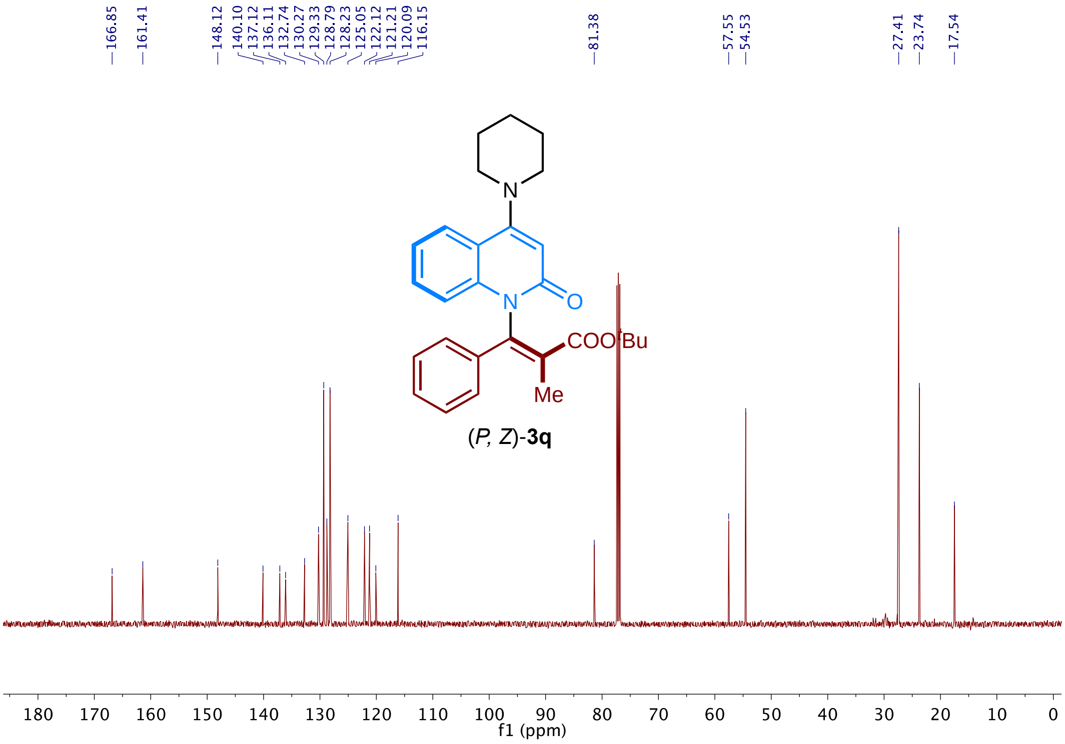

Supplementary Fig. 82. ^{13}**C NMR spectrum of (*P*, *Z*)-3q**

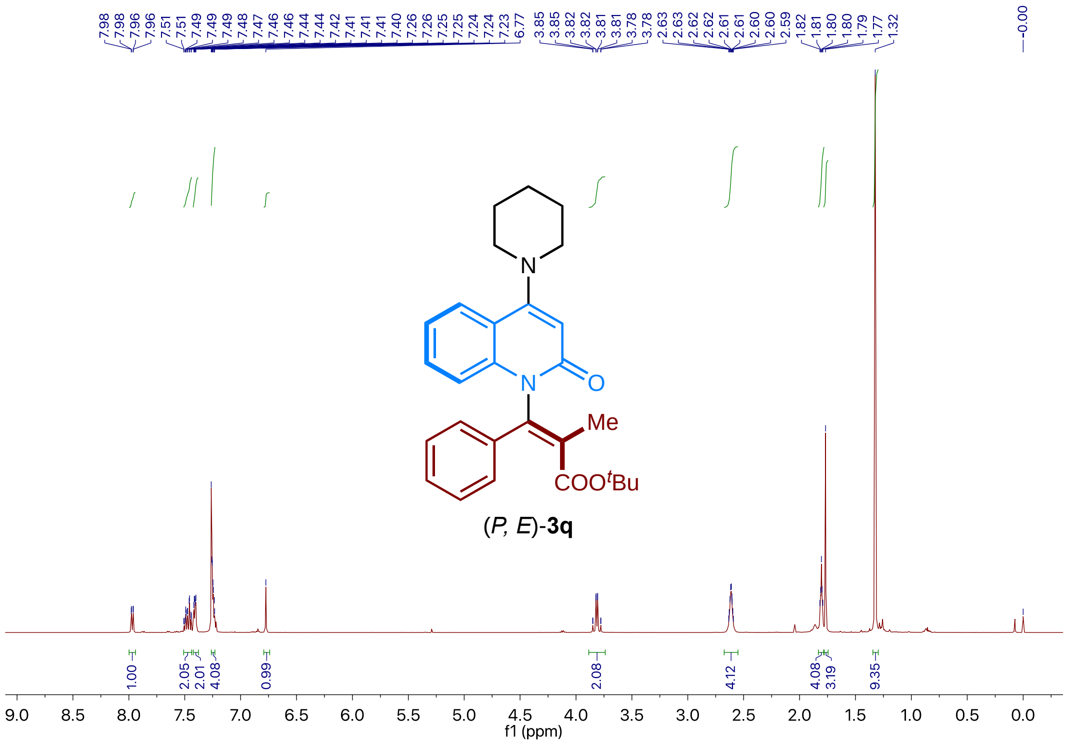

Supplementary Fig. 83. ^{1}H NMR spectrum of (*P*, *E*)-3q

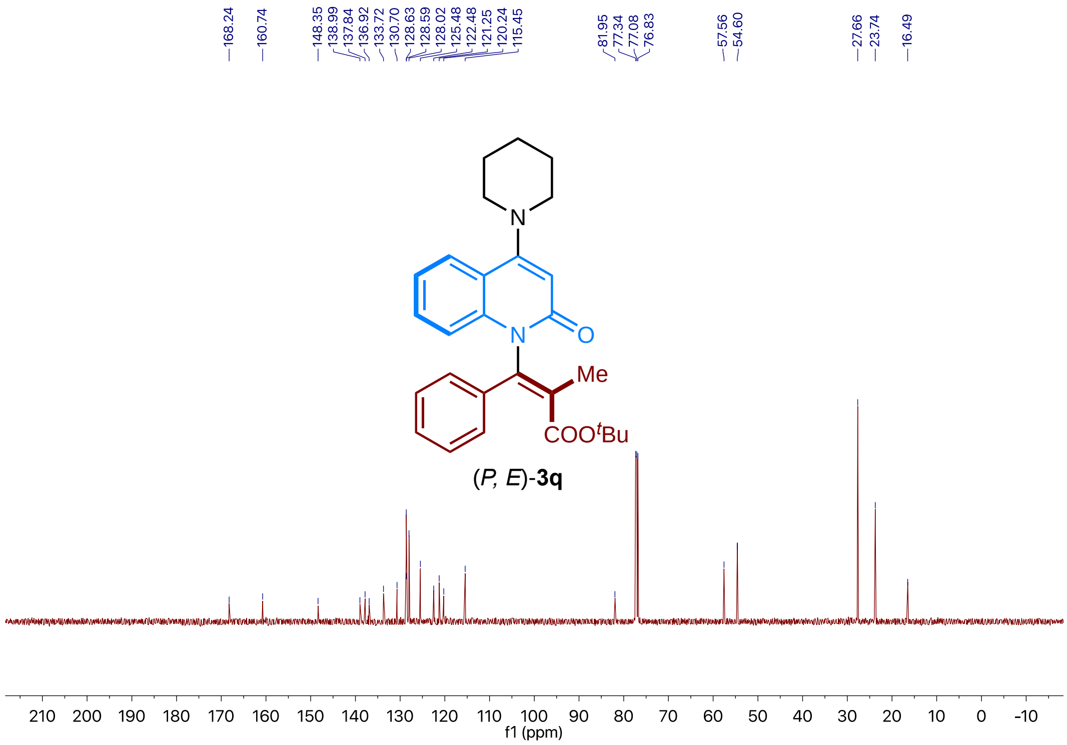

Supplementary Fig. 84. ^{13}C NMR spectrum of (*P*, *E*)-3q

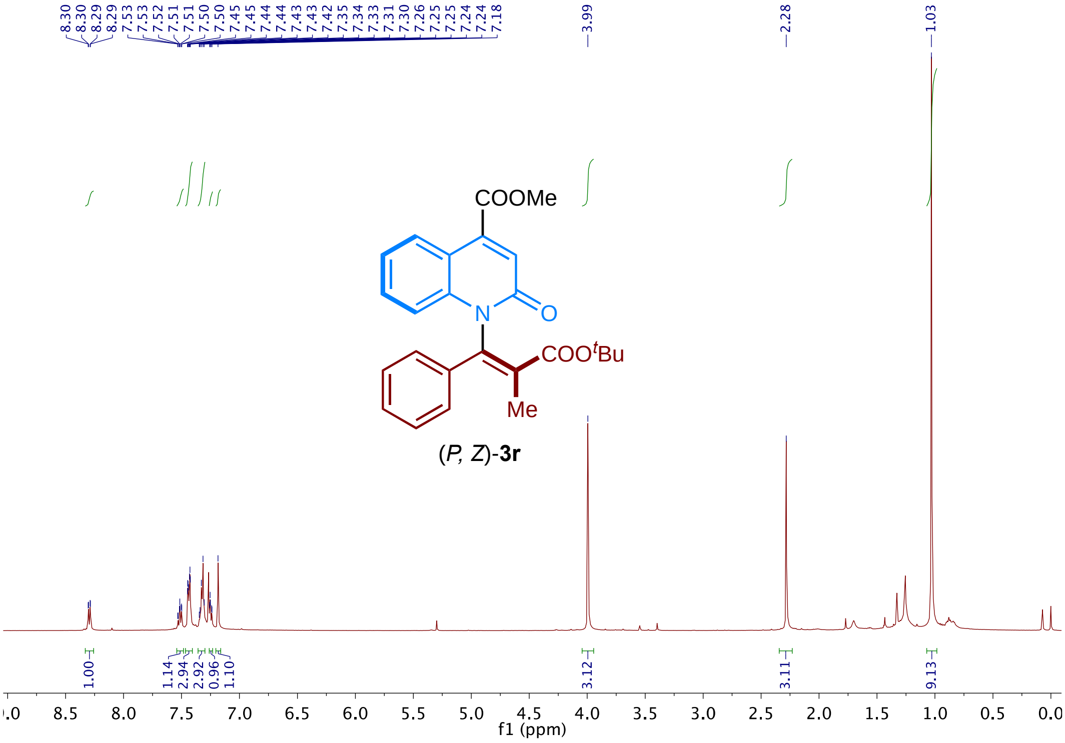

Supplementary Fig. 85. ^{1}H NMR spectrum of (*P*, *Z*)-3r

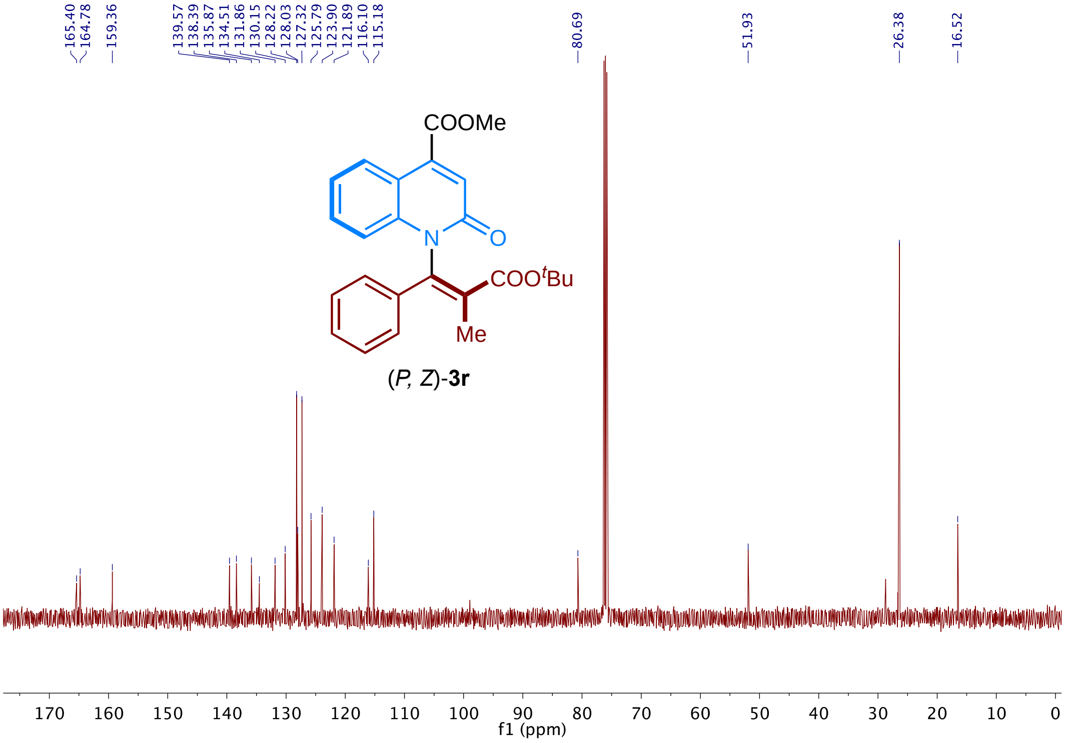

Supplementary Fig. 86. ^{13}C NMR spectrum of (*P*, *Z*)-3r

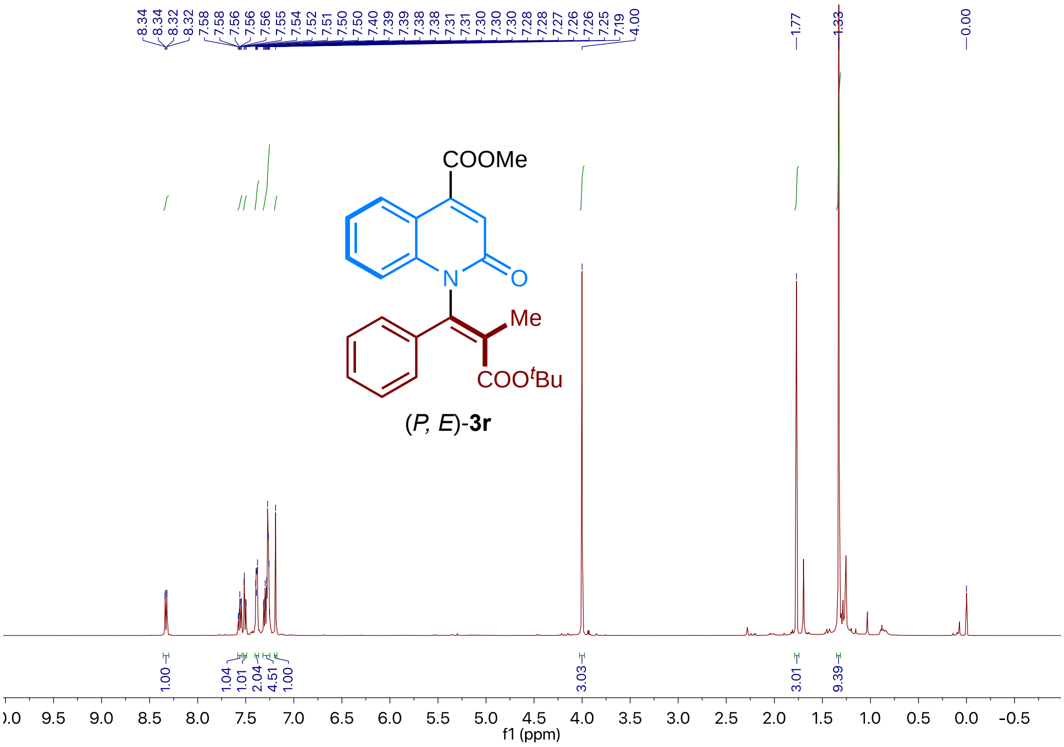

Supplementary Fig. 87. ¹H NMR spectrum of (*P*, *E*)-3r

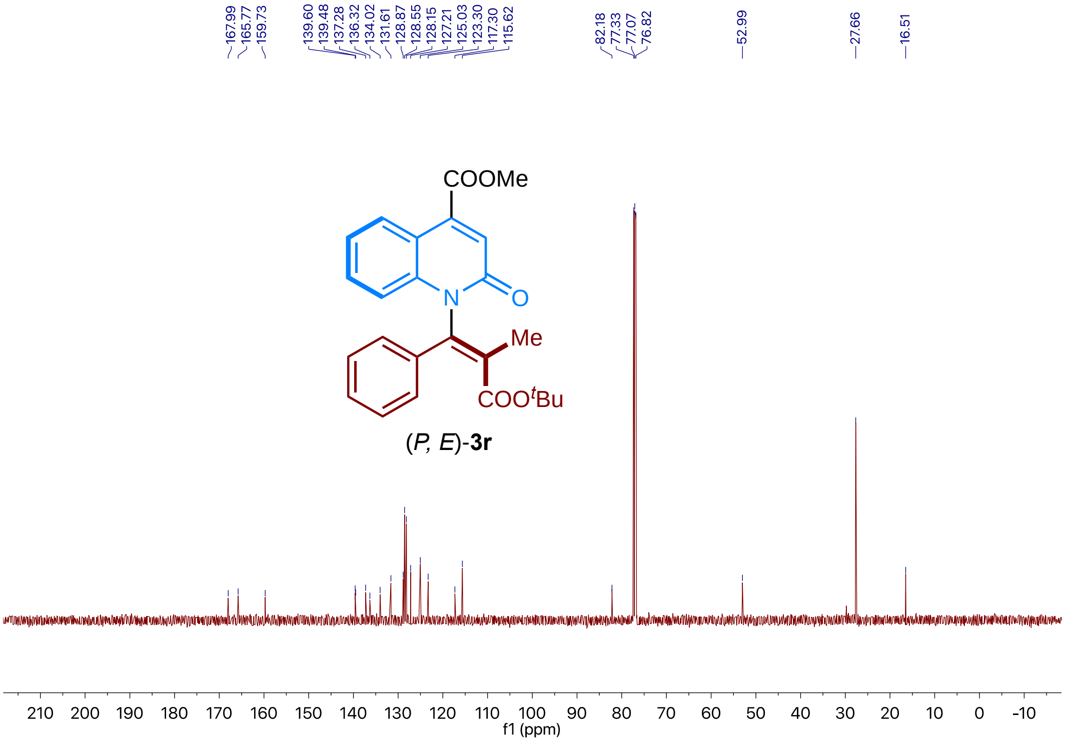

Supplementary Fig. 88. ¹³C NMR spectrum of (*P*, *E*)-3r

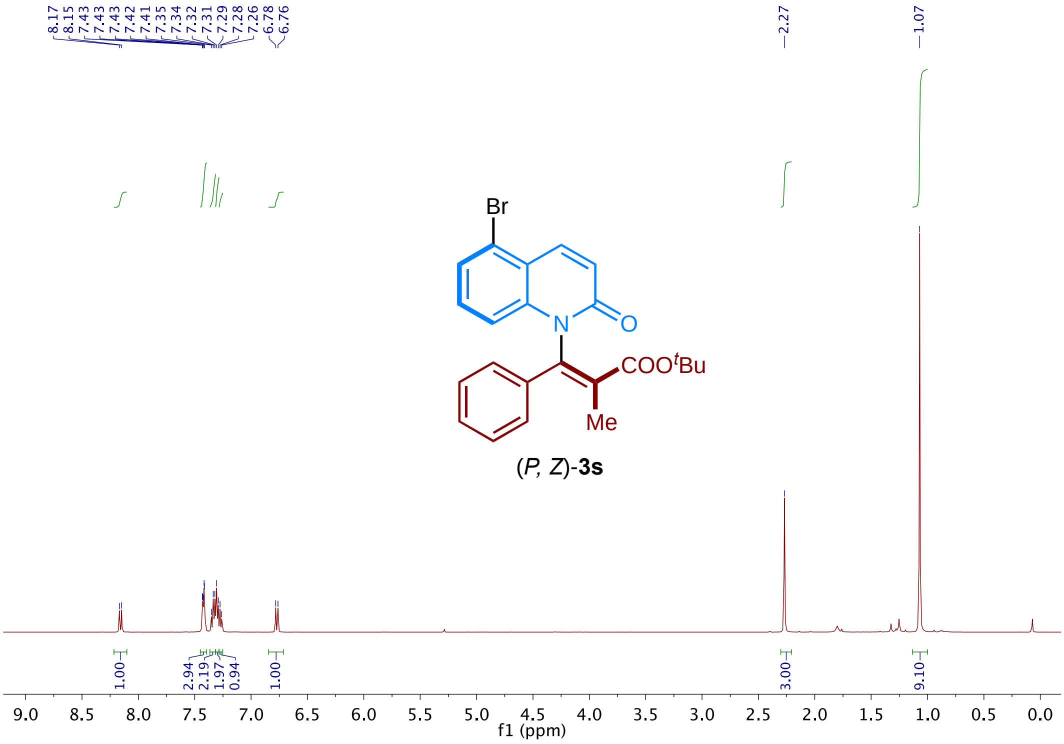

Supplementary Fig. 89. ^{1}**H NMR spectrum of (*P*, *Z*)-3s**

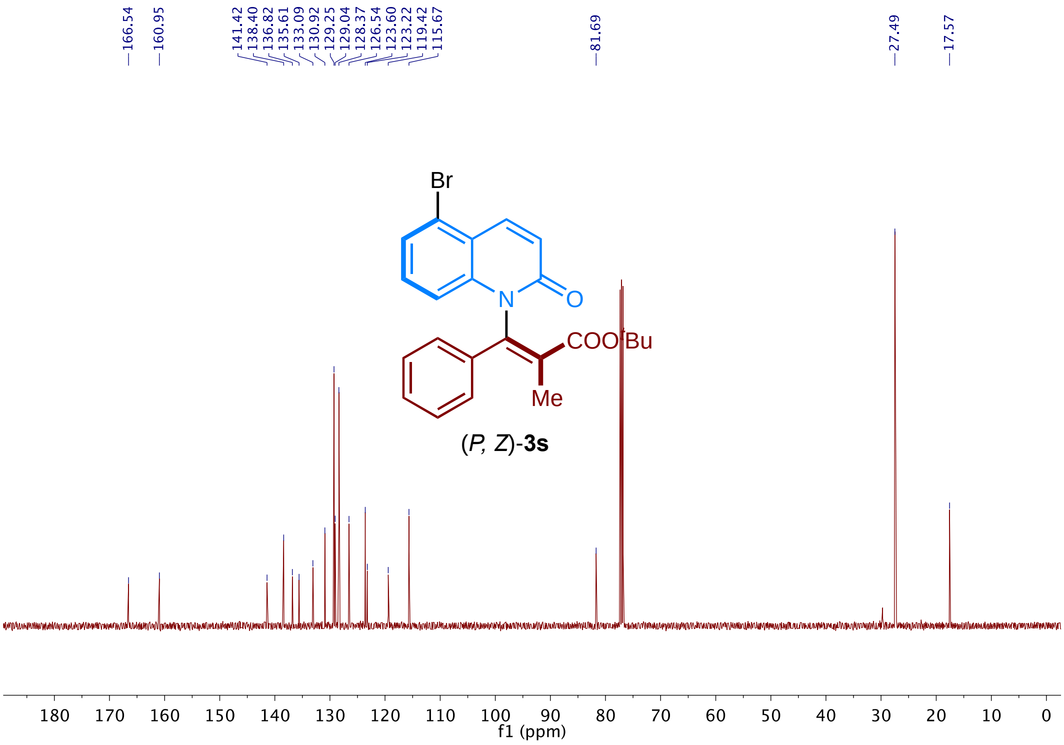

Supplementary Fig. 90. ^{13}**C NMR spectrum of (*P*, *Z*)-3s**

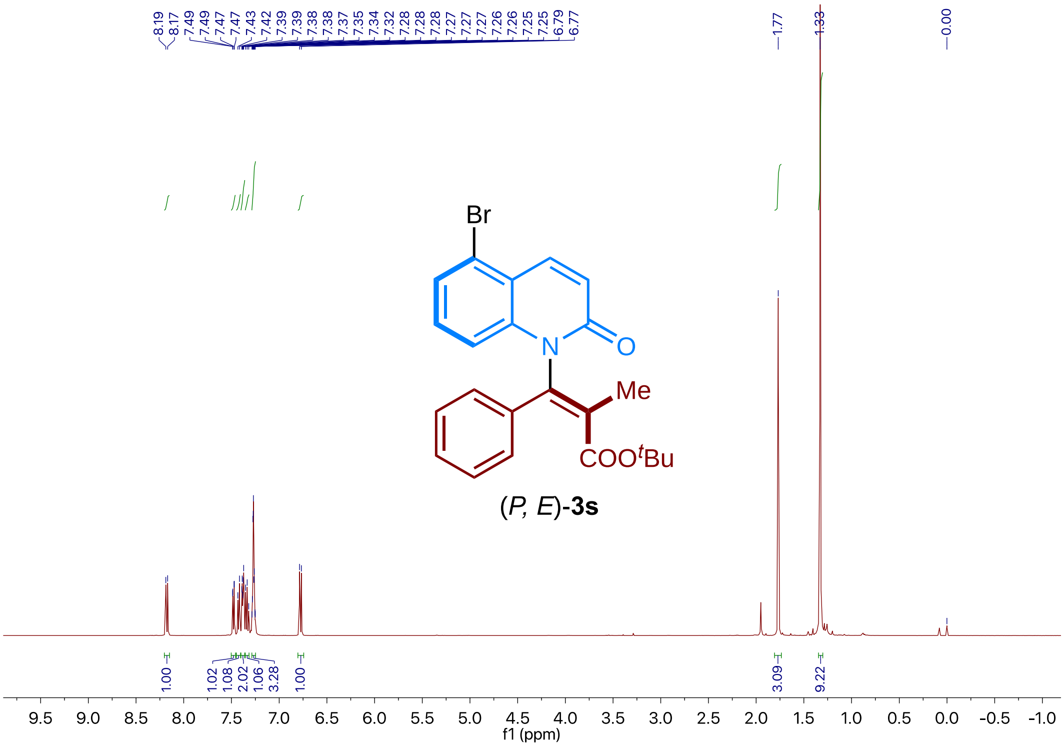

Supplementary Fig. 91. ^{1}H NMR spectrum of (*P*, *E*)-3s

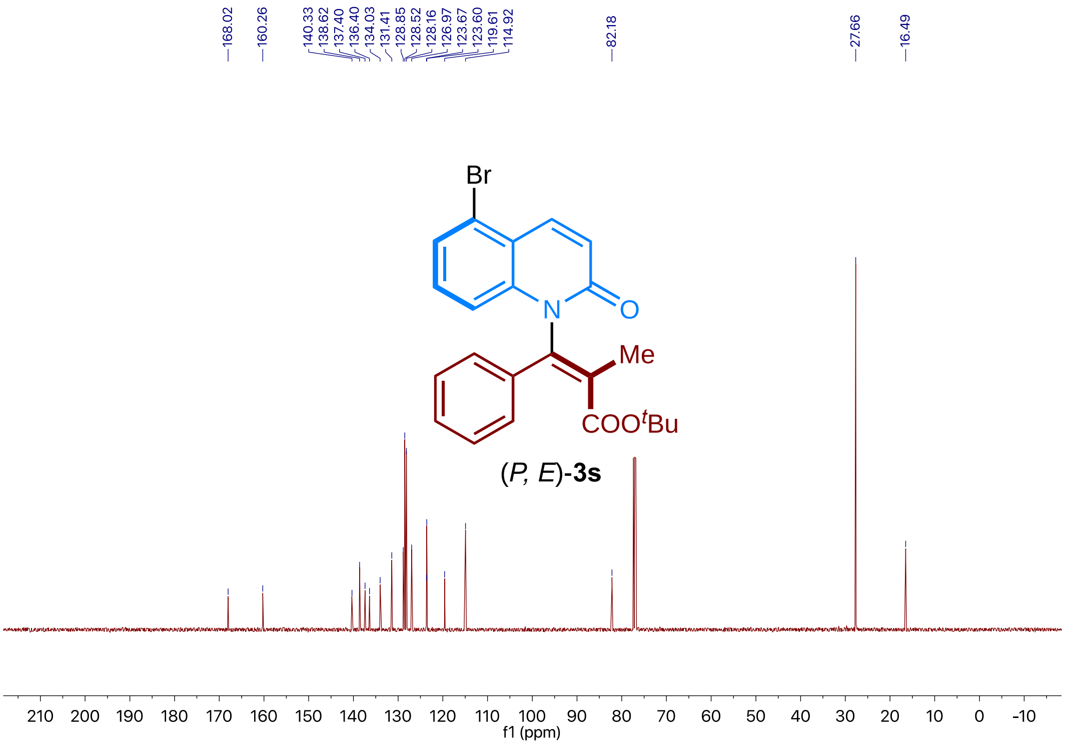

Supplementary Fig. 92. ^{13}C NMR spectrum of (*P*, *E*)-3s

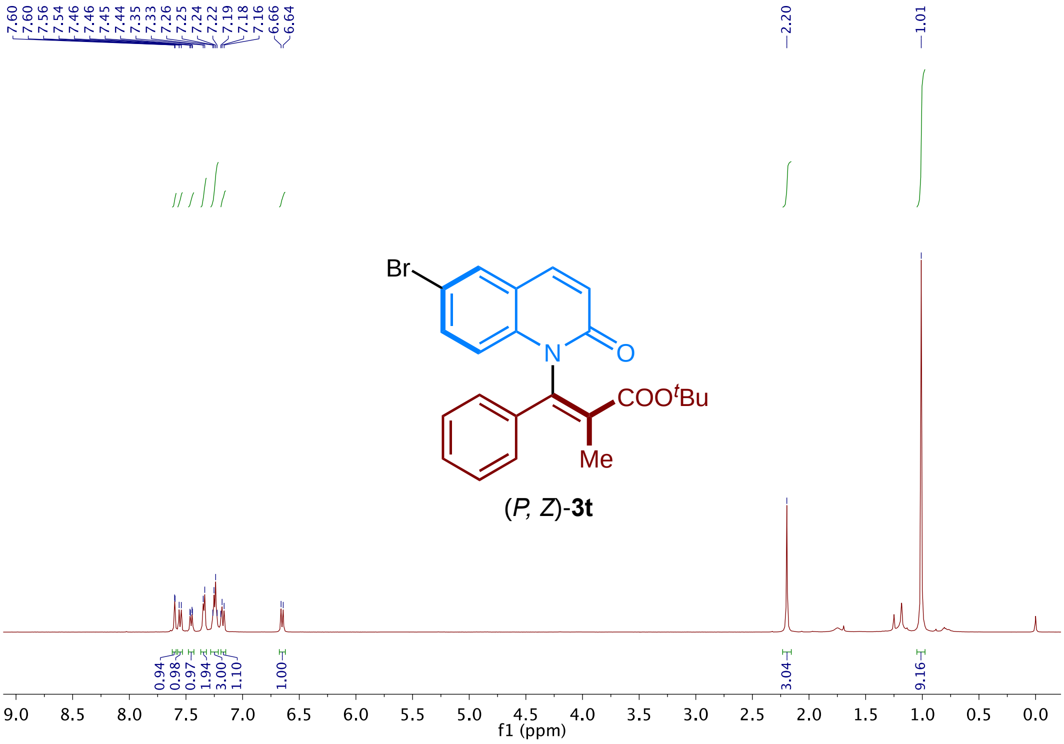

Supplementary Fig. 93. ¹H NMR spectrum of (*P*, *Z*)-3t

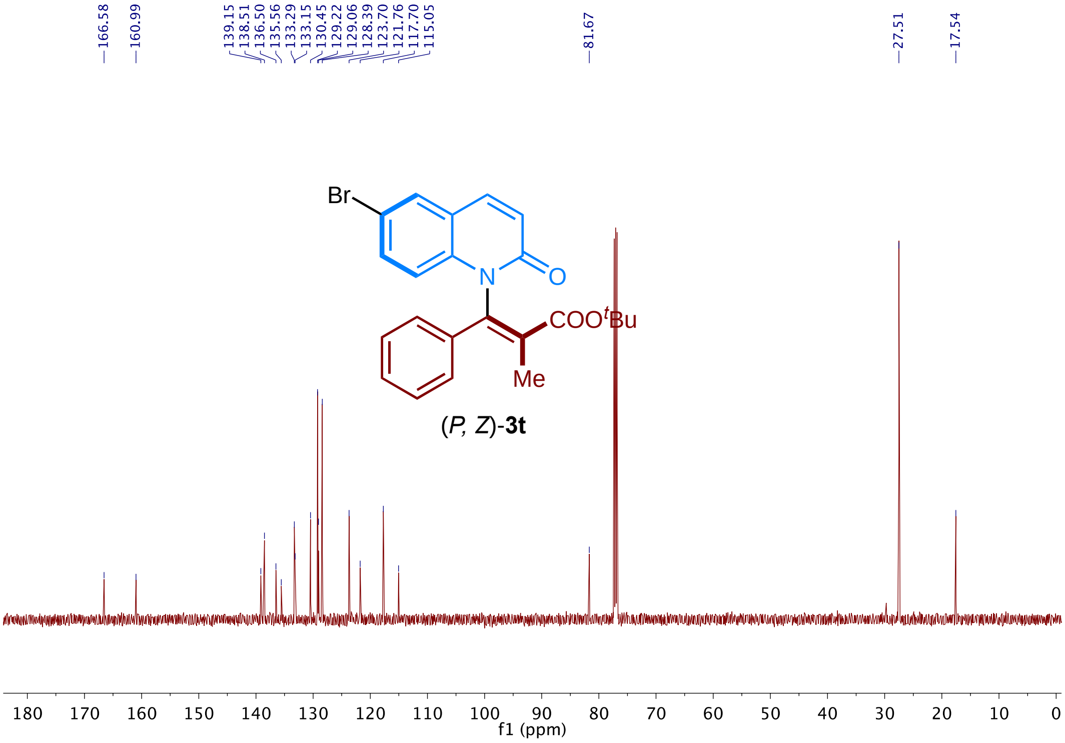

Supplementary Fig. 94. ¹³C NMR spectrum of (*P*, *Z*)-3t

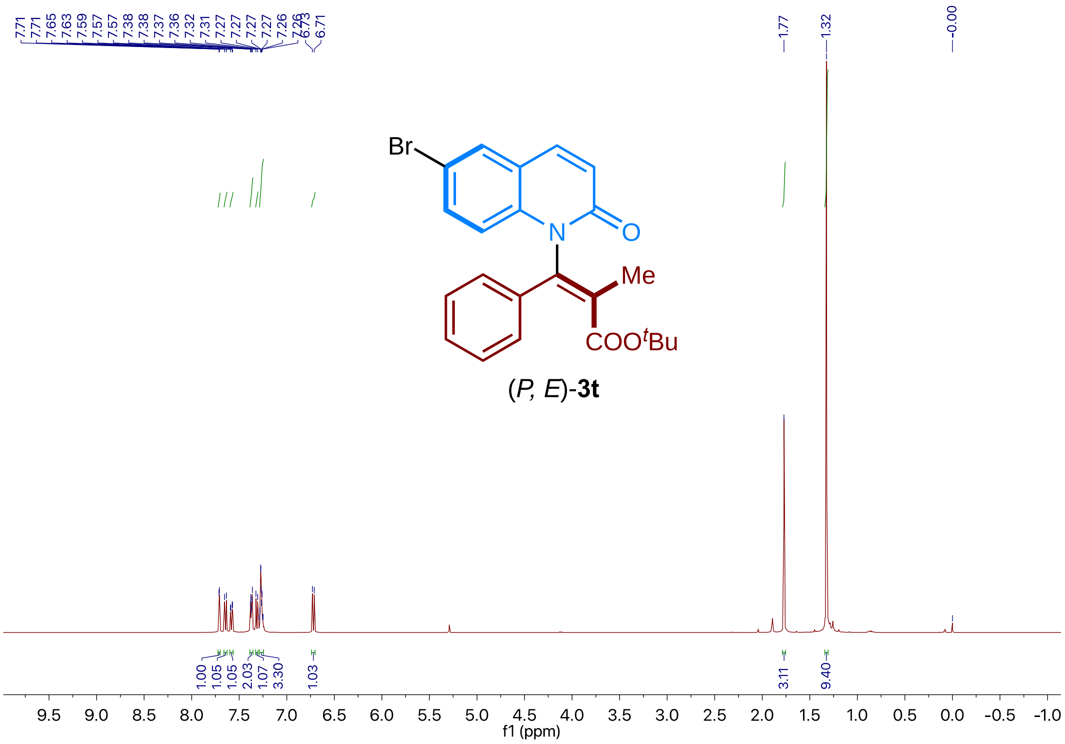

Supplementary Fig. 95. ^{1}H NMR spectrum of (*P*, *E*)-3t

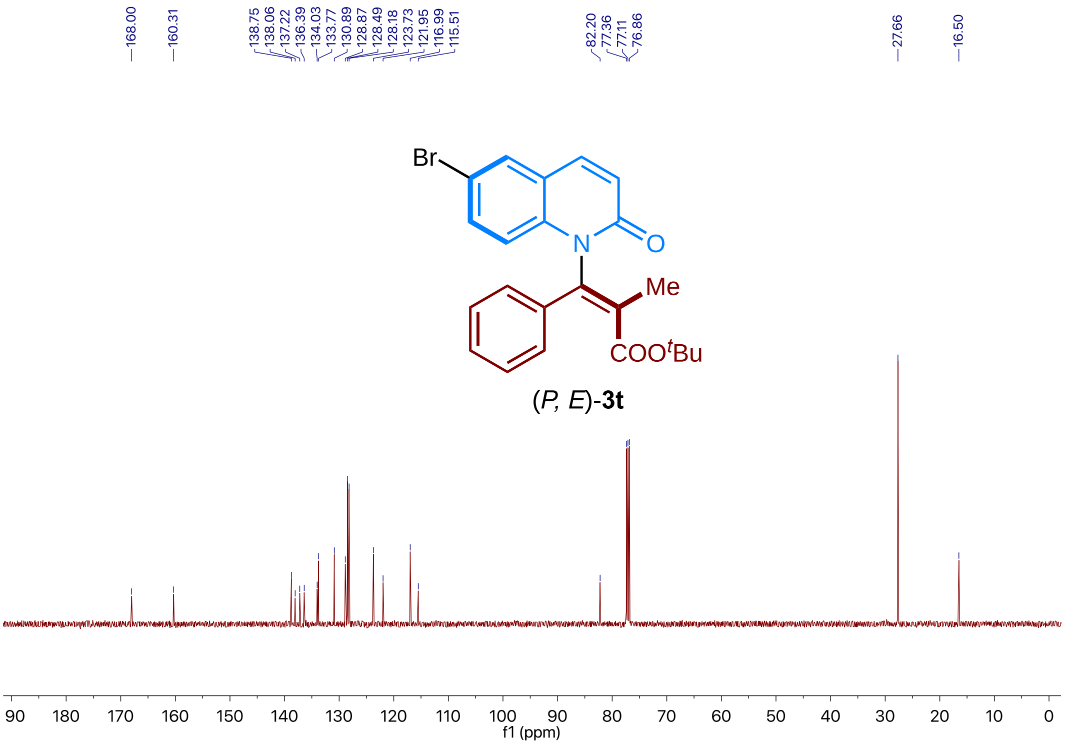

Supplementary Fig. 96. ^{13}C NMR spectrum of (*P*, *E*)-3t

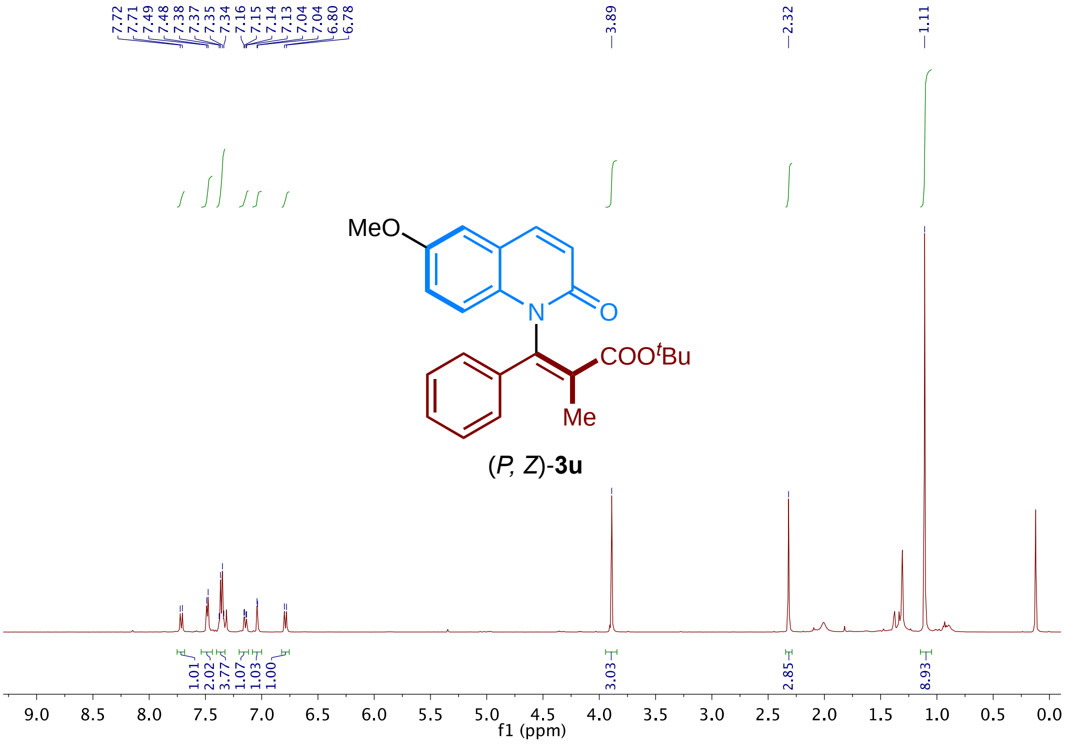

Supplementary Fig. 97. ^{1}H NMR spectrum of (*P*, *Z*)-3u

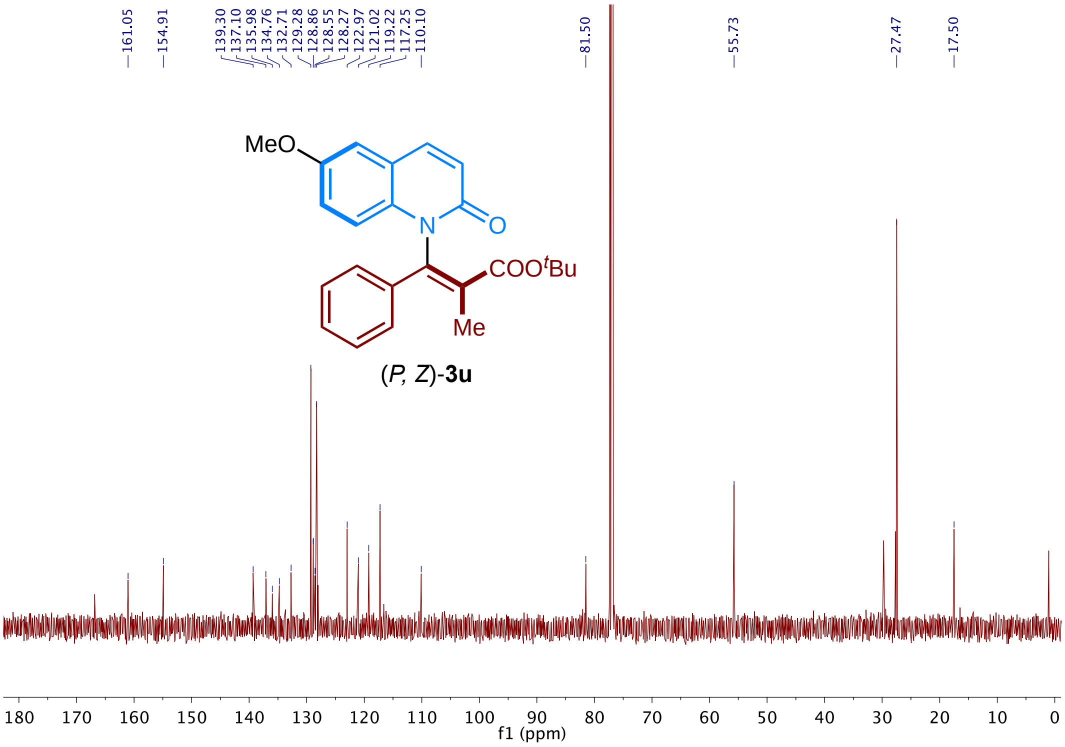

Supplementary Fig. 98. ^{13}C NMR spectrum of (*P*, *Z*)-3u

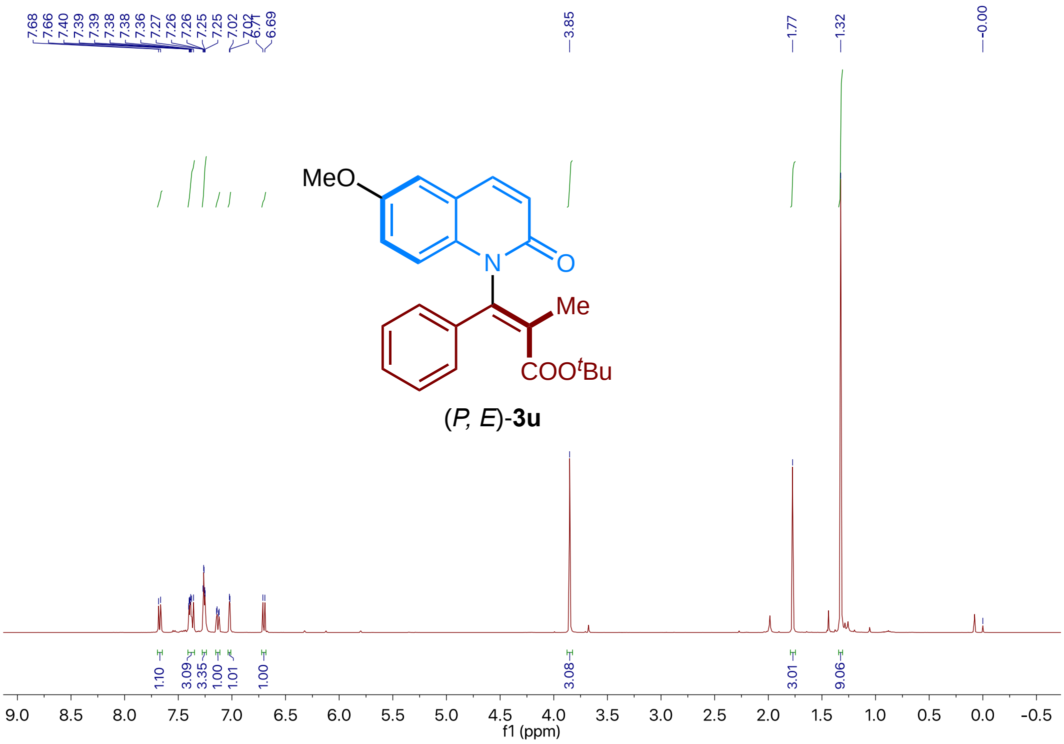

Supplementary Fig. 99. ^{1}**H NMR spectrum of (*P*, *E*)-3u**

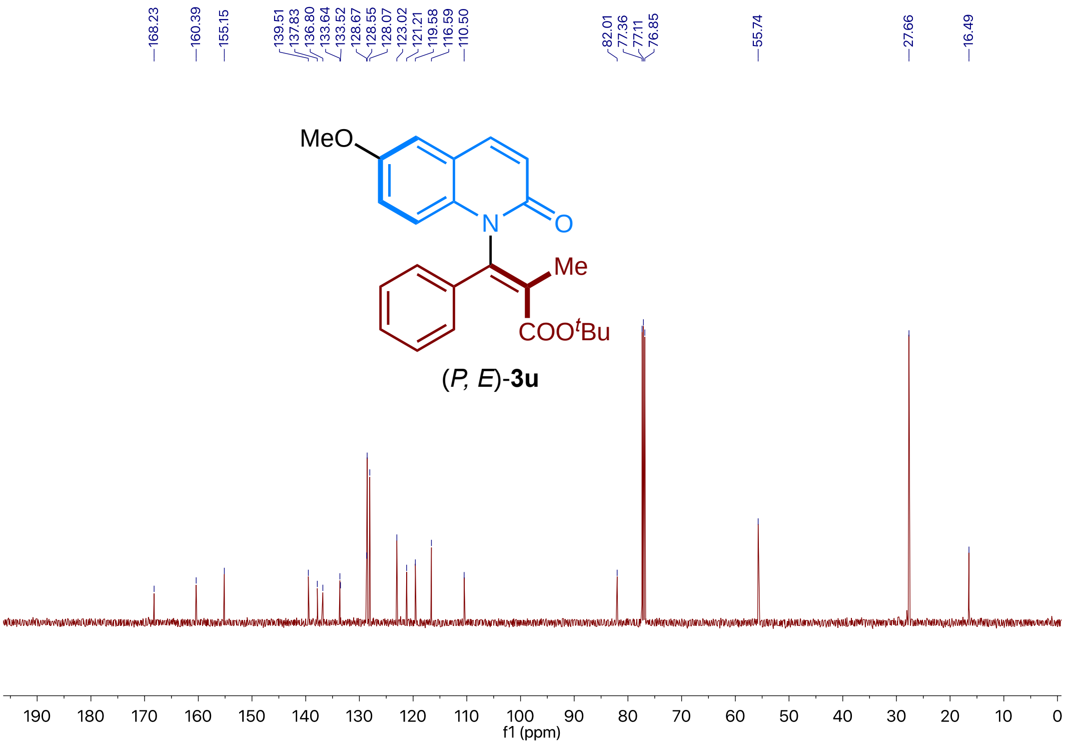

Supplementary Fig. 100. ^{13}**C NMR spectrum of (*P*, *E*)-3u**

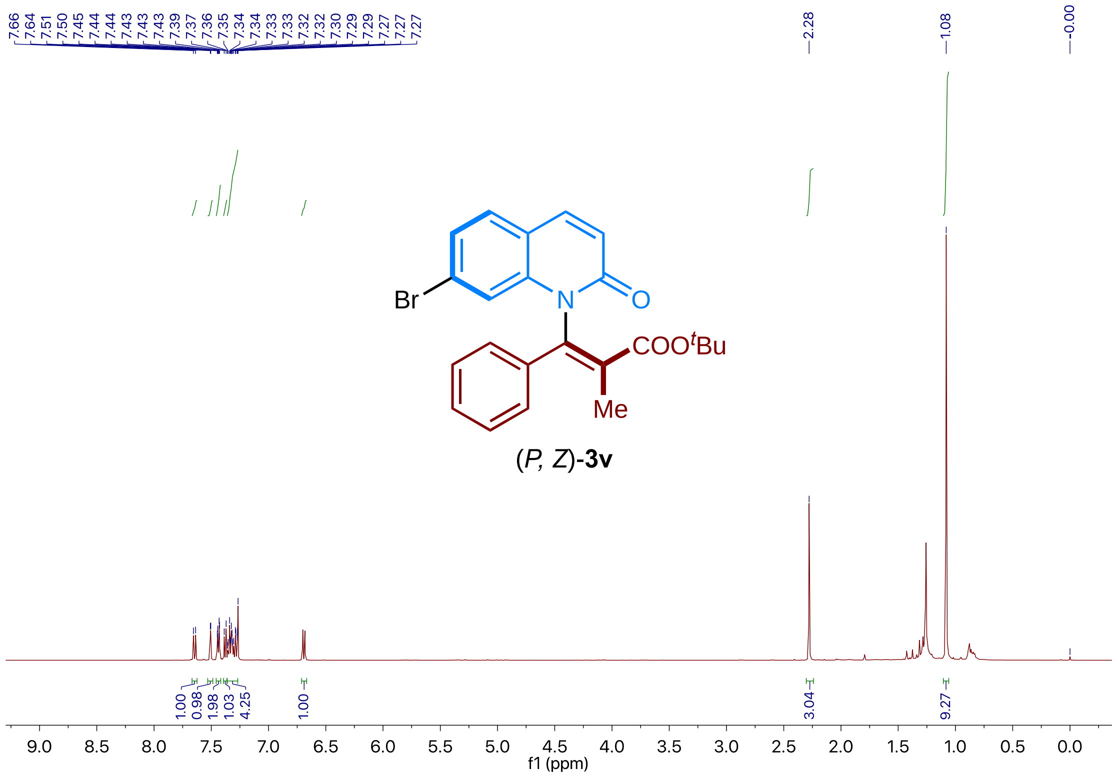

Supplementary Fig. 101. ^{1}**H NMR spectrum of (*P*, *Z*)-3v**

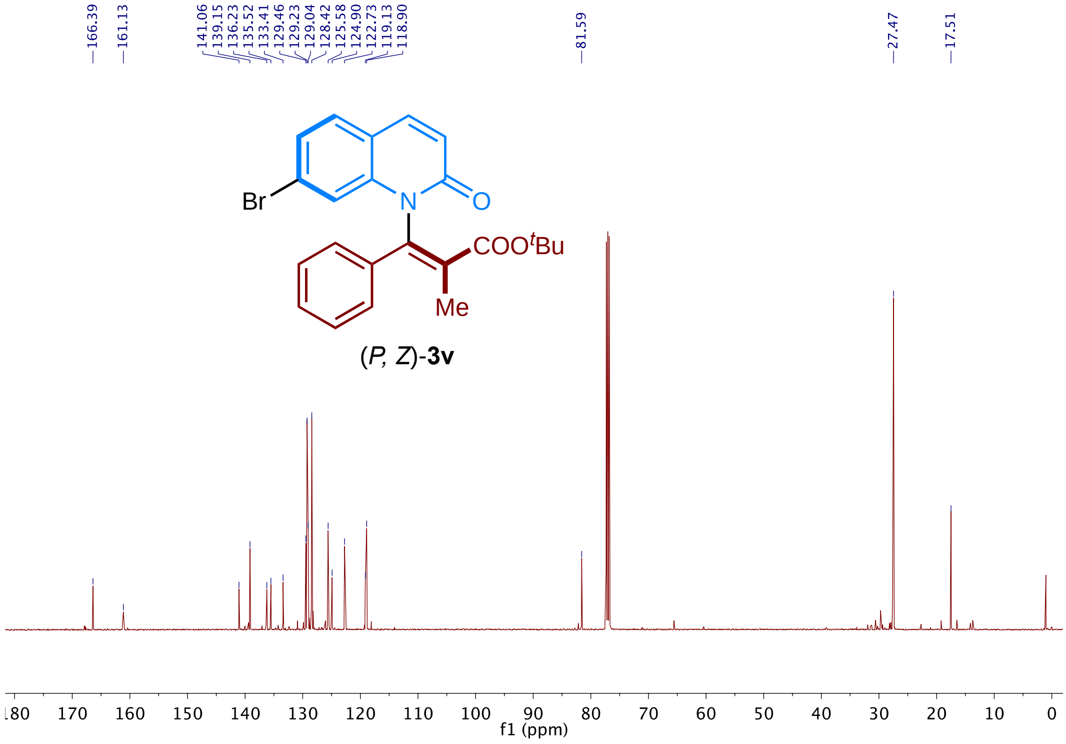

Supplementary Fig. 102. ^{13}**C NMR spectrum of (*P*, *Z*)-3v**

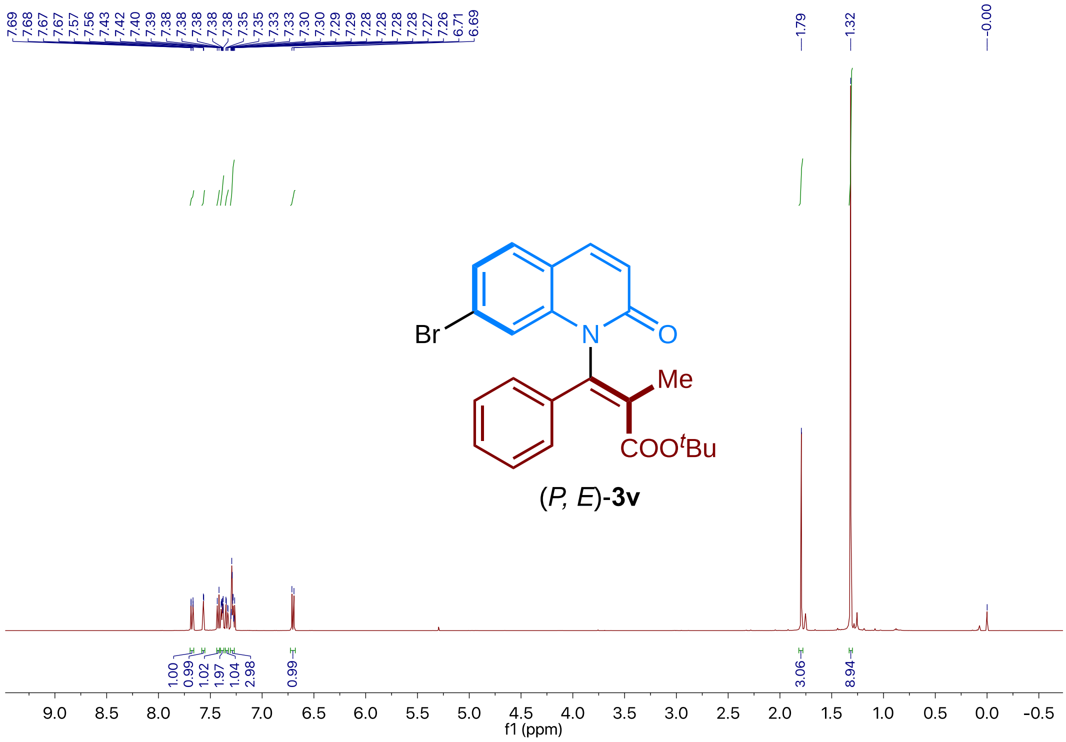

Supplementary Fig. 103. ^{1}H NMR spectrum of (*P*, *E*)-3v

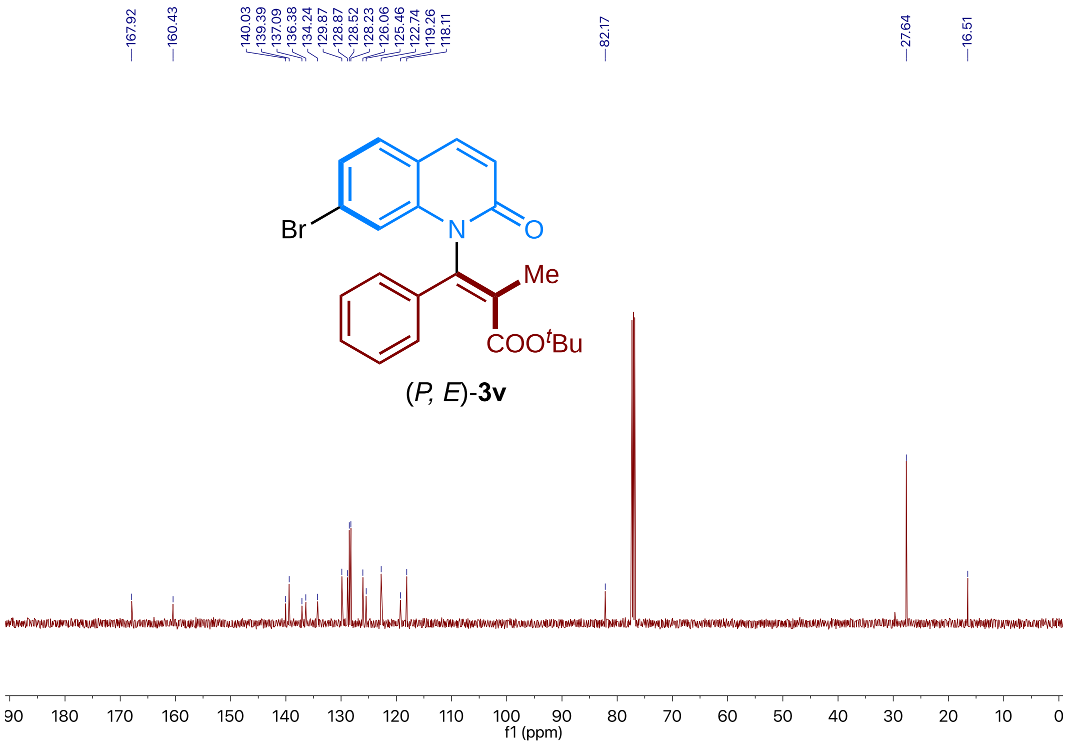

Supplementary Fig. 104. ^{13}C NMR spectrum of (*P*, *E*)-3v

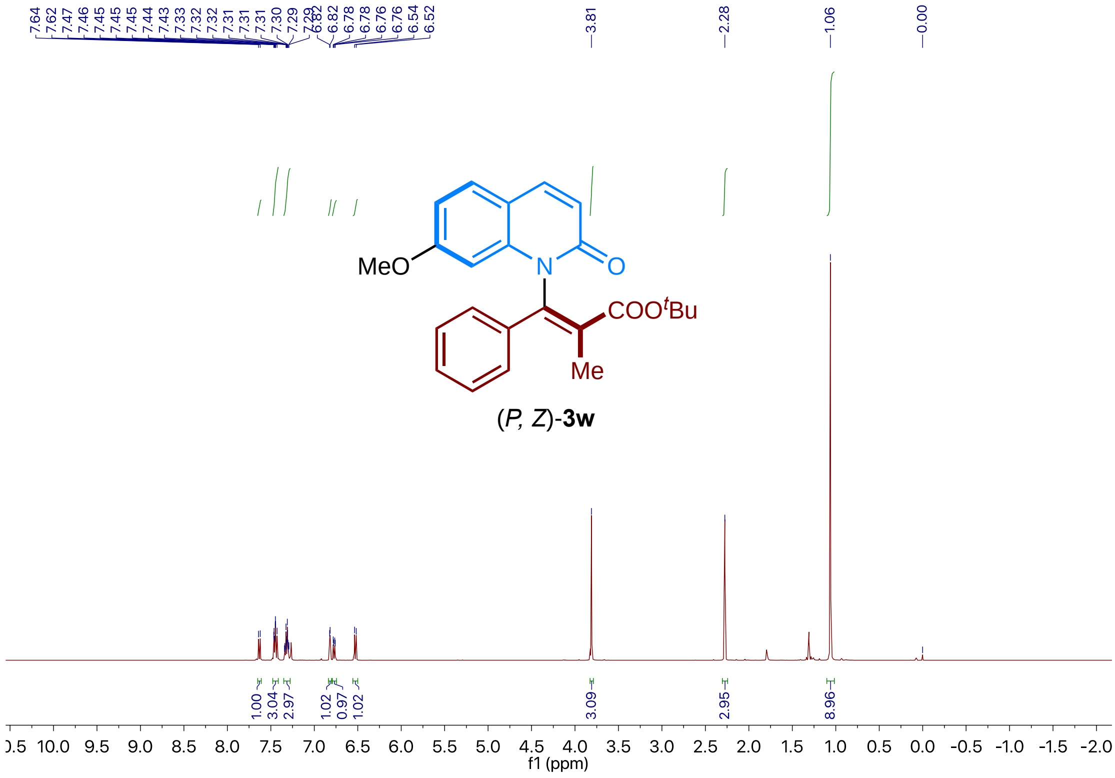

Supplementary Fig. 105. ^{1}H NMR spectrum of (*P*, *Z*)-3w

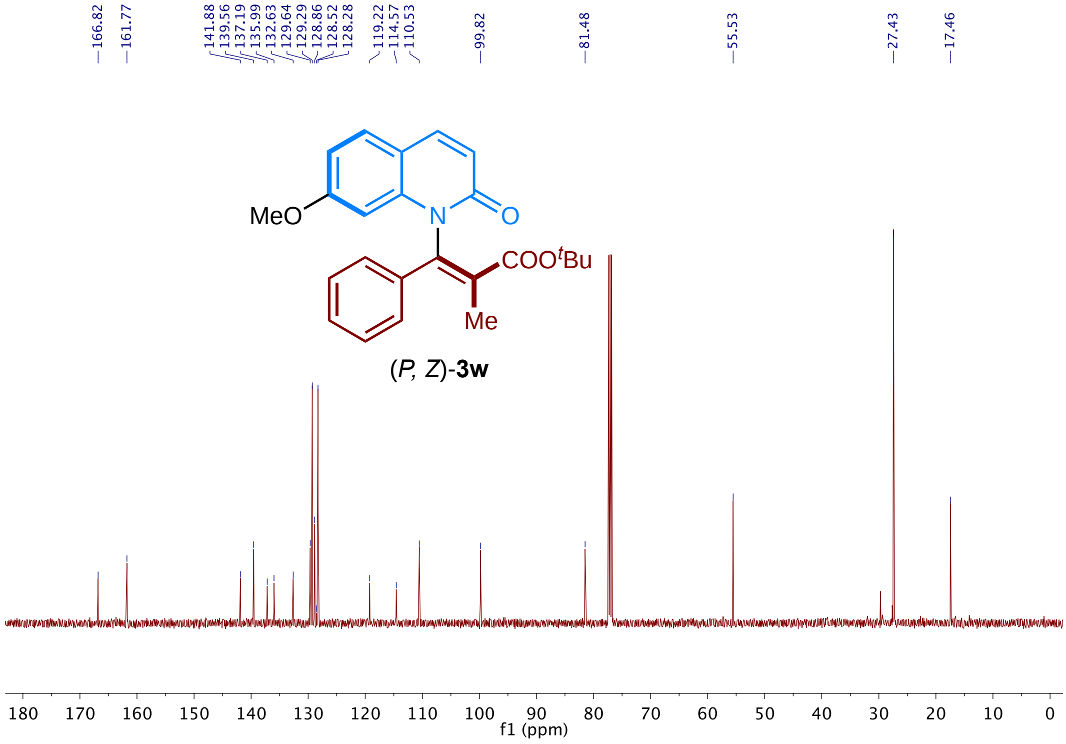

Supplementary Fig. 106. ^{13}C NMR spectrum of (*P*, *Z*)-3w

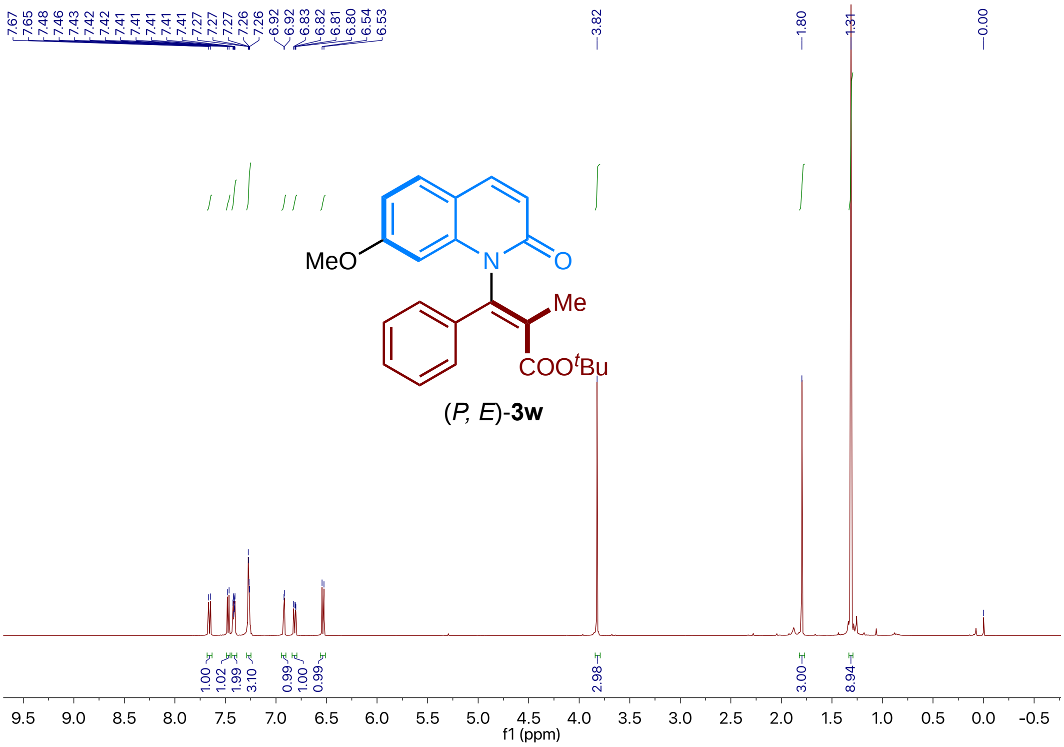

Supplementary Fig. 107. ^{1}H NMR spectrum of (*P*, *E*)-3w

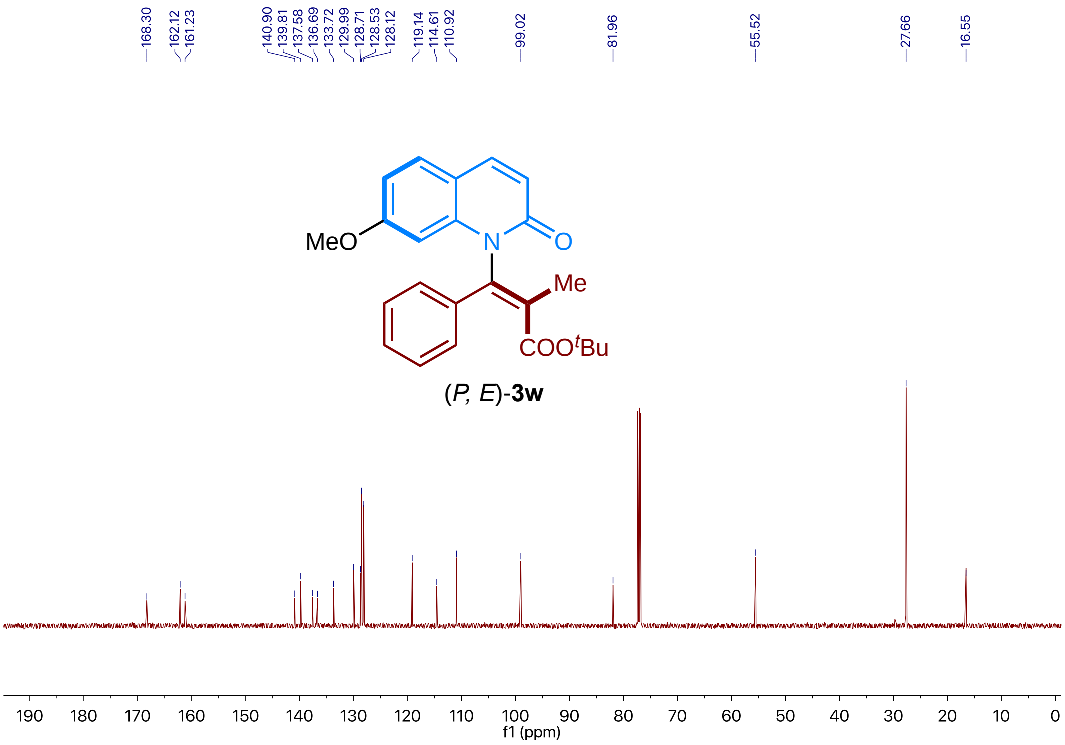

Supplementary Fig. 108. ^{13}C NMR spectrum of (*P*, *E*)-3w

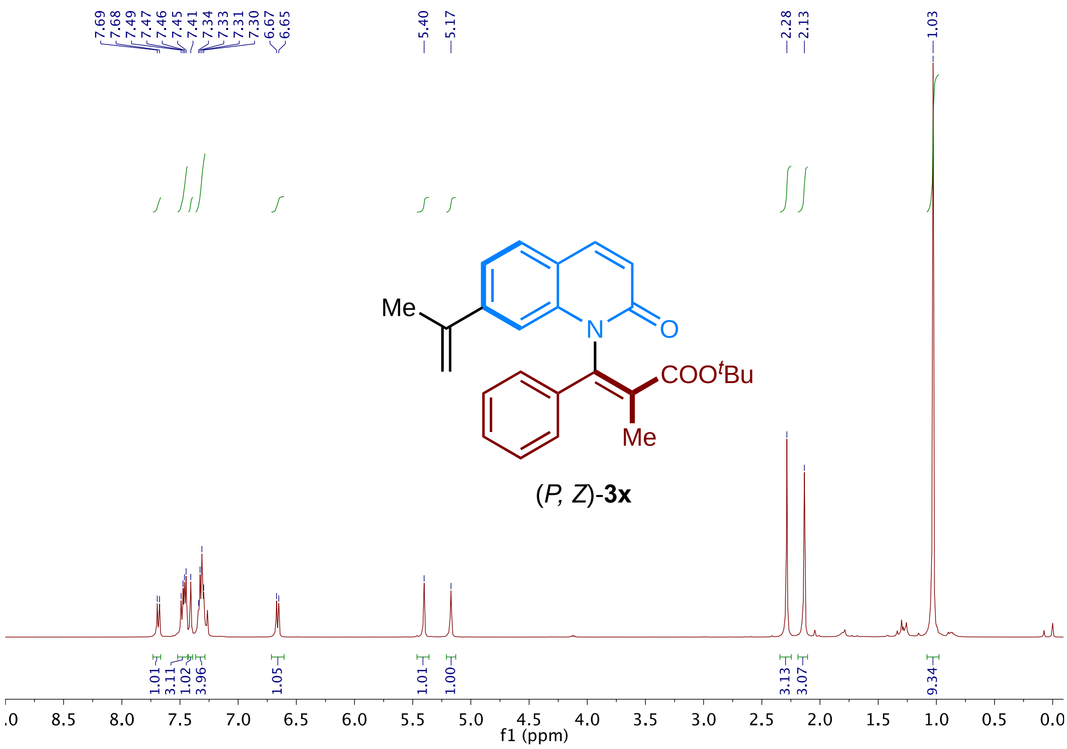

Supplementary Fig. 109. ^{1}H NMR spectrum of (*P*, *Z*)-3x

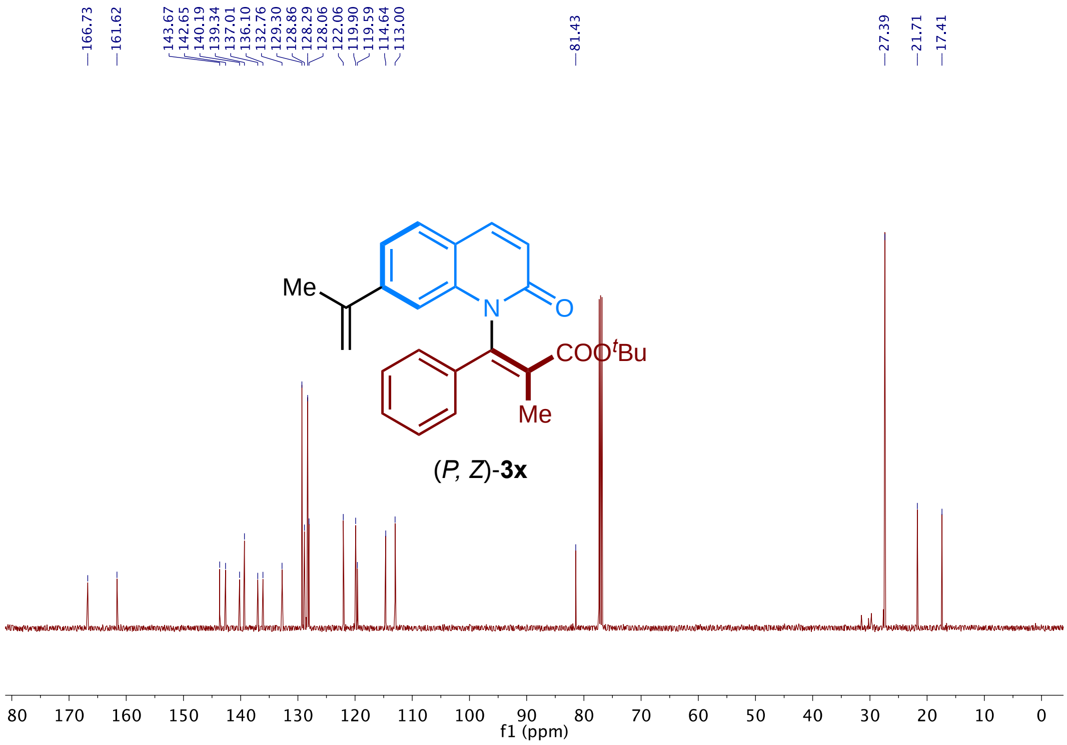

Supplementary Fig. 110. ^{13}C NMR spectrum of (*P*, *Z*)-3x

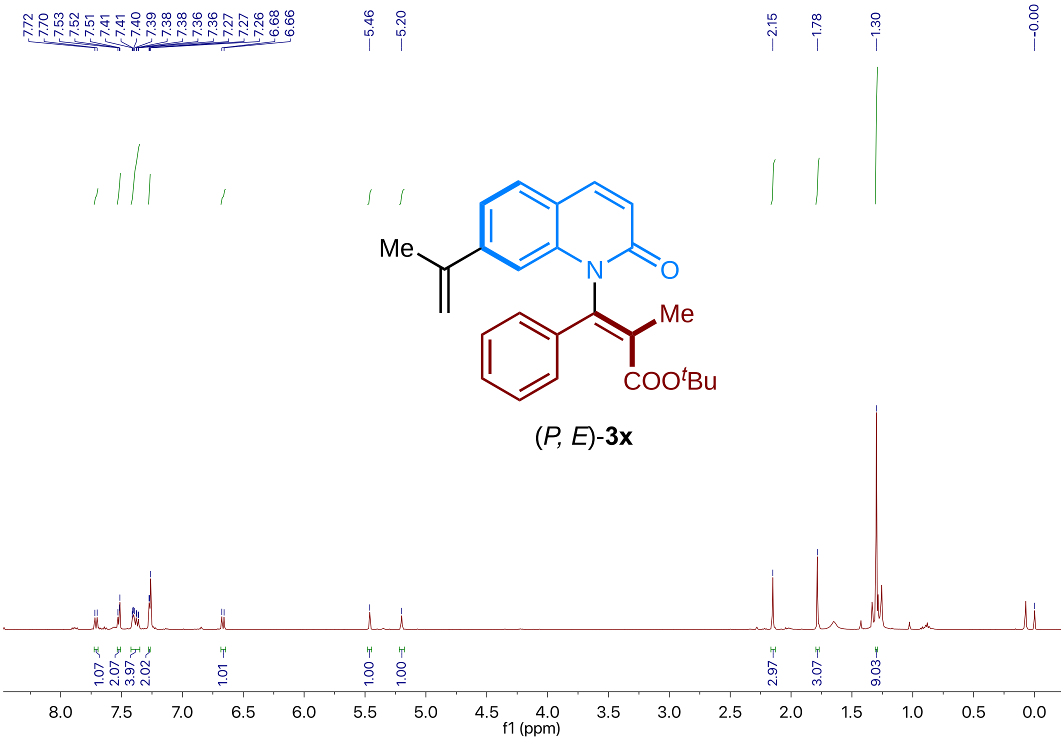

Supplementary Fig. 111. ^{1}**H NMR spectrum of (*P*, *E*)-3x**

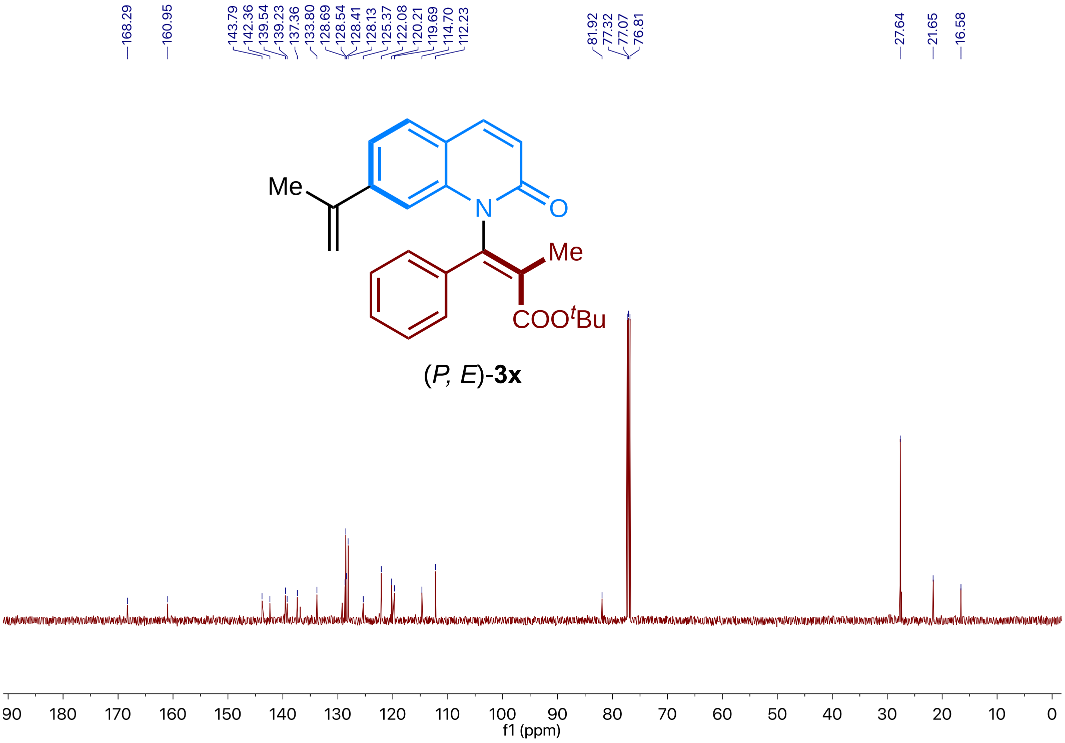

Supplementary Fig. 112. ^{13}**C NMR spectrum of (*P*, *E*)-3x**

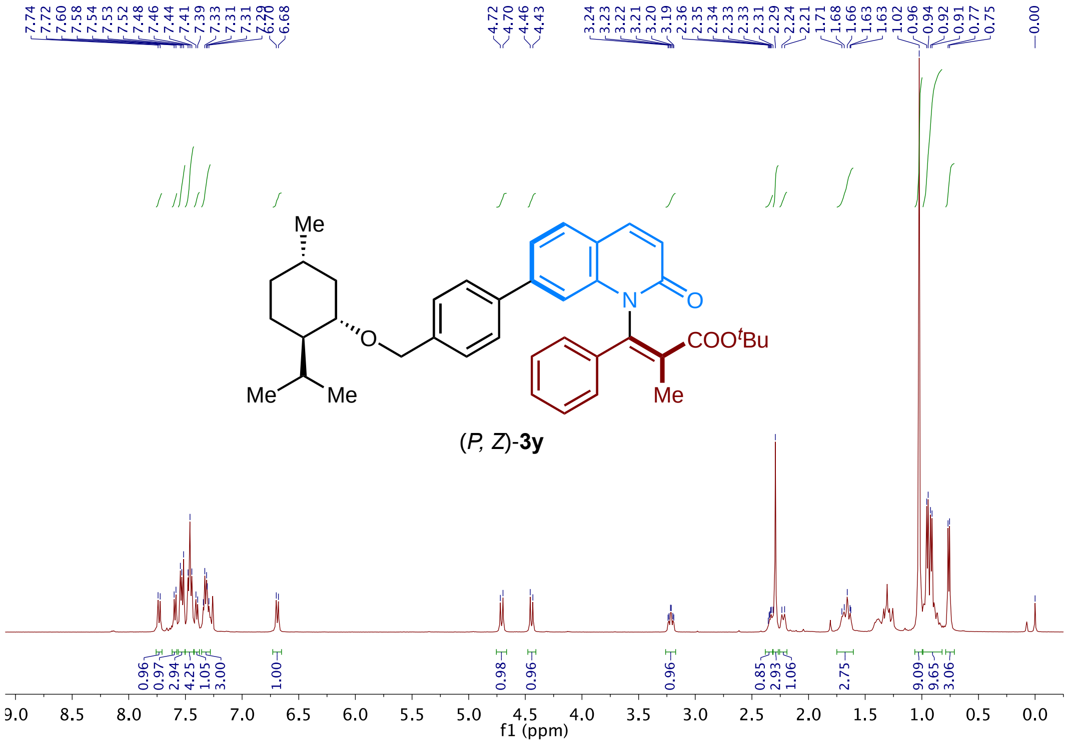

Supplementary Fig. 113. ¹H NMR spectrum of (*P*, *Z*)-3y

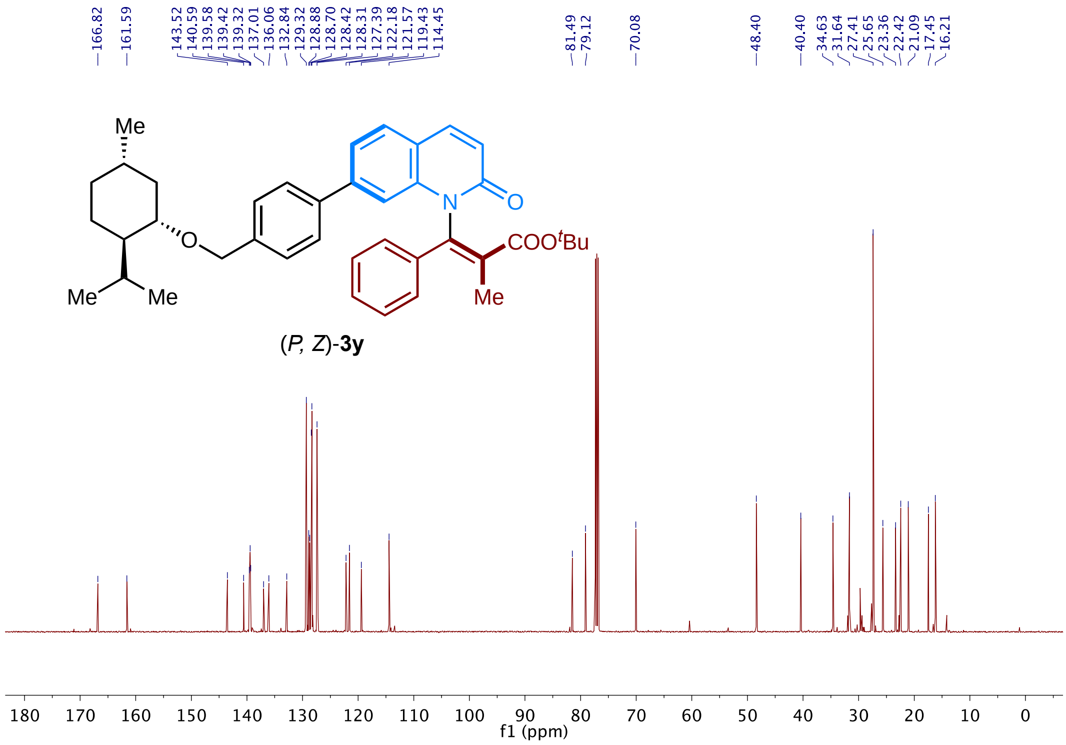

Supplementary Fig. 114. ¹³C NMR spectrum of (*P*, *Z*)-3y

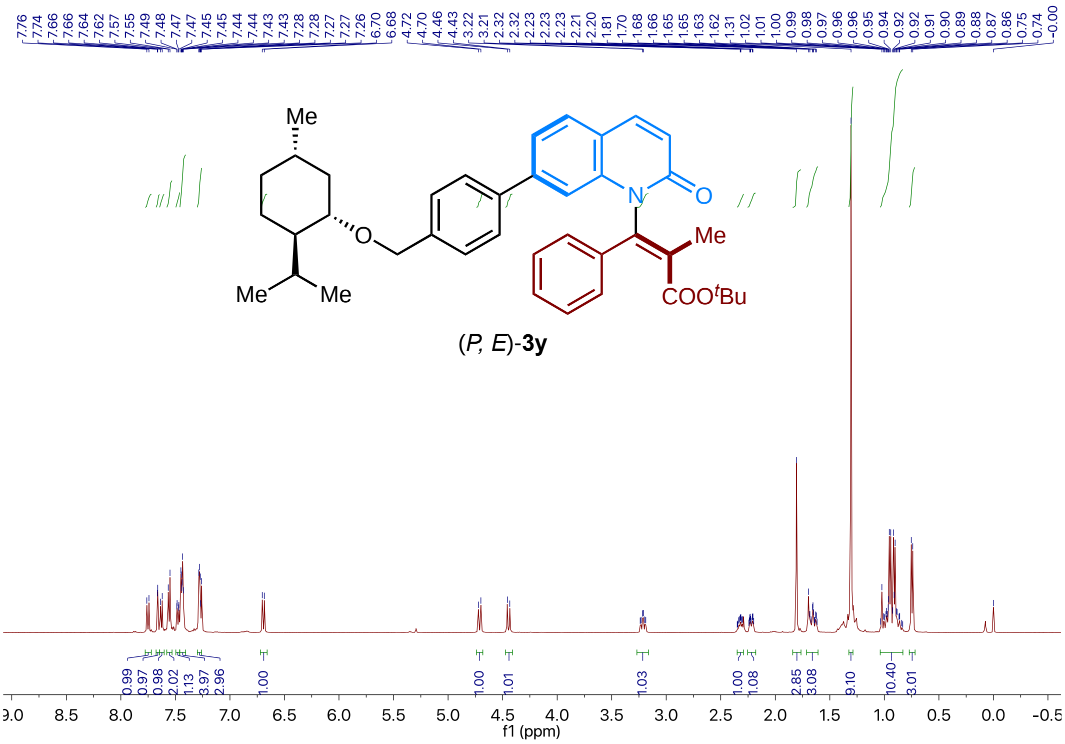

Supplementary Fig. 115. ^{1}H NMR spectrum of (*P*, *E*)-3y

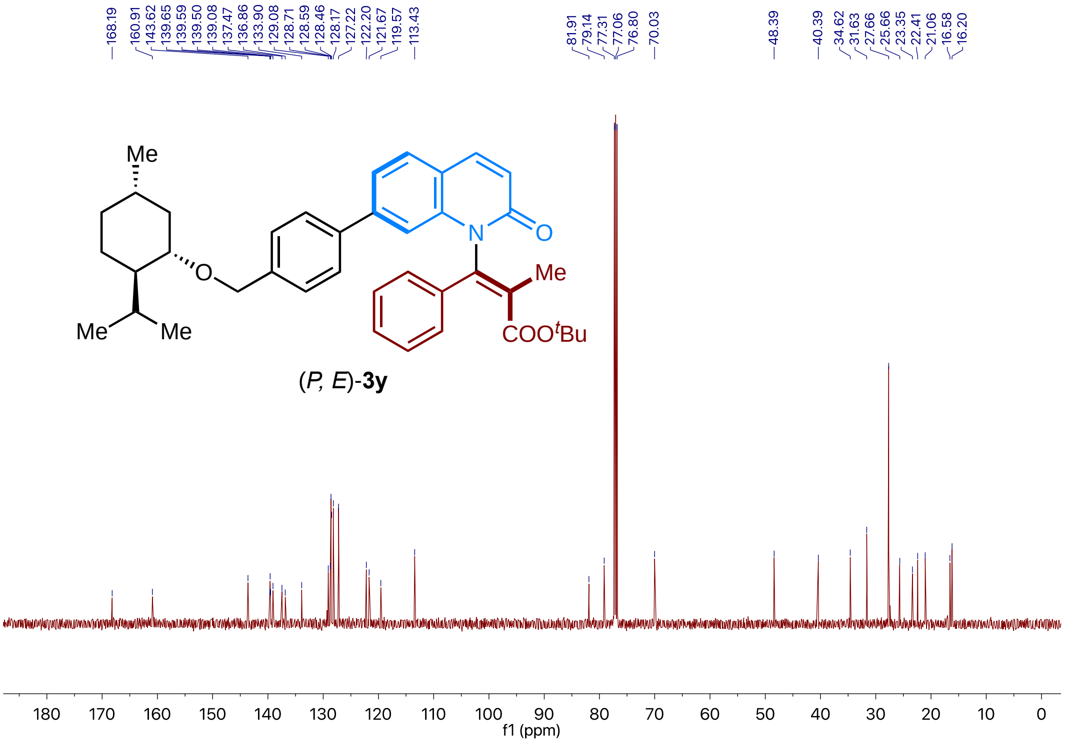

Supplementary Fig. 116. ^{13}C NMR spectrum of (*P*, *E*)-3y

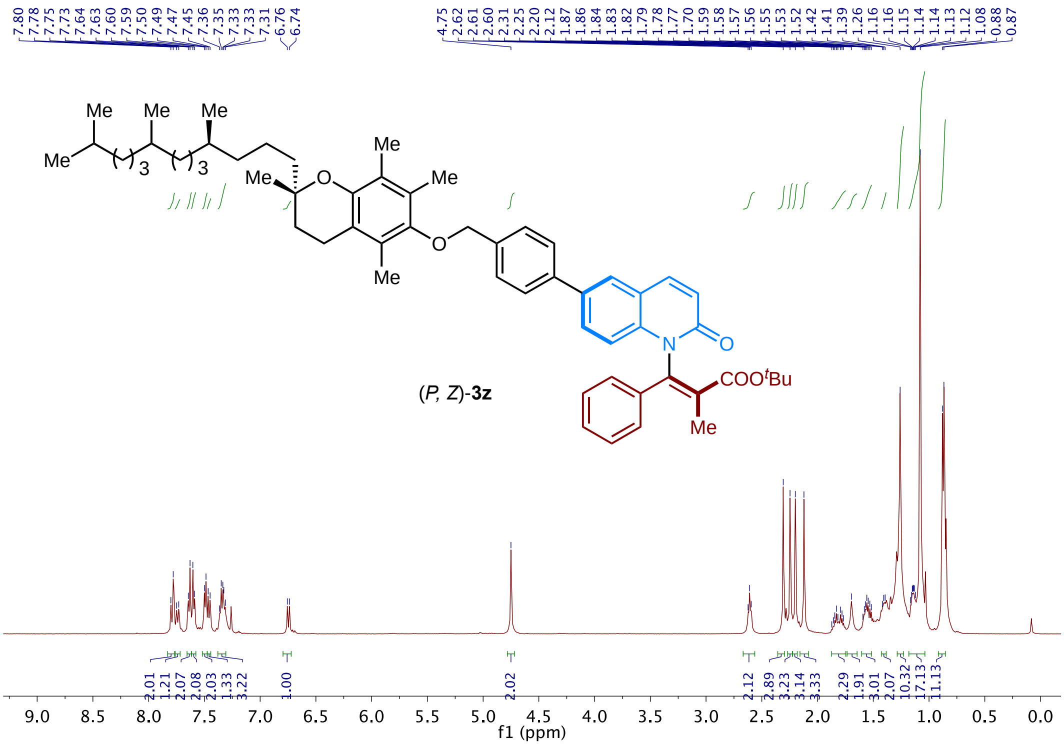

Supplementary Fig. 117. ^{1}H NMR spectrum of (*P*, *Z*)-3z

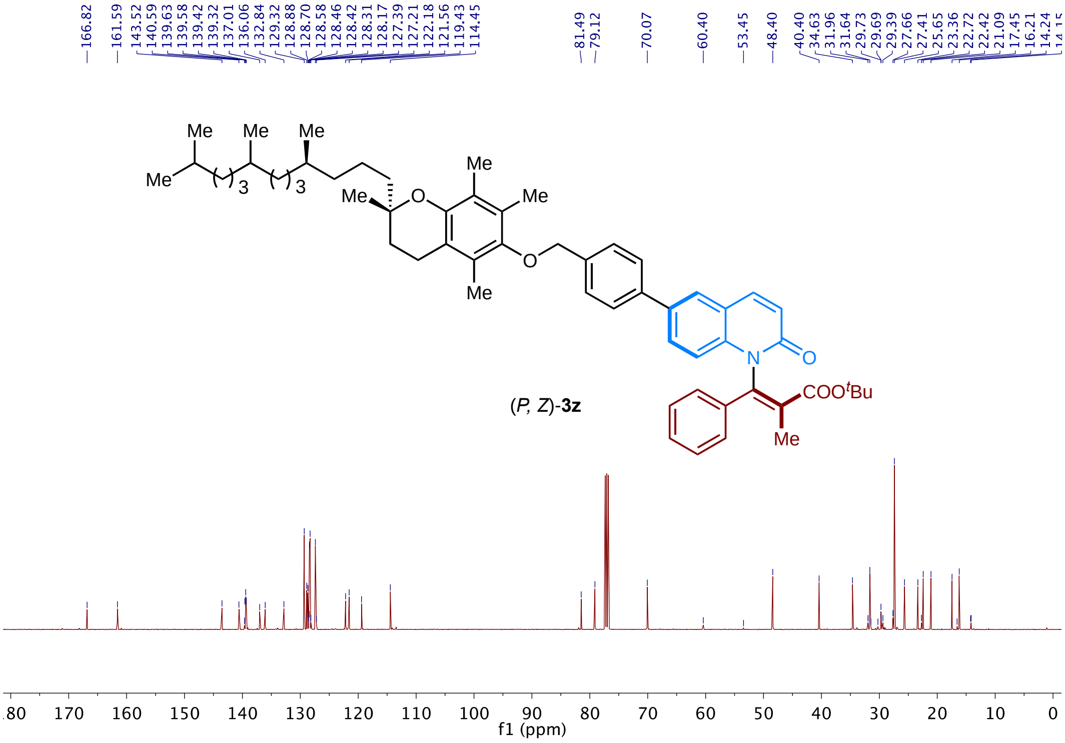

Supplementary Fig. 118. ^{13}C NMR spectrum of (*P*, *Z*)-3z

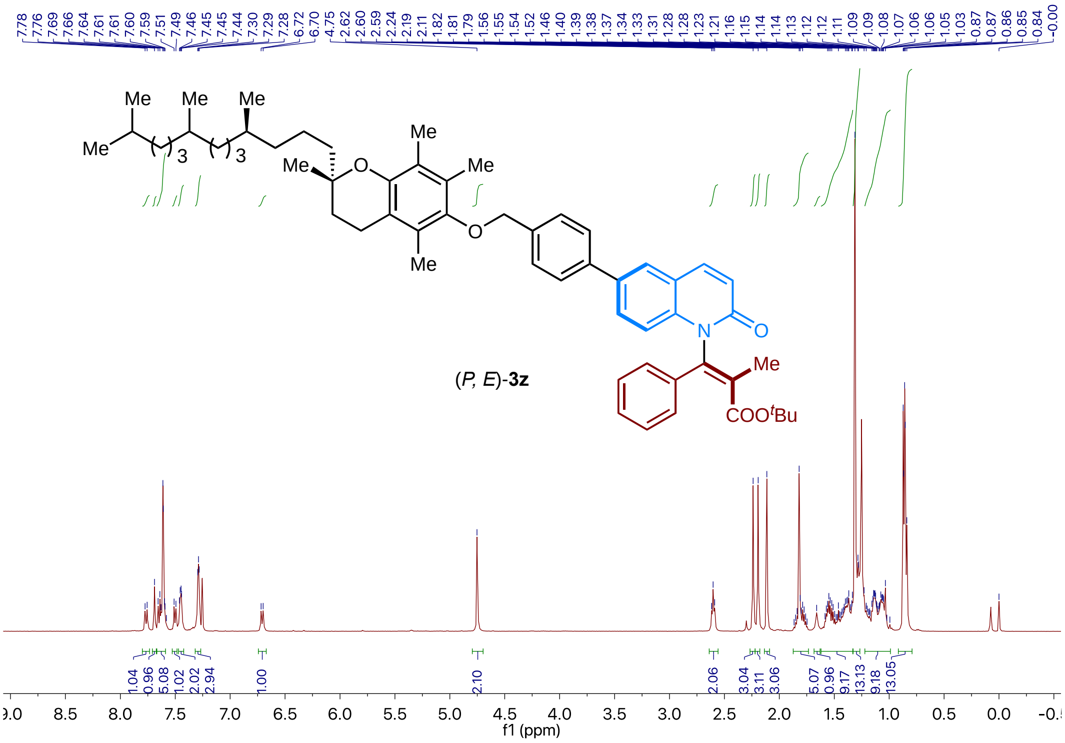

Supplementary Fig. 119. ^{1}H NMR spectrum of (*P*, *E*)-3z

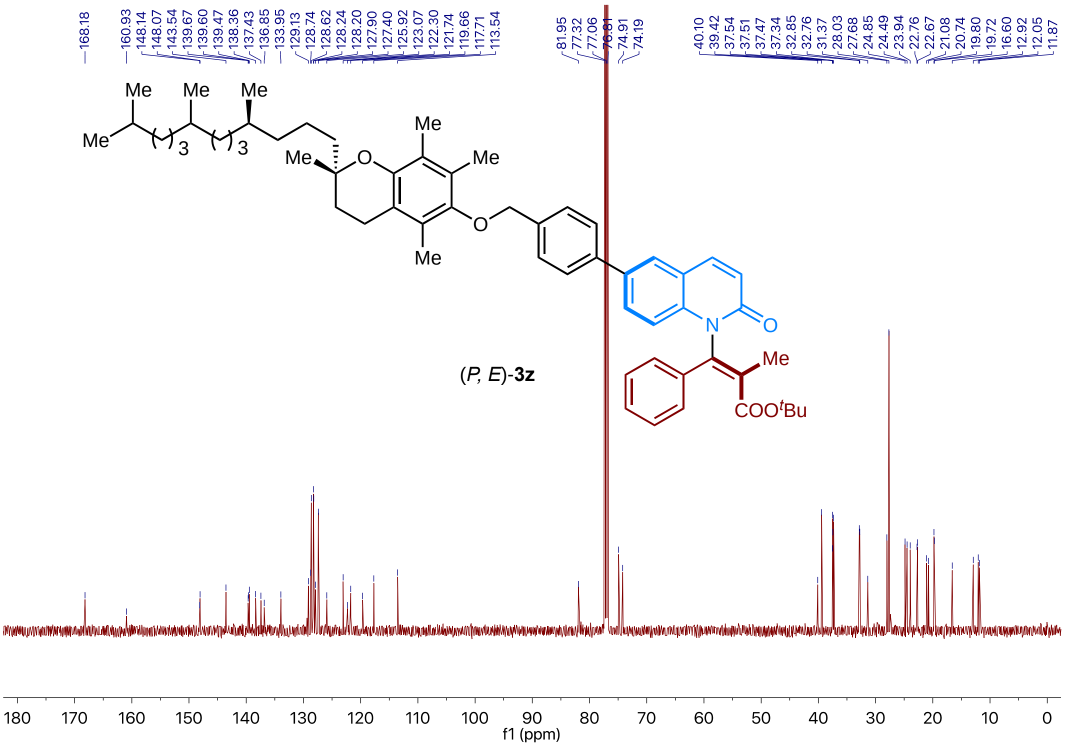

Supplementary Fig. 120. ^{13}C NMR spectrum of (*P*, *E*)-3z

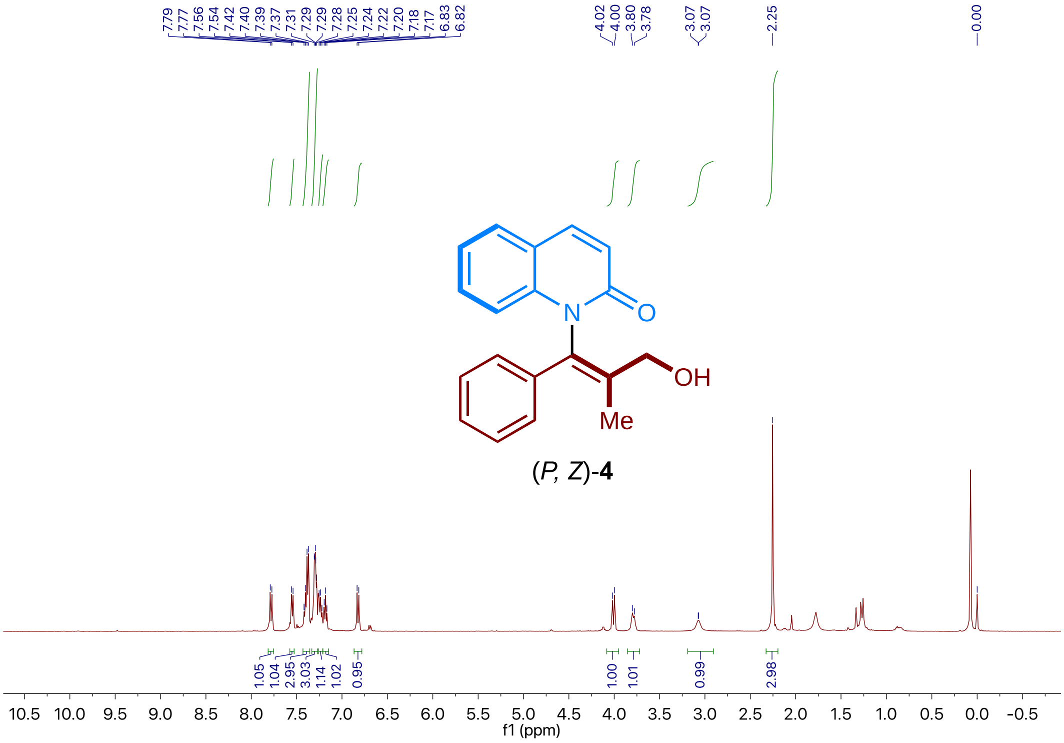

Supplementary Fig. 121. ^{1}H NMR spectrum of (*P*, *Z*)-4

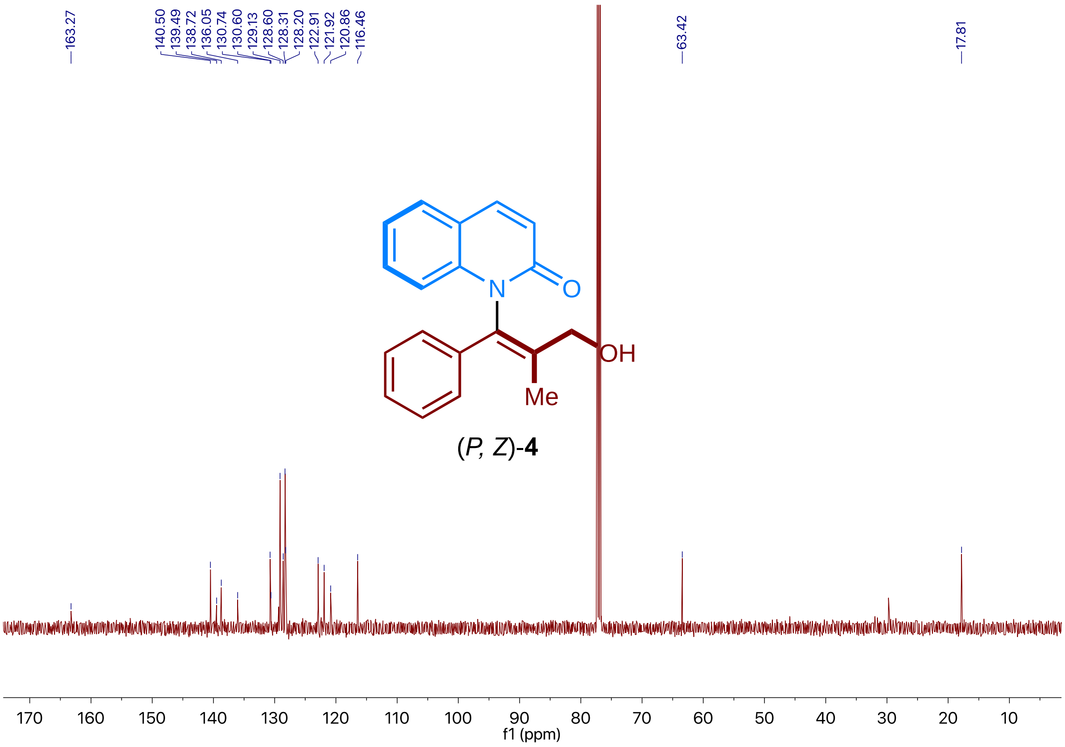

Supplementary Fig. 122. ^{13}C NMR spectrum of (*P*, *Z*)-4

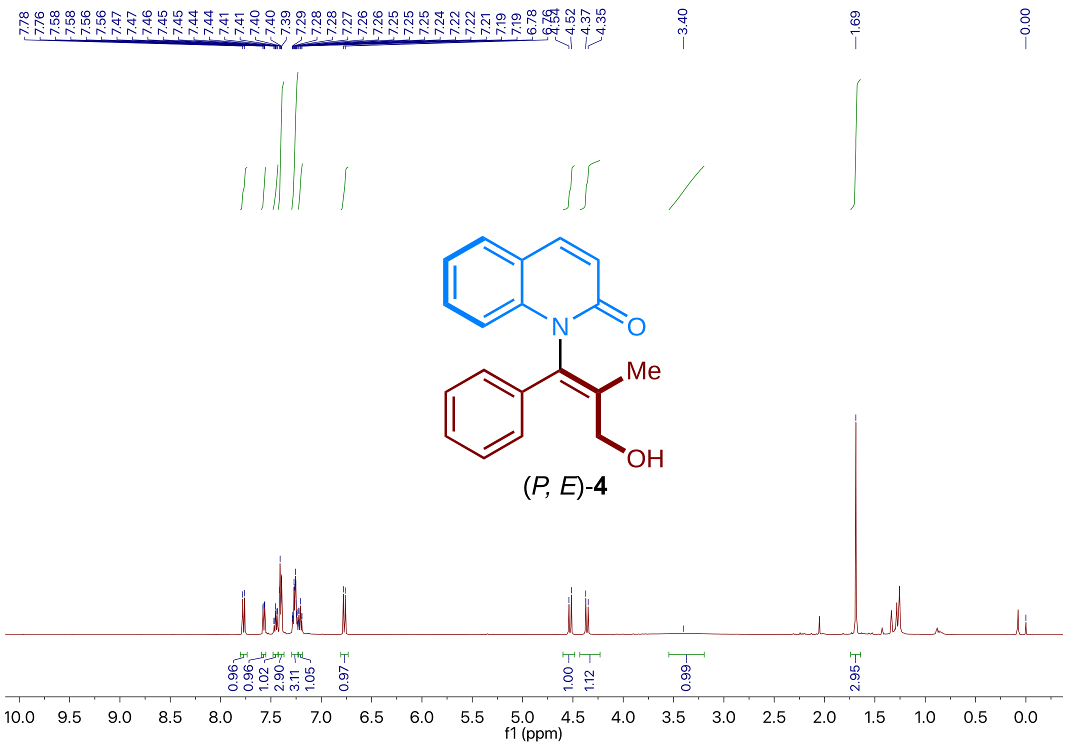

Supplementary Fig. 123. ^{1}H NMR spectrum of (*P*, *E*)-4

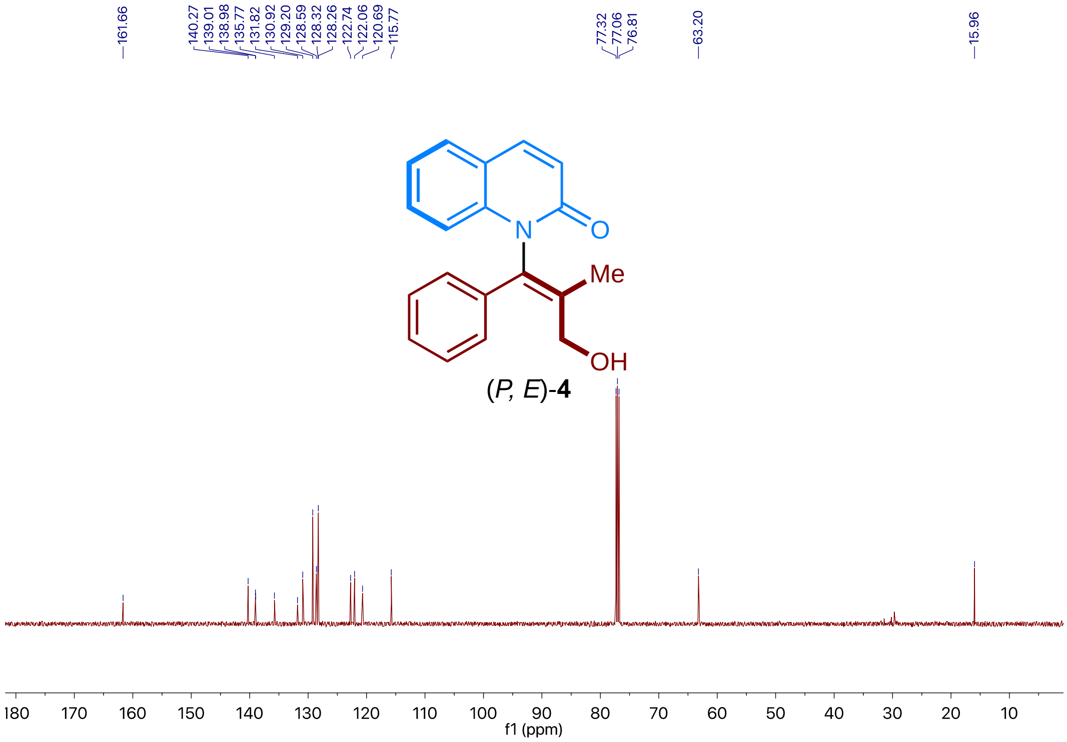

Supplementary Fig. 124. ^{13}C NMR spectrum of (*P*, *E*)-4

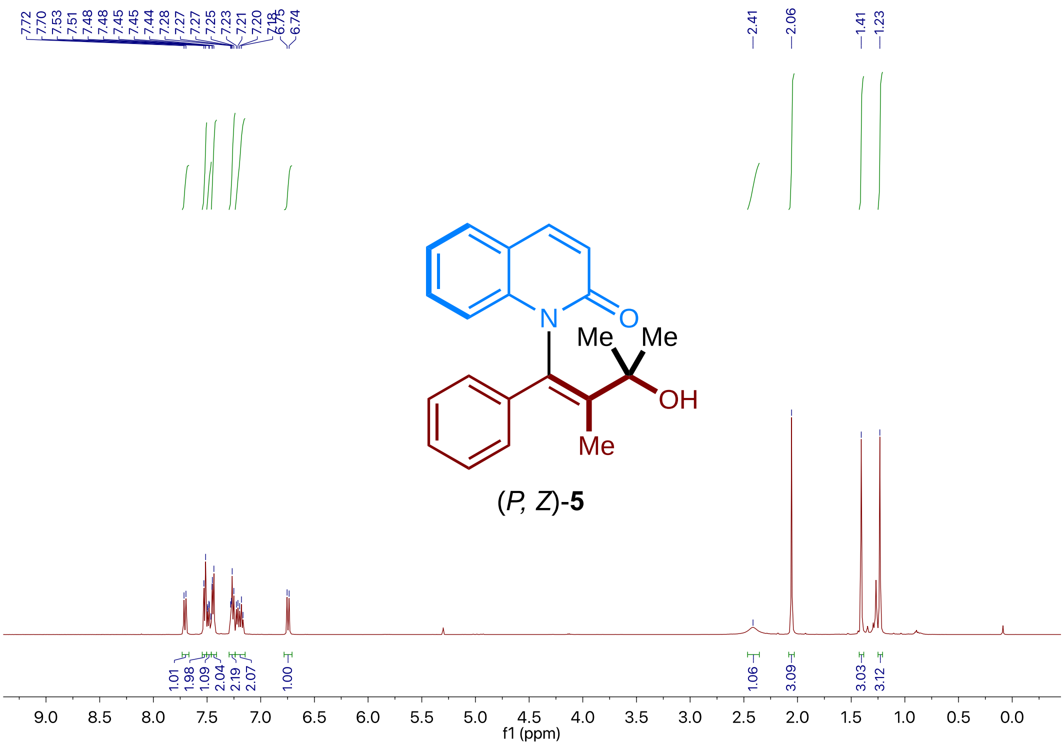

Supplementary Fig. 125. ^{1}H NMR spectrum of (*P*, *Z*)-5

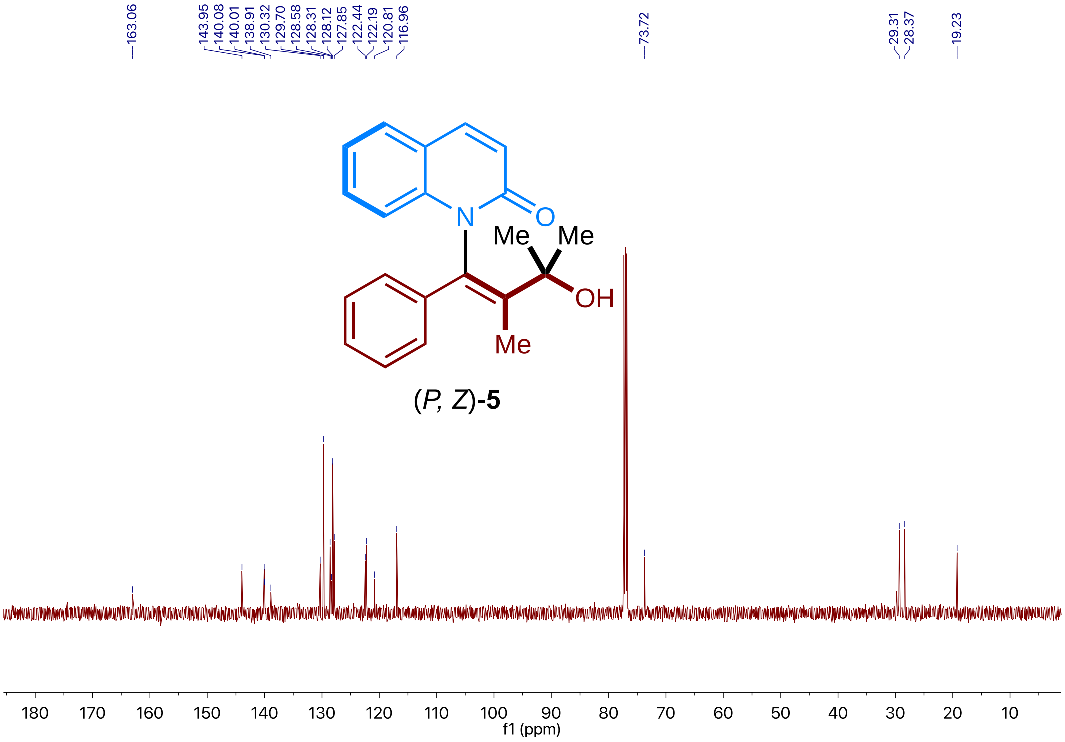

Supplementary Fig. 126. ^{13}C NMR spectrum of (*P*, *Z*)-5

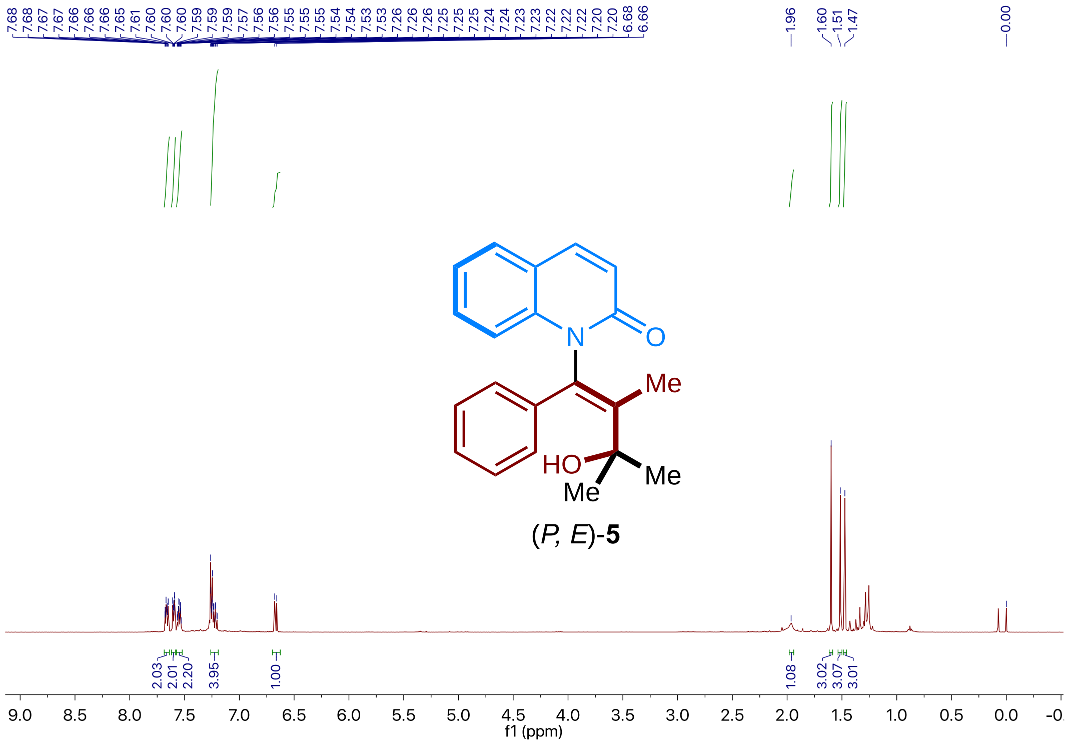

Supplementary Fig. 127. ^{1}H NMR spectrum of (*P*, *E*)-5

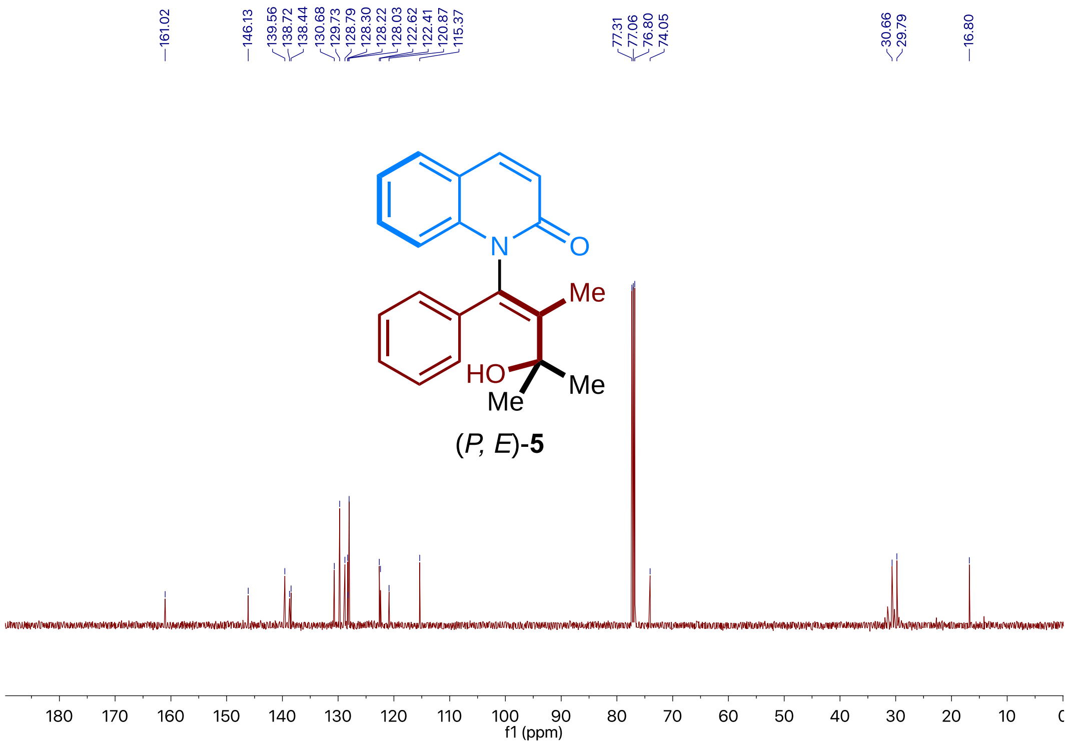

Supplementary Fig. 128. ^{13}C NMR spectrum of (*P*, *E*)-5

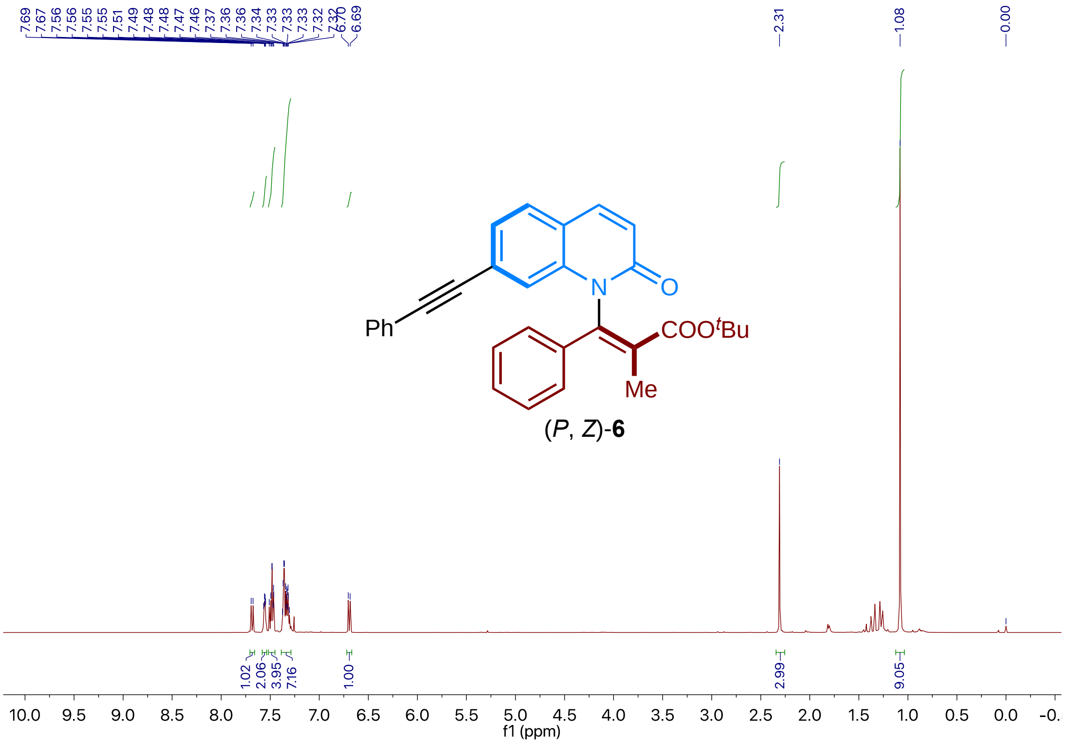

Supplementary Fig. 129. ^{1}H NMR spectrum of (*P*, *Z*)-6

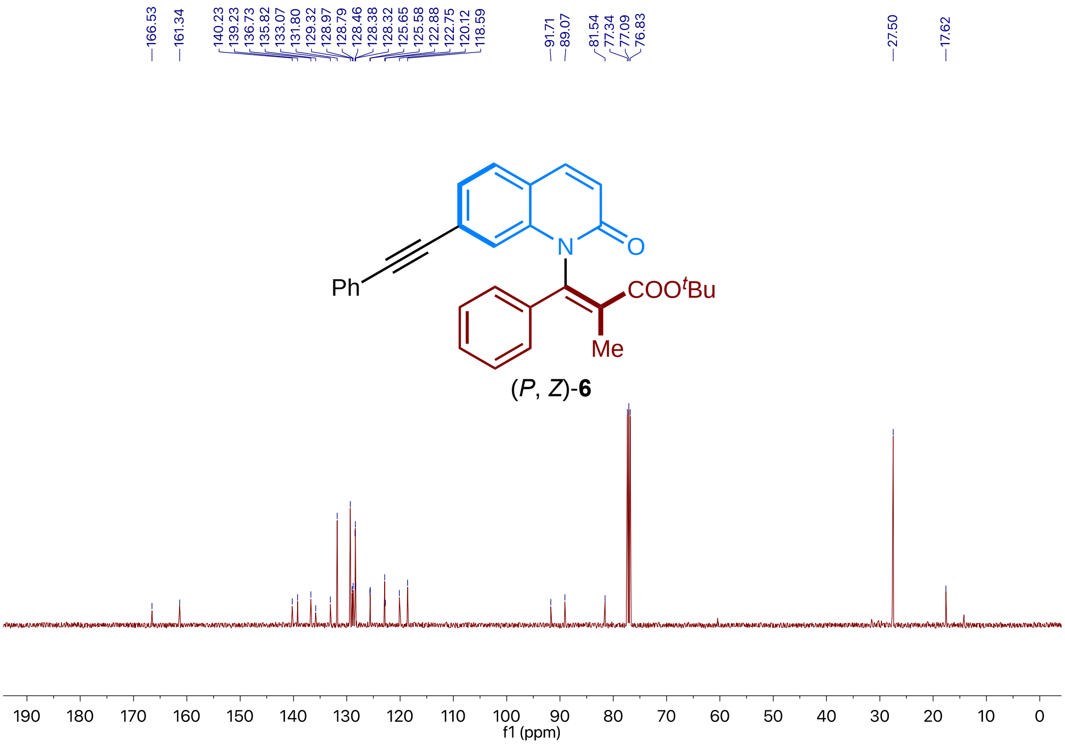

Supplementary Fig. 130. ^{13}C NMR spectrum of (*P*, *Z*)-6

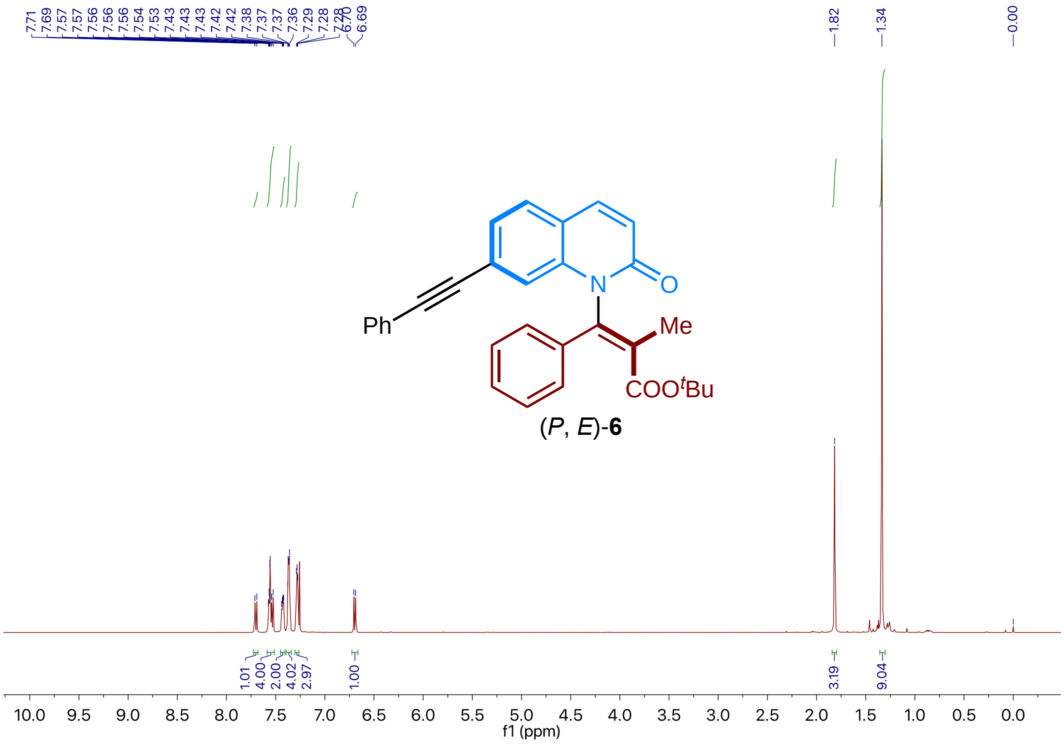

Supplementary Fig. 131. ^{1}**H NMR spectrum of (*P*, *E*)-6**

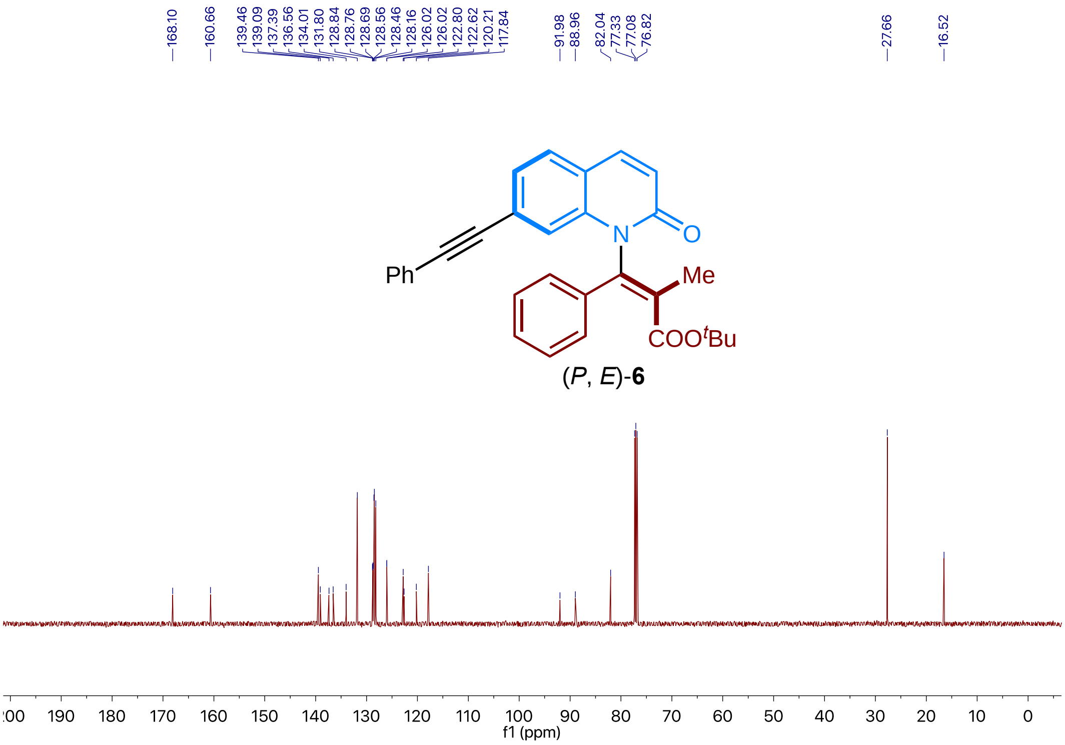

Supplementary Fig. 132. ^{13}**C NMR spectrum of (*P*, *E*)-6**

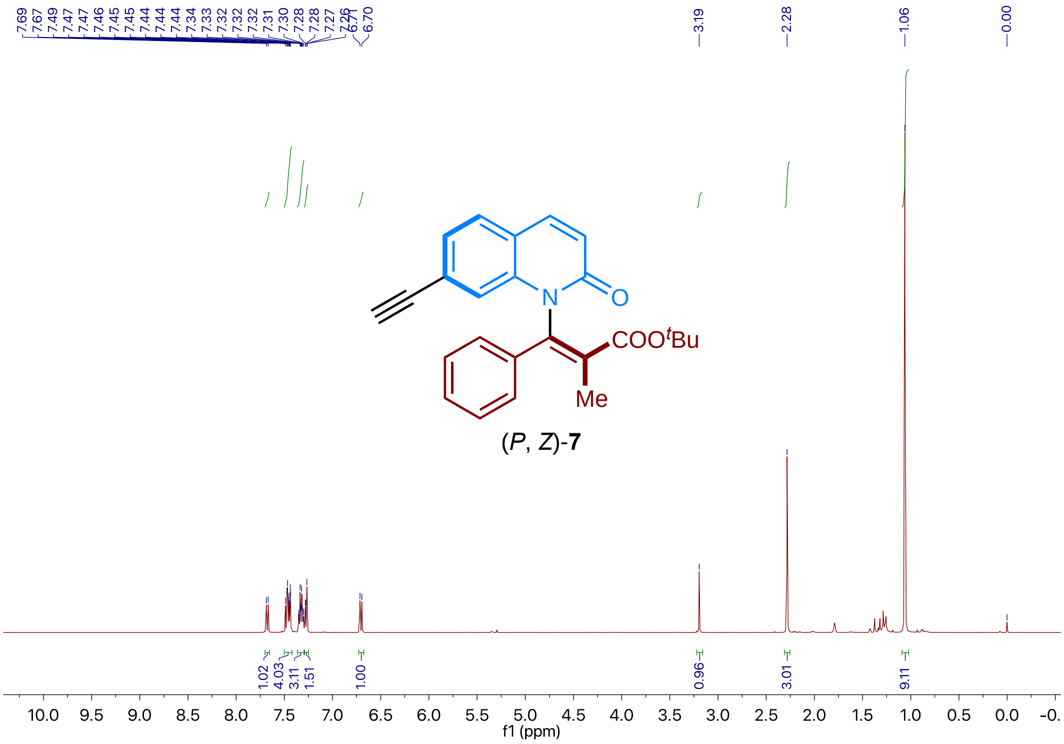

Supplementary Fig. 133. ^{1}**H NMR spectrum of (*P*, *Z*)-7**

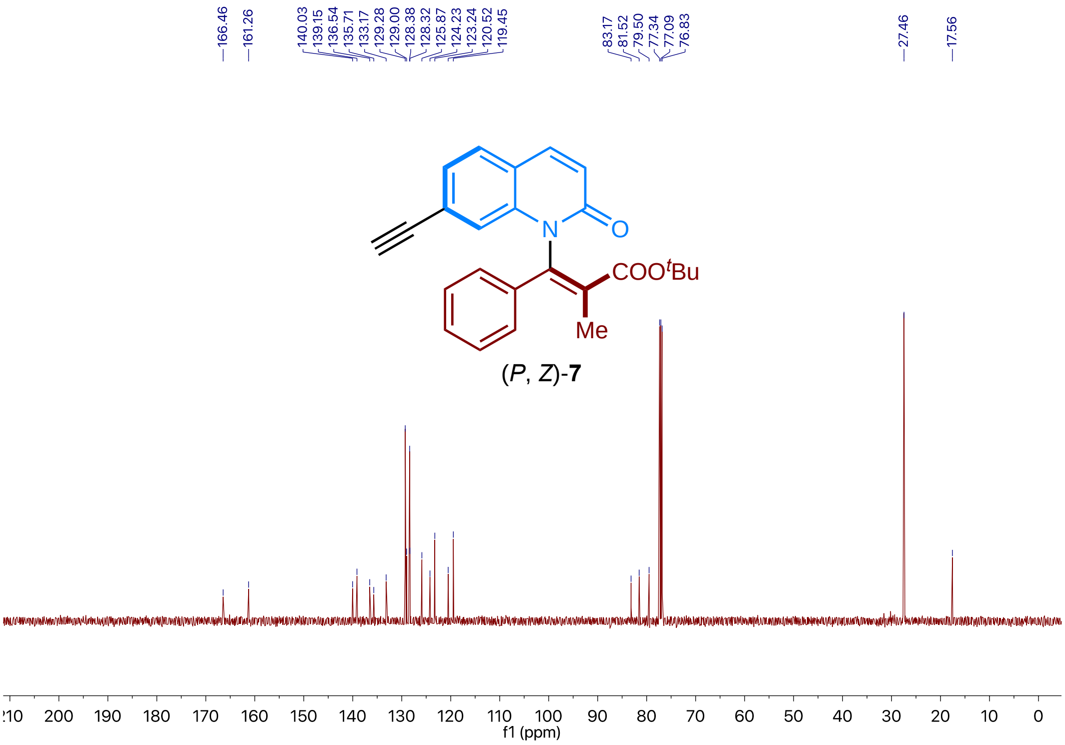

Supplementary Fig. 134. ^{13}**C NMR spectrum of (*P*, *Z*)-7**

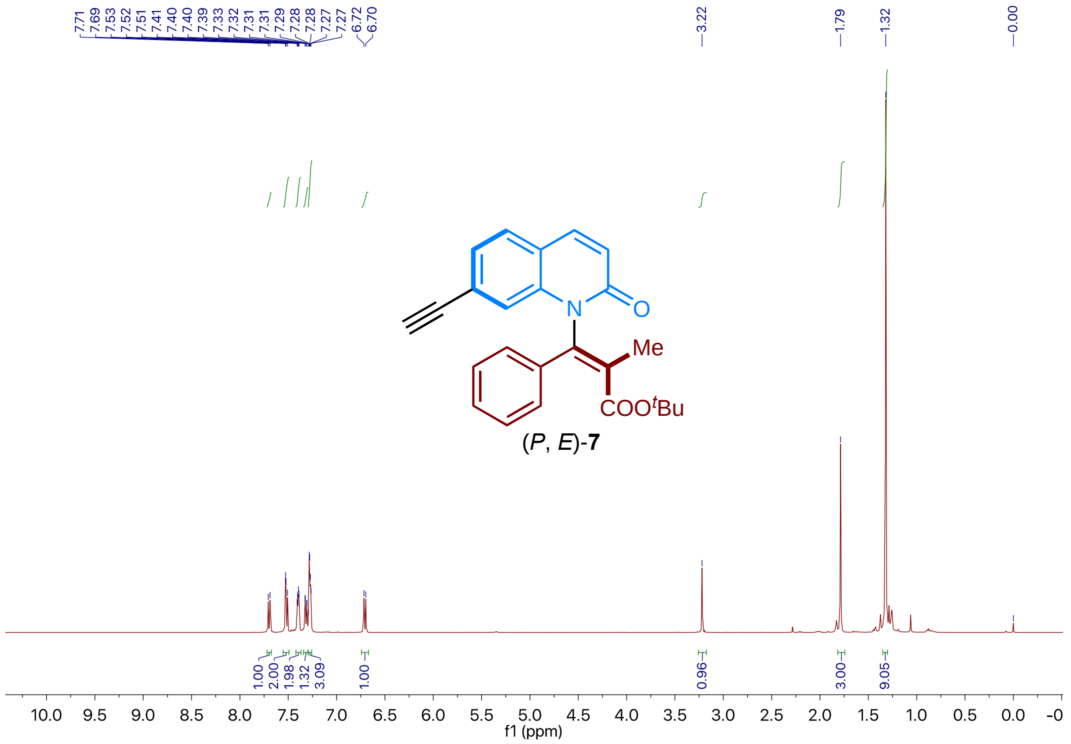

Supplementary Fig. 135. ^{1}H NMR spectrum of (*P*, *E*)-7

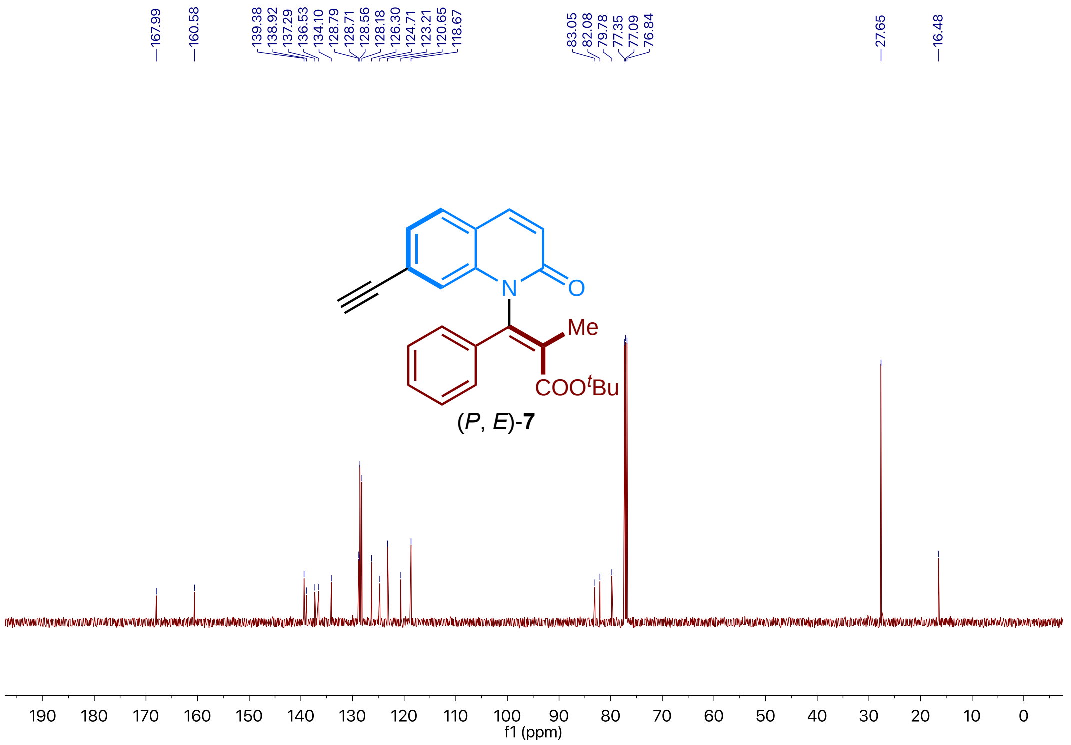

Supplementary Fig. 136. ^{13}C NMR spectrum of (*P*, *E*)-7

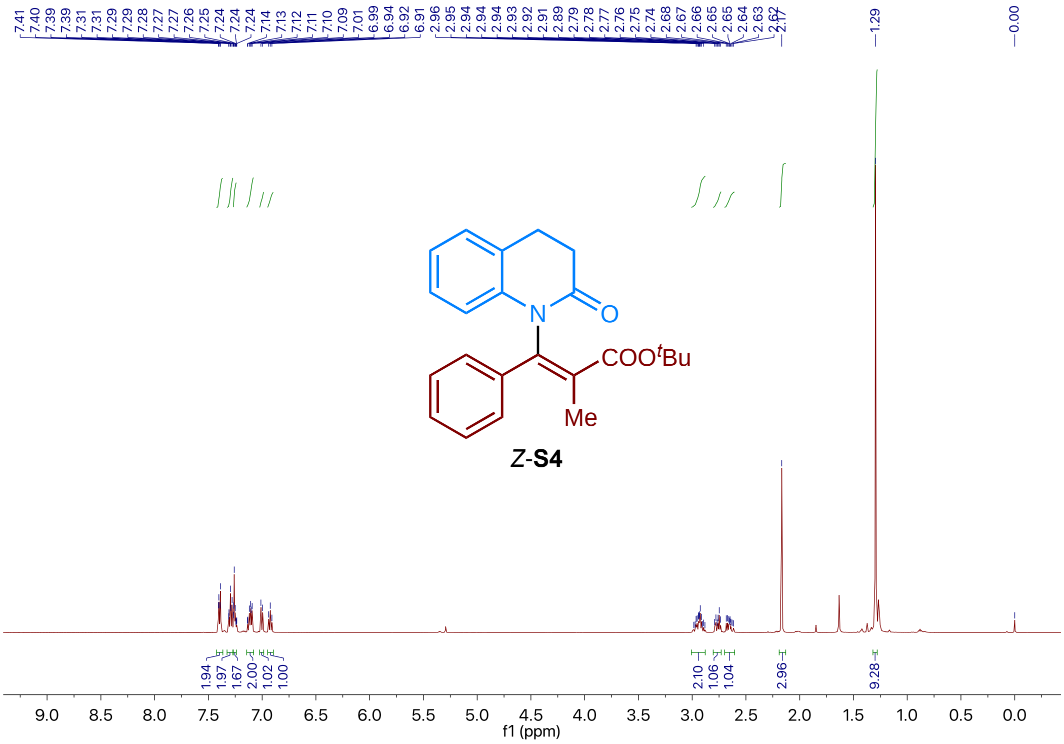

Supplementary Fig. 137. ^{1}H NMR spectrum of *Z*-S4

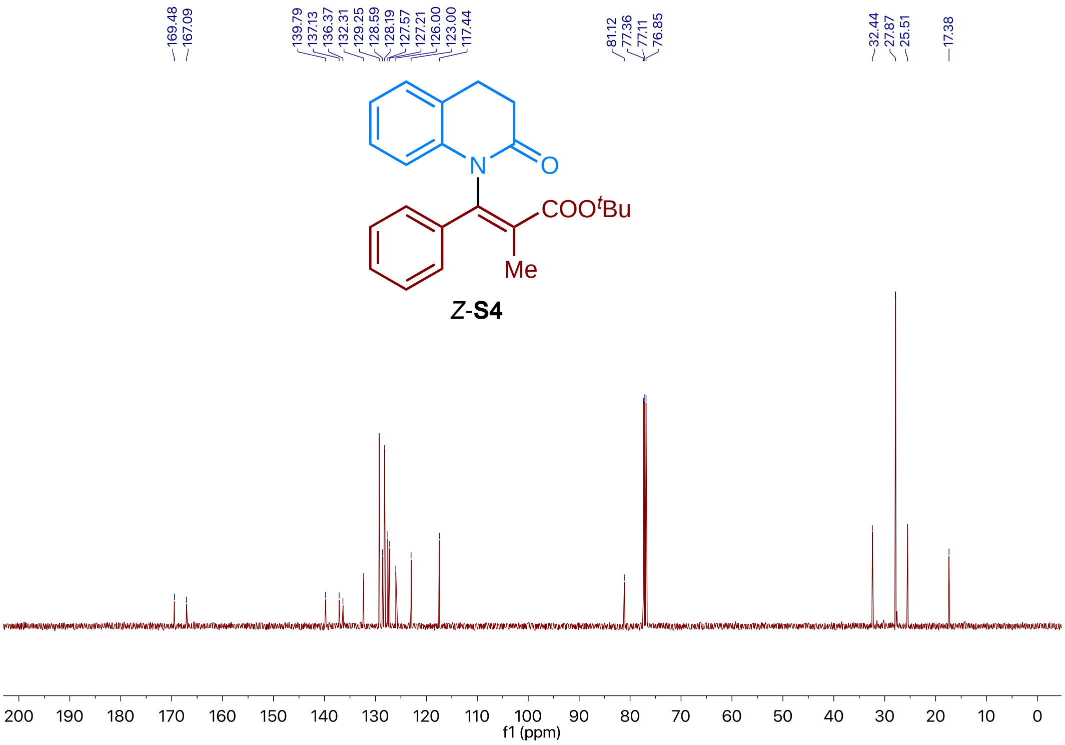

Supplementary Fig. 138. ^{13}C NMR spectrum of *Z*-S4

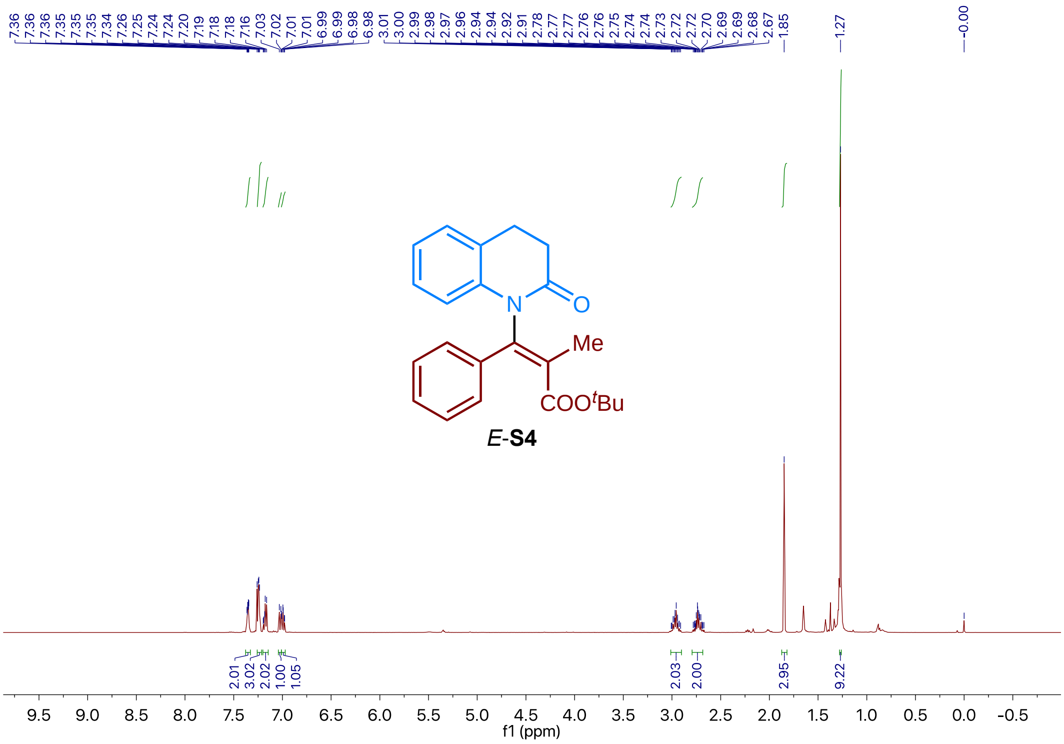

Supplementary Fig. 139. ^{1}H NMR spectrum of *E*-S4

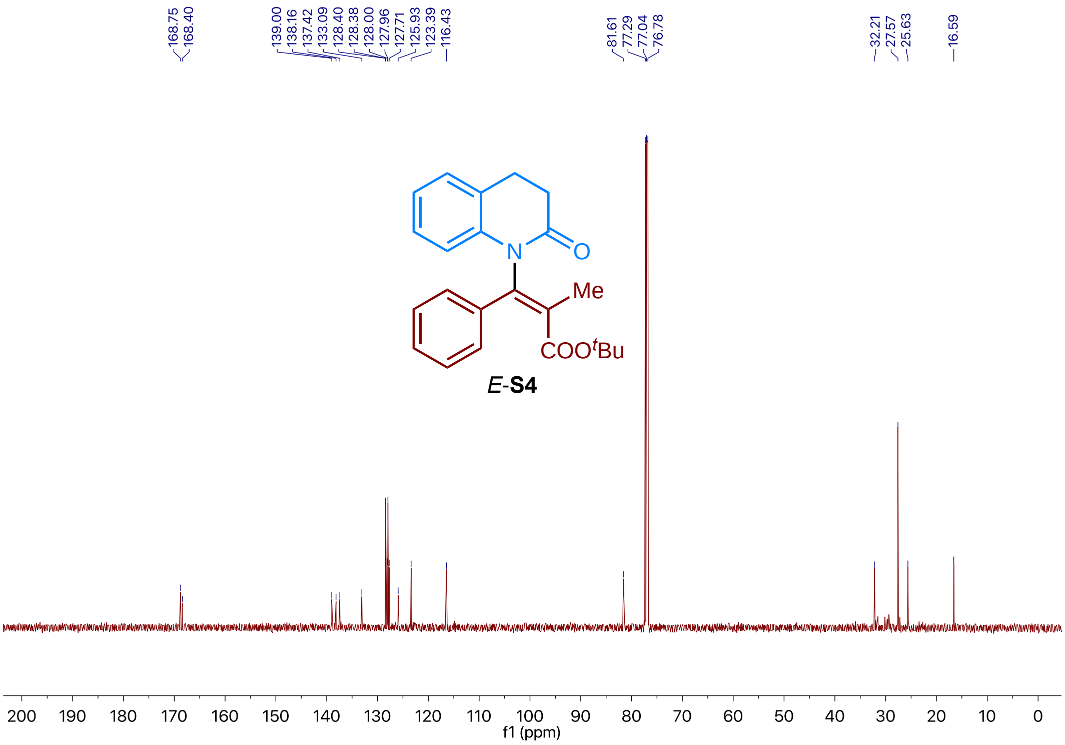

Supplementary Fig. 140. ^{13}C NMR spectrum of *E*-S4

HPLC spectrum data

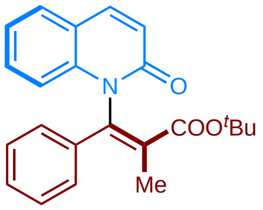

(*P*, *Z*)-**3a**

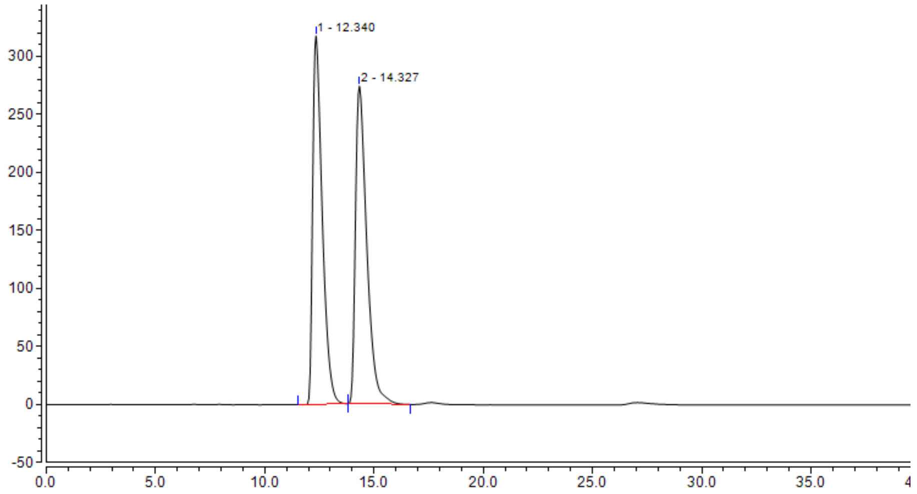

Peak	Ret. Time (min)	Area (mAu*min)	Height (mAu)	Area %	Height %
1	12.340	160.688	317.392	49.45	53.69
2	14.327	164.233	273.727	50.55	46.31

Supplementary Fig. 141. HPLC spectrum of racemic (*P*, *Z*)-3a

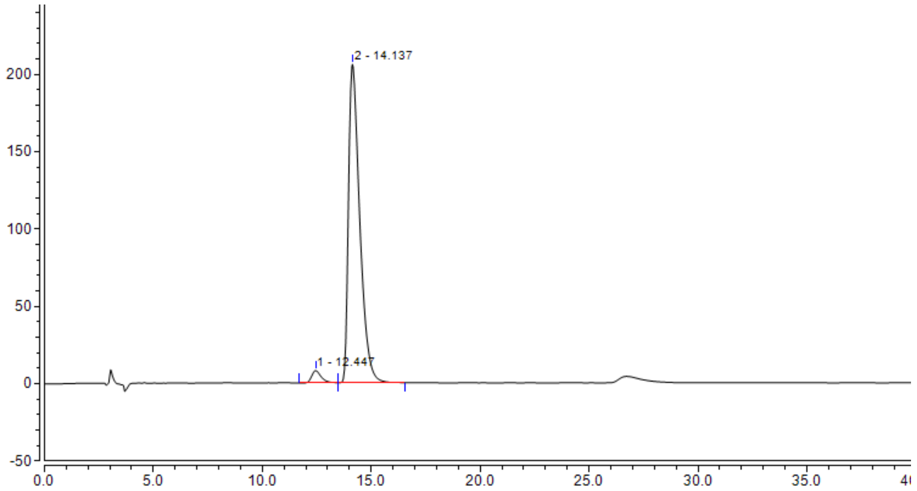

Peak	Ret. Time (min)	Area (mAu*min)	Height (mAu)	Area %	Height %
1	12.447	3.914	7.943	3.08	3.72
2	14.137	123.192	205.858	96.92	96.28

Supplementary Fig. 142. HPLC spectrum of chiral (*P*, *Z*)-3a

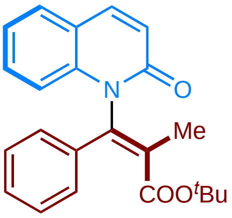

(*P*, *E*)-**3a**

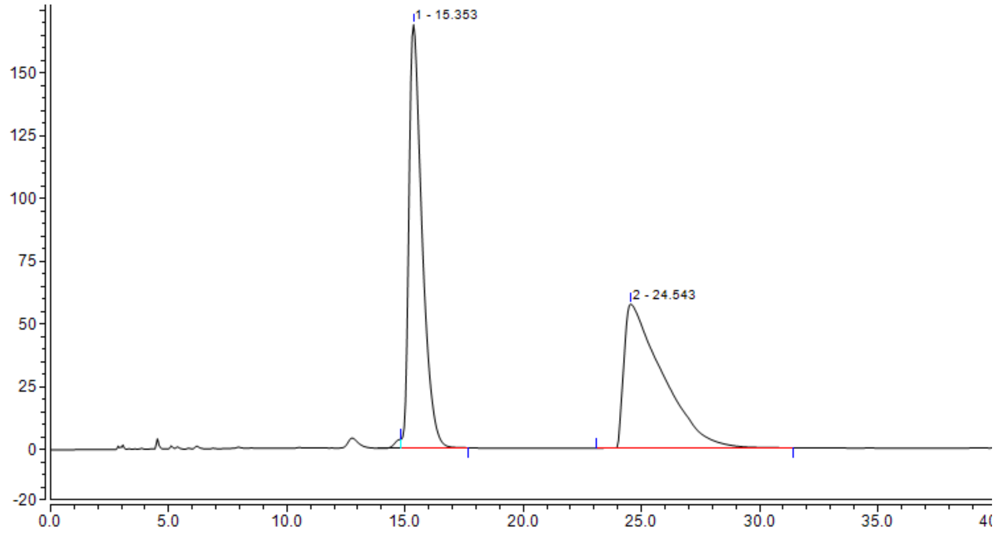

Peak	Ret. Time (min)	Area (mAu*min)	Height (mAu)	Area %	Height %
1	15.353	108.716	168.680	50.03	74.63
2	24.543	108.579	57.338	49.97	25.37

Supplementary Fig. 143. HPLC spectrum of racemic (*P*, *E*)-3a

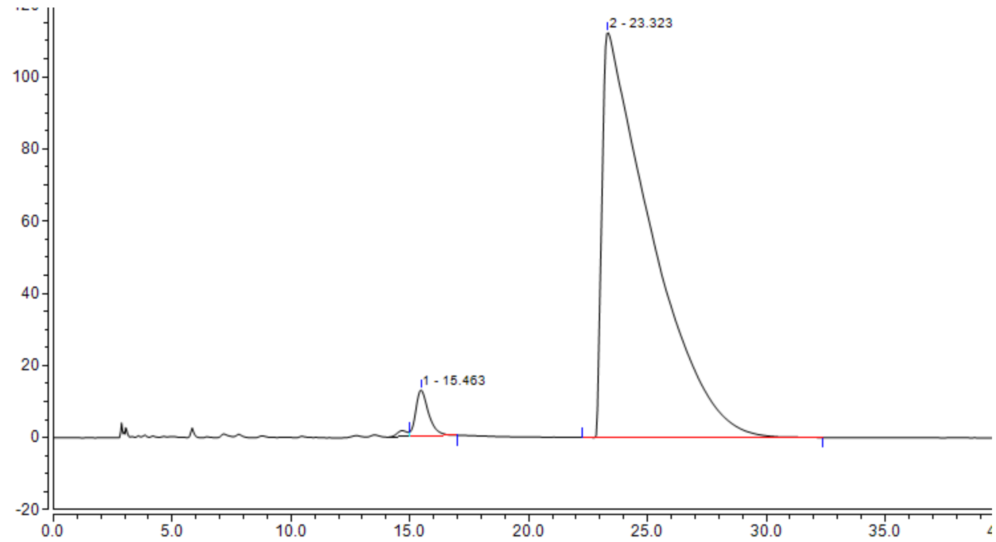

Peak	Ret. Time (min)	Area (mAu*min)	Height (mAu)	Area %	Height %
1	15.463	8.009	12.848	2.88	10.27
2	23.323	270.152	112.303	97.12	89.73

Supplementary Fig. 144. HPLC spectrum of chiral (*P*, *E*)-3a

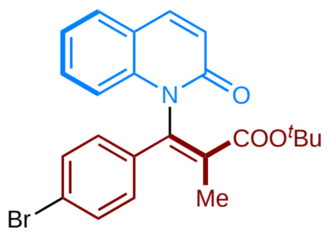

(*P*, *Z*)-**3b**

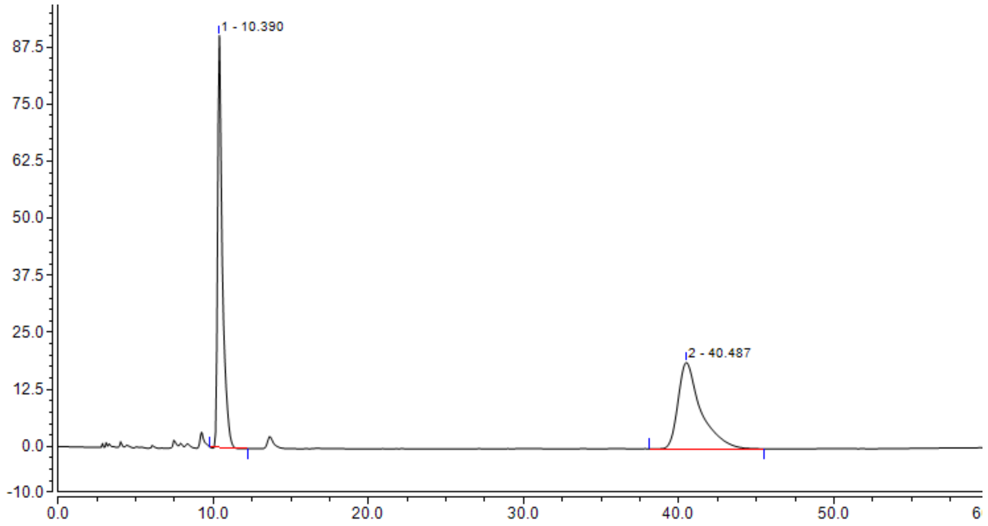

Peak	Ret. Time (min)	Area (mAu*min)	Height (mAu)	Area %	Height %
1	10.390	31.857	90.221	49.81	82.67
2	40.487	32.101	18.914	50.19	17.33

Supplementary Fig. 145. HPLC spectrum of racemic (*P*, *Z*)-3b

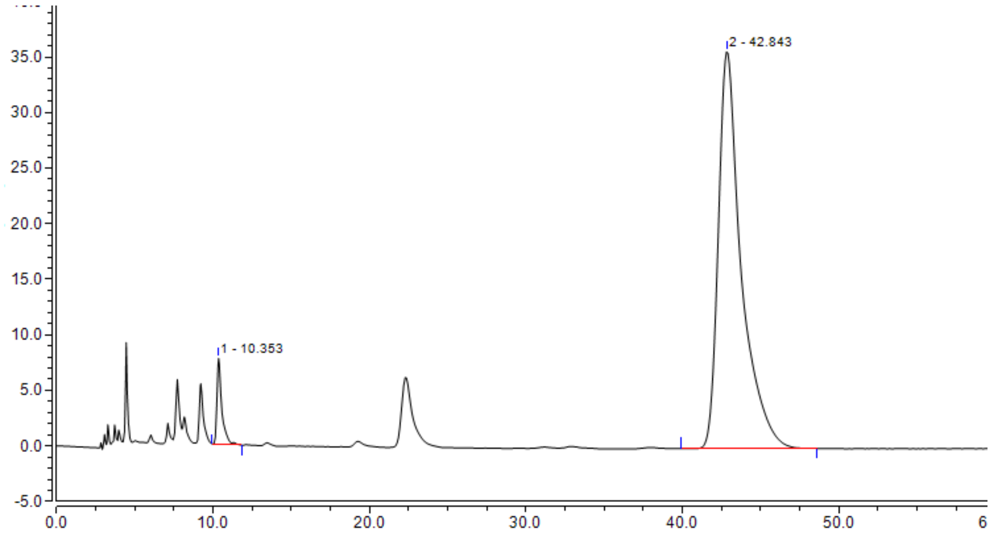

Peak	Ret. Time (min)	Area (mAu*min)	Height (mAu)	Area %	Height %
1	10.353	2.782	7.724	4.39	17.76
2	42.843	60.548	35.760	95.61	82.24

Supplementary Fig. 146. HPLC spectrum of chiral (*P*, *Z*)-3b

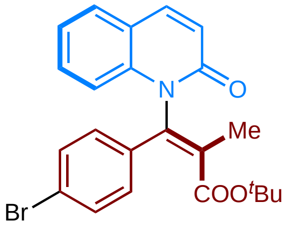

(*P*, *E*)-**3b**

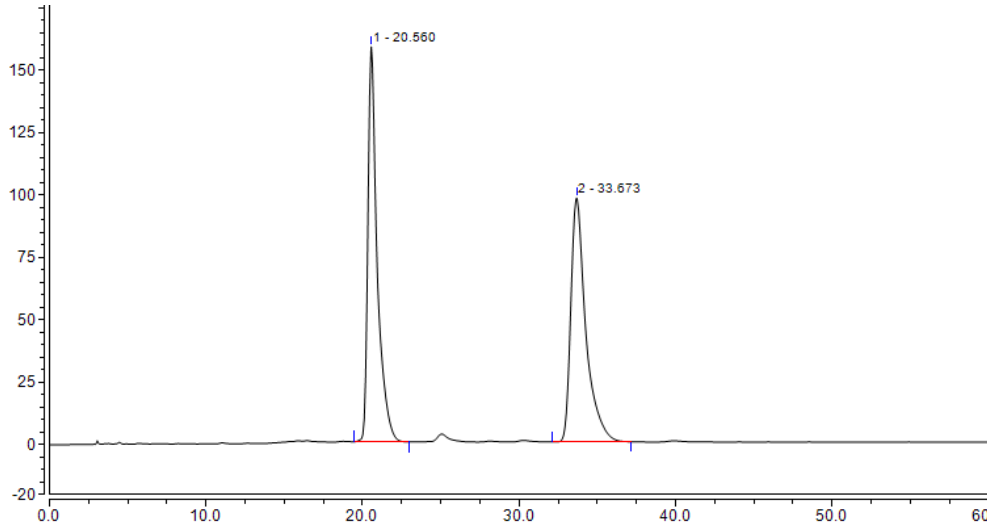

Peak	Ret. Time (min)	Area (mAu*min)	Height (mAu)	Area %	Height %
1	20.560	109.176	158.260	50.22	61.82
2	33.673	108.218	97.750	49.78	38.18

Supplementary Fig. 147. HPLC spectrum of racemic (*P*, *E*)-3b

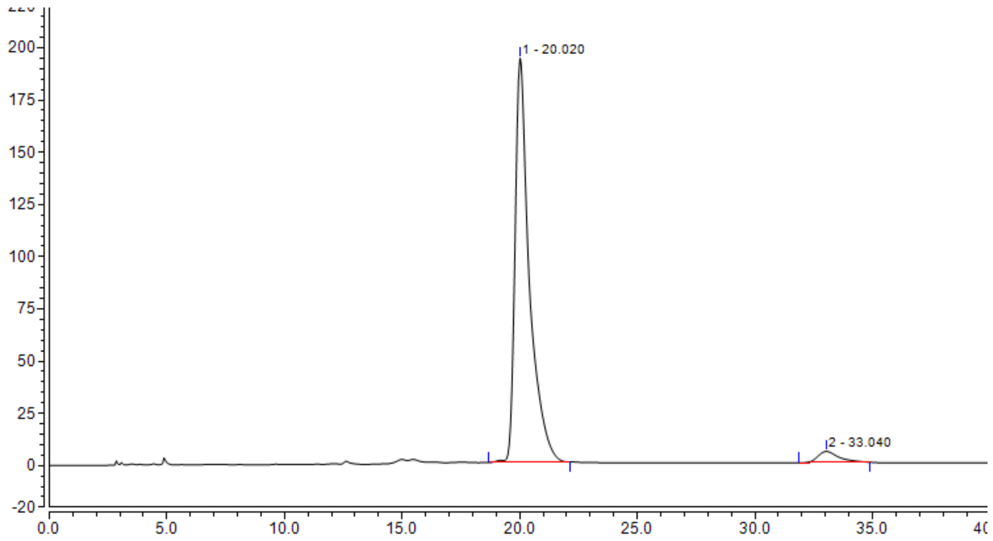

Peak	Ret. Time (min)	Area (mAu*min)	Height (mAu)	Area %	Height %
1	20.020	136.301	193.492	96.06	97.30
2	33.040	5.595	5.369	3.94	2.70

Supplementary Fig. 148. HPLC spectrum of chiral (*P*, *E*)-3b

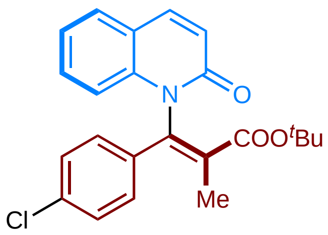

(*P*, *Z*)-**3c**

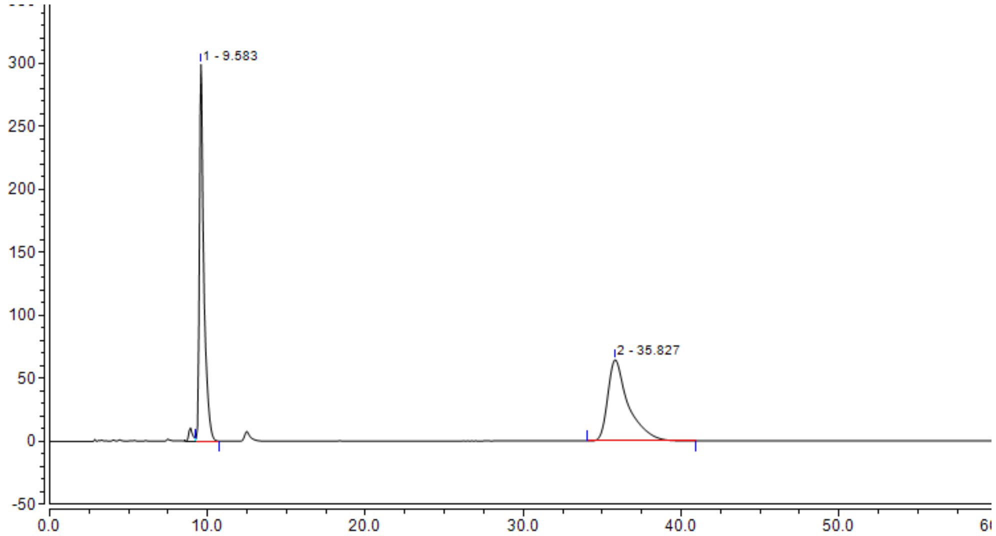

Peak	Ret. Time (min)	Area (mAu*min)	Height (mAu)	Area %	Height %
1	9.583	96.842	298.764	49.98	82.26
2	35.827	96.919	64.435	50.02	17.74

Supplementary Fig. 149. HPLC spectrum of racemic (*P*, *Z*)-3c

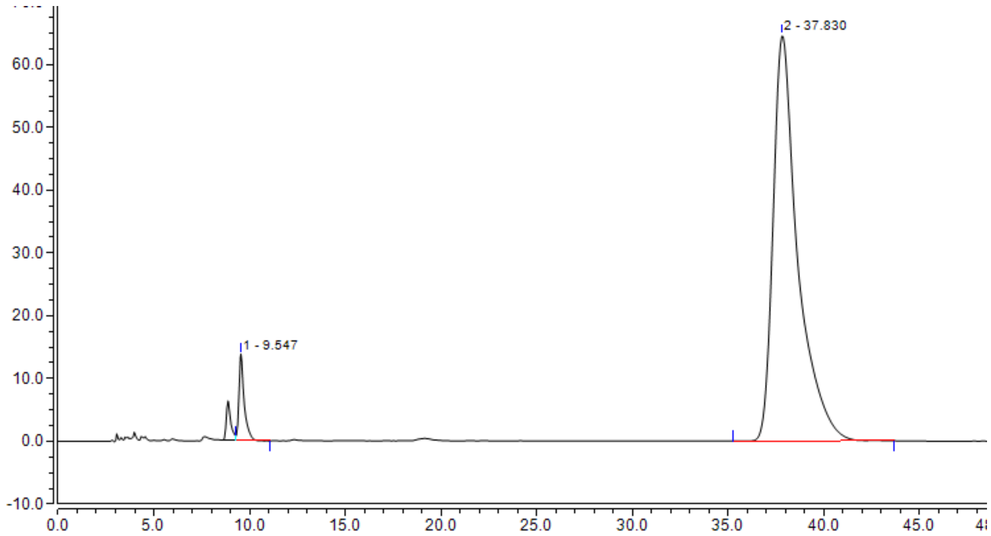

Peak	Ret. Time (min)	Area (mAu*min)	Height (mAu)	Area %	Height %
1	9.547	4.305	13.756	4.28	17.55
2	37.830	96.370	64.624	95.72	82.45

Supplementary Fig. 150. HPLC spectrum of chiral (*P*, *Z*)-3c

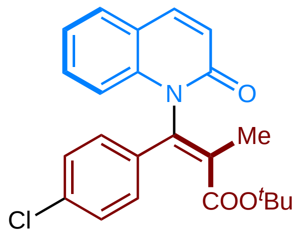

(*P*, *E*)-**3c**

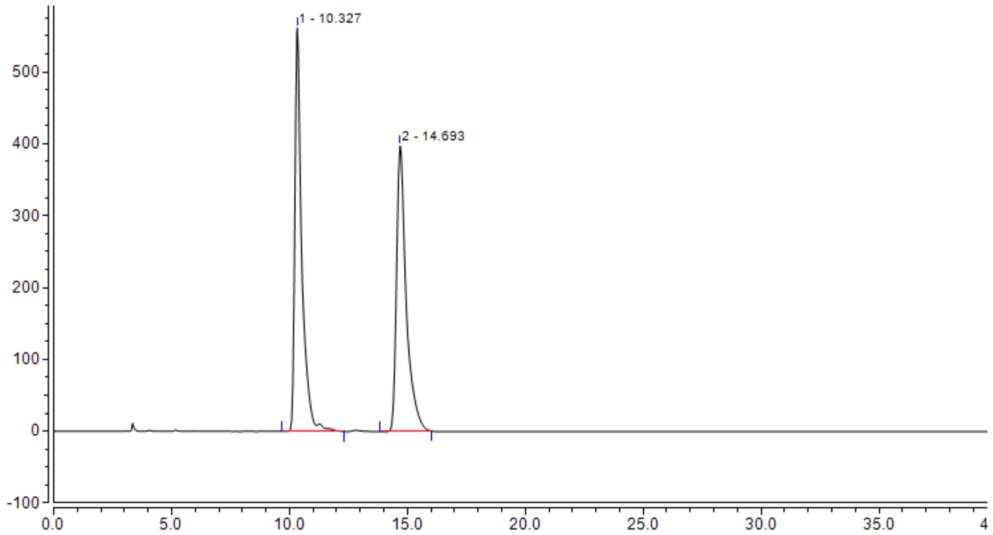

Peak	Ret. Time (min)	Area (mAu*min)	Height (mAu)	Area %	Height %
1	10.327	193.329	561.602	50.60	58.54
2	14.693	188.723	397.677	49.40	41.46

Supplementary Fig. 151. HPLC spectrum of racemic (*P*, *E*)-3c

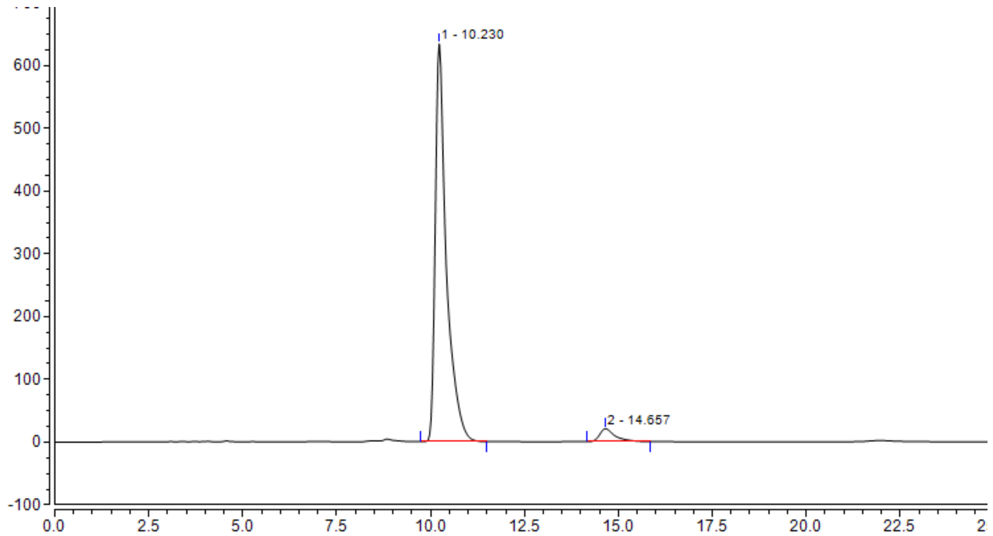

Peak	Ret. Time (min)	Area (mAu*min)	Height (mAu)	Area %	Height %
1	10.230	217.747	633.648	95.73	96.85
2	14.657	9.721	20.641	4.27	3.15

Supplementary Fig. 152. HPLC spectrum of chiral (*P*, *E*)-3c

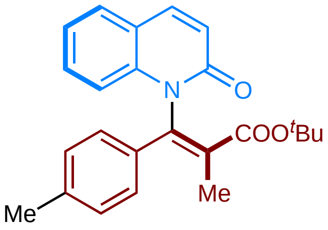

(*P*, *Z*)-**3d**

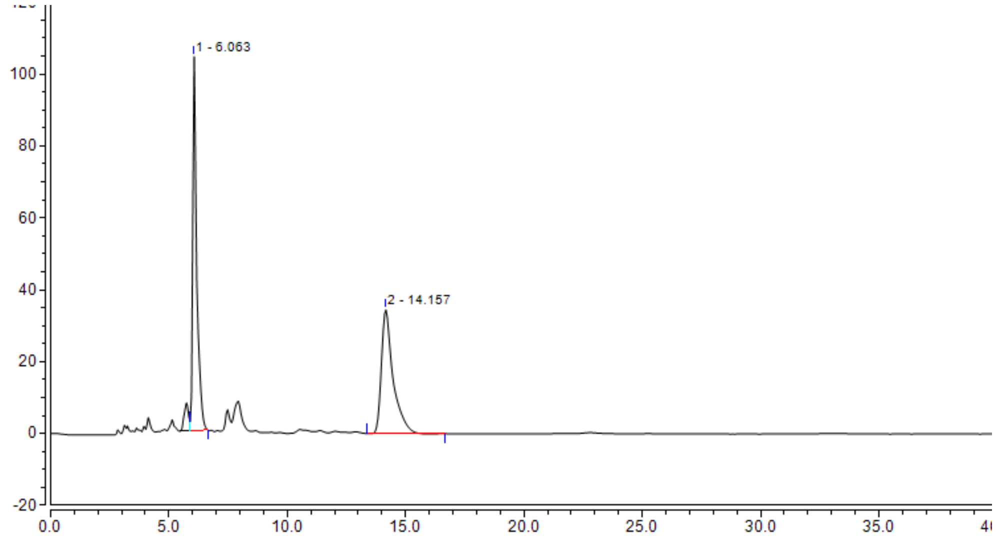

Peak	Ret. Time (min)	Area (mAu*min)	Height (mAu)	Area %	Height %
1	6.063	20.215	104.082	50.67	75.10
2	14.157	19.681	34.515	49.33	24.90

Supplementary Fig. 153. HPLC spectrum of racemic (*P*, *Z*)-3d

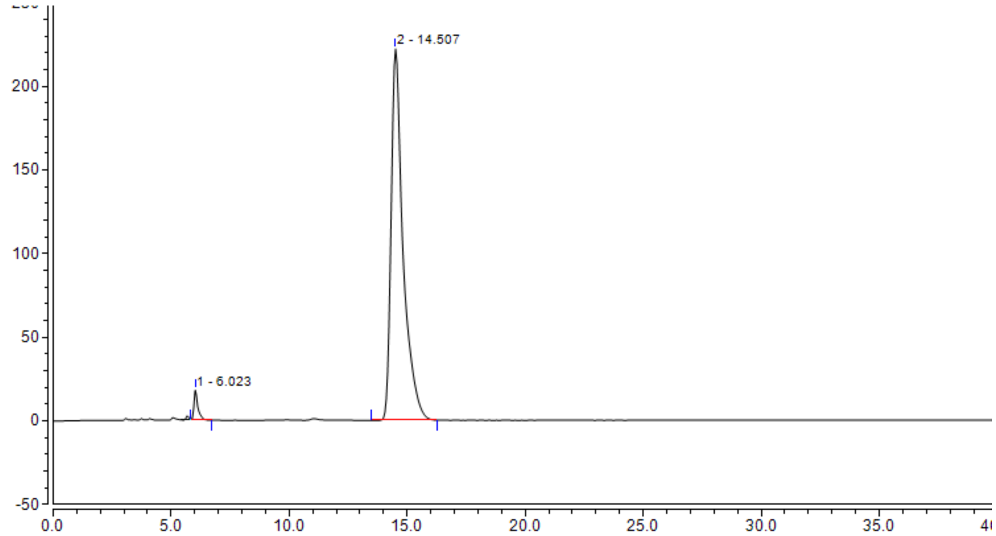

Peak	Ret. Time (min)	Area (mAu*min)	Height (mAu)	Area %	Height %
1	6.023	3.394	17.871	2.54	7.45
2	14.507	130.425	221.990	97.46	92.55

Supplementary Fig. 154. HPLC spectrum of chiral (*P*, *Z*)-3d

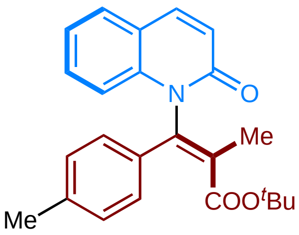

(*P*, *E*)-**3d**

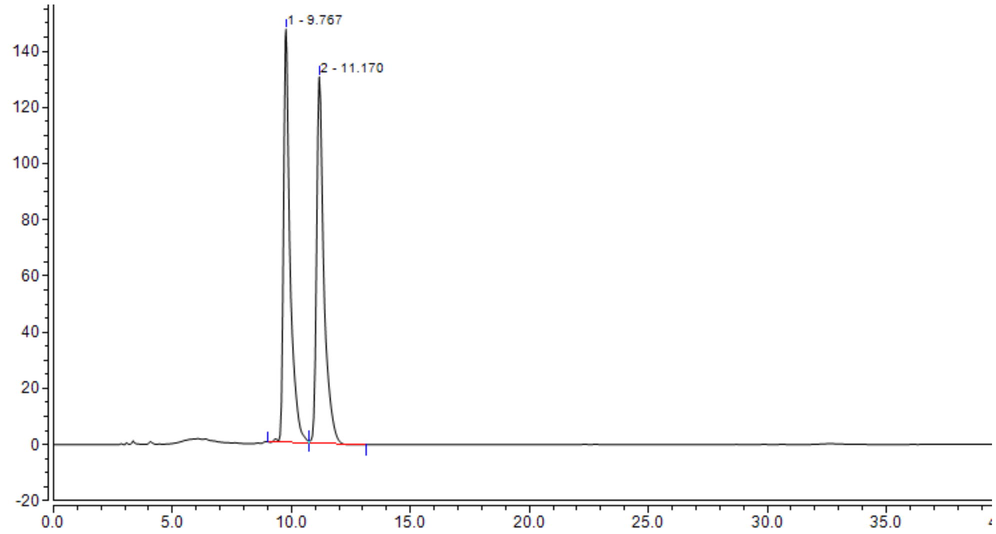

Peak	Ret. Time (min)	Area (mAu*min)	Height (mAu)	Area %	Height %
1	9.767	46.787	147.038	50.22	52.99
2	11.170	46.386	130.427	49.78	47.01

Supplementary Fig. 155. HPLC spectrum of racemic (*P*, *E*)-3d

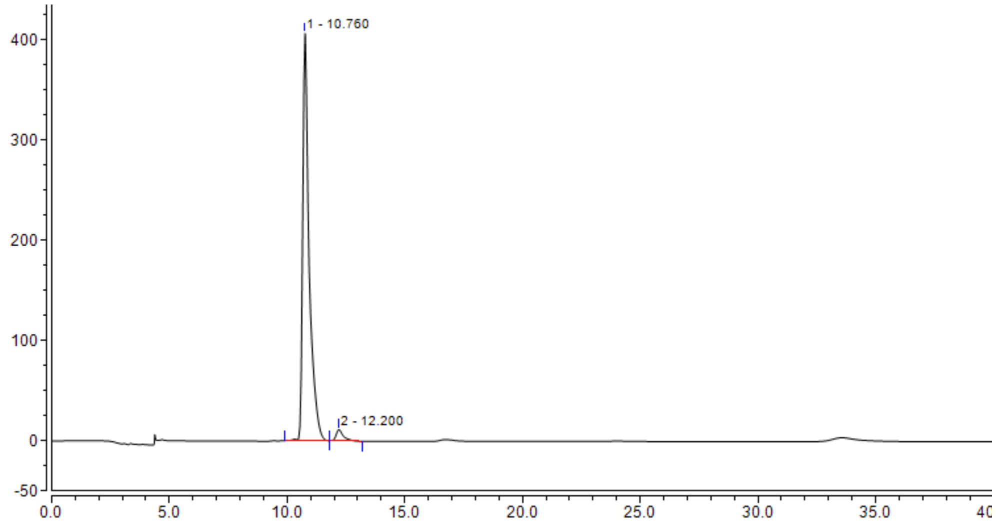

Peak	Ret. Time (min)	Area (mAu*min)	Height (mAu)	Area %	Height %
1	10.760	128.295	406.163	97.04	97.31
2	12.200	3.915	11.210	2.96	2.69

Supplementary Fig. 156. HPLC spectrum of chiral (*P*, *E*)-3d

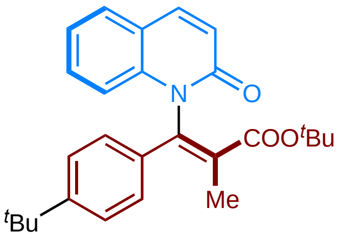

(*P*, *Z*)-**3e**

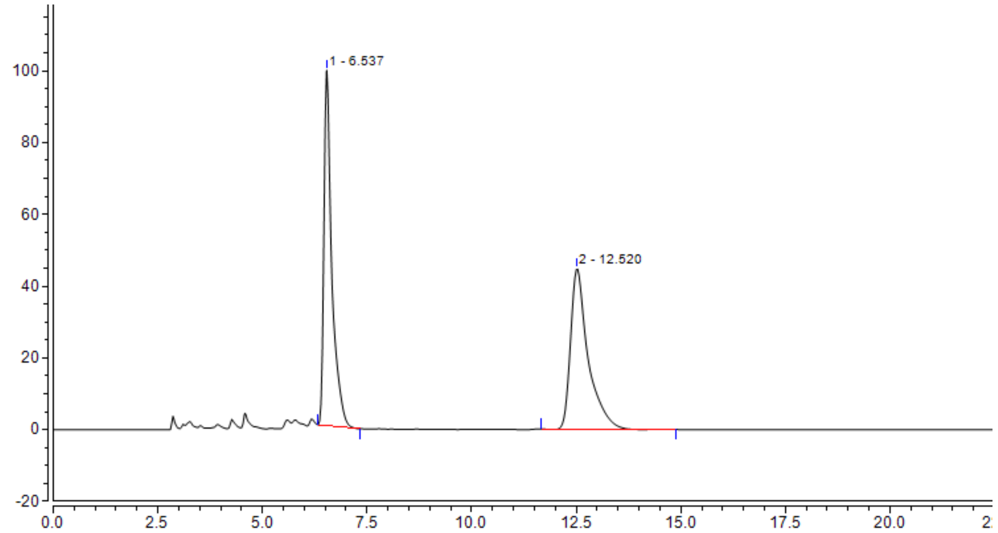

Peak	Ret. Time (min)	Area (mAu*min)	Height (mAu)	Area %	Height %
1	6.537	21.592	99.062	50.00	68.87
2	12.520	21.594	44.777	50.00	31.13

Supplementary Fig. 157. HPLC spectrum of racemic (*P*, *Z*)-3e

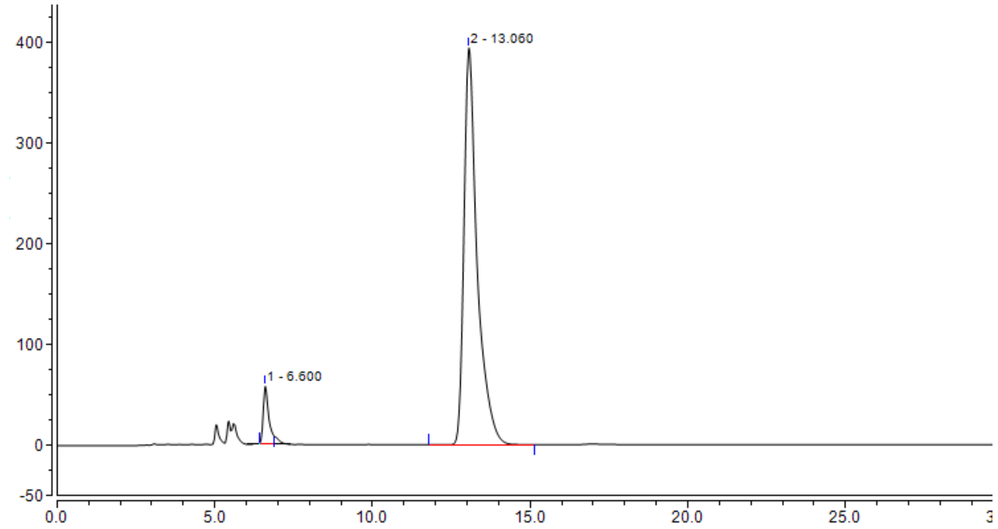

Peak	Ret. Time (min)	Area (mAu*min)	Height (mAu)	Area %	Height %
1	6.600	11.740	57.628	5.78	12.76
2	13.060	191.459	393.943	94.22	87.24

Supplementary Fig. 158. HPLC spectrum of chiral (*P*, *Z*)-3e

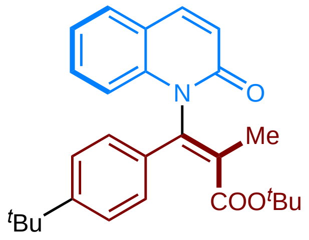

(*P*, *E*)-**3e**

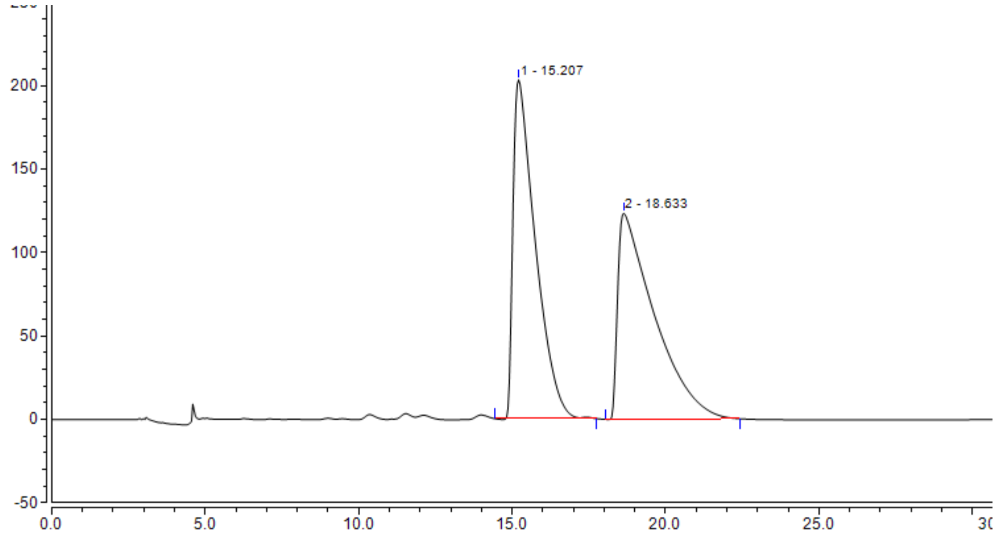

Peak	Ret. Time (min)	Area (mAu*min)	Height (mAu)	Area %	Height %
1	15.207	170.897	203.136	50.04	62.20
2	18.633	170.605	123.447	49.96	37.80

Supplementary Fig. 159. HPLC spectrum of racemic (*P*, *E*)-3e

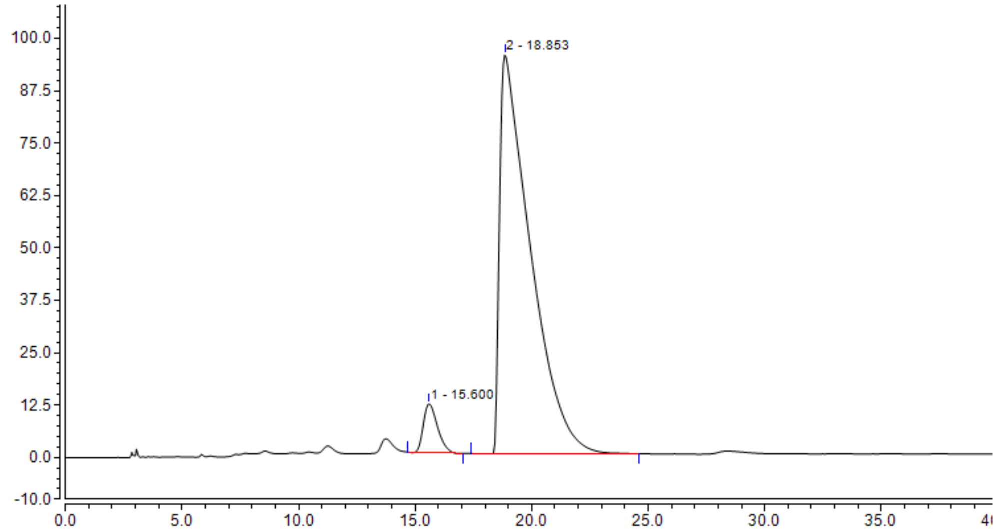

Peak	Ret. Time (min)	Area (mAu*min)	Height (mAu)	Area %	Height %
1	15.600	8.122	11.628	5.29	10.89
2	18.853	145.504	95.132	94.71	89.11

Supplementary Fig. 160. HPLC spectrum of chiral (*P*, *E*)-3e

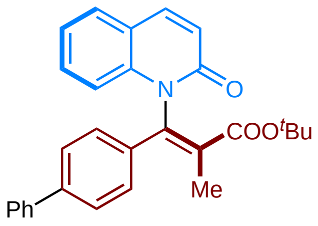

(*P*, *Z*)-**3f**

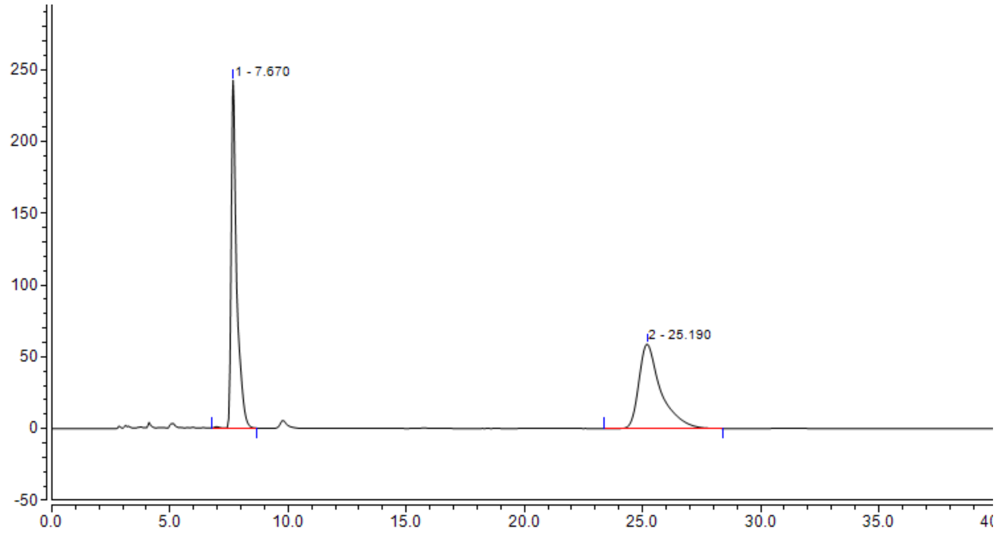

Peak	Ret. Time (min)	Area (mAu*min)	Height (mAu)	Area %	Height %
1	7.670	65.061	242.222	50.76	80.47
2	25.190	63.106	58.781	49.24	19.53

Supplementary Fig. 161. HPLC spectrum of racemic (*P*, *Z*)-3f

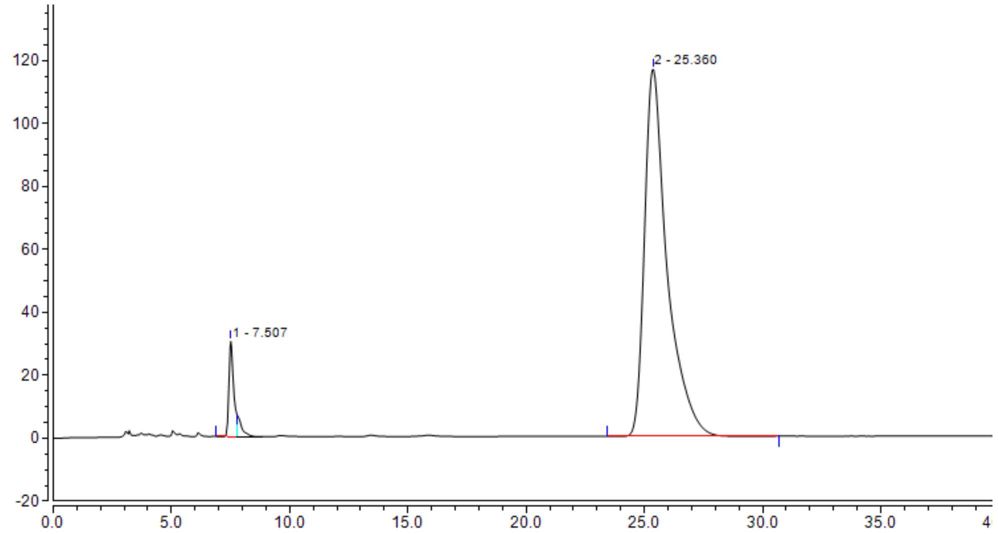

Peak	Ret. Time (min)	Area (mAu*min)	Height (mAu)	Area %	Height %
1	7.507	6.919	30.256	5.08	20.57
2	25.360	129.183	116.838	94.92	79.43

Supplementary Fig. 162. HPLC spectrum of chiral (*P*, *Z*)-3f

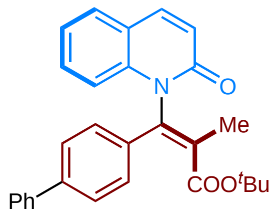

(P, E)-**3f**

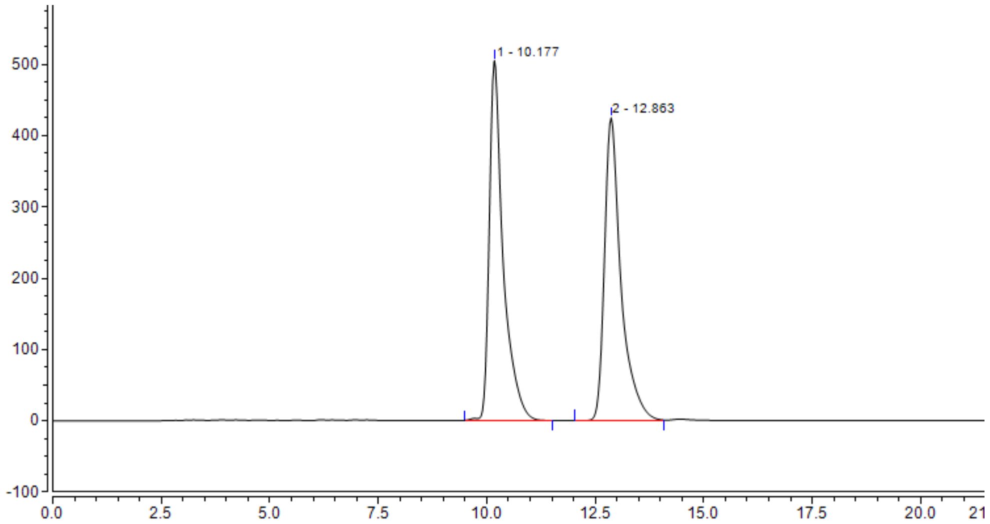

Peak	Ret. Time (min)	Area (mAu*min)	Height (mAu)	Area %	Height %
1	10.177	186.967	504.843	50.04	54.30
2	12.863	186.702	424.808	49.96	45.70

Supplementary Fig. 163. HPLC spectrum of racemic (*P*, *E*)-3f

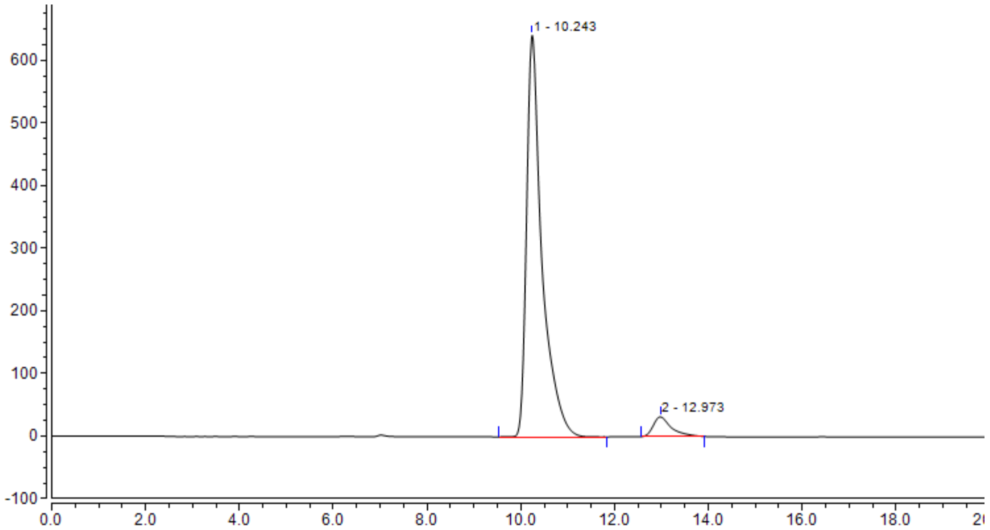

Peak	Ret. Time (min)	Area (mAu*min)	Height (mAu)	Area %	Height %
1	10.243	243.726	641.582	94.49	95.30
2	12.973	14.209	31.643	5.51	4.70

Supplementary Fig. 164. HPLC spectrum of chiral (*P*, *E*)-3f

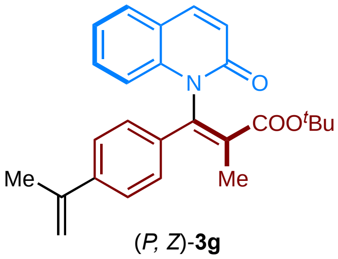

(*P*, *Z*)-**3g**

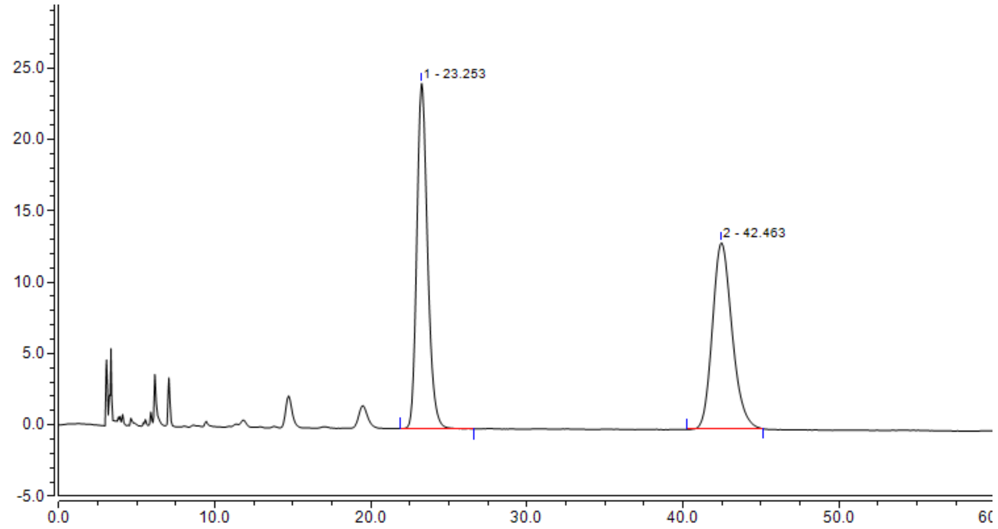

Peak	Ret. Time (min)	Area (mAu*min)	Height (mAu)	Area %	Height %
1	23.253	19.276	24.189	50.32	64.95
2	42.463	19.031	13.054	49.68	35.05

Supplementary Fig. 165. HPLC spectrum of racemic (*P*, *Z*)-3g

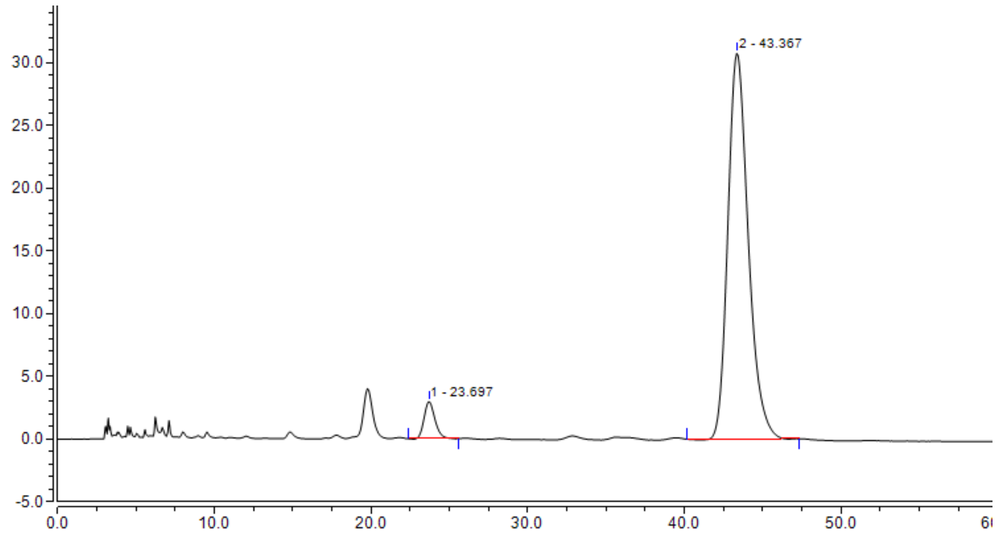

Peak	Ret. Time (min)	Area (mAu*min)	Height (mAu)	Area %	Height %
1	23.697	2.346	2.964	4.82	8.79
2	43.367	46.288	30.761	95.18	91.21

Supplementary Fig. 166. HPLC spectrum of chiral (*P*, *Z*)-3g

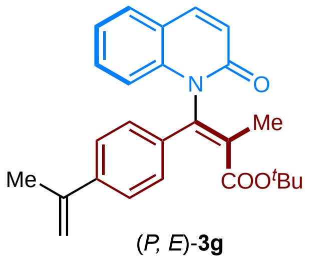

(*P, E*)-**3g**

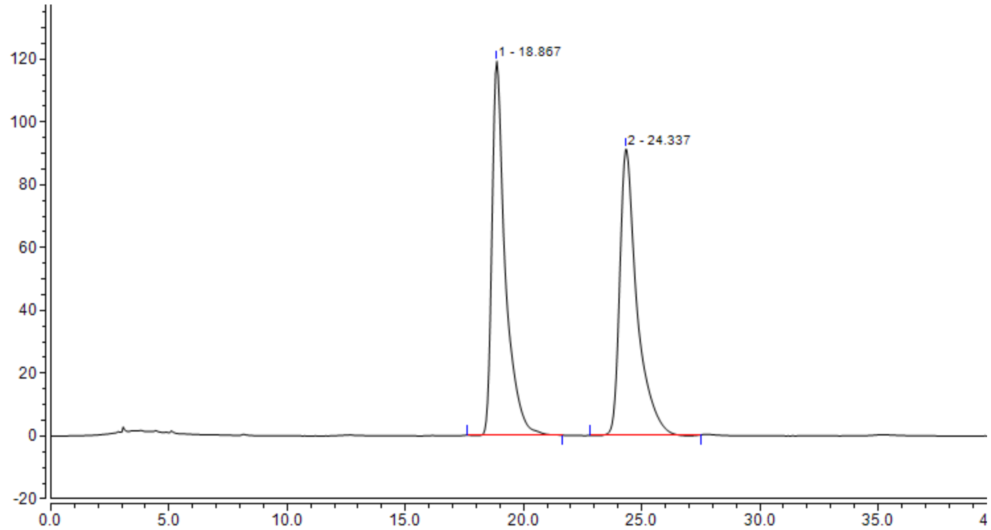

Peak	Ret. Time (min)	Area (mAu*min)	Height (mAu)	Area %	Height %
1	18.867	77.771	119.158	50.39	56.65
2	24.337	76.571	91.176	49.61	43.35

Supplementary Fig. 167. HPLC spectrum of racemic (*P, E*)-3g

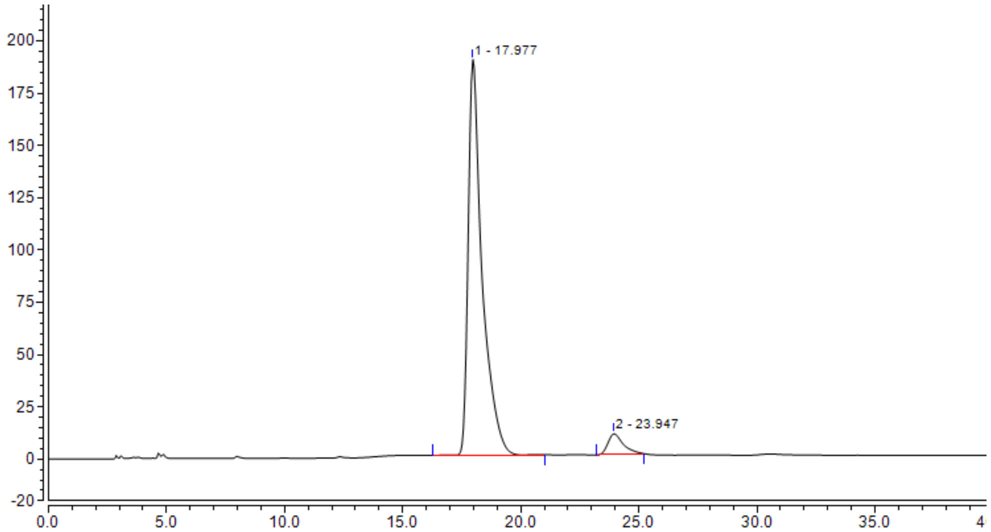

Peak	Ret. Time (min)	Area (mAu*min)	Height (mAu)	Area %	Height %
1	17.977	129.636	189.248	94.53	95.02
2	23.947	7.502	9.918	5.47	4.98

Supplementary Fig. 168. HPLC spectrum of chiral (*P, E*)-3g

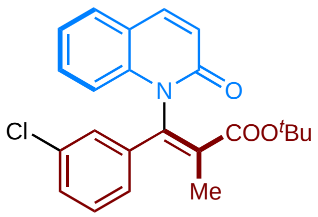

(*P*, *Z*)-**3h**

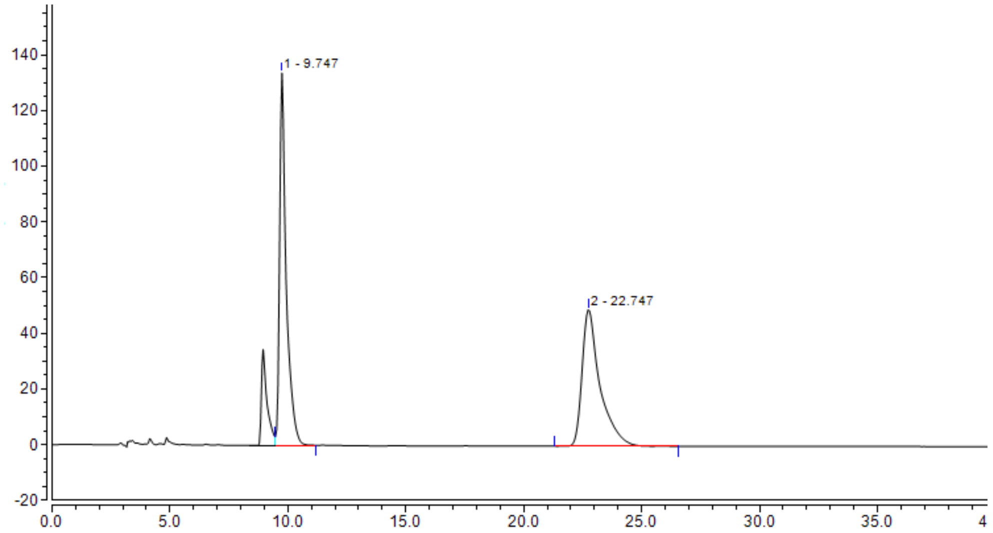

Peak	Ret. Time (min)	Area (mAu*min)	Height (mAu)	Area %	Height %
1	9.747	43.739	133.712	50.45	73.14
2	22.747	42.962	49.093	49.55	26.86

Supplementary Fig. 169. HPLC spectrum of racemic (*P*, *Z*)-3h

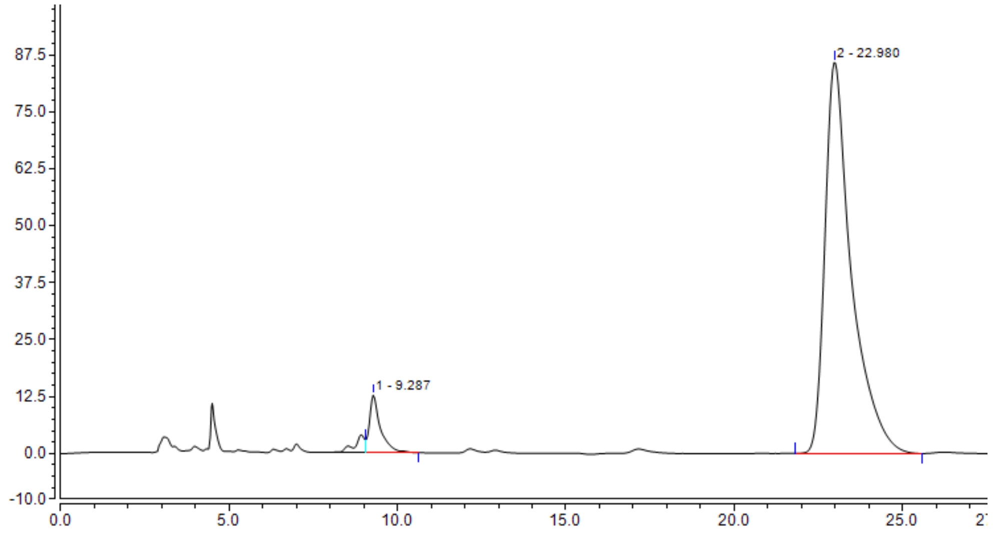

Peak	Ret. Time (min)	Area (mAu*min)	Height (mAu)	Area %	Height %
1	9.287	4.718	12.586	5.69	12.78
2	22.980	78.267	85.904	94.31	87.22

Supplementary Fig. 170. HPLC spectrum of chiral (*P*, *Z*)-3h

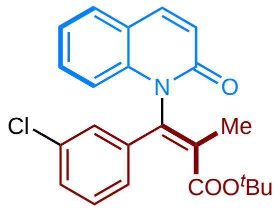

(*P*, *E*)-**3h**

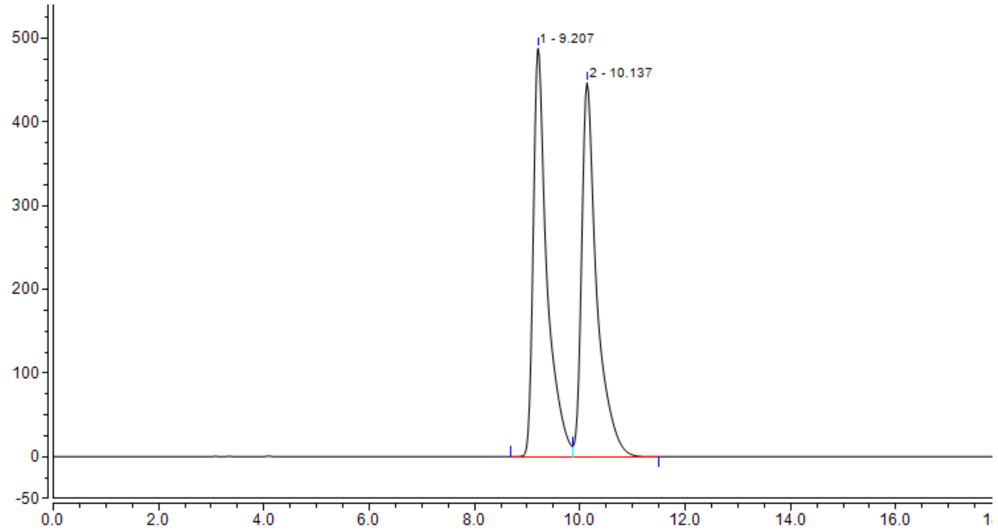

Peak	Ret. Time (min)	Area (mAu*min)	Height (mAu)	Area %	Height %
1	9.207	147.705	487.987	49.81	52.18
2	10.137	148.807	447.199	50.19	47.82

Supplementary Fig. 171. HPLC spectrum of racemic (*P*, *E*)-3h

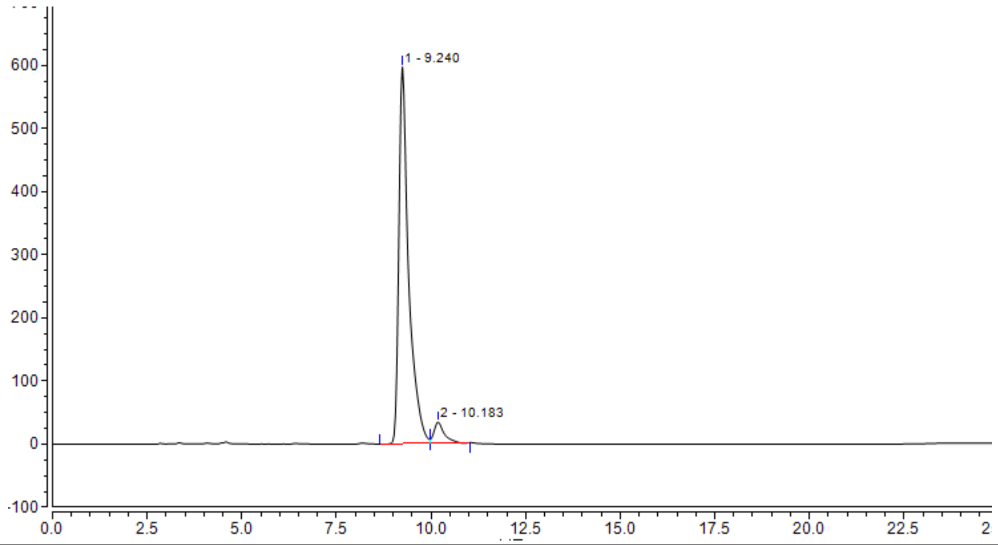

Peak	Ret. Time (min)	Area (mAu*min)	Height (mAu)	Area %	Height %
1	9.240	179.460	596.965	94.58	94.70
2	10.183	10.284	33.430	5.42	5.30

Supplementary Fig. 172. HPLC spectrum of chiral (*P*, *E*)-3h

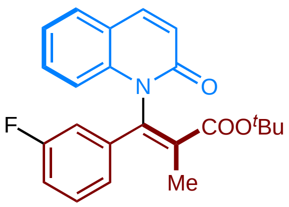

(*P*, *Z*)-**3i**

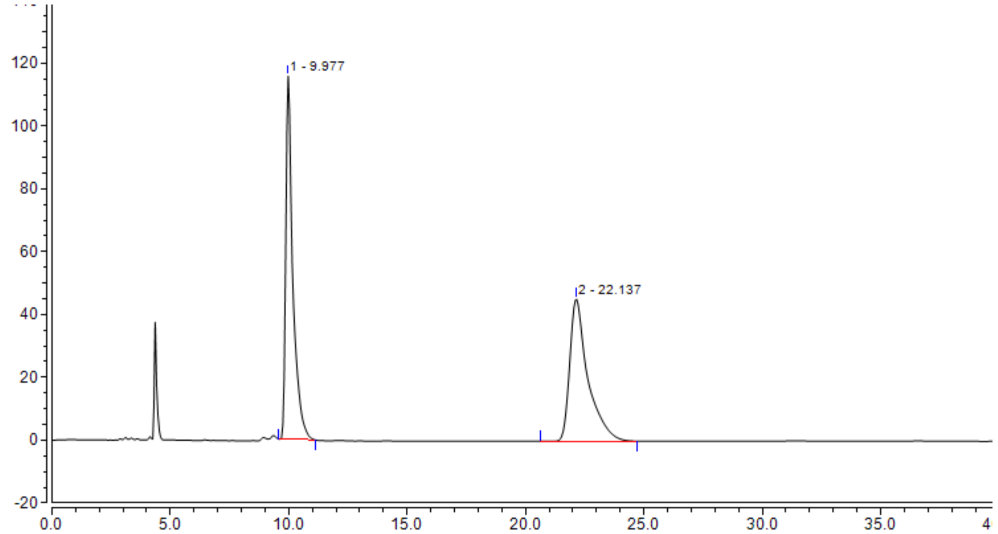

Peak	Ret. Time (min)	Area (mAu*min)	Height (mAu)	Area %	Height %
1	9.977	39.974	115.633	49.96	71.84
2	22.137	40.034	45.330	50.04	28.16

Supplementary Fig. 173. HPLC spectrum of racemic (*P*, *Z*)-3i

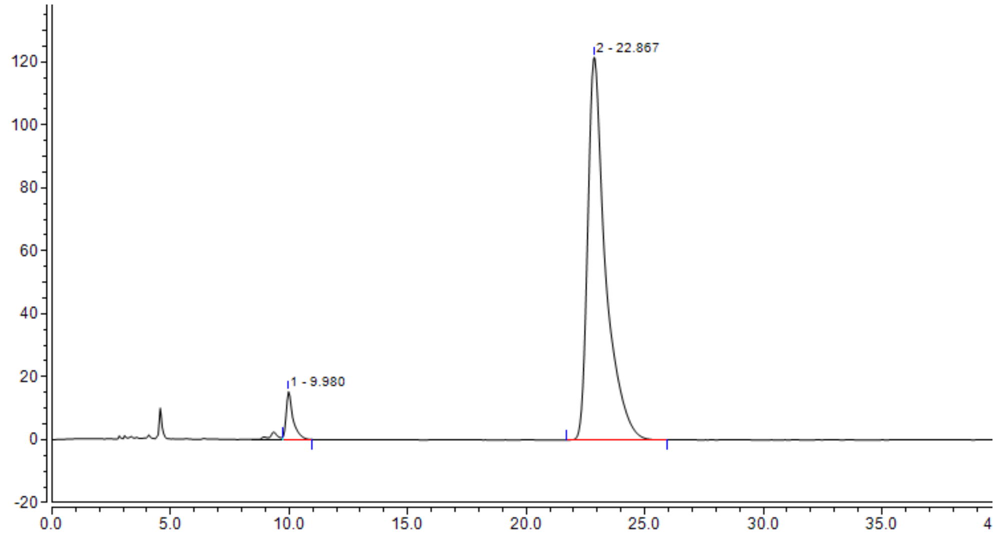

Peak	Ret. Time (min)	Area (mAu*min)	Height (mAu)	Area %	Height %
1	9.980	5.114	15.286	4.62	11.17
2	22.867	105.515	121.589	95.38	88.83

Supplementary Fig. 174. HPLC spectrum of chiral (*P*, *Z*)-3i

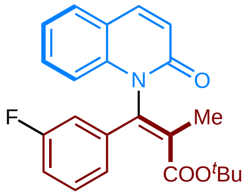

(*P*, *E*)-**3i**

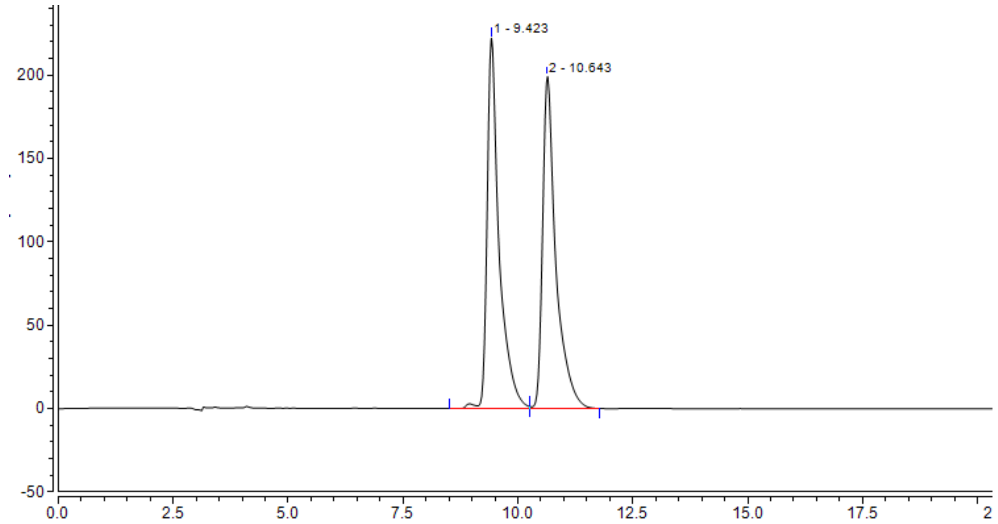

Peak	Ret. Time (min)	Area (mAu*min)	Height (mAu)	Area %	Height %
1	9.423	67.315	222.136	50.25	52.74
2	10.643	66.634	199.079	49.75	47.26

Supplementary Fig. 175. HPLC spectrum of racemic (*P*, *E*)-3i

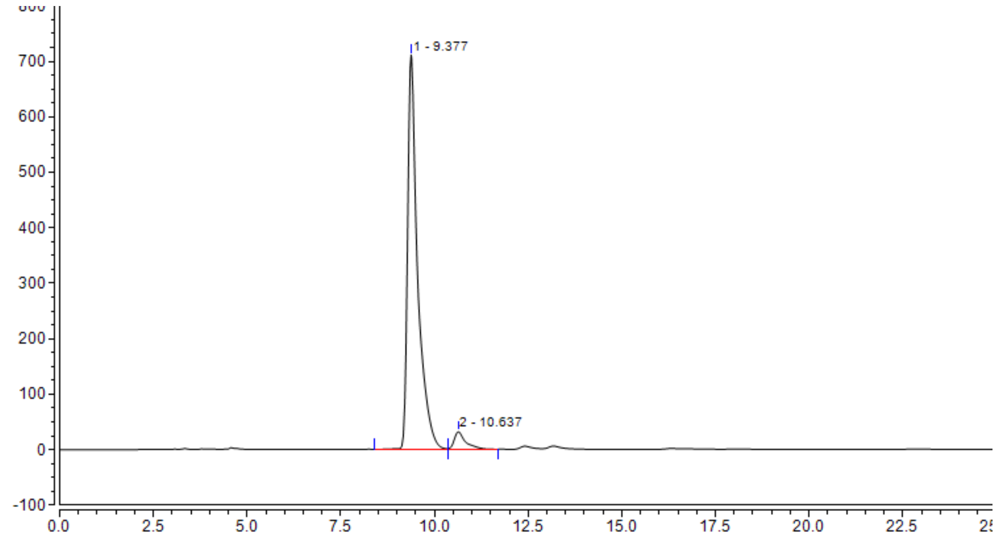

Peak	Ret. Time (min)	Area (mAu*min)	Height (mAu)	Area %	Height %
1	9.377	221.284	710.755	95.17	95.72
2	10.637	11.219	31.813	4.83	4.28

Supplementary Fig. 176. HPLC spectrum of chiral (*P*, *E*)-3i

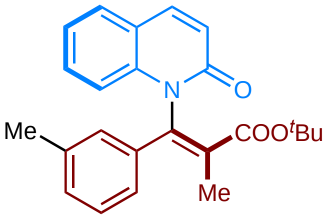

(*P*, *Z*)-**3j**

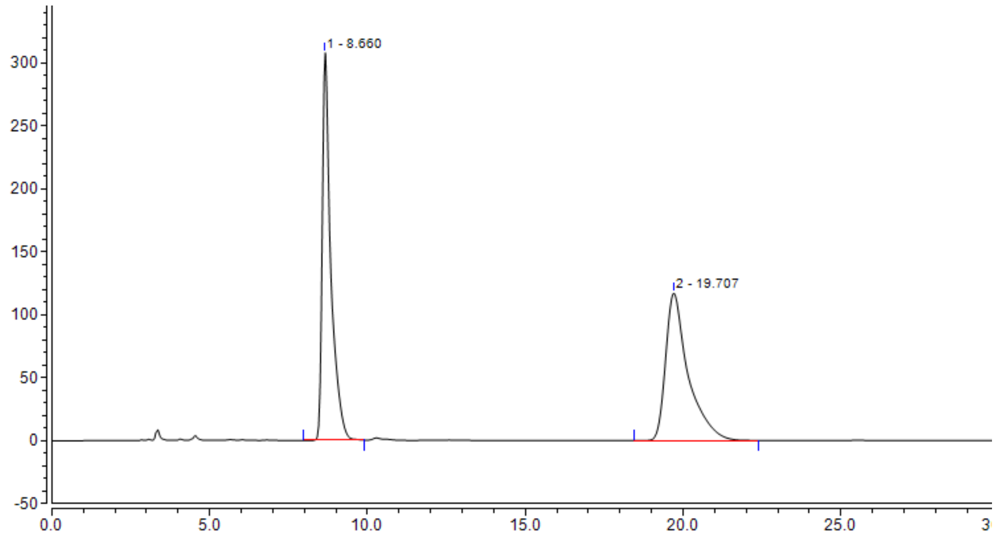

Peak	Ret. Time (min)	Area (mAu*min)	Height (mAu)	Area %	Height %
1	8.660	97.210	307.736	50.17	72.45
2	19.707	96.540	116.996	49.83	27.55

Supplementary Fig. 177. HPLC spectrum of racemic (*P*, *Z*)-3j

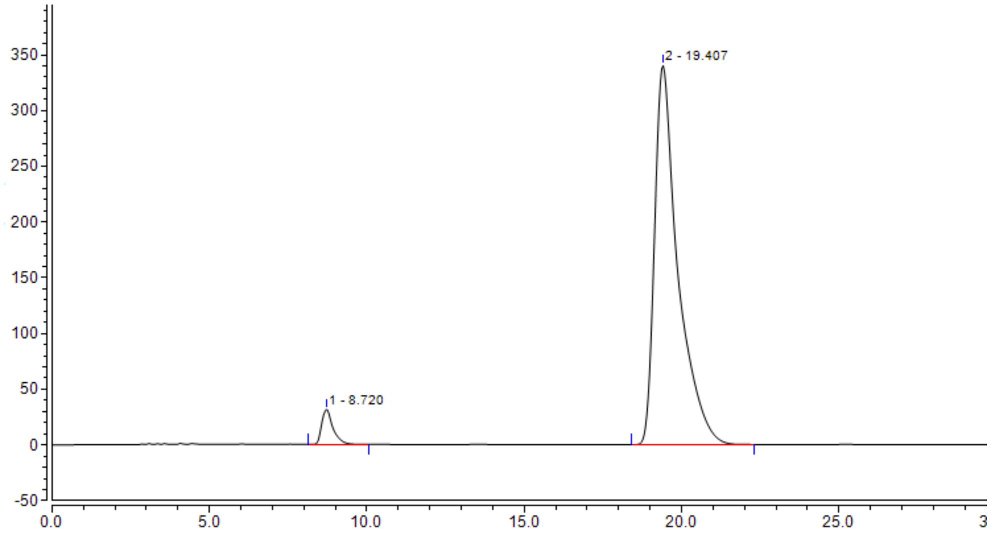

Peak	Ret. Time (min)	Area (mAu*min)	Height (mAu)	Area %	Height %
1	8.720	13.074	31.467	4.28	8.47
2	19.407	292.194	340.148	95.72	91.53

Supplementary Fig. 178. HPLC spectrum of chiral (*P*, *Z*)-3j

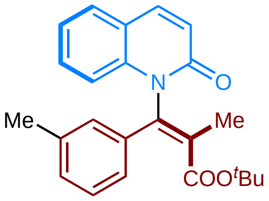

(*P*, *E*)-**3j**

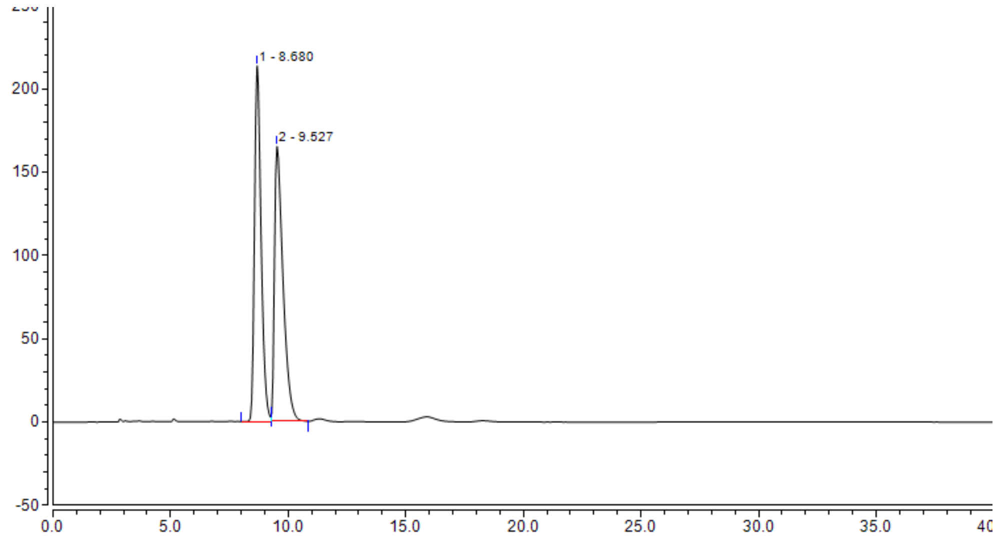

Peak	Ret. Time (min)	Area (mAu*min)	Height (mAu)	Area %	Height %
1	8.680	69.288	213.562	49.85	56.40
2	9.527	69.712	165.115	50.15	43.60

Supplementary Fig. 179. HPLC spectrum of racemic (*P*, *E*)-3j

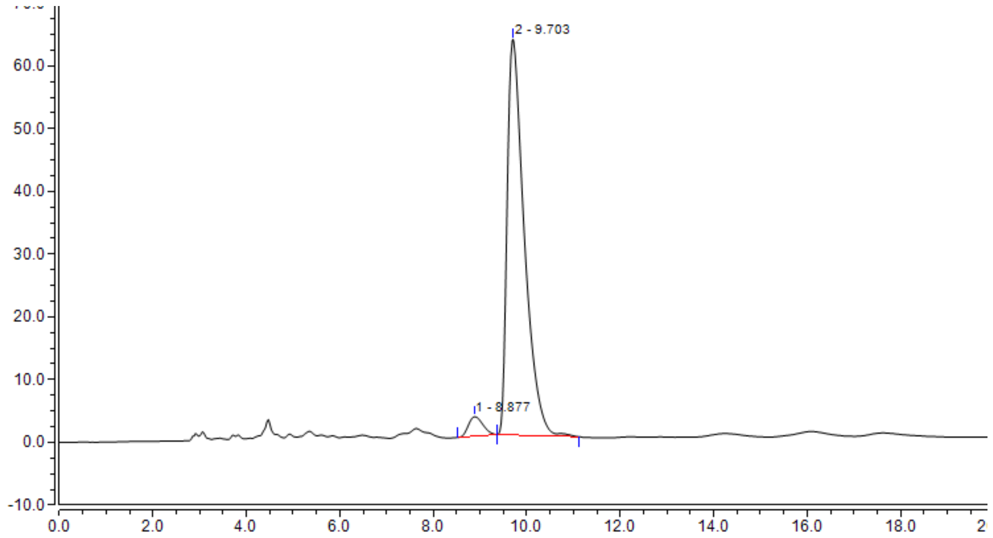

Peak	Ret. Time (min)	Area (mAu*min)	Height (mAu)	Area %	Height %
1	8.877	1.128	3.121	4.04	4.71
2	9.703	26.836	63.109	95.96	95.29

Supplementary Fig. 180. HPLC spectrum of chiral (*P*, *E*)-3j

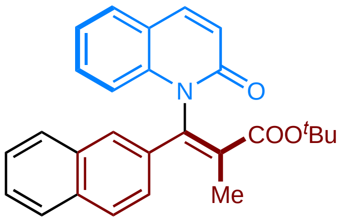

(*P*, *Z*)-**3k**

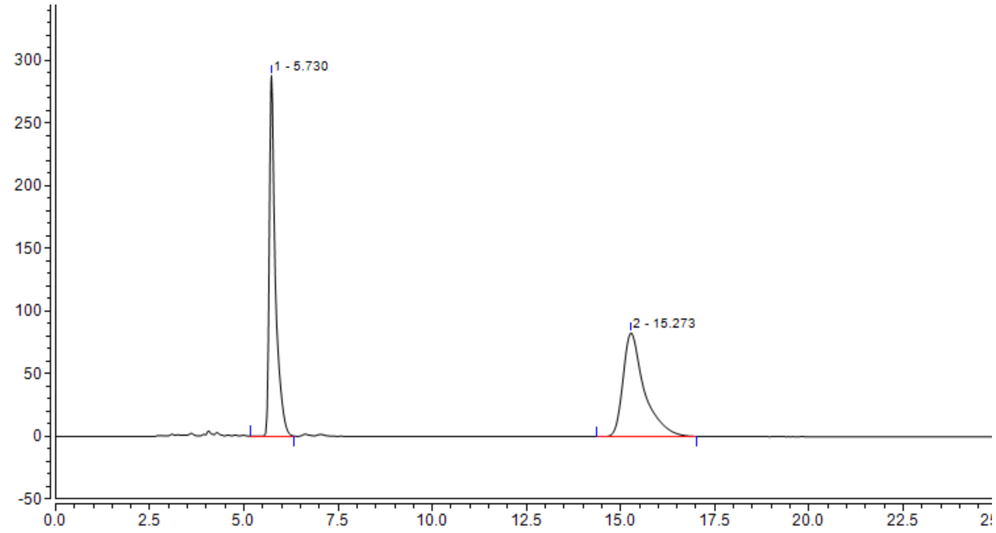

Peak	Ret. Time (min)	Area (mAu*min)	Height (mAu)	Area %	Height %
1	5.730	54.082	287.847	50.05	77.68
2	15.273	53.977	82.730	49.95	22.32

Supplementary Fig. 181. HPLC spectrum of racemic (*P*, *Z*)-3k

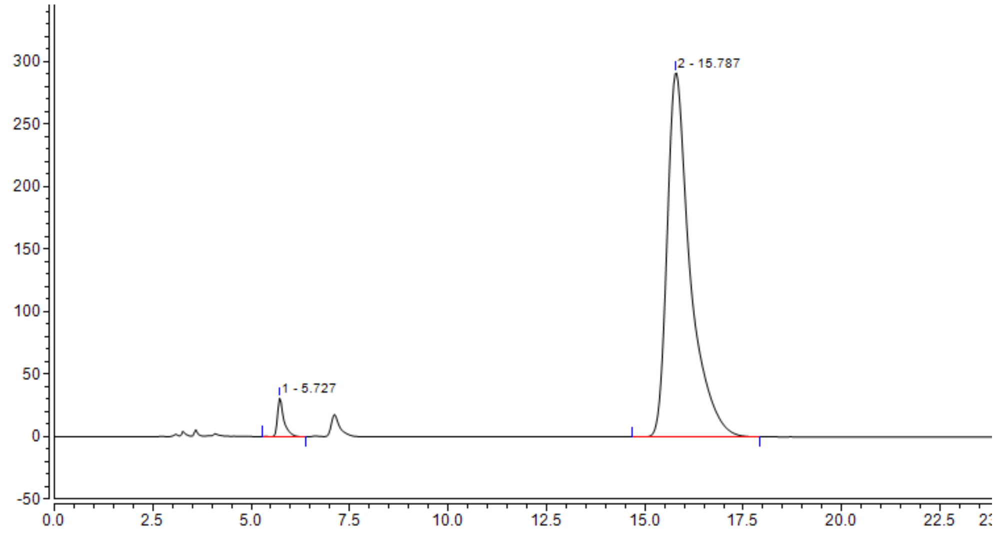

Peak	Ret. Time (min)	Area (mAu*min)	Height (mAu)	Area %	Height %
1	5.727	5.781	31.100	2.85	9.63
2	15.787	196.861	291.727	97.15	90.37

Supplementary Fig. 182. HPLC spectrum of chiral (*P*, *Z*)-3k

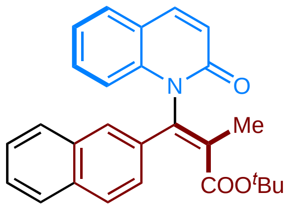

(*P*, *E*)-**3k**

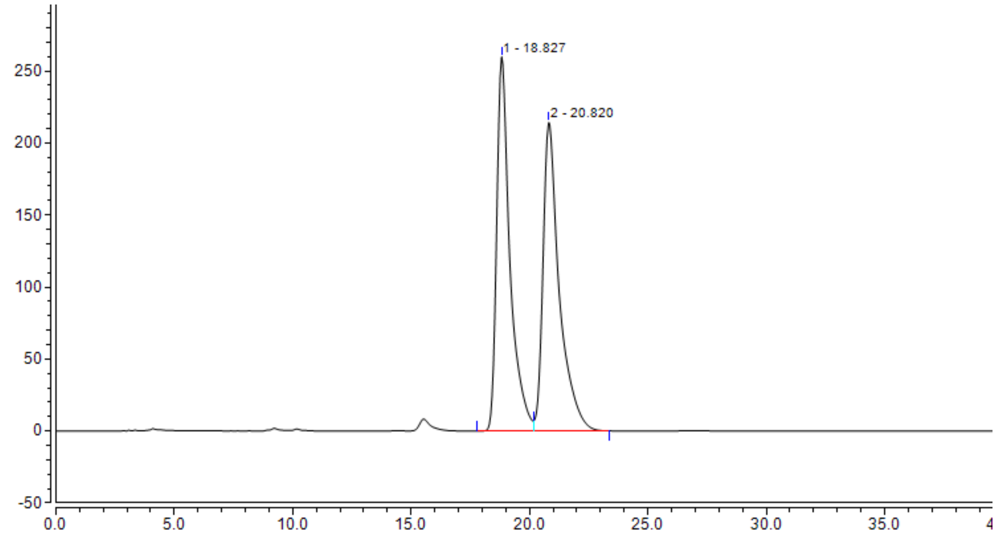

Peak	Ret. Time (min)	Area (mAu*min)	Height (mAu)	Area %	Height %
1	18.827	168.727	259.878	49.72	54.80
2	20.820	170.626	214.337	50.28	45.20

Supplementary Fig. 183. HPLC spectrum of racemic (*P*, *E*)-3k

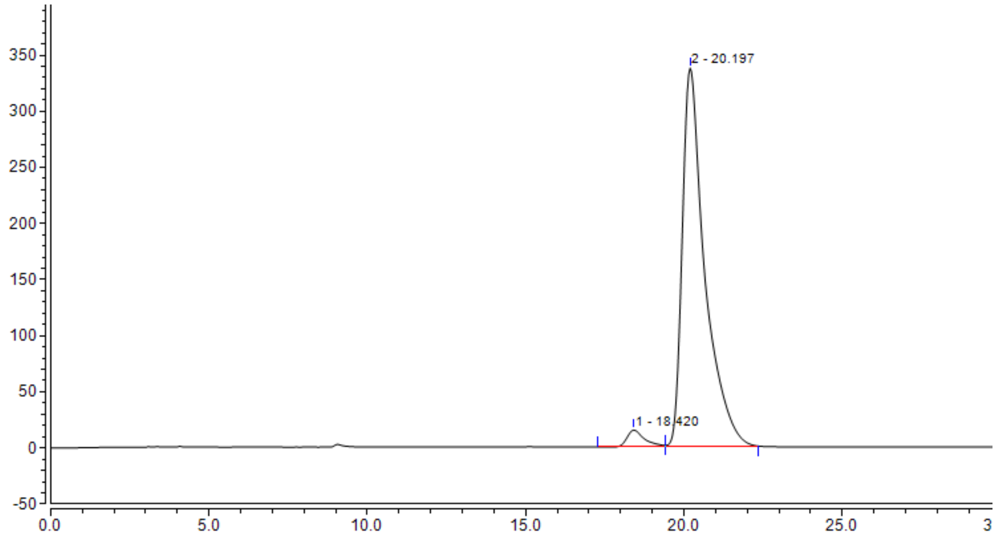

Peak	Ret. Time (min)	Area (mAu*min)	Height (mAu)	Area %	Height %
1	18.420	9.492	14.925	3.31	4.24
2	20.197	277.630	337.287	96.69	95.76

Supplementary Fig. 184. HPLC spectrum of chiral (*P*, *E*)-3k

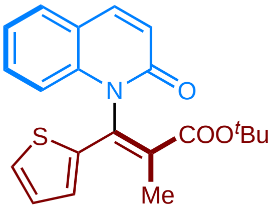

(*P*, *Z*)-**3l**

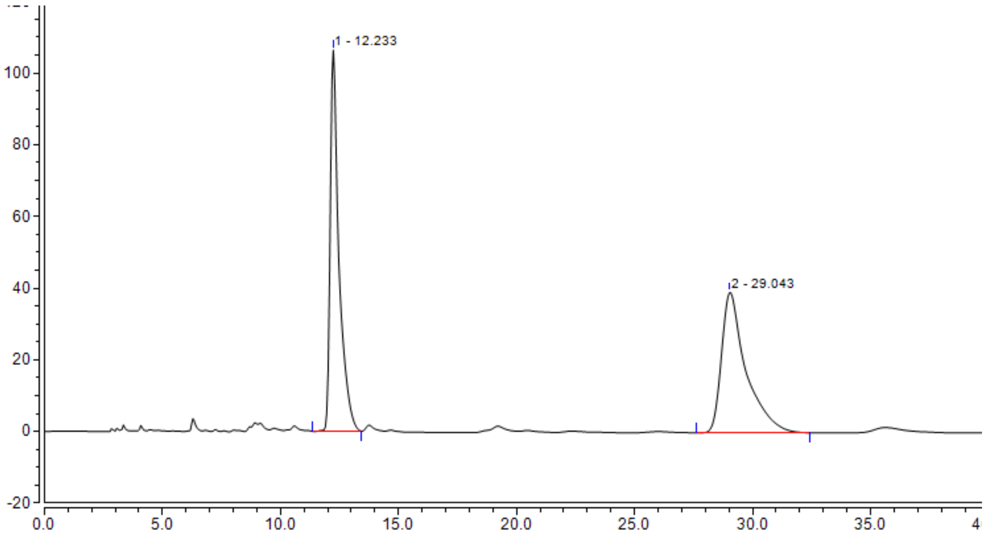

Peak	Ret. Time (min)	Area (mAu*min)	Height (mAu)	Area %	Height %
1	12.233	45.878	106.339	49.46	73.07
2	29.043	46.876	39.191	50.54	26.93

Supplementary Fig. 185. HPLC spectrum of racemic (*P*, *Z*)-3l

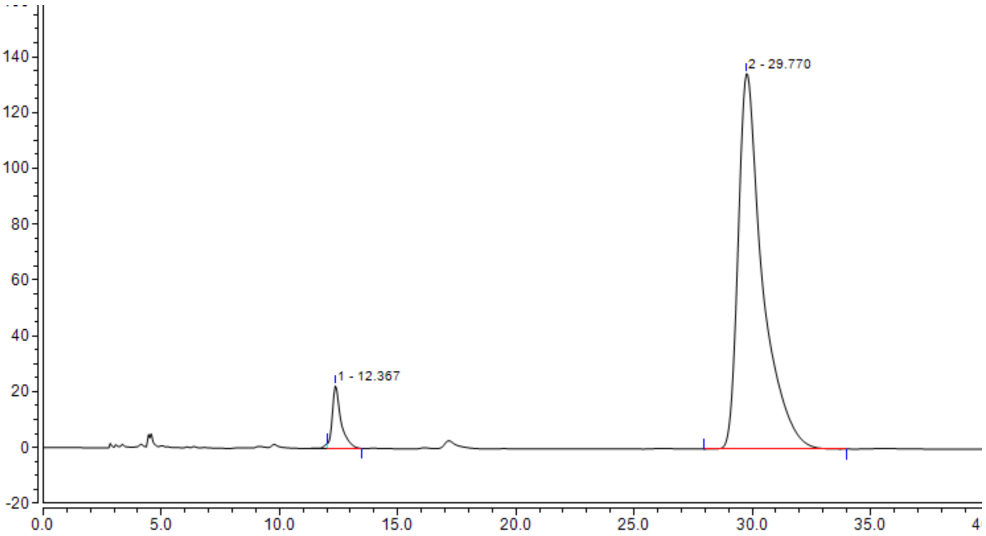

Peak	Ret. Time (min)	Area (mAu*min)	Height (mAu)	Area %	Height %
1	12.367	9.583	22.538	5.65	14.34
2	29.770	159.962	134.669	94.35	85.66

Supplementary Fig. 186. HPLC spectrum of chiral (*P*, *Z*)-3l

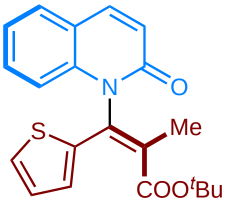

(*P*, *E*)-**3l**

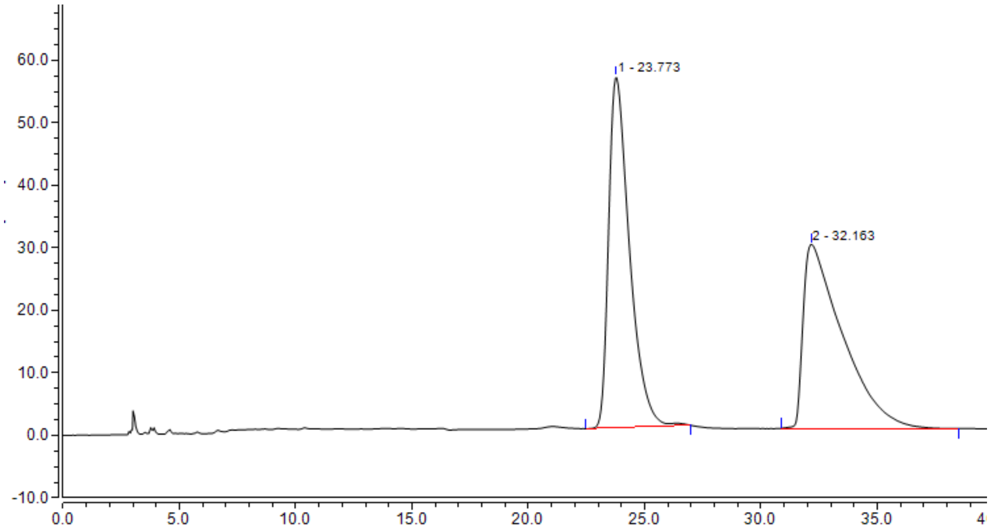

Peak	Ret. Time (min)	Area (mAu*min)	Height (mAu)	Area %	Height %
1	23.773	58.435	56.102	50.32	65.58
2	32.163	57.689	29.451	49.68	34.42

Supplementary Fig. 187. HPLC spectrum of racemic (*P*, *E*)-3l

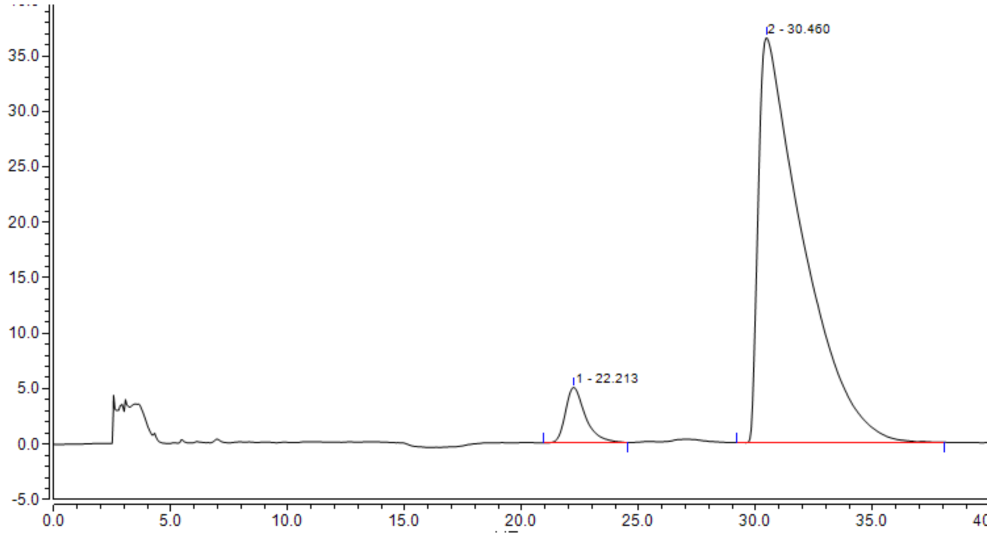

Peak	Ret. Time (min)	Area (mAu*min)	Height (mAu)	Area %	Height %
1	22.213	4.911	4.987	5.73	12.02
2	30.460	80.792	36.489	94.27	87.98

Supplementary Fig. 188. HPLC spectrum of chiral (*P*, *E*)-3l

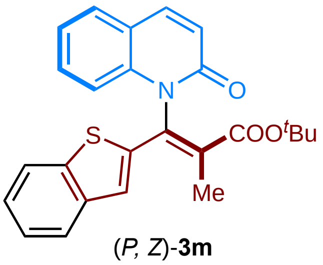

(*P*, *Z*)-**3m**

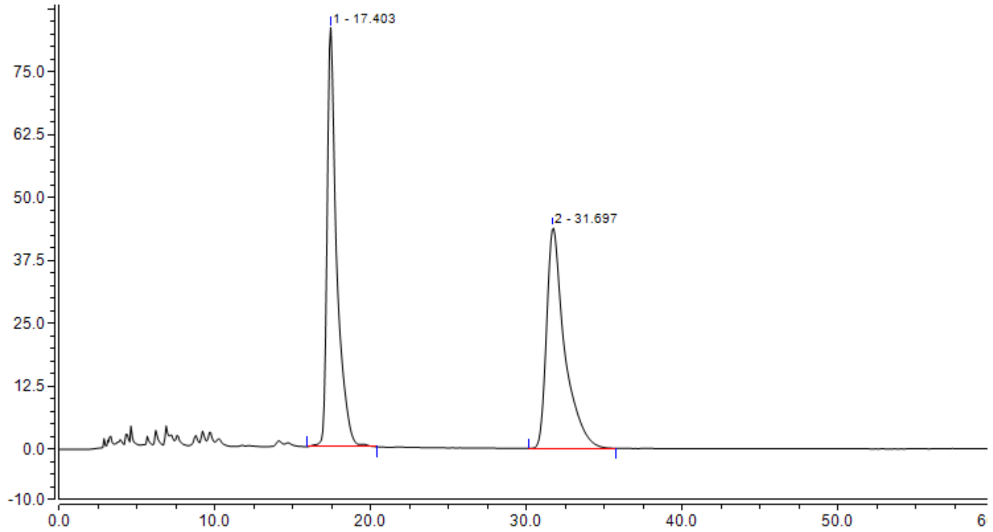

Peak	Ret. Time (min)	Area (mAu*min)	Height (mAu)	Area %	Height %
1	17.403	59.772	83.299	50.50	65.49
2	31.697	58.579	43.895	49.50	34.51

Supplementary Fig. 189. HPLC spectrum of racemic (*P*, *Z*)-3m

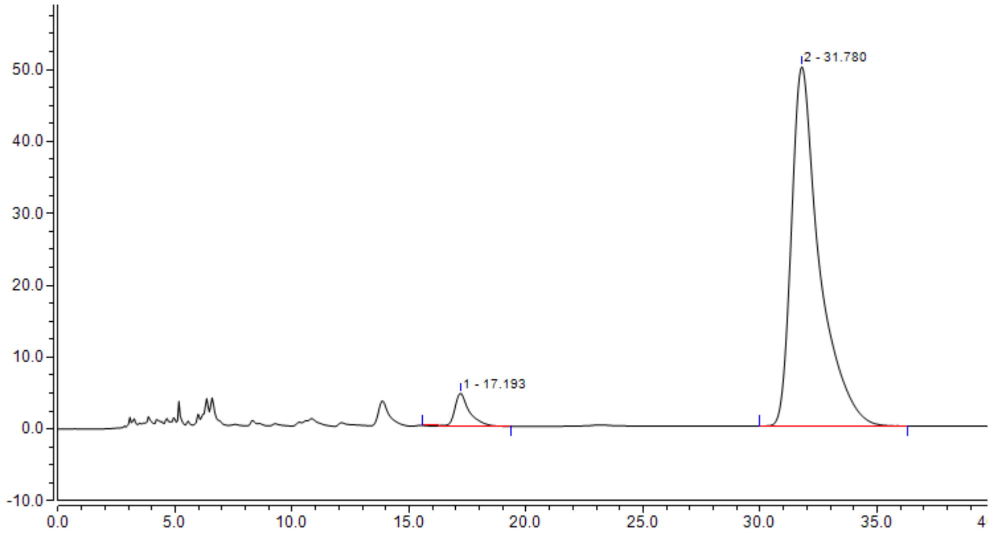

Peak	Ret. Time (min)	Area (mAu*min)	Height (mAu)	Area %	Height %
1	17.193	3.127	4.523	4.32	8.30
2	31.780	69.203	49.999	95.68	91.70

Supplementary Fig. 190. HPLC spectrum of chiral (*P*, *Z*)-3m

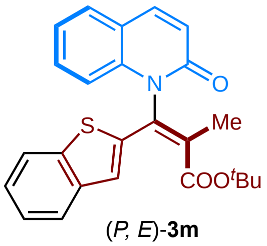

(*P*, *E*)-**3m**

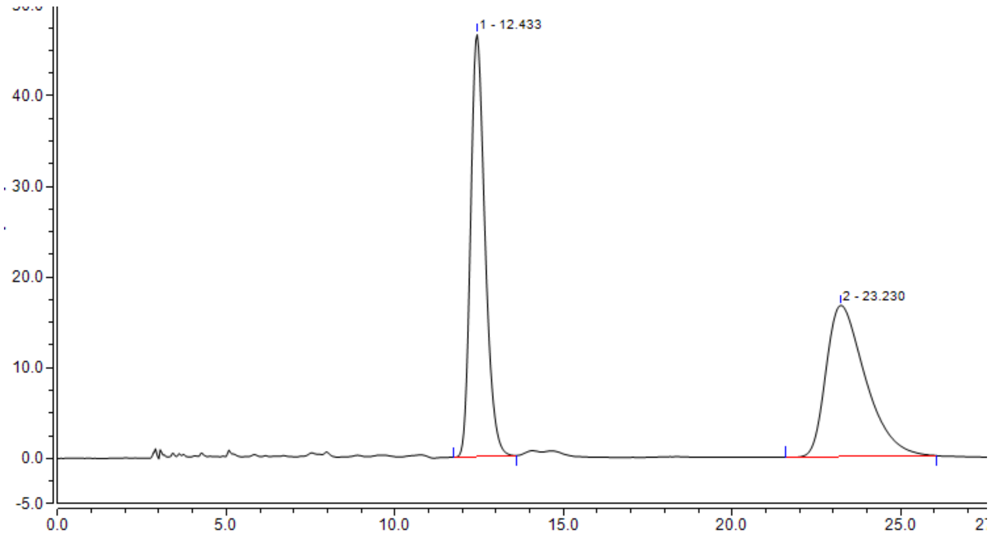

Peak	Ret. Time (min)	Area (mAu*min)	Height (mAu)	Area %	Height %
1	12.433	23.124	46.586	50.63	73.62
2	23.230	22.552	16.692	49.37	26.38

Supplementary Fig. 191. HPLC spectrum of racemic (*P*, *E*)-3m

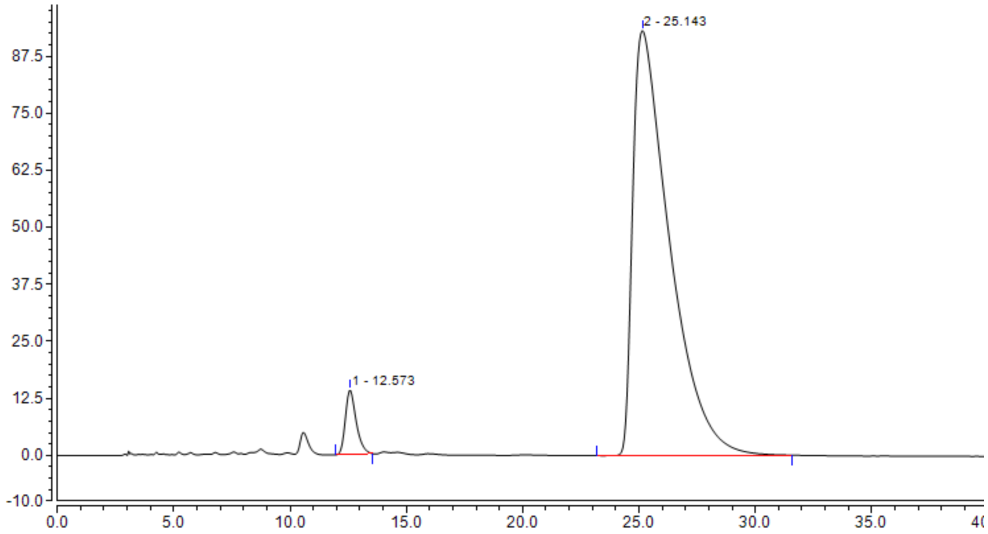

Peak	Ret. Time (min)	Area (mAu*min)	Height (mAu)	Area %	Height %
1	12.573	7.606	14.100	4.26	13.15
2	25.143	170.881	93.141	95.74	86.85

Supplementary Fig. 192. HPLC spectrum of chiral (*P*, *E*)-3m

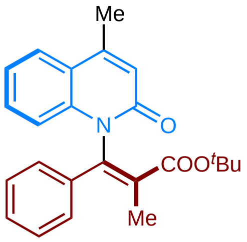

(*P*, *Z*)-**3n**

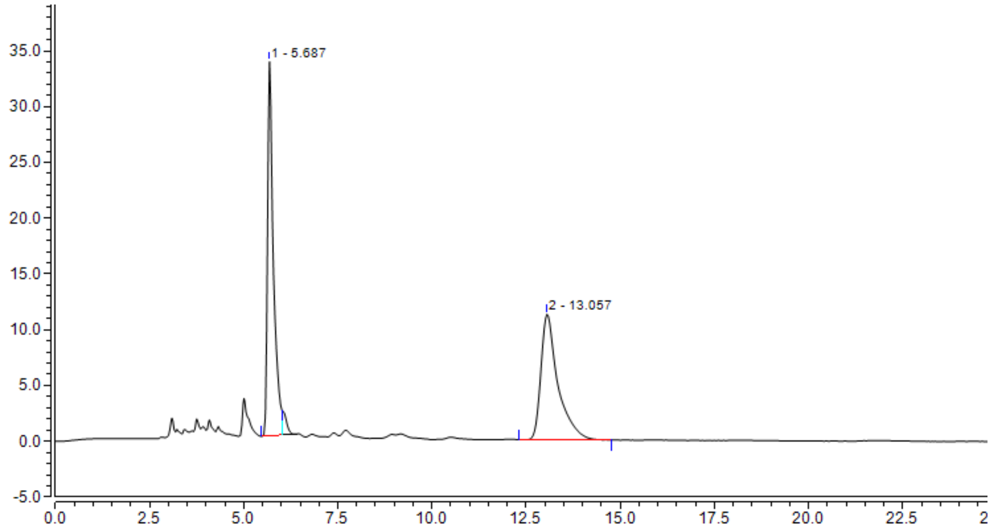

Peak	Ret. Time (min)	Area (mAu*min)	Height (mAu)	Area %	Height %
1	5.687	6.071	33.540	50.90	74.88
2	13.057	5.856	11.252	49.10	25.12

Supplementary Fig. 193. HPLC spectrum of racemic (*P*, *Z*)-3n

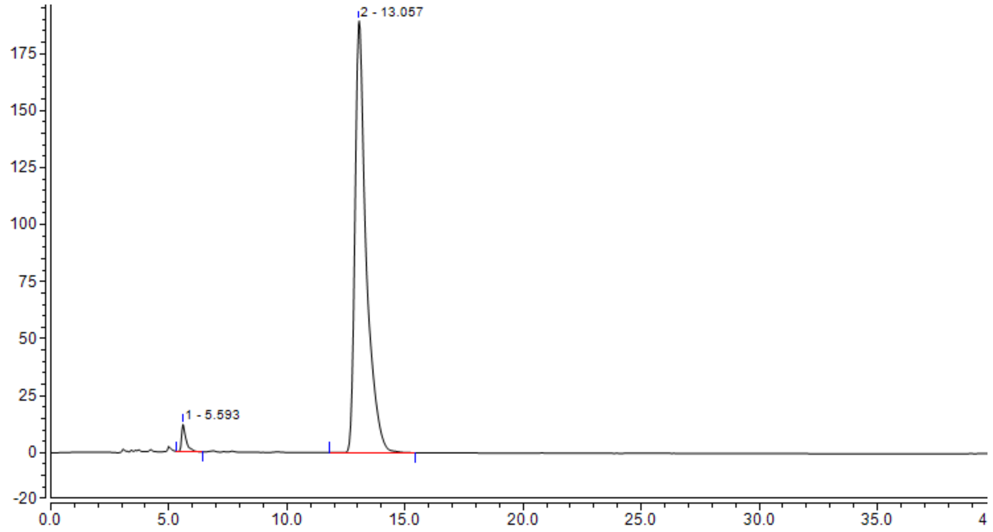

Peak	Ret. Time (min)	Area (mAu*min)	Height (mAu)	Area %	Height %
1	5.593	2.523	11.866	2.36	5.90
2	13.057	104.407	189.253	97.64	94.10

Supplementary Fig. 194. HPLC spectrum of chiral (*P*, *Z*)-3n

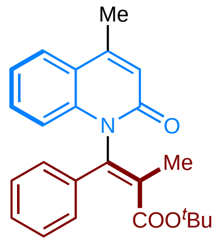

(*P*, *E*)-**3n**

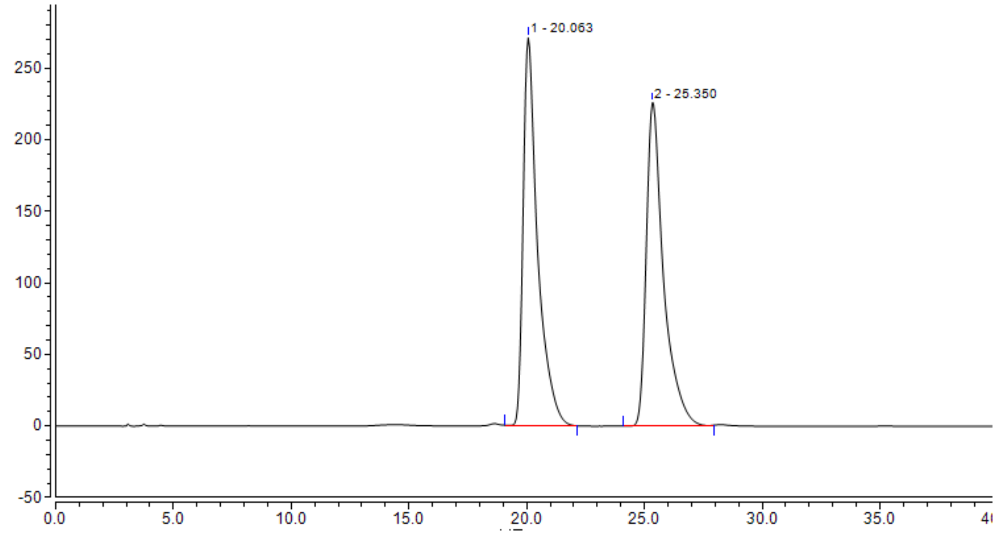

Peak	Ret. Time (min)	Area (mAu*min)	Height (mAu)	Area %	Height %
1	20.063	192.378	270.858	50.20	54.52
2	25.350	190.850	225.973	49.80	45.48

Supplementary Fig. 195. HPLC spectrum of racemic (*P*, *E*)-3n

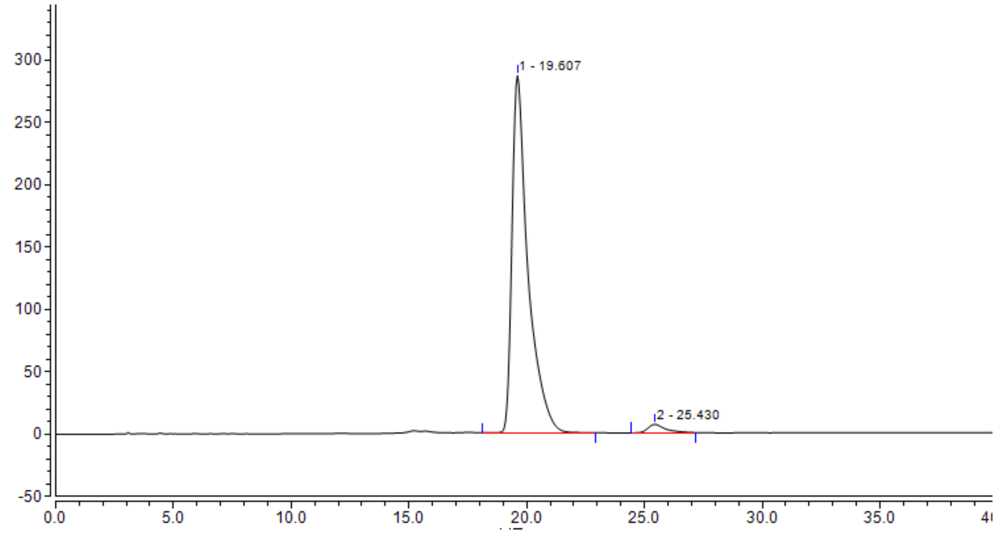

Peak	Ret. Time (min)	Area (mAu*min)	Height (mAu)	Area %	Height %
1	19.607	225.139	286.438	97.48	97.69
2	25.430	5.817	6.762	2.52	2.31

Supplementary Fig. 196. HPLC spectrum of chiral (*P*, *E*)-3n

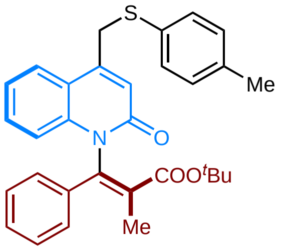

(*P*, *Z*)-**3o**

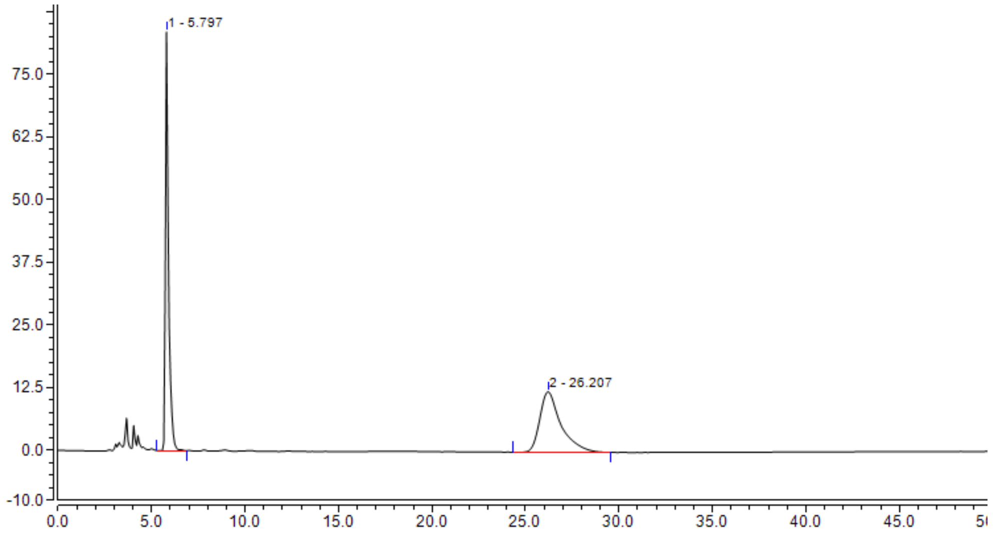

Peak	Ret. Time (min)	Area (mAu*min)	Height (mAu)	Area %	Height %
1	5.797	16.055	83.499	50.31	87.36
2	26.207	15.860	12.079	49.69	12.64

Supplementary Fig. 197. HPLC spectrum of racemic (*P*, *Z*)-3o

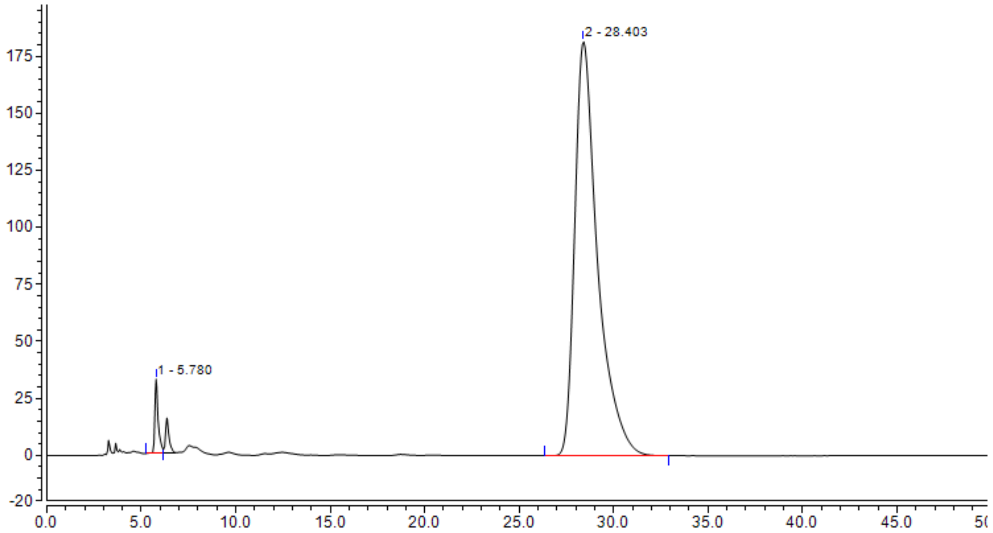

Peak	Ret. Time (min)	Area (mAu*min)	Height (mAu)	Area %	Height %
1	5.780	6.406	32.456	2.45	15.17
2	28.403	254.795	181.489	97.55	84.83

Supplementary Fig. 198. HPLC spectrum of chiral (*P*, *Z*)-3o

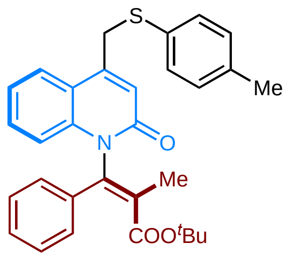

(*P*, *E*)-**3o**

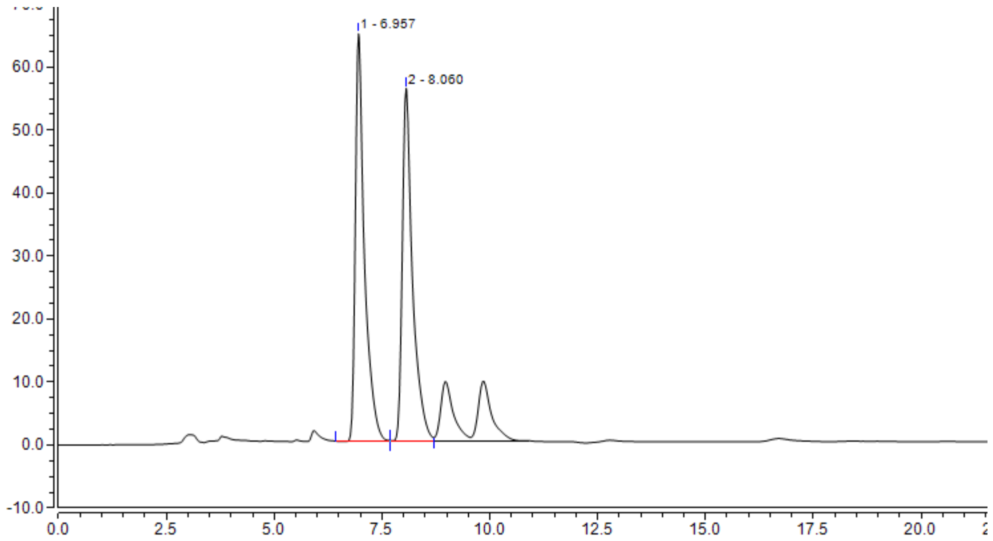

Peak	Ret. Time (min)	Area (mAu*min)	Height (mAu)	Area %	Height %
1	6.957	15.791	64.686	50.65	53.59
2	8.060	15.385	56.013	49.35	46.41

Supplementary Fig. 199. HPLC spectrum of racemic (*P*, *E*)-3o

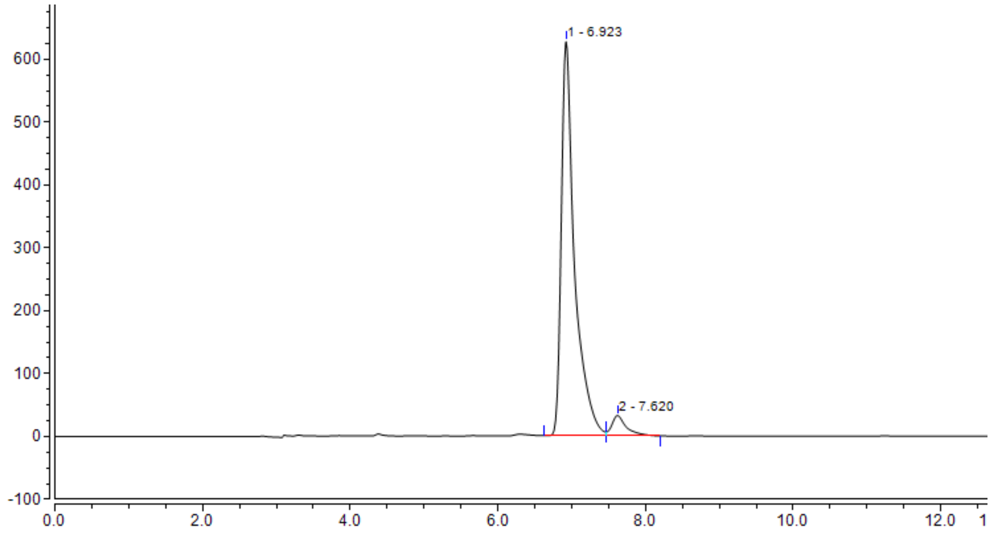

Peak	Ret. Time (min)	Area (mAu*min)	Height (mAu)	Area %	Height %
1	6.923	130.420	626.871	94.65	95.08
2	7.620	7.372	32.456	5.35	4.92

Supplementary Fig. 200. HPLC spectrum of chiral (*P*, *E*)-3o

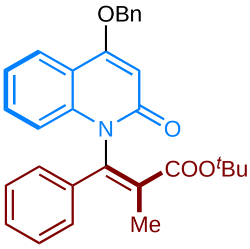

(P, Z)-**3p**

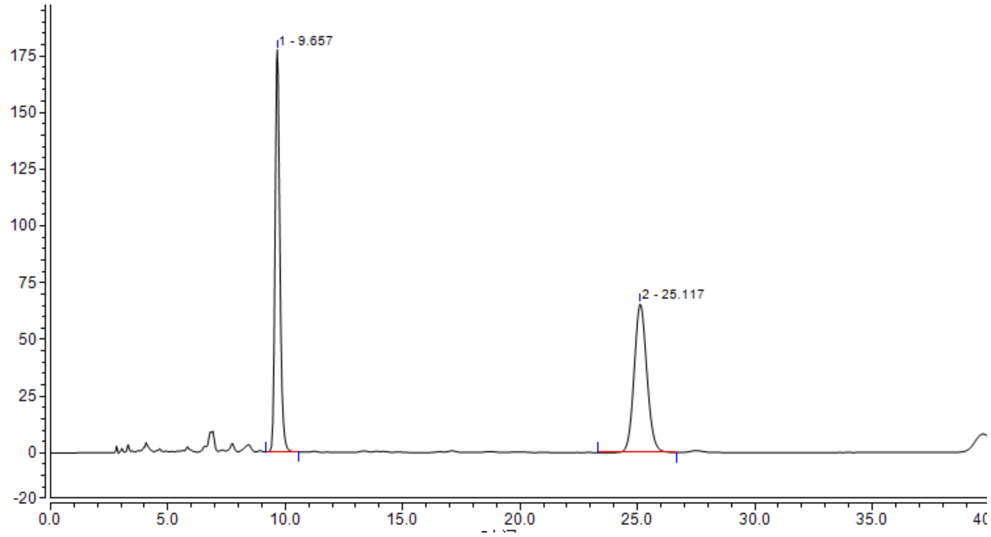

Peak	Ret. Time (min)	Area (mAu*min)	Height (mAu)	Area %	Height %
1	9.657	41.801	177.306	50.39	73.02
2	25.117	41.158	65.514	49.61	26.98

Supplementary Fig. 201. HPLC spectrum of racemic (*P*, *Z*)-3p

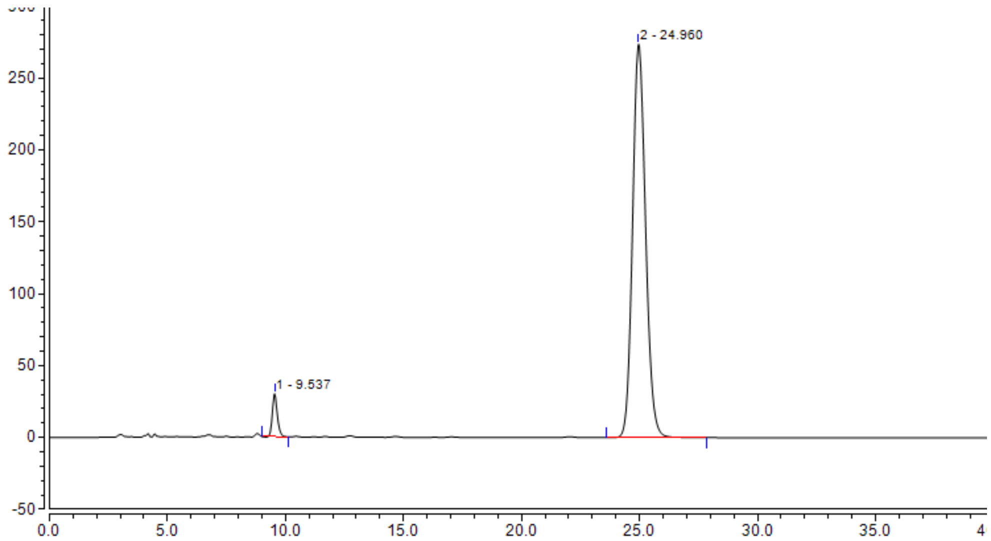

Peak	Ret. Time (min)	Area (mAu*min)	Height (mAu)	Area %	Height %
1	9.537	5.387	29.945	2.96	9.86
2	24.960	176.613	273.706	97.04	90.14

Supplementary Fig. 202. HPLC spectrum of chiral (*P*, *Z*)-3p

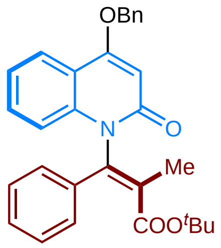

(P, E)-**3p**

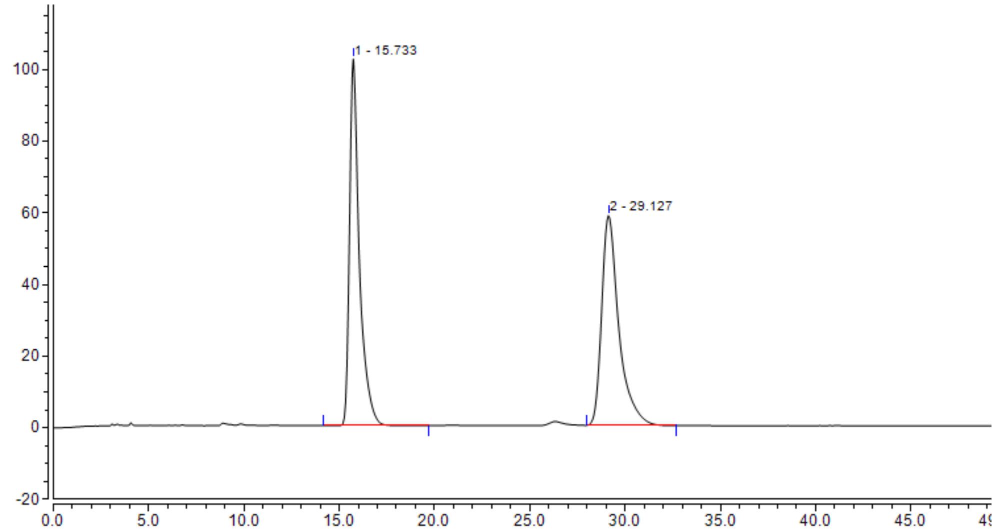

Peak	Ret. Time (min)	Area (mAu*min)	Height (mAu)	Area %	Height %
1	15.733	61.270	102.319	50.07	63.59
2	29.127	61.092	58.578	49.93	36.41

Supplementary Fig. 203. HPLC spectrum of racemic (*P*, *E*)-3p

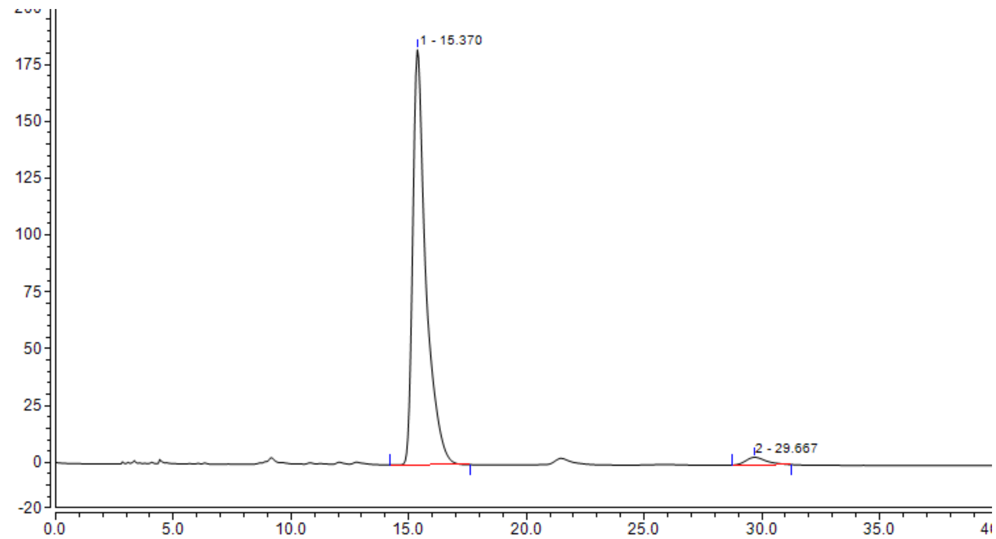

Peak	Ret. Time (min)	Area (mAu*min)	Height (mAu)	Area %	Height %
1	15.370	117.912	182.505	97.19	98.19
2	29.667	3.405	3.360	2.81	1.81

Supplementary Fig. 204. HPLC spectrum of chiral (*P*, *E*)-3p

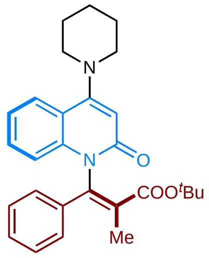

(P, Z)-**3q**

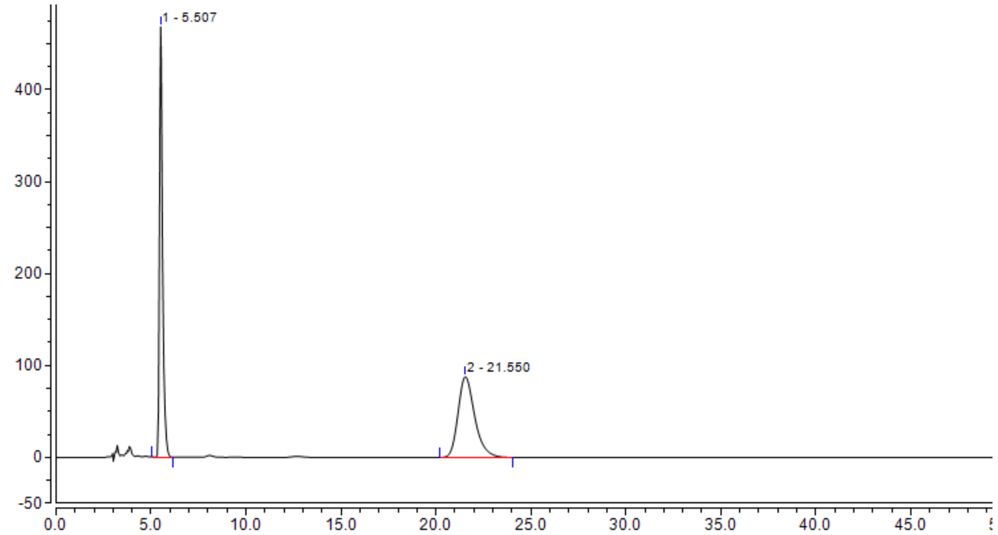

Peak	Ret. Time (min)	Area (mAu*min)	Height (mAu)	Area %	Height %
1	5.507	87.134	468.026	49.31	84.18
2	21.550	89.557	87.971	50.69	15.82

Supplementary Fig. 205. HPLC spectrum of racemic (*P*, *Z*)-3q

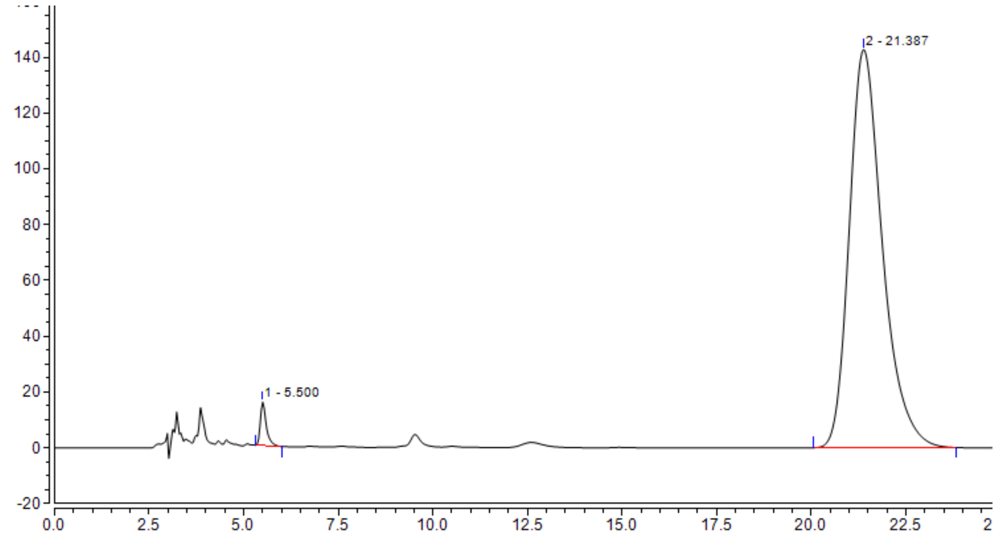

Peak	Ret. Time (min)	Area (mAu*min)	Height (mAu)	Area %	Height %
1	5.500	2.851	15.642	1.93	9.88
2	21.387	144.886	142.672	98.07	90.12

Supplementary Fig. 206. HPLC spectrum of chiral (*P*, *Z*)-3q

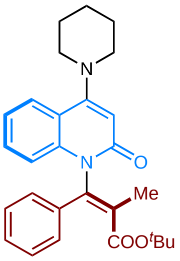

(*P*, *E*)-**3q**

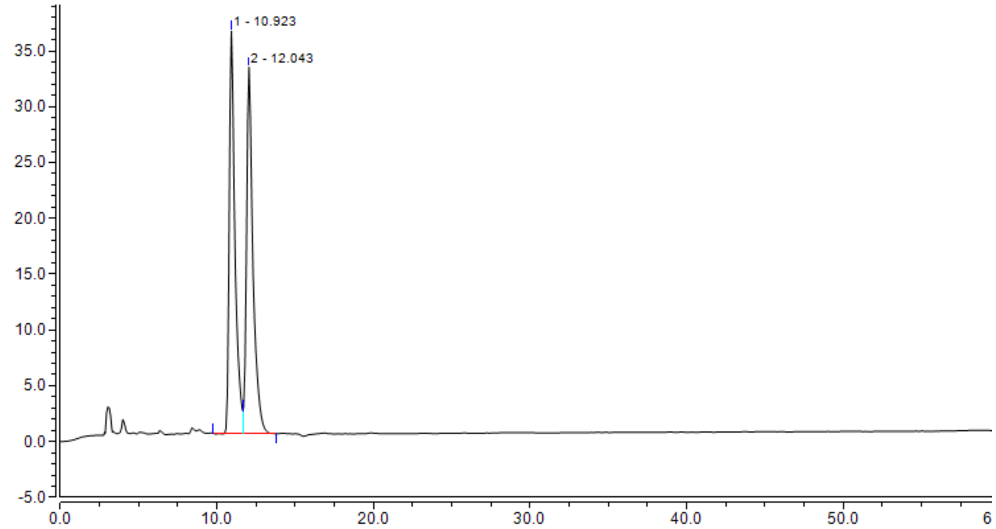

Peak	Ret. Time (min)	Area (mAu*min)	Height (mAu)	Area %	Height %
1	10.923	15.182	36.065	49.14	52.35
2	12.043	15.714	32.831	50.86	47.65

Supplementary Fig. 207. HPLC spectrum of racemic (*P*, *E*)-3q

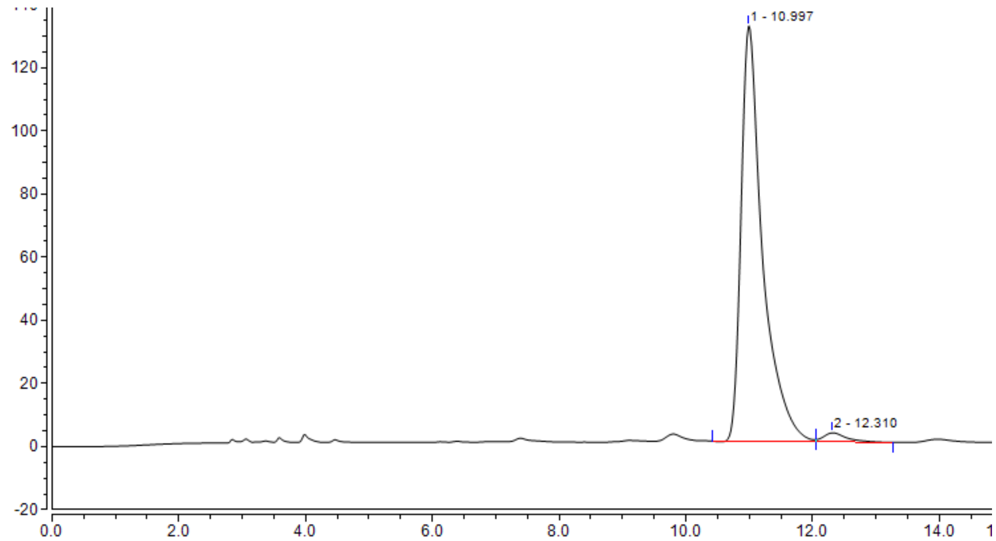

Peak	Ret. Time (min)	Area (mAu*min)	Height (mAu)	Area %	Height %
1	10.997	51.810	131.666	97.64	97.83
2	12.310	1.255	2.917	2.36	2.17

Supplementary Fig. 208. HPLC spectrum of chiral (*P*, *E*)-3q

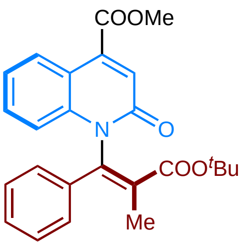

(*P*, *Z*)-**3r**

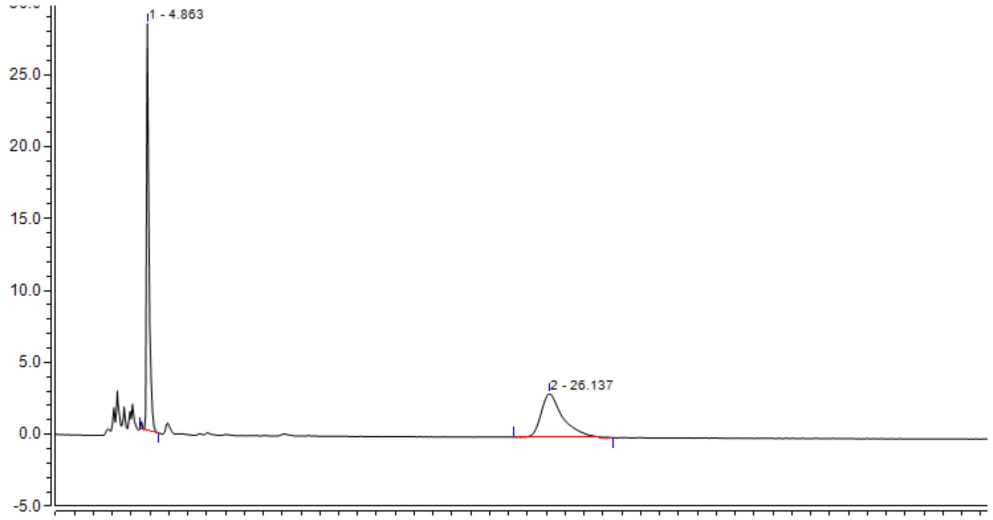

Peak	Ret. Time (min)	Area (mAu*min)	Height (mAu)	Area %	Height %
1	4.863	4.144	28.256	50.62	90.37
2	26.137	4.044	3.011	49.38	9.63

Supplementary Fig. 209. HPLC spectrum of racemic (*P*, *Z*)-3r

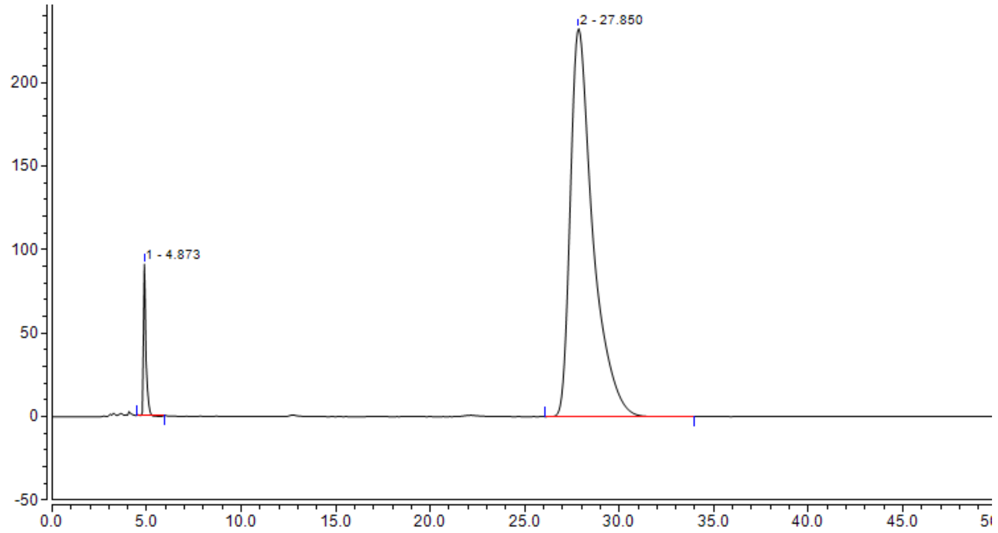

Peak	Ret. Time (min)	Area (mAu*min)	Height (mAu)	Area %	Height %
1	4.873	12.777	90.491	3.84	28.03
2	27.850	319.942	232.356	96.16	71.97

Supplementary Fig. 210. HPLC spectrum of chiral (*P*, *Z*)-3r

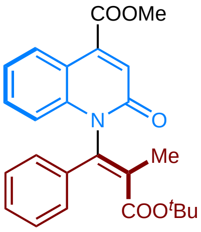

(*P*, *E*)-**3r**

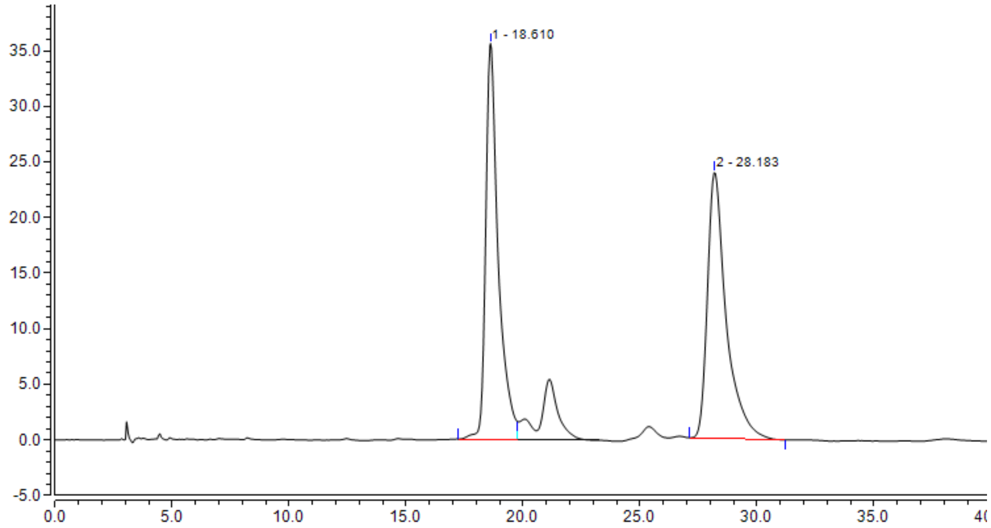

Peak	Ret. Time (min)	Area (mAu*min)	Height (mAu)	Area %	Height %
1	18.610	22.252	35.629	50.04	59.79
2	28.183	22.215	23.962	49.96	40.21

Supplementary Fig. 211. HPLC spectrum of racemic (*P*, *E*)-3r

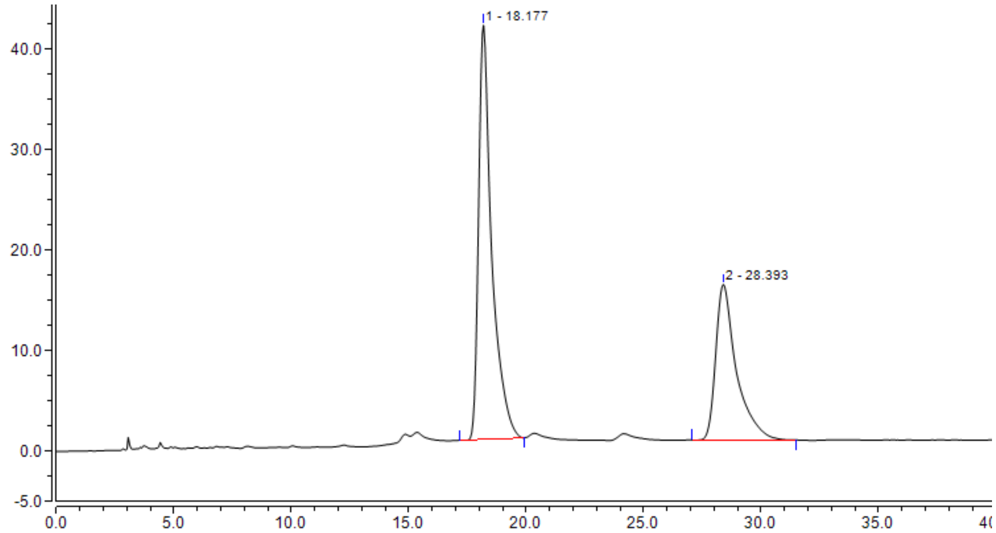

Peak	Ret. Time (min)	Area (mAu*min)	Height (mAu)	Area %	Height %
1	18.177	26.892	41.213	63.14	72.69
2	28.393	15.702	15.487	36.86	27.31

Supplementary Fig. 212. HPLC spectrum of chiral (*P*, *E*)-3r

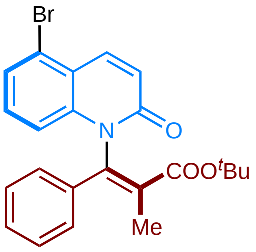

(*P*, *Z*)-**3s**

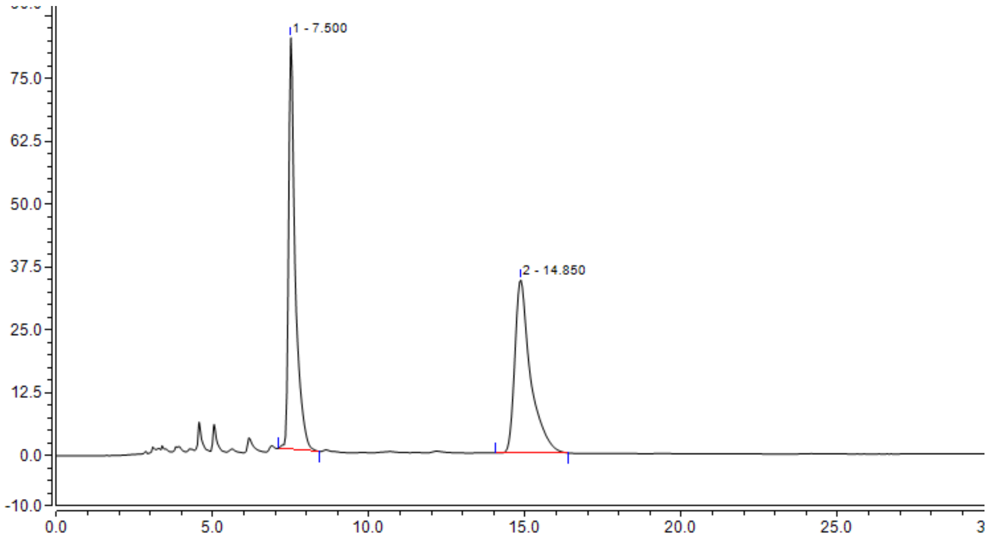

Peak	Ret. Time (min)	Area (mAu*min)	Height (mAu)	Area %	Height %
1	7.500	20.247	81.839	50.38	70.37
2	14.850	19.942	34.465	49.62	29.63

Supplementary Fig. 213. HPLC spectrum of racemic (*P*, *Z*)-3s

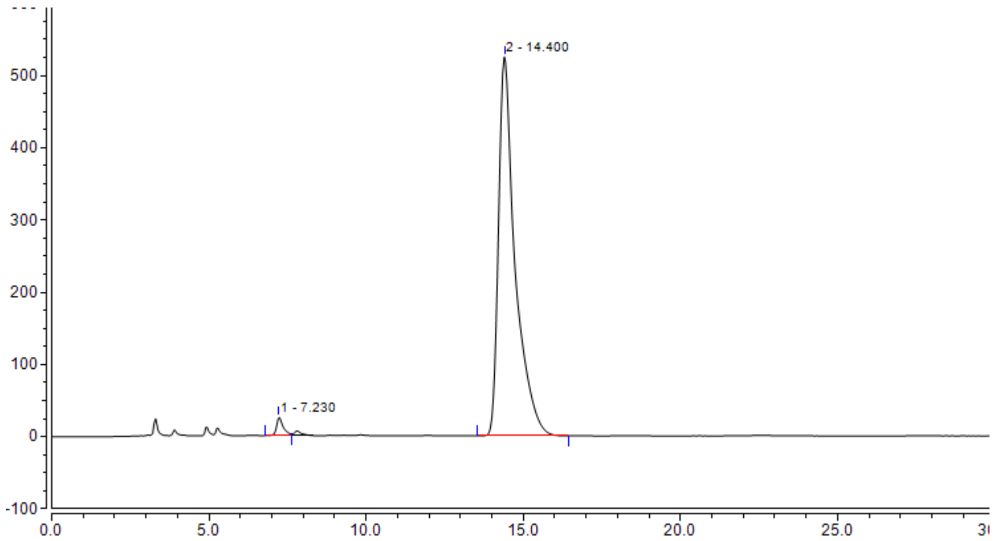

Peak	Ret. Time (min)	Area (mAu*min)	Height (mAu)	Area %	Height %
1	7.230	6.236	25.183	1.91	4.58
2	14.400	320.920	524.797	98.09	95.42

Supplementary Fig. 214. HPLC spectrum of chiral (*P*, *Z*)-3s

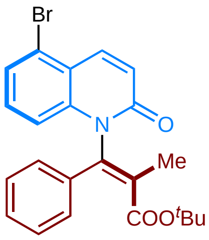

(*P*, *E*)-**3s**

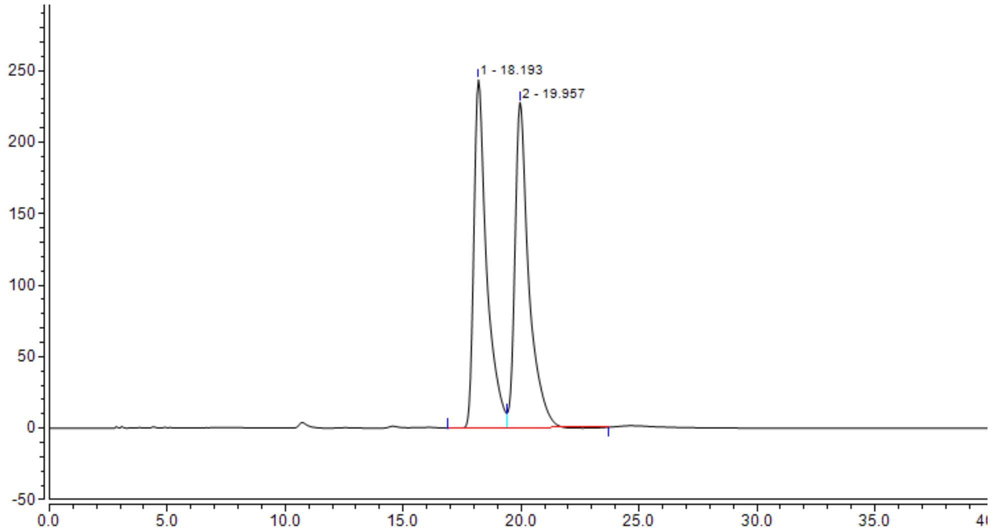

Peak	Ret. Time (min)	Area (mAu*min)	Height (mAu)	Area %	Height %
1	18.193	153.623	243.508	49.82	51.71
2	19.957	154.722	227.430	50.18	48.29

Supplementary Fig. 215. HPLC spectrum of racemic (*P*, *E*)-3s

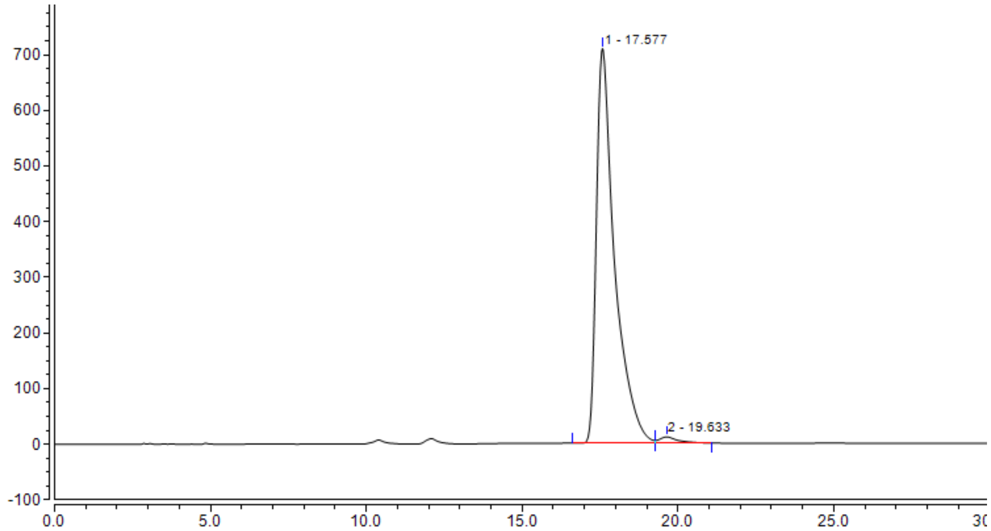

Peak	Ret. Time (min)	Area (mAu*min)	Height (mAu)	Area %	Height %
1	17.577	480.214	708.963	98.44	98.46
2	19.633	7.597	11.112	1.56	1.54

Supplementary Fig. 216. HPLC spectrum of chiral (*P*, *E*)-3s

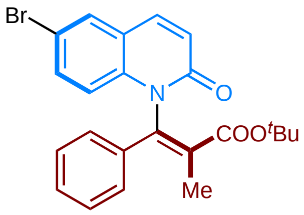

(P, Z)-**3t**

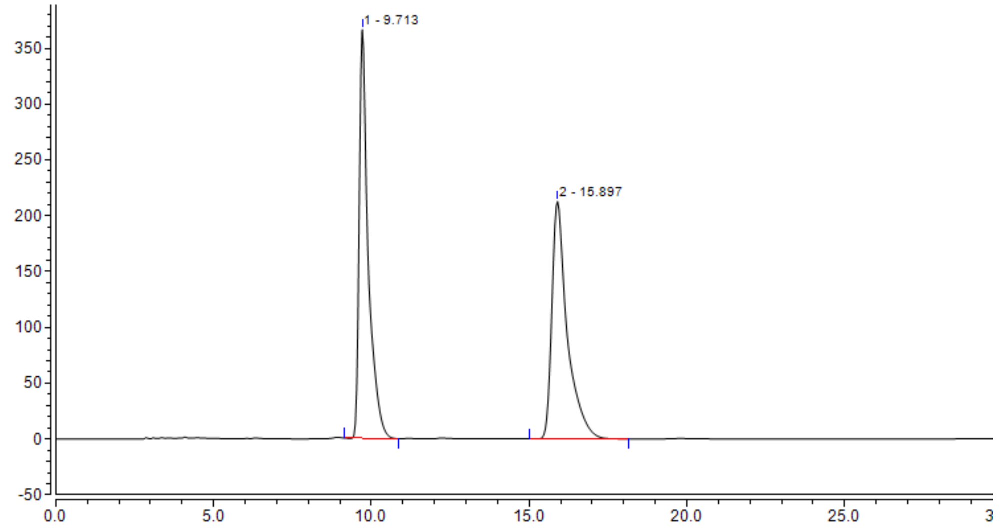

Peak	Ret. Time (min)	Area (mAu*min)	Height (mAu)	Area %	Height %
1	9.713	121.540	365.963	50.32	63.23
2	15.897	120.015	212.861	49.68	36.77

Supplementary Fig. 217. HPLC spectrum of racemic (*P*, *Z*)-3t

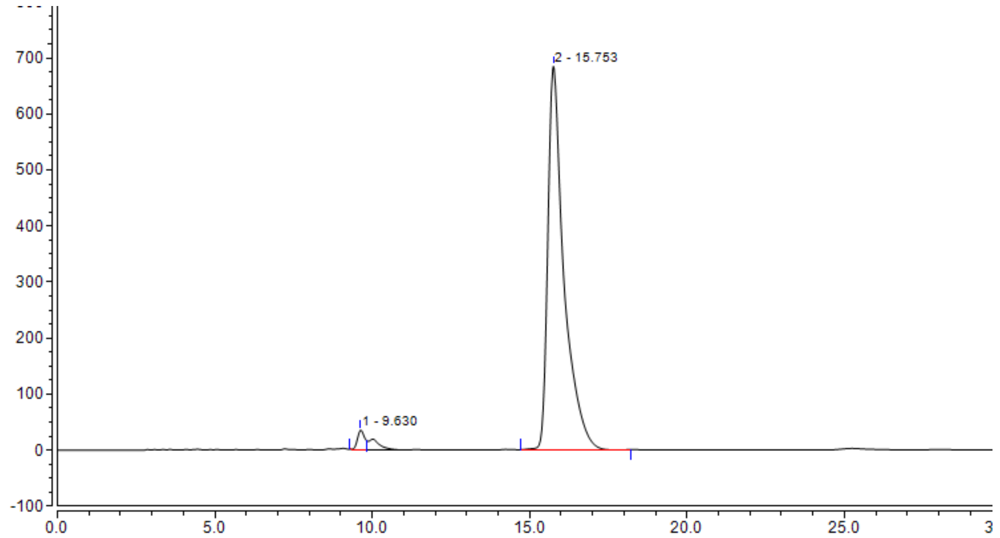

Peak	Ret. Time (min)	Area (mAu*min)	Height (mAu)	Area %	Height %
1	9.630	8.908	34.650	2.15	4.82
2	15.753	404.730	684.236	97.85	95.18

Supplementary Fig. 218. HPLC spectrum of chiral (*P*, *Z*)-3t

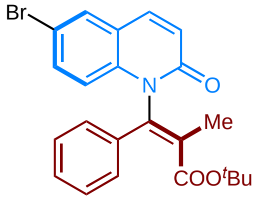

(*P*, *E*)-**3t**

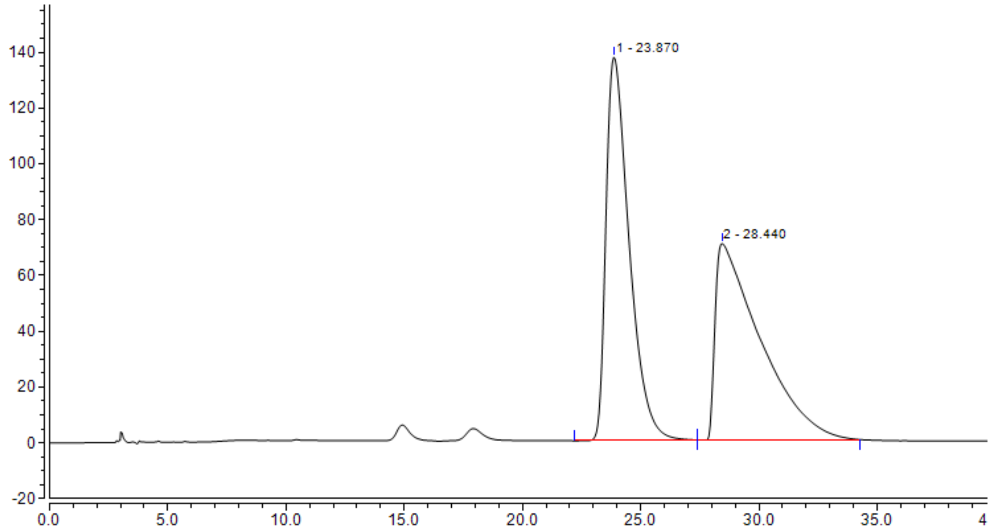

Peak	Ret. Time (min)	Area (mAu*min)	Height (mAu)	Area %	Height %
1	23.870	156.330	137.413	50.11	66.13
2	28.440	155.674	70.388	49.89	33.87

Supplementary Fig. 219. HPLC spectrum of racemic (*P*, *E*)-3t

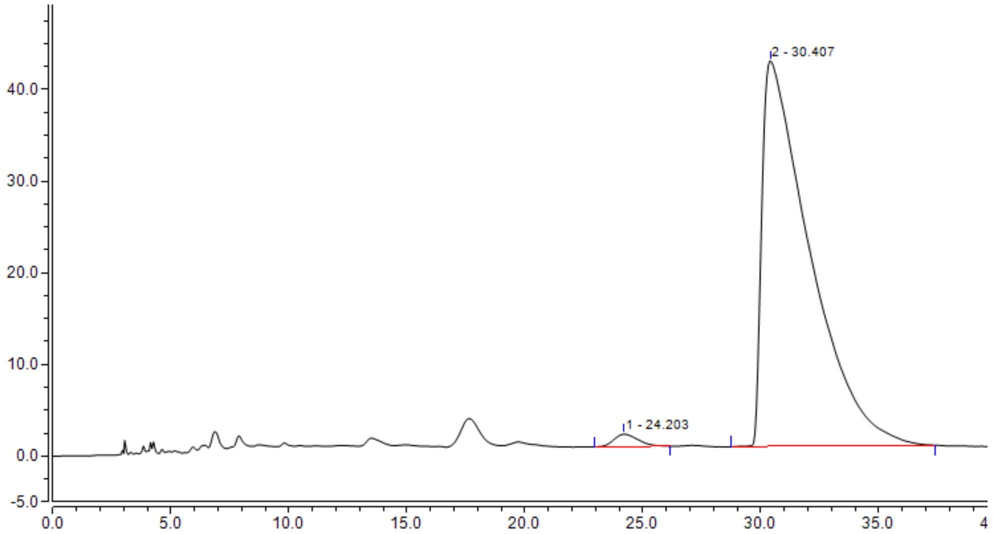

Peak	Ret. Time (min)	Area (mAu*min)	Height (mAu)	Area %	Height %
1	24.203	1.627	1.363	1.62	3.14
2	30.407	98.799	42.070	98.38	96.86

Supplementary Fig. 220. HPLC spectrum of chiral (*P*, *E*)-3t

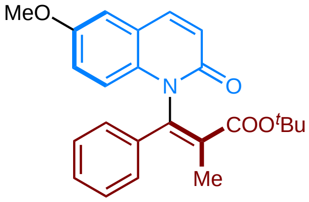

(*P*, *Z*)-**3u**

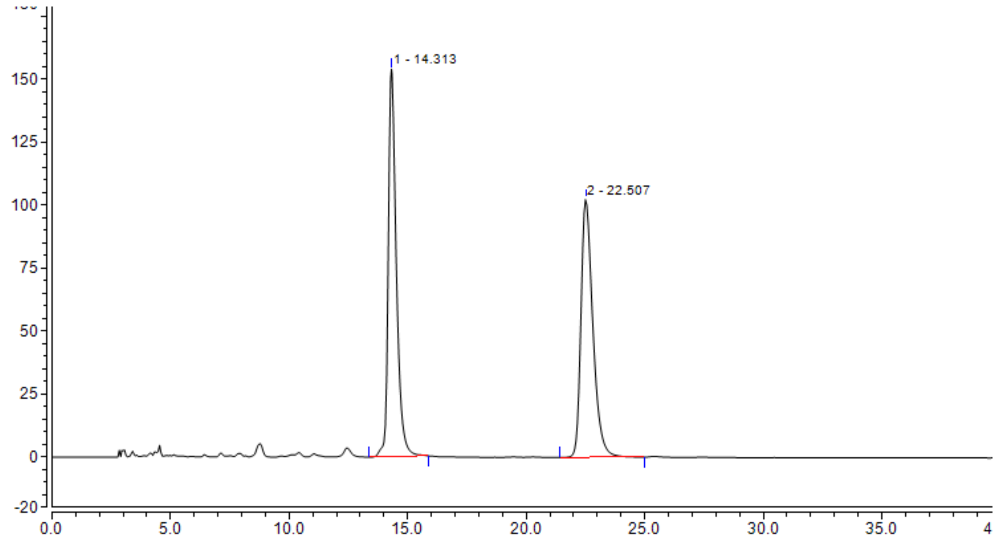

Peak	Ret. Time (min)	Area (mAu*min)	Height (mAu)	Area %	Height %
1	14.313	60.555	153.873	50.39	60.02
2	22.507	59.621	102.483	49.61	39.98

Supplementary Fig. 221. HPLC spectrum of racemic (*P*, *Z*)-3u

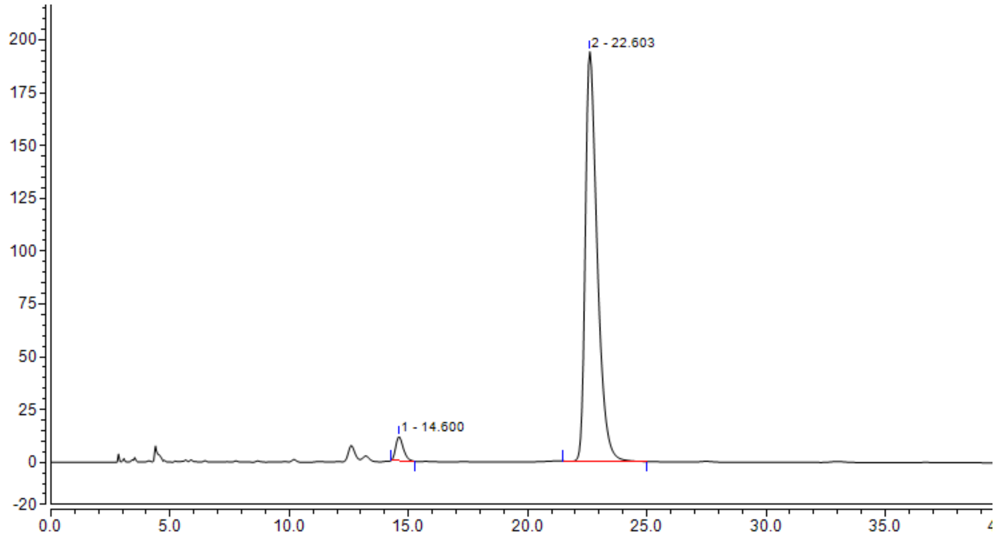

Peak	Ret. Time (min)	Area (mAu*min)	Height (mAu)	Area %	Height %
1	14.600	4.240	11.434	3.65	5.57
2	22.603	112.077	193.942	96.35	94.43

Supplementary Fig. 222. HPLC spectrum of chiral (*P*, *Z*)-3u

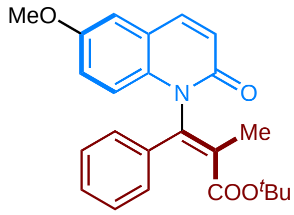

(*P*, *E*)-**3u**

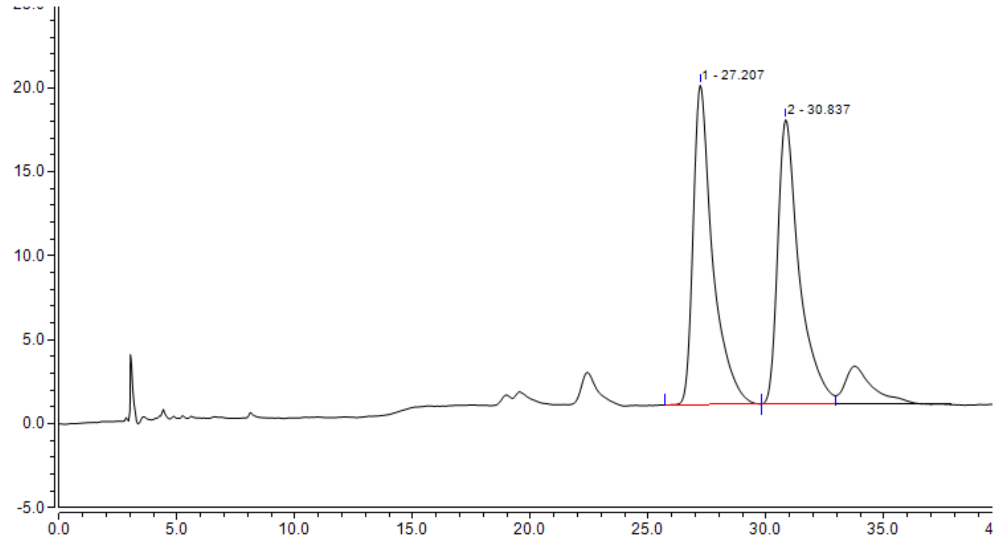

Peak	Ret. Time (min)	Area (mAu*min)	Height (mAu)	Area %	Height %
1	27.207	18.269	19.025	50.40	52.89
2	30.837	17.978	16.944	49.60	47.11

Supplementary Fig. 223. HPLC spectrum of racemic (*P*, *E*)-3u

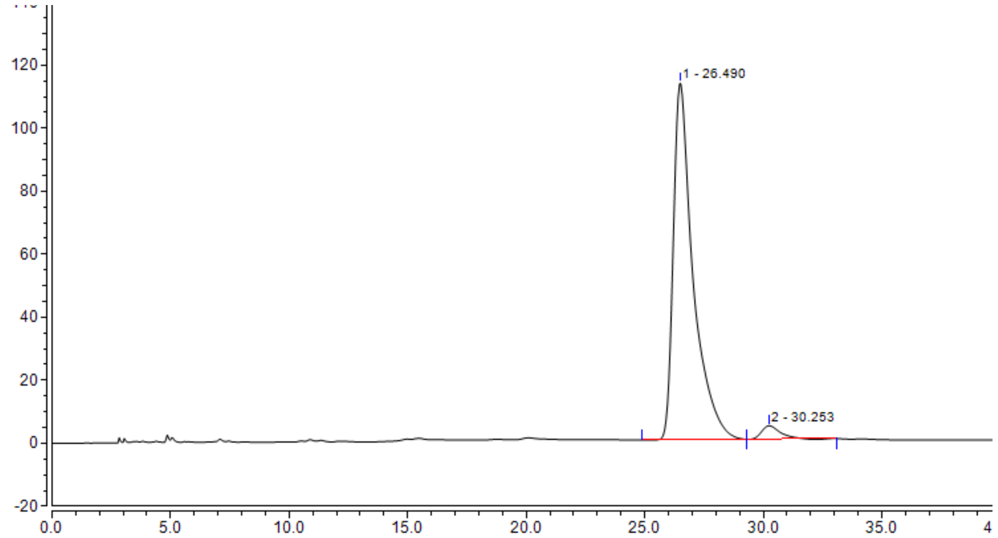

Peak	Ret. Time (min)	Area (mAu*min)	Height (mAu)	Area %	Height %
1	26.490	109.270	113.194	96.81	96.42
2	30.253	3.602	4.201	3.19	3.58

Supplementary Fig. 224. HPLC spectrum of chiral (*P*, *E*)-3u

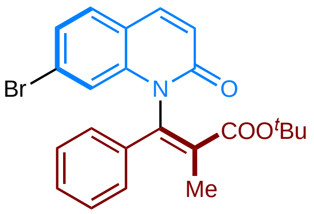

(P, Z)-**3v**

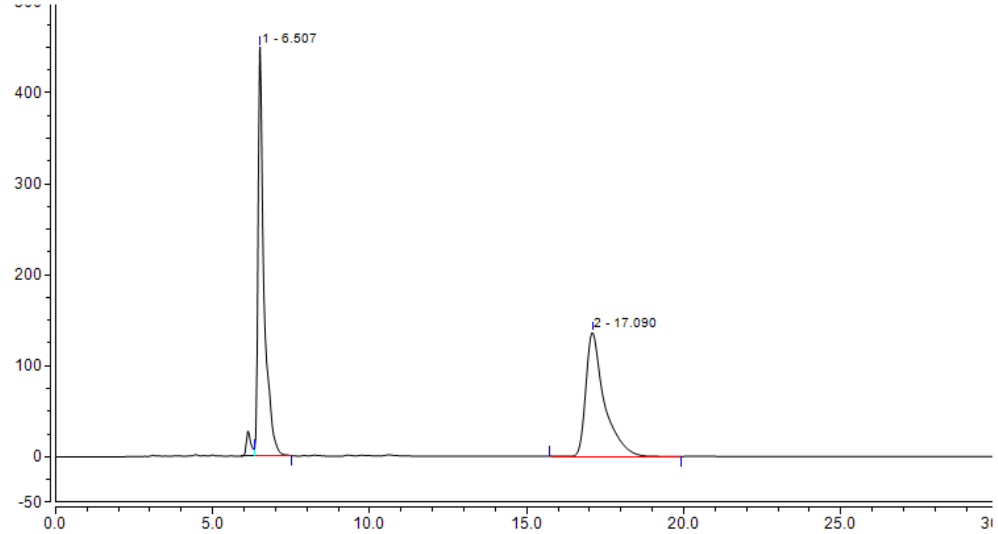

Peak	Ret. Time (min)	Area (mAu*min)	Height (mAu)	Area %	Height %
1	6.507	99.009	449.528	51.77	76.74
2	17.090	92.251	136.254	48.23	23.26

Supplementary Fig. 225. HPLC spectrum of racemic (*P*, *Z*)-3v

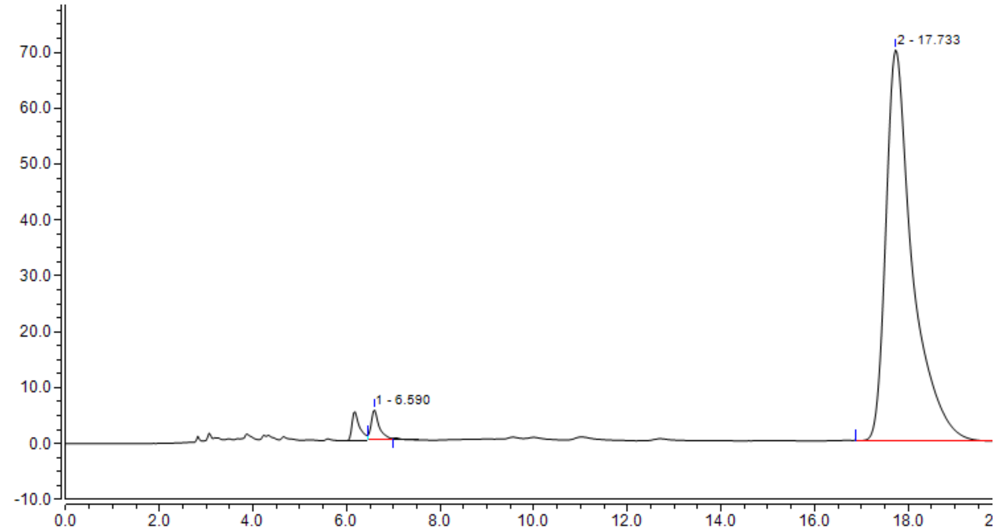

Peak	Ret. Time (min)	Area (mAu*min)	Height (mAu)	Area %	Height %
1	6.590	1.107	5.446	2.36	7.23
2	17.733	45.873	69.928	97.64	92.77

Supplementary Fig. 226. HPLC spectrum of chiral (*P*, *Z*)-3v

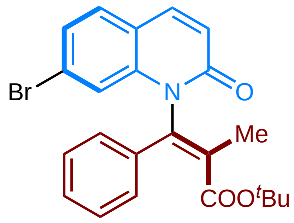

(P, E)-**3v**

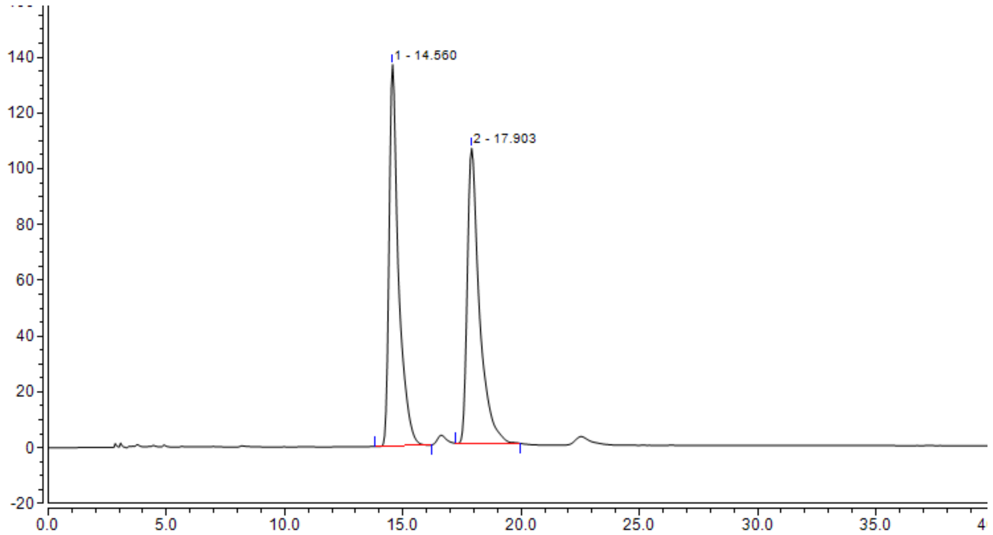

Peak	Ret. Time (min)	Area (mAu*min)	Height (mAu)	Area %	Height %
1	14.560	63.057	136.740	50.10	56.38
2	17.903	62.809	105.795	49.90	43.62

Supplementary Fig. 227. HPLC spectrum of racemic (*P*, *E*)-3v

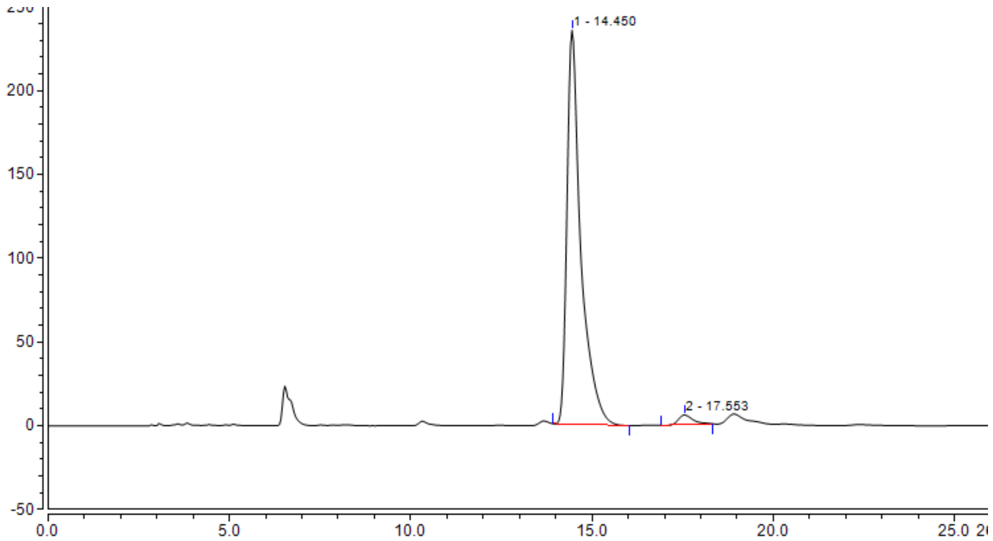

Peak	Ret. Time (min)	Area (mAu*min)	Height (mAu)	Area %	Height %
1	14.450	104.408	235.147	97.50	97.59
2	17.553	2.677	5.816	2.50	2.41

Supplementary Fig. 228. HPLC spectrum of chiral (*P*, *E*)-3v

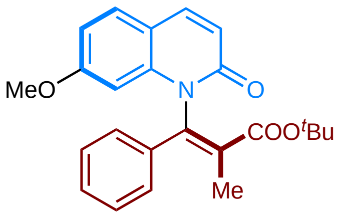

(*P, Z*)-**3w**

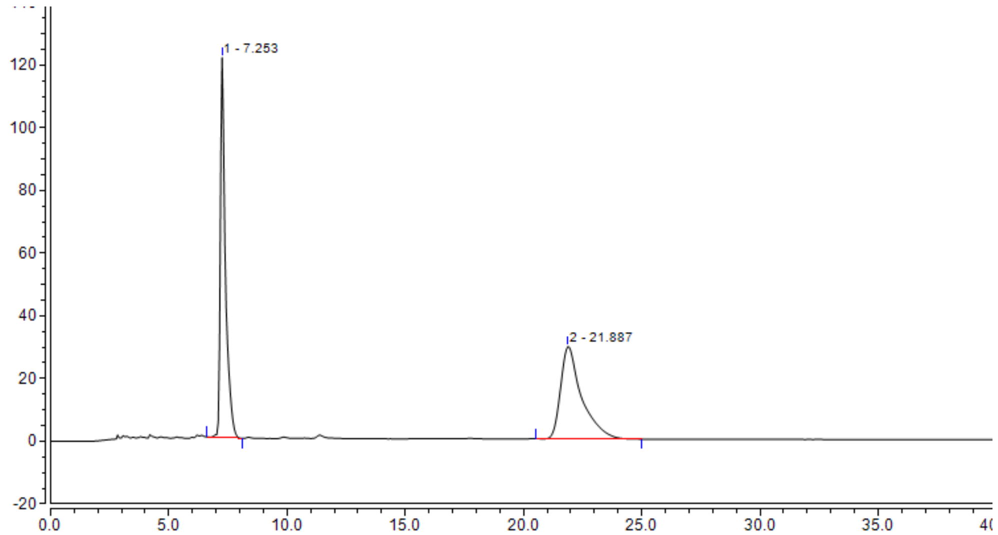

Peak	Ret. Time (min)	Area (mAu*min)	Height (mAu)	Area %	Height %
1	7.253	30.135	121.217	49.88	80.44
2	21.887	30.280	29.483	50.12	19.56

Supplementary Fig. 229. HPLC spectrum of racemic (*P, Z*)-3w

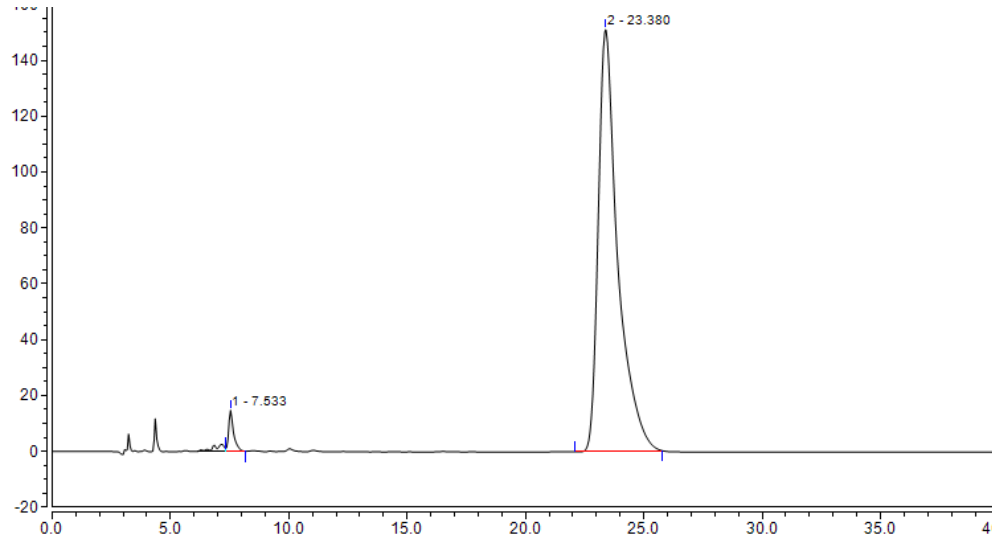

Peak	Ret. Time (min)	Area (mAu*min)	Height (mAu)	Area %	Height %
1	7.533	3.563	14.752	2.41	8.90
2	23.880	144.153	151.010	97.59	91.10

Supplementary Fig. 230. HPLC spectrum of chiral (*P, Z*)-3w

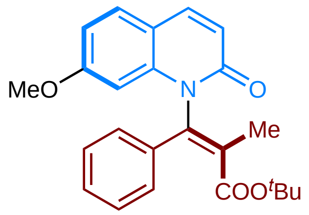

(*P*, *E*)-**3w**

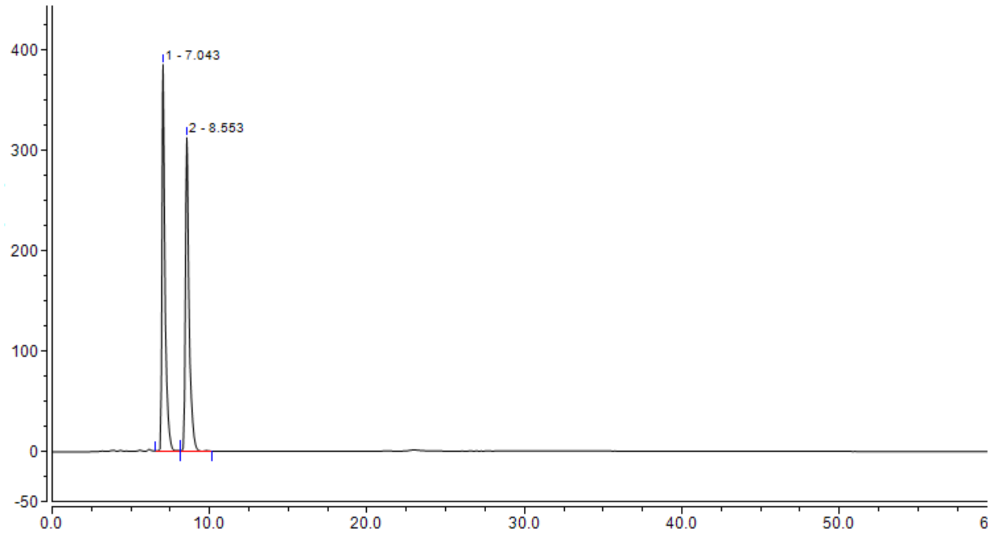

Peak	Ret. Time (min)	Area (mAu*min)	Height (mAu)	Area %	Height %
1	7.043	84.155	384.985	50.34	55.23
2	8.553	83.005	312.099	49.66	44.77

Supplementary Fig. 231. HPLC spectrum of racemic (*P*, *E*)-3w

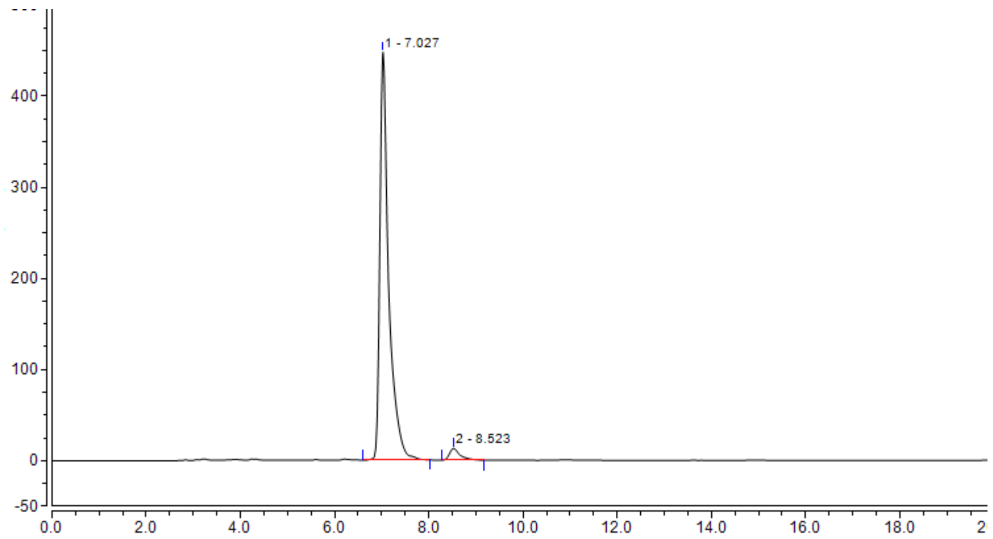

Peak	Ret. Time (min)	Area (mAu*min)	Height (mAu)	Area %	Height %
1	7.027	97.171	447.506	96.64	97.21
2	8.523	3.382	12.840	3.36	2.79

Supplementary Fig. 232. HPLC spectrum of chiral (*P*, *E*)-3w

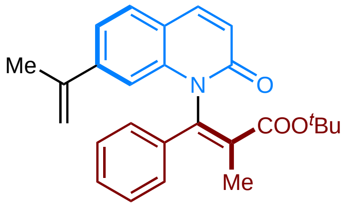

(*P*, *Z*)-**3x**

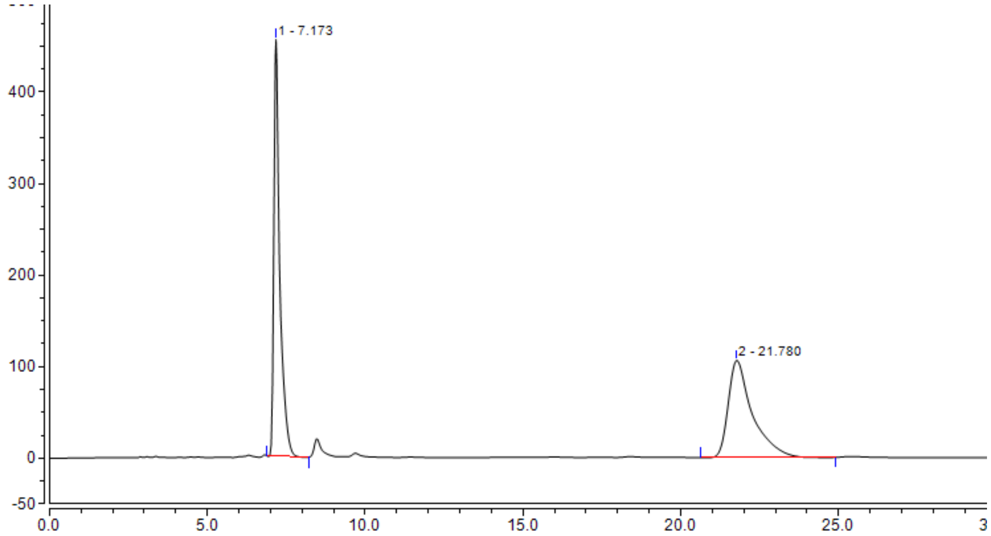

Peak	Ret. Time (min)	Area (mAu*min)	Height (mAu)	Area %	Height %
1	7.173	98.254	454.769	51.15	81.07
2	21.780	93.824	106.197	48.85	18.93

Supplementary Fig. 233. HPLC spectrum of racemic (*P*, *Z*)-3x

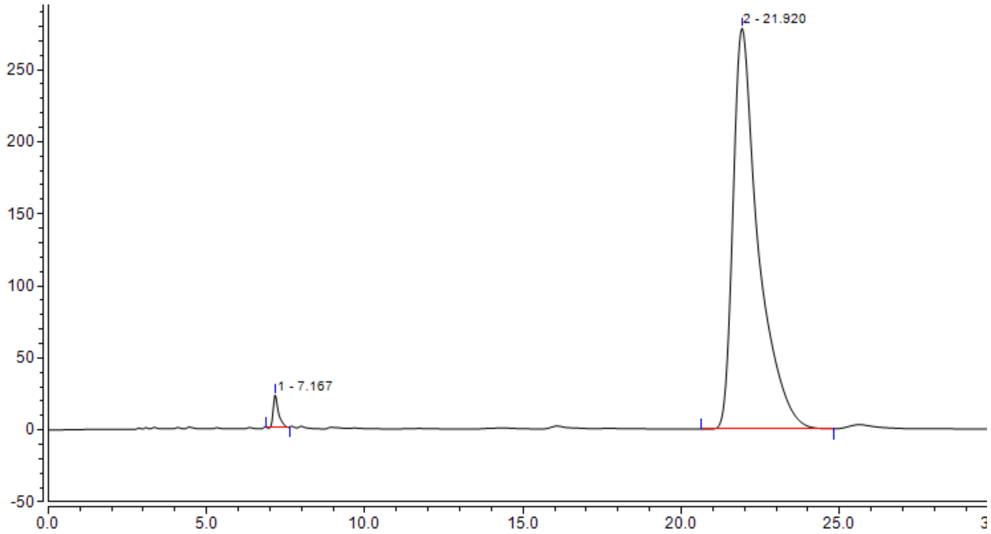

Peak	Ret. Time (min)	Area (mAu*min)	Height (mAu)	Area %	Height %
1	7.167	4.239	22.489	1.62	7.48
2	21.920	256.936	278.304	98.38	92.52

Supplementary Fig. 234. HPLC spectrum of chiral (*P*, *Z*)-3x

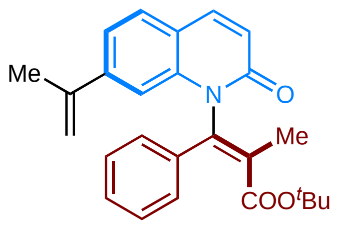

(*P*, *E*)-**3x**

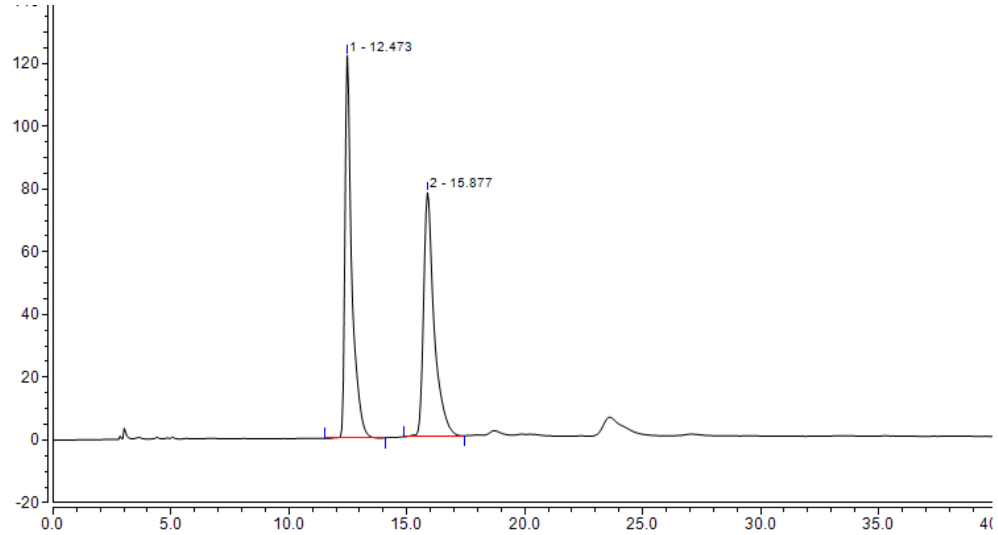

Peak	Ret. Time (min)	Area (mAu*min)	Height (mAu)	Area %	Height %
1	12.473	41.143	121.998	50.50	61.08
2	15.877	40.323	77.733	49.50	38.92

Supplementary Fig. 235. HPLC spectrum of racemic (*P*, *E*)-3x

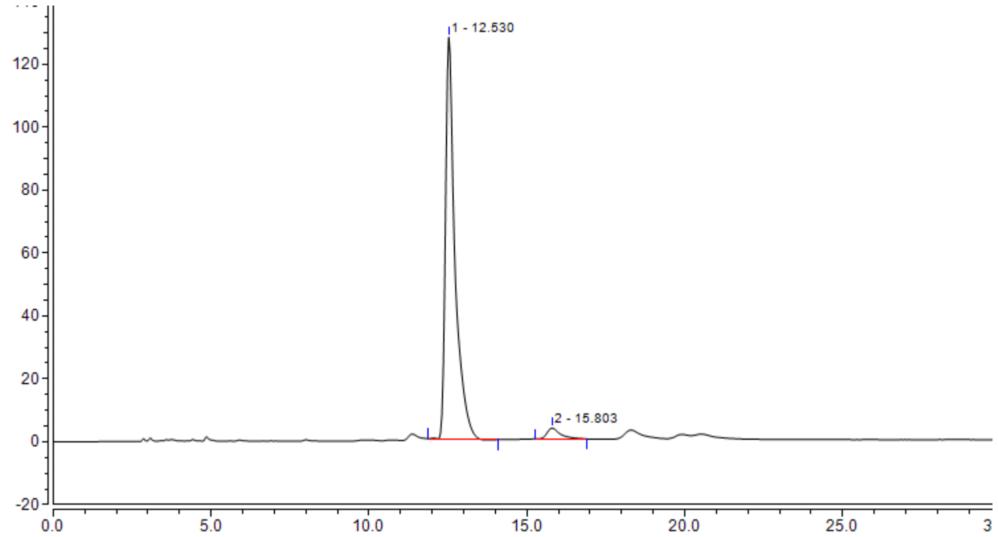

Peak	Ret. Time (min)	Area (mAu*min)	Height (mAu)	Area %	Height %
1	12.530	44.545	127.767	96.35	97.36
2	15.803	1.686	3.464	3.65	2.64

Supplementary Fig. 236. HPLC spectrum of chiral (*P*, *E*)-3x

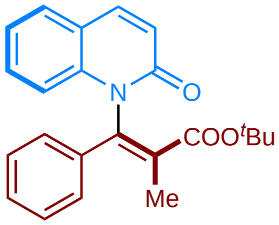

(*P*, *Z*)-**3a**

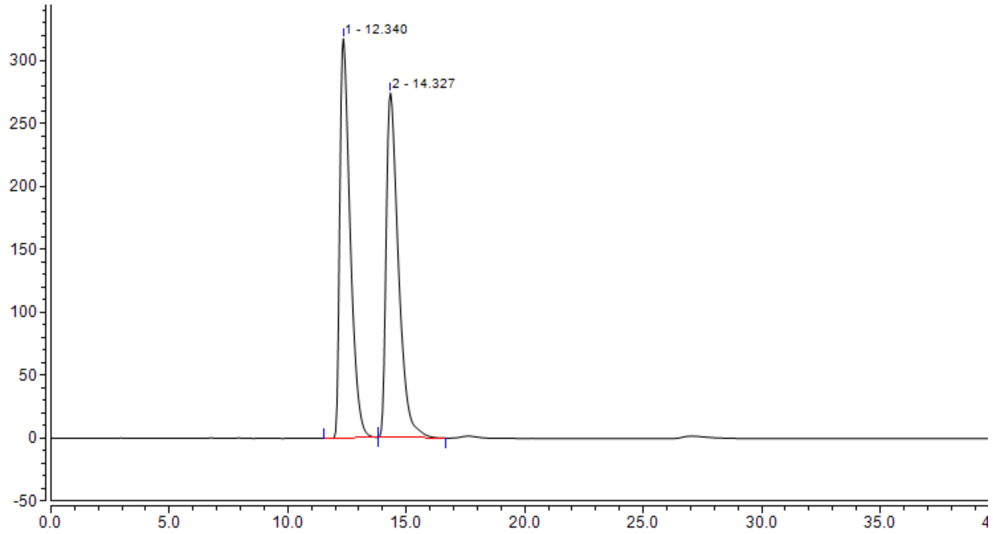

Peak	Ret. Time (min)	Area (mAu*min)	Height (mAu)	Area %	Height %
1	12.340	160.688	317.392	49.45	53.69
2	14.327	164.233	273.727	50.55	46.31

Supplementary Fig. 237. HPLC spectrum of racemic (*P*, *Z*)-3a

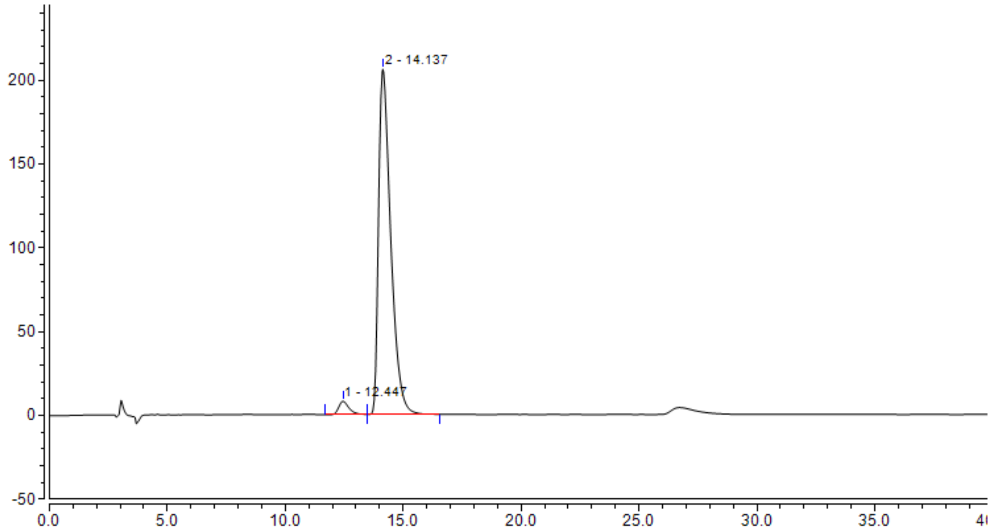

Peak	Ret. Time (min)	Area (mAu*min)	Height (mAu)	Area %	Height %
1	12.447	3.914	7.943	3.08	3.72
2	14.137	123.192	205.858	96.92	96.28

Supplementary Fig. 238. HPLC spectrum of chiral (*P*, *Z*)-3a

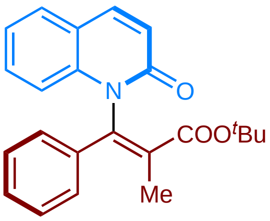

(*M*, *Z*)-**3a**

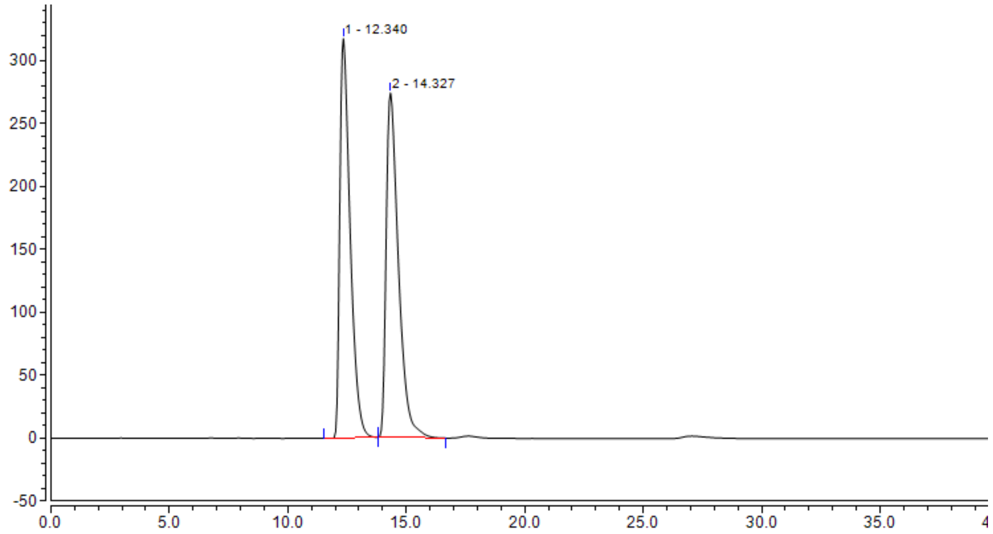

Peak	Ret. Time (min)	Area (mAu*min)	Height (mAu)	Area %	Height %
1	12.340	160.688	317.392	49.45	53.69
2	14.327	164.233	273.727	50.55	46.31

Supplementary Fig. 239. HPLC spectrum of racemic (*M*, *Z*)-3a

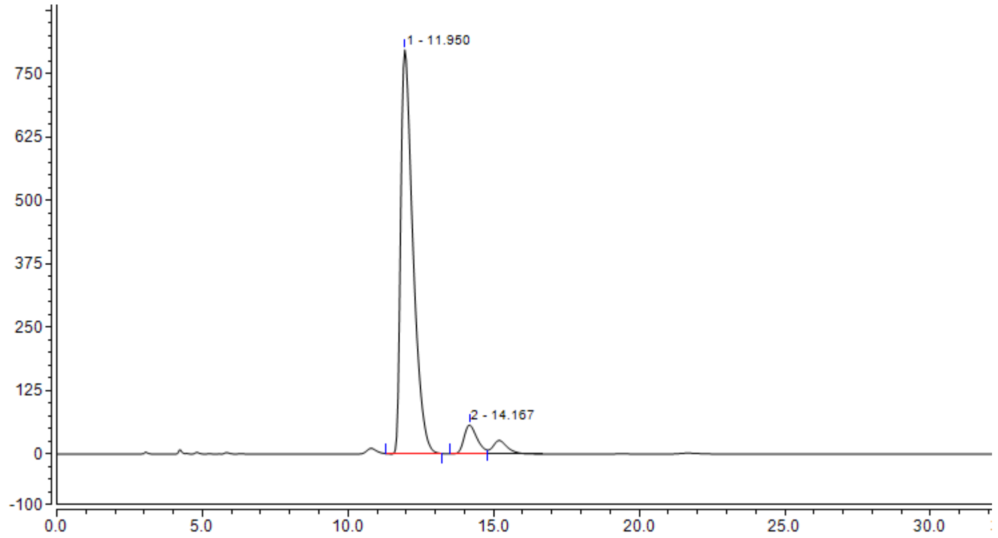

Peak	Ret. Time (min)	Area (mAu*min)	Height (mAu)	Area %	Height %
1	11.960	387.802	796.609	92.88	93.33
2	14.167	29.726	56.894	7.12	6.67

Supplementary Fig. 240. HPLC spectrum of chiral (*M*, *Z*)-3a

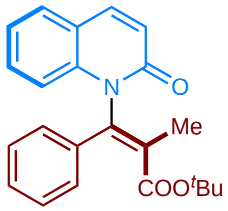

(*P*, *E*)-**3a**

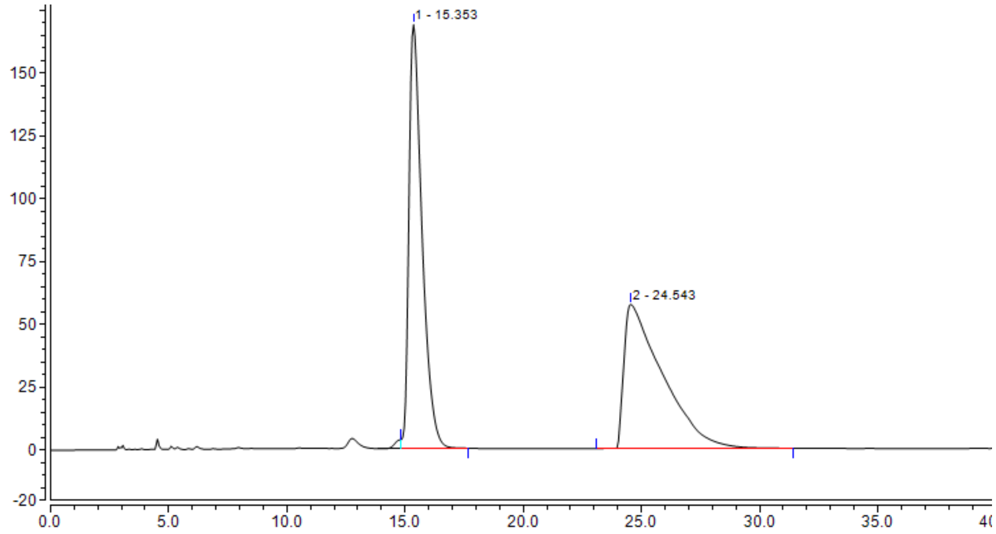

Peak	Ret. Time (min)	Area (mAu*min)	Height (mAu)	Area %	Height %
1	15.353	108.716	168.680	50.03	74.63
2	24.543	108.579	57.338	49.97	25.37

Supplementary Fig. 241. HPLC spectrum of racemic (*P*, *E*)-3a

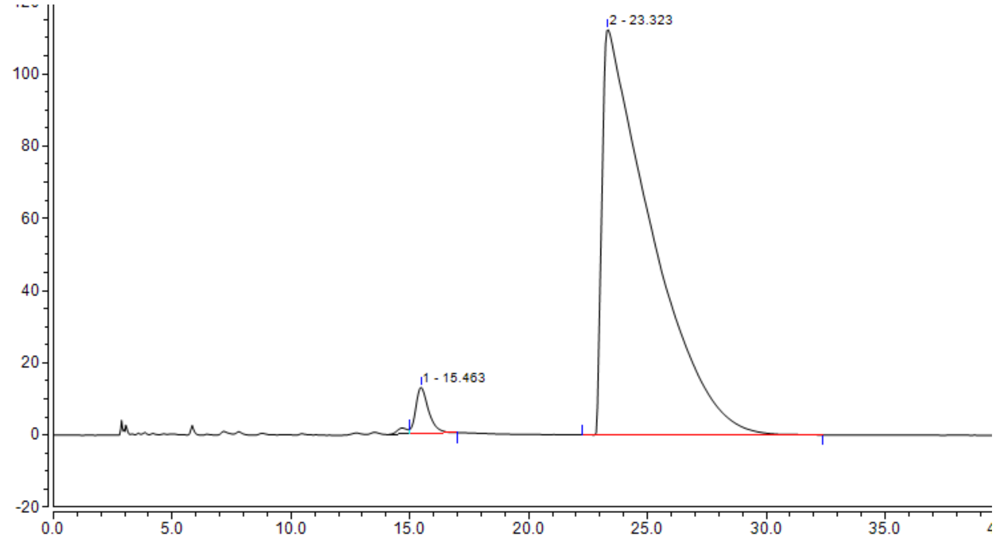

Peak	Ret. Time (min)	Area (mAu*min)	Height (mAu)	Area %	Height %
1	15.463	8.009	12.848	2.88	10.27
2	23.323	270.152	112.303	97.12	89.73

Supplementary Fig. 242. HPLC spectrum of chiral (*P*, *E*)-3a

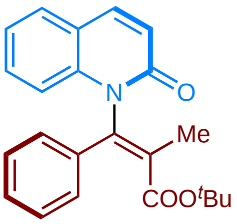

(*M, E*)-**3a**

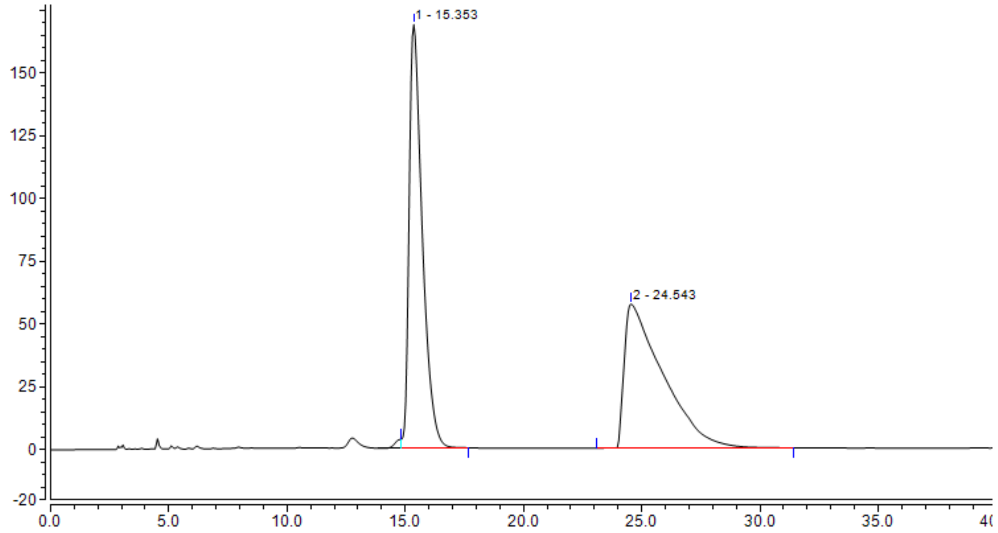

Peak	Ret. Time (min)	Area (mAu*min)	Height (mAu)	Area %	Height %
1	15.353	108.716	168.680	50.03	74.63
2	24.543	108.579	57.338	49.97	25.37

Supplementary Fig. 243. HPLC spectrum of racemic (*M, E*)-3a

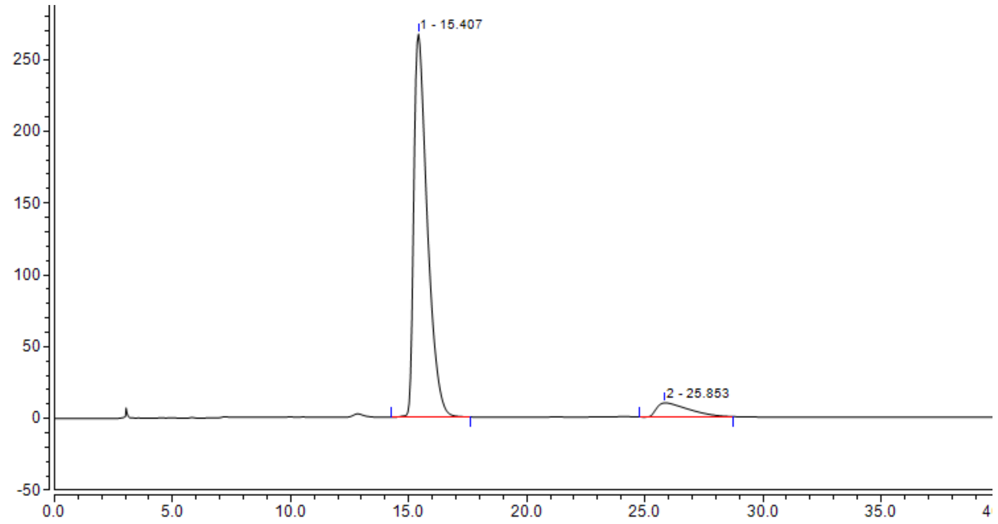

Peak	Ret. Time (min)	Area (mAu*min)	Height (mAu)	Area %	Height %
1	15.407	176.556	267.040	92.06	96.47
2	25.853	15.237	9.780	7.94	3.53

Supplementary Fig. 244. HPLC spectrum of chiral (*M, E*)-3a

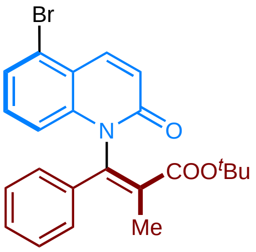

(*P*, *Z*)-**3s**

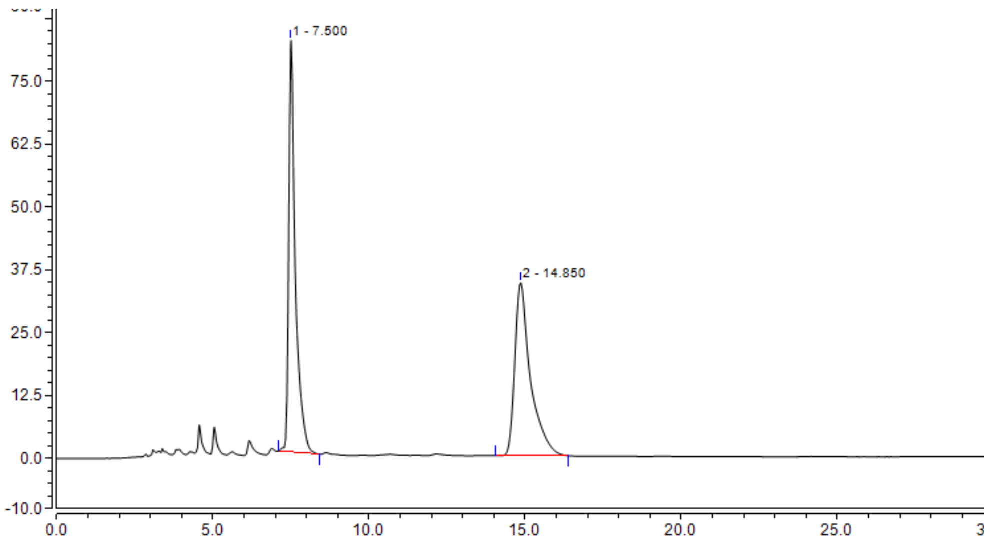

Peak	Ret. Time (min)	Area (mAu*min)	Height (mAu)	Area %	Height %
1	7.500	20.247	81.839	50.38	70.37
2	14.850	19.942	34.465	49.62	29.63

Supplementary Fig. 245. HPLC spectrum of racemic (*P*, *Z*)-3s

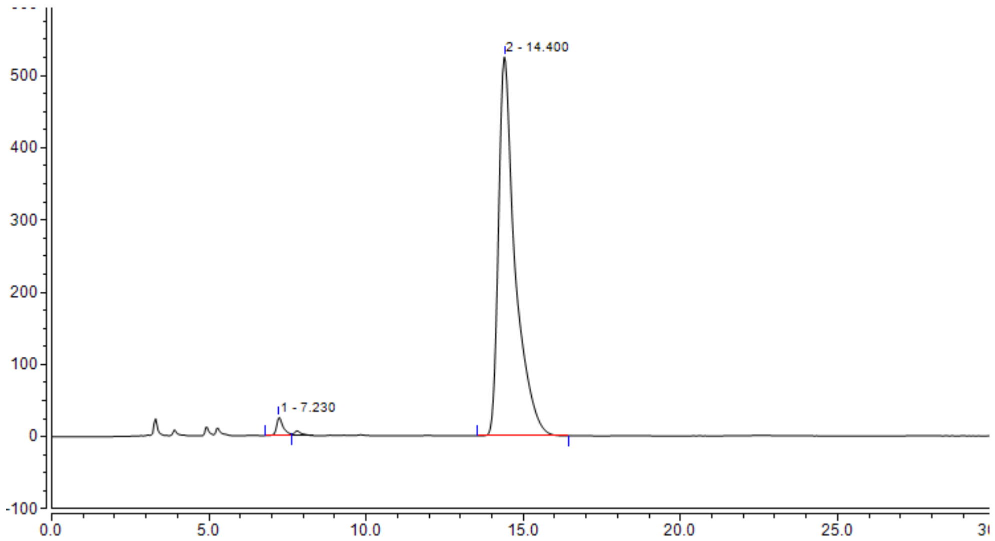

Peak	Ret. Time (min)	Area (mAu*min)	Height (mAu)	Area %	Height %
1	7.230	6.236	25.183	1.91	4.58
2	14.400	320.920	524.797	98.09	95.42

Supplementary Fig. 246. HPLC spectrum of chiral (*P*, *Z*)-3s

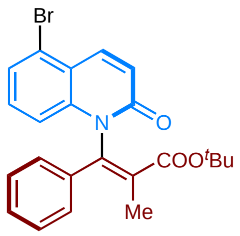

(*M*, *Z*)-**3s**

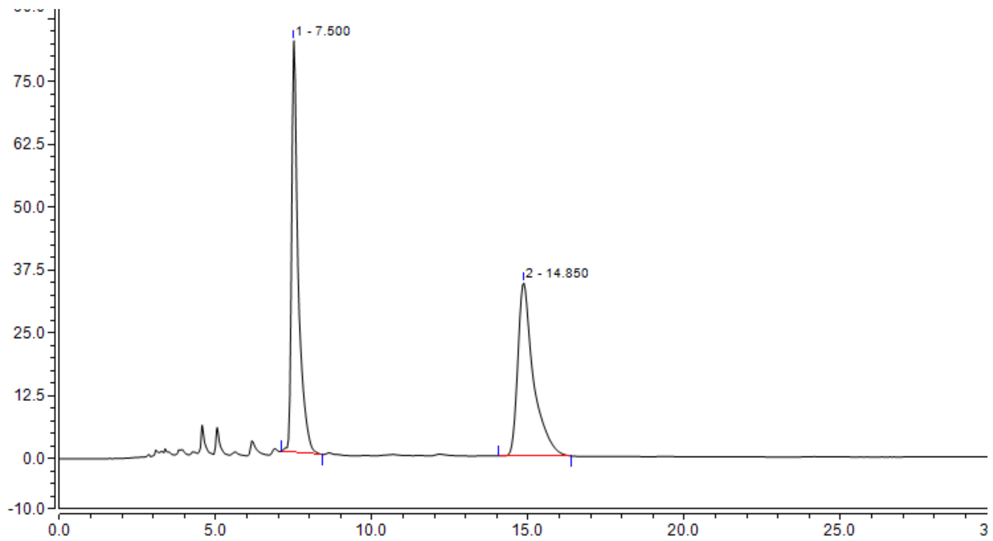

Peak	Ret. Time (min)	Area (mAu*min)	Height (mAu)	Area %	Height %
1	7.500	20.247	81.839	50.38	70.37
2	14.850	19.942	34.465	49.62	29.63

Supplementary Fig. 247. HPLC spectrum of racemic (*M*, *Z*)-3s

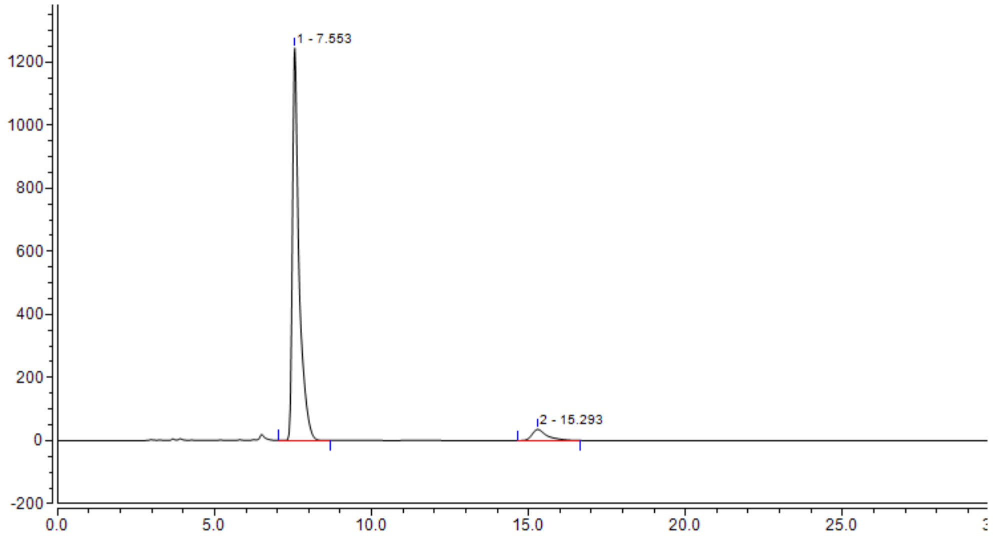

Peak	Ret. Time (min)	Area (mAu*min)	Height (mAu)	Area %	Height %
1	7.553	305.037	1243.669	93.91	97.23
2	15.293	19.790	35.465	6.09	2.77

Supplementary Fig. 248. HPLC spectrum of chiral (*M*, *Z*)-3s

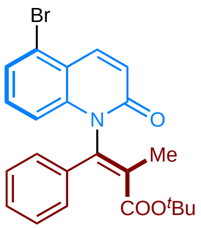

(*P*, *E*)-**3s**

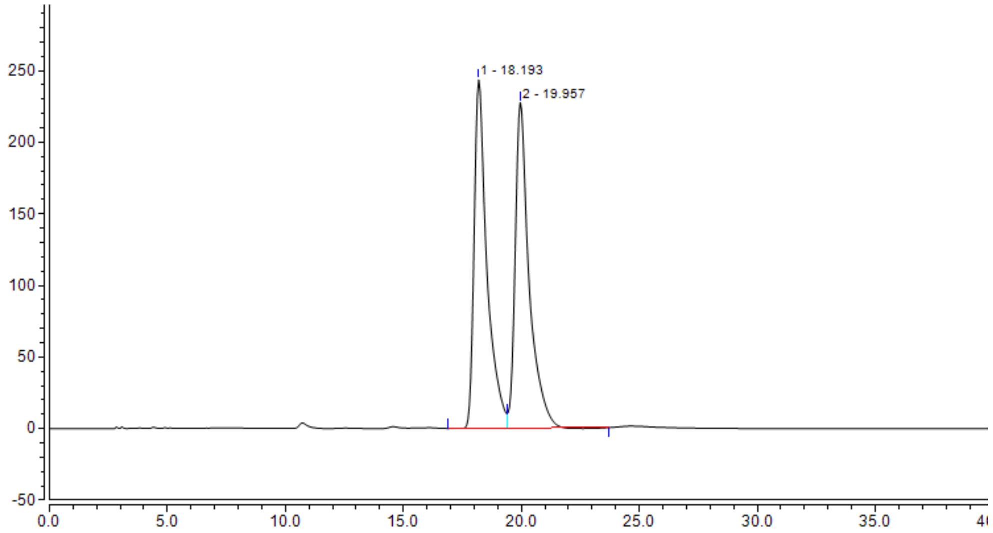

Peak	Ret. Time (min)	Area (mAu*min)	Height (mAu)	Area %	Height %
1	18.193	153.623	243.508	49.82	51.71
2	19.957	154.722	227.430	50.18	48.29

Supplementary Fig. 249. HPLC spectrum of racemic (*P*, *E*)-3s

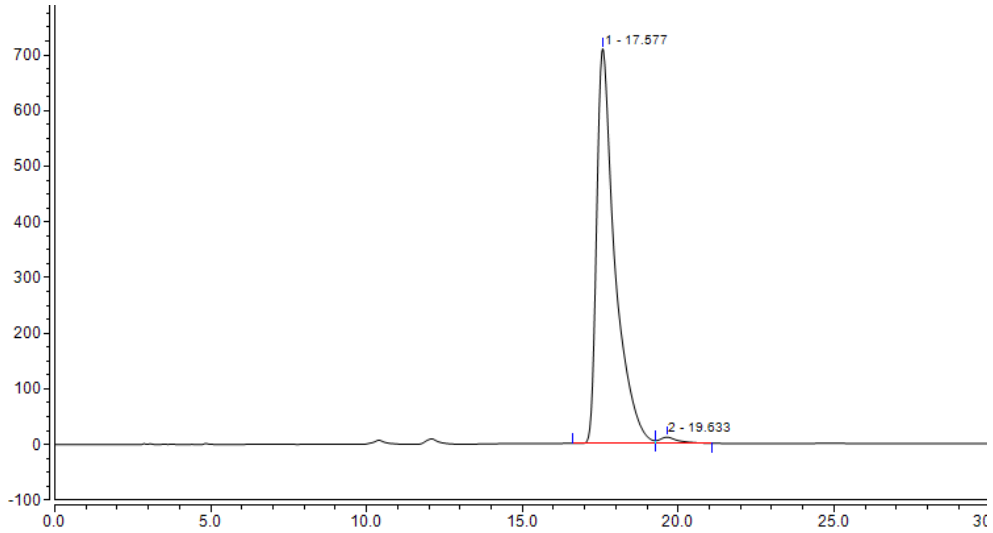

Peak	Ret. Time (min)	Area (mAu*min)	Height (mAu)	Area %	Height %
1	17.577	480.214	708.963	98.44	98.46
2	19.633	7.597	11.112	1.56	1.54

Supplementary Fig. 250. HPLC spectrum of chiral (*P*, *E*)-3s

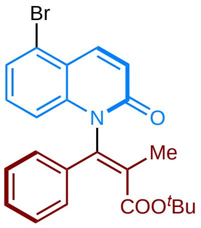

(M, E)-**3s**

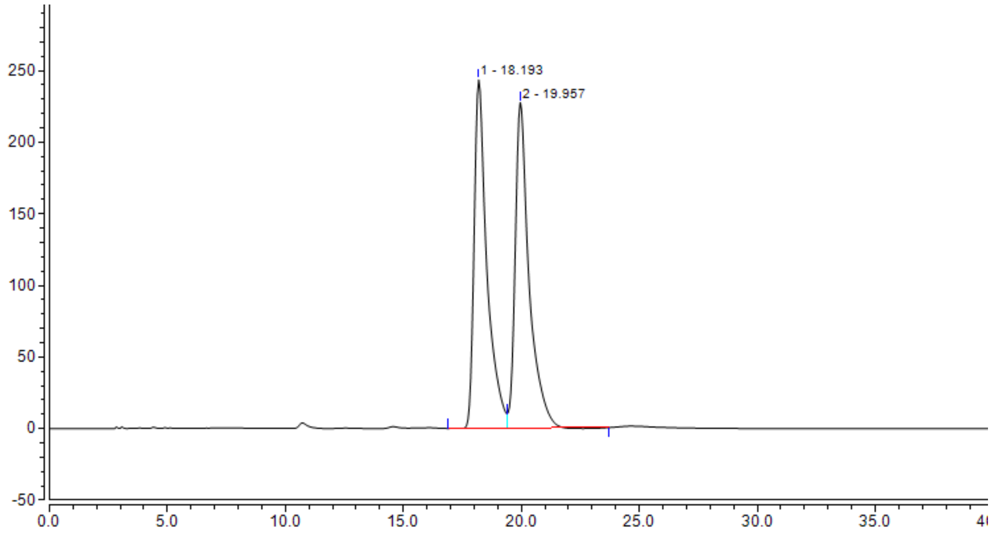

Peak	Ret. Time (min)	Area (mAu*min)	Height (mAu)	Area %	Height %
1	18.193	153.623	243.508	49.82	51.71
2	19.957	154.722	227.430	50.18	48.29

Supplementary Fig. 251. HPLC spectrum of racemic (*M*, *E*)-3s

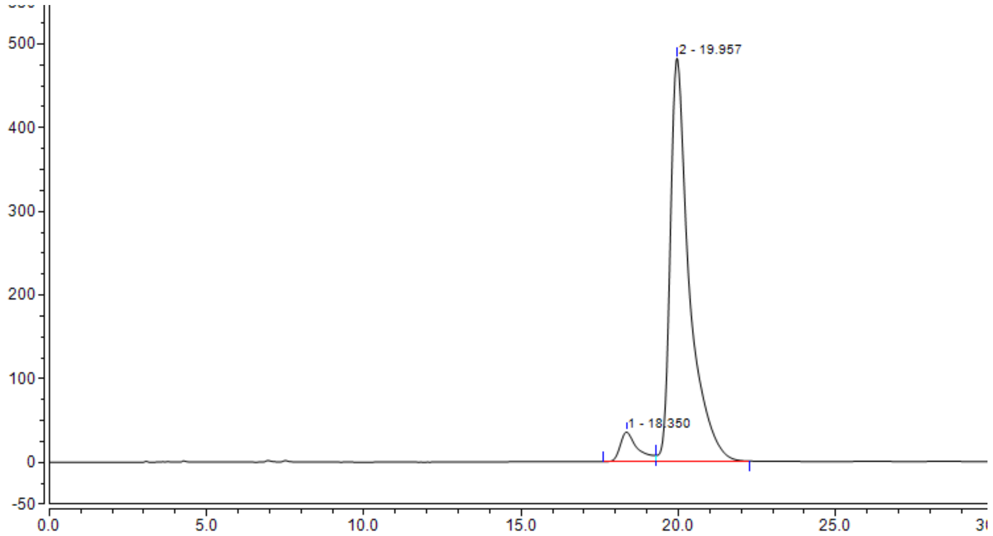

Peak	Ret. Time (min)	Area (mAu*min)	Height (mAu)	Area %	Height %
1	18.350	22.506	35.109	6.24	6.79
2	19.957	338.245	481.880	93.76	93.21

Supplementary Fig. 252. HPLC spectrum of chiral (*M*, *E*)-3s

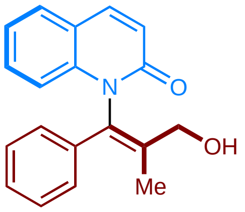

(*P*, *Z*)-**4**

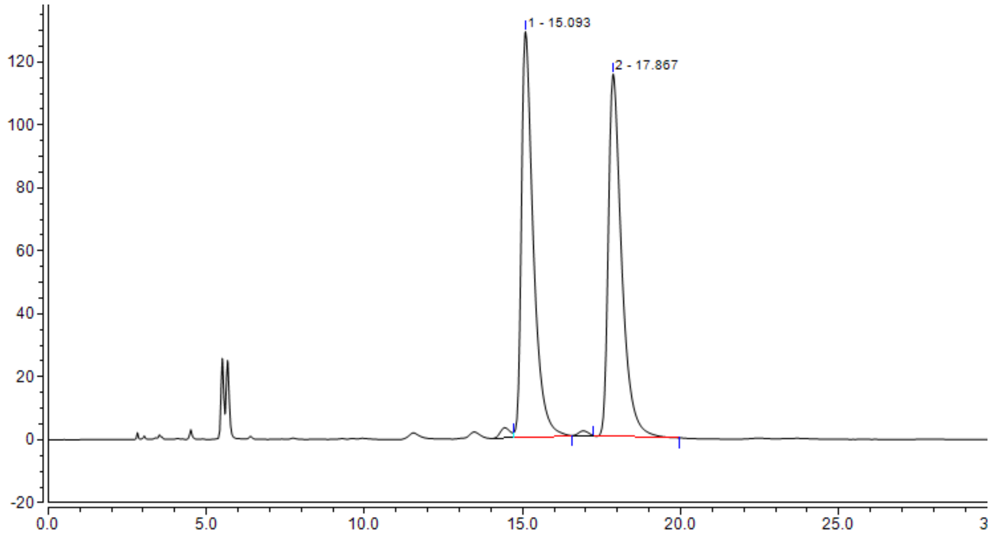

Peak	Ret. Time (min)	Area (mAu*min)	Height (mAu)	Area %	Height %
1	15.093	56.387	128.951	50.13	52.85
2	17.867	56.106	115.049	49.87	47.15

Supplementary Fig. 253. HPLC spectrum of racemic (*P*, *Z*)-4

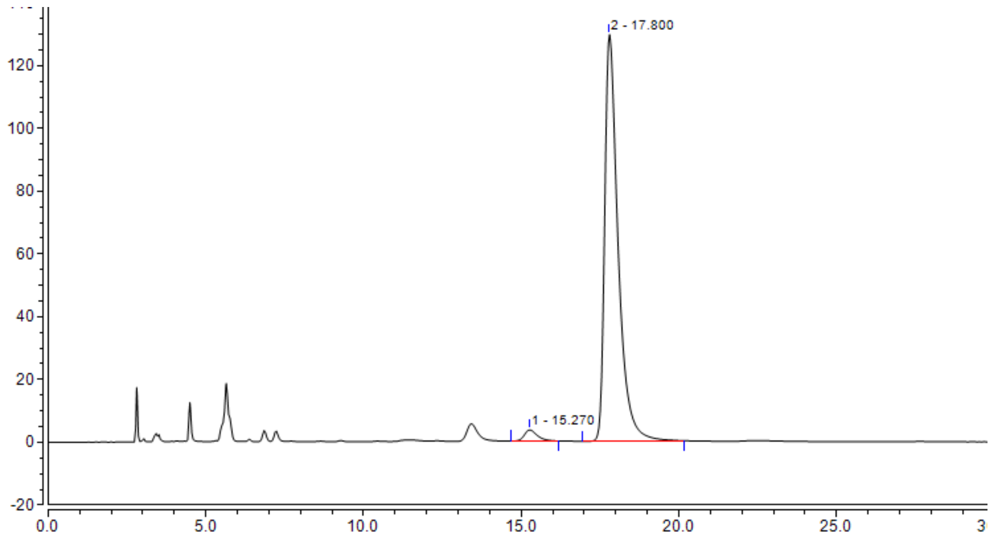

Peak	Ret. Time (min)	Area (mAu*min)	Height (mAu)	Area %	Height %
1	15.270	1.682	3.556	2.57	2.67
2	17.800	63.657	129.573	97.43	97.33

Supplementary Fig. 254. HPLC spectrum of chiral (*P*, *Z*)-4

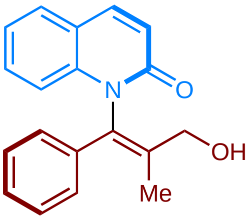

(*M, Z*)-**4**

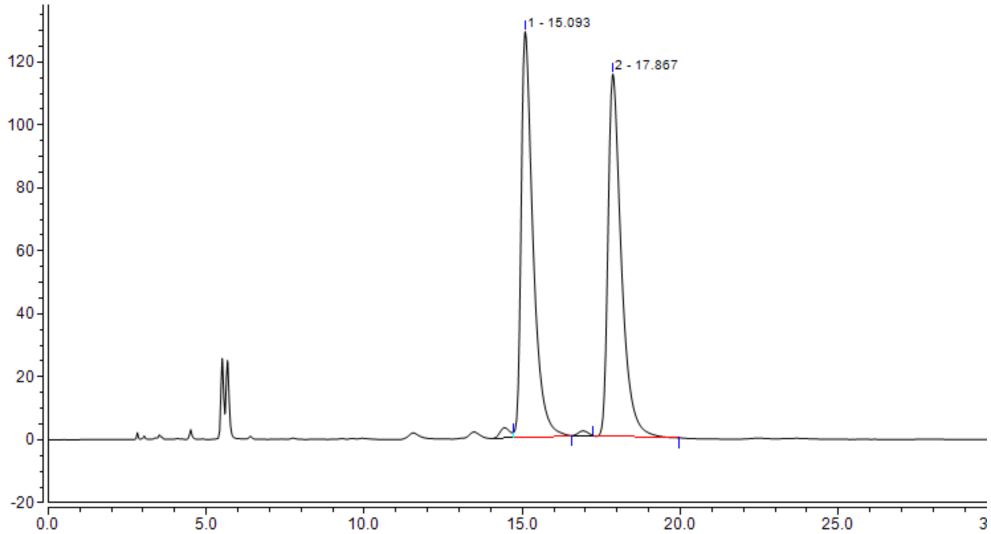

Peak	Ret. Time (min)	Area (mAu*min)	Height (mAu)	Area %	Height %
1	15.093	56.387	128.951	50.13	52.85
2	17.867	56.106	115.049	49.87	47.15

Supplementary Fig. 255. HPLC spectrum of racemic (*M, Z*)-4

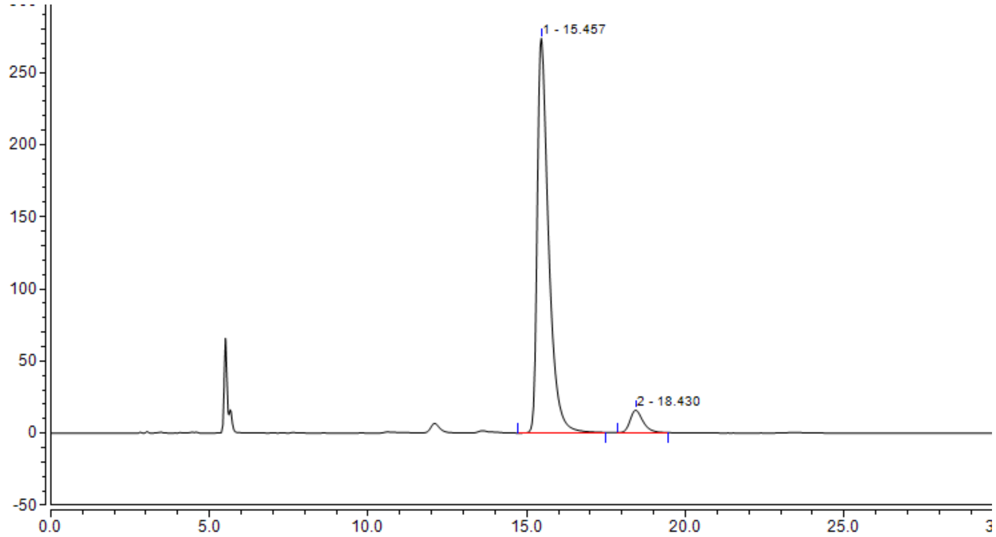

Peak	Ret. Time (min)	Area (mAu*min)	Height (mAu)	Area %	Height %
1	15.457	113.138	273.578	93.91	94.63
2	18.430	7.333	15.526	6.09	5.37

Supplementary Fig. 256. HPLC spectrum of chiral (*M, Z*)-4

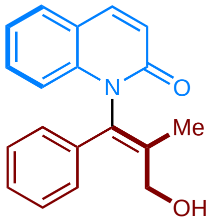

(*P*, *E*)-**4**

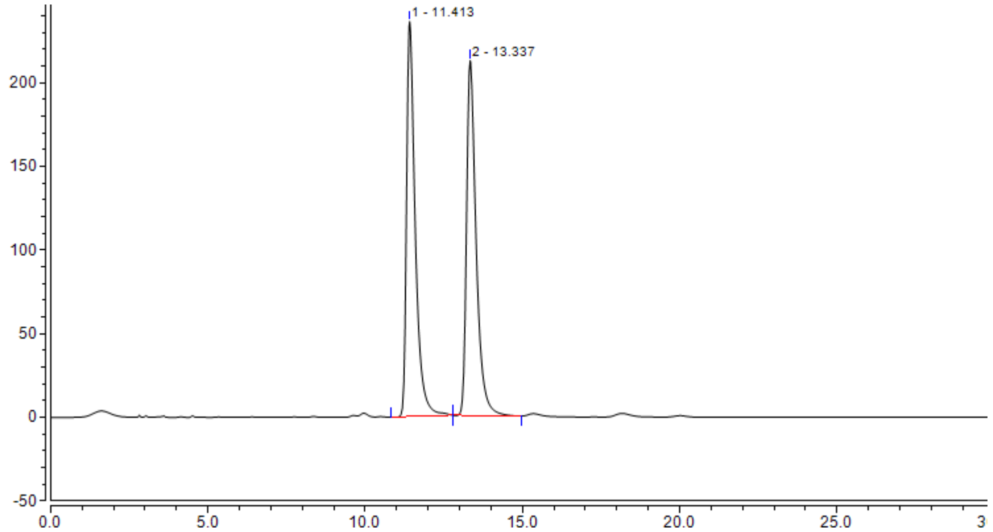

Peak	Ret. Time (min)	Area (mAu*min)	Height (mAu)	Area %	Height %
1	11.413	75.618	236.244	50.16	52.69
2	13.337	75.130	212.122	49.84	47.31

Supplementary Fig. 257. HPLC spectrum of racemic (*P*, *E*)-4

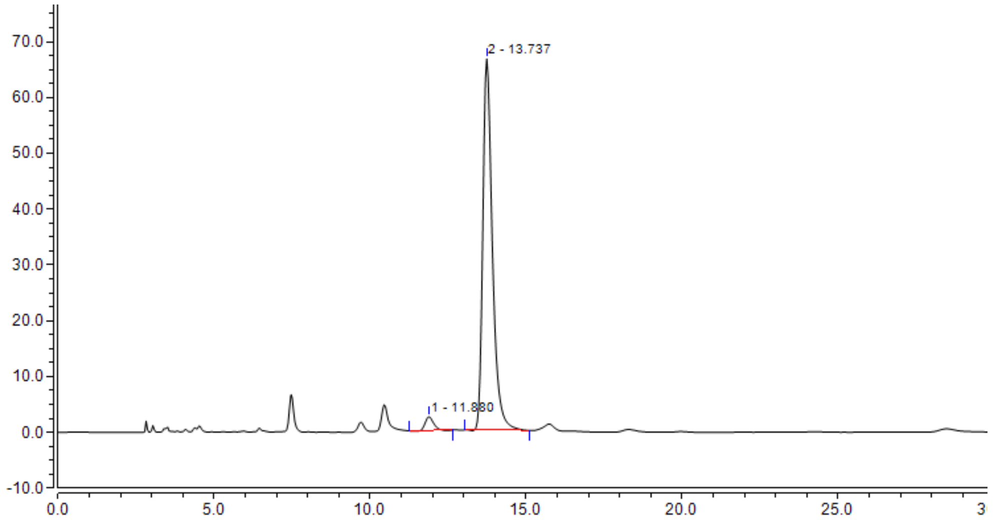

Peak	Ret. Time (min)	Area (mAu*min)	Height (mAu)	Area %	Height %
1	11.880	0.712	2.443	2.93	3.54
2	13.737	23.601	66.492	97.07	96.46

Supplementary Fig. 258. HPLC spectrum of chiral (*P*, *E*)-4

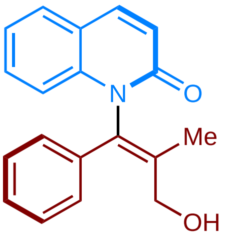

(*M, E*)-**4**

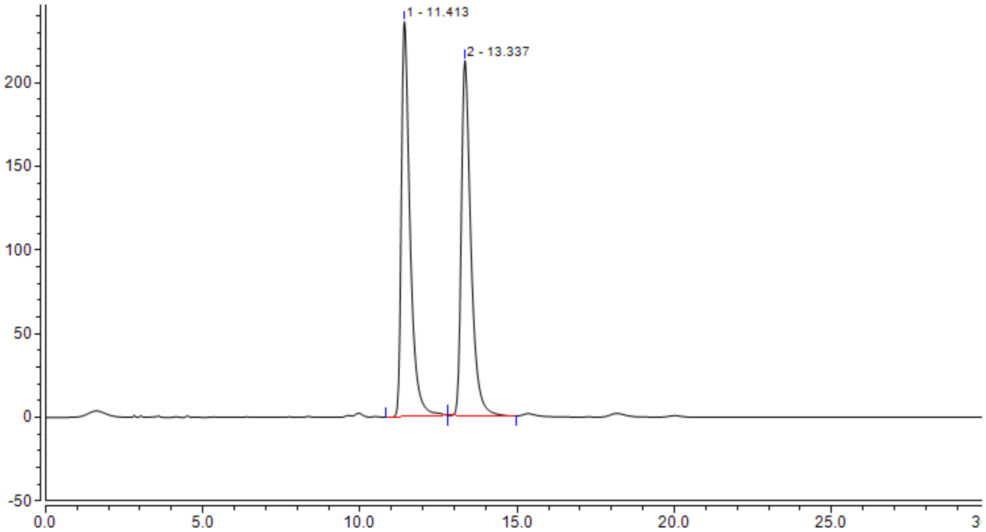

Peak	Ret. Time (min)	Area (mAu*min)	Height (mAu)	Area %	Height %
1	11.413	75.618	236.244	50.16	52.69
2	13.337	75.130	212.122	49.84	47.31

Supplementary Fig. 259. HPLC spectrum of racemic (*M, E*)-4

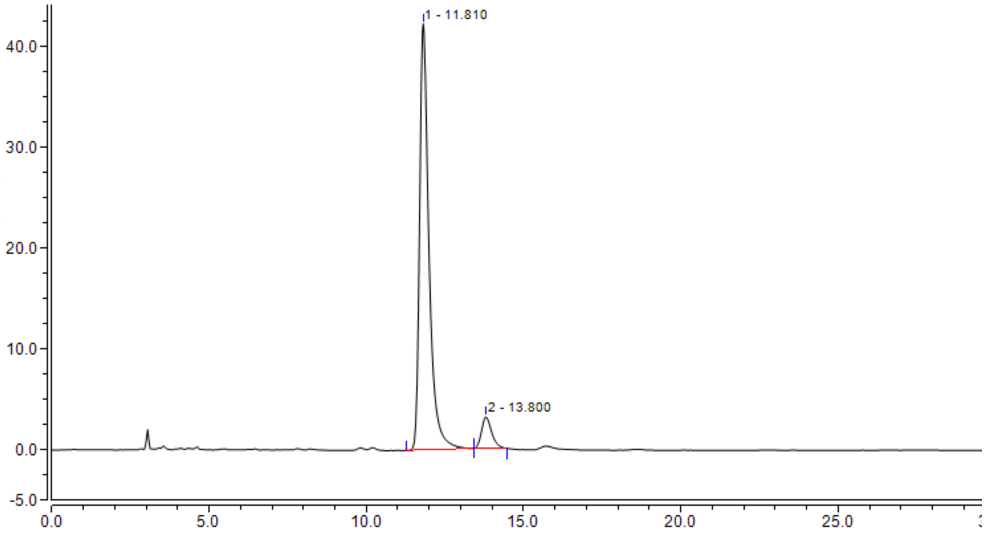

Peak	Ret. Time (min)	Area (mAu*min)	Height (mAu)	Area %	Height %
1	11.810	14.613	42.287	92.61	93.14
2	13.800	1.166	3.117	7.39	6.86

Supplementary Fig. 260. HPLC spectrum of chiral (*M, E*)-4

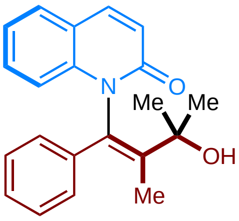

(*P*, *Z*)-**5**

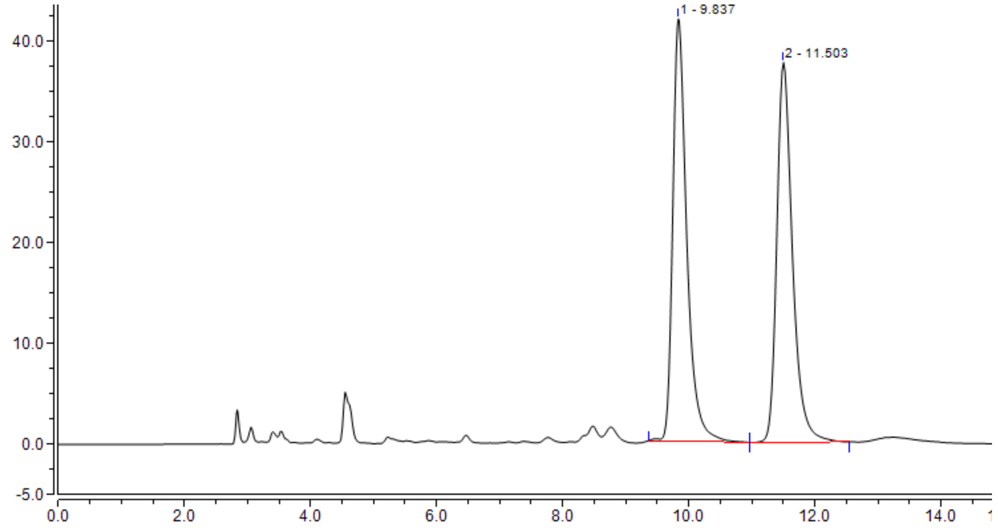

Peak	Ret. Time (min)	Area (mAu*min)	Height (mAu)	Area %	Height %
1	9.837	10.806	41.937	49.92	52.66
2	11.503	10.839	37.707	50.08	47.34

Supplementary Fig. 261. HPLC spectrum of racemic (*P*, *Z*)-5

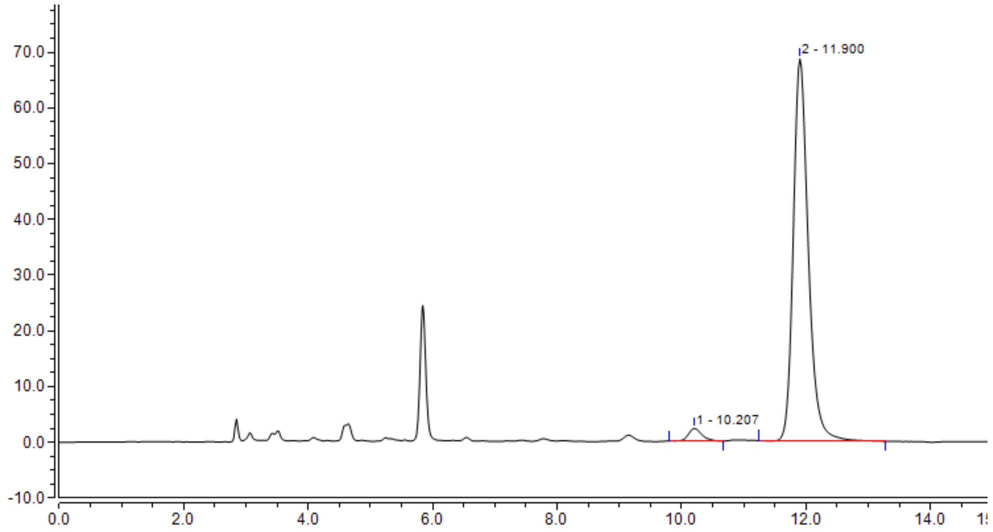

Peak	Ret. Time (min)	Area (mAu*min)	Height (mAu)	Area %	Height %
1	10.207	0.561	2.253	2.85	3.18
2	11.900	19.129	68.551	97.15	96.82

Supplementary Fig. 262. HPLC spectrum of chiral (*P*, *Z*)-5

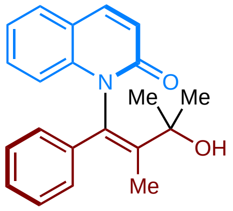

(*M*, *Z*)-**5**

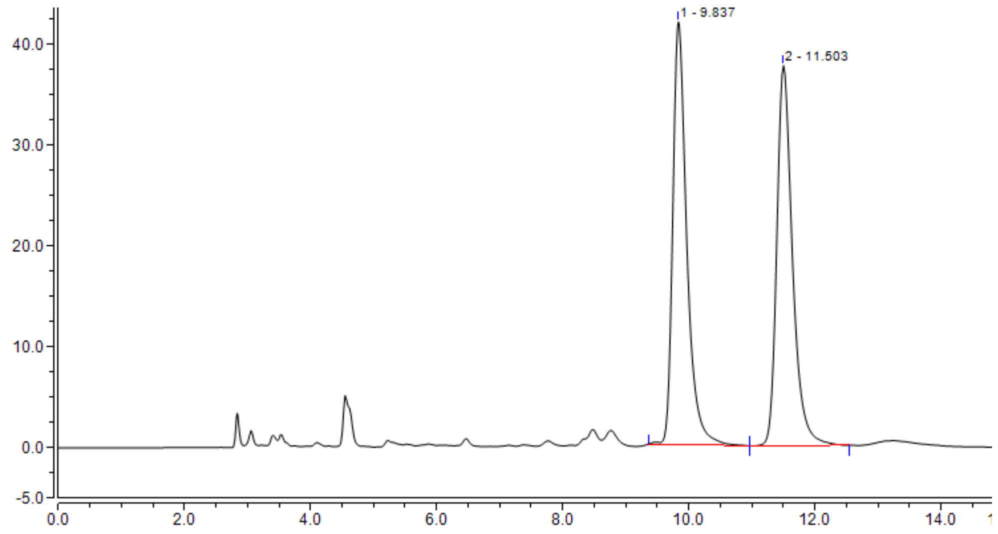

Peak	Ret. Time (min)	Area (mAu*min)	Height (mAu)	Area %	Height %
1	9.837	10.806	41.937	49.92	52.66
2	11.503	10.839	37.707	50.08	47.34

Supplementary Fig. 263. HPLC spectrum of racemic (*M*, *Z*)-5

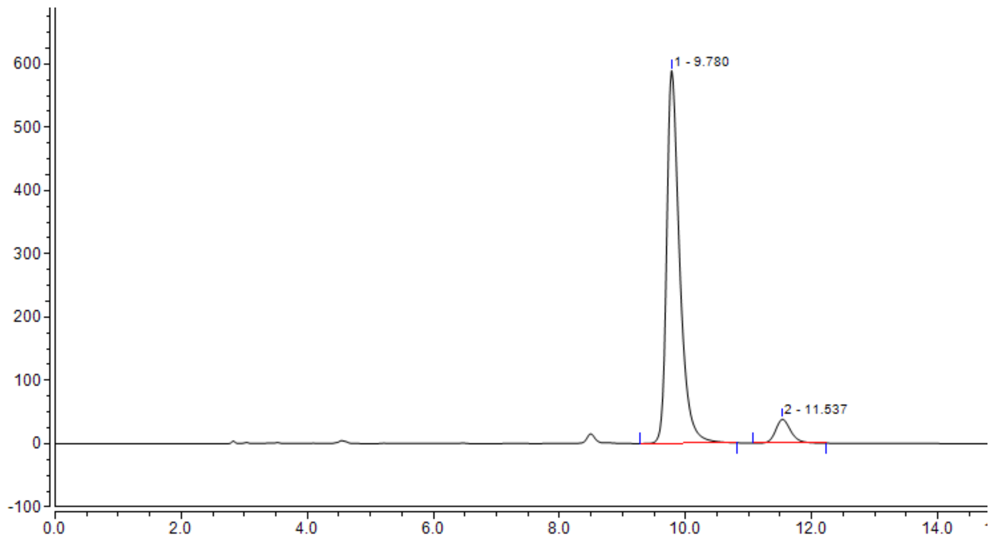

Peak	Ret. Time (min)	Area (mAu*min)	Height (mAu)	Area %	Height %
1	9.780	140.164	588.896	92.98	93.98
2	11.537	10.589	37.701	7.02	6.02

Supplementary Fig. 264. HPLC spectrum of chiral (*M*, *Z*)-5

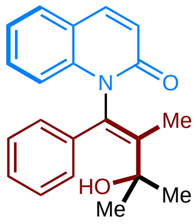

(*P*, *E*)-**5**

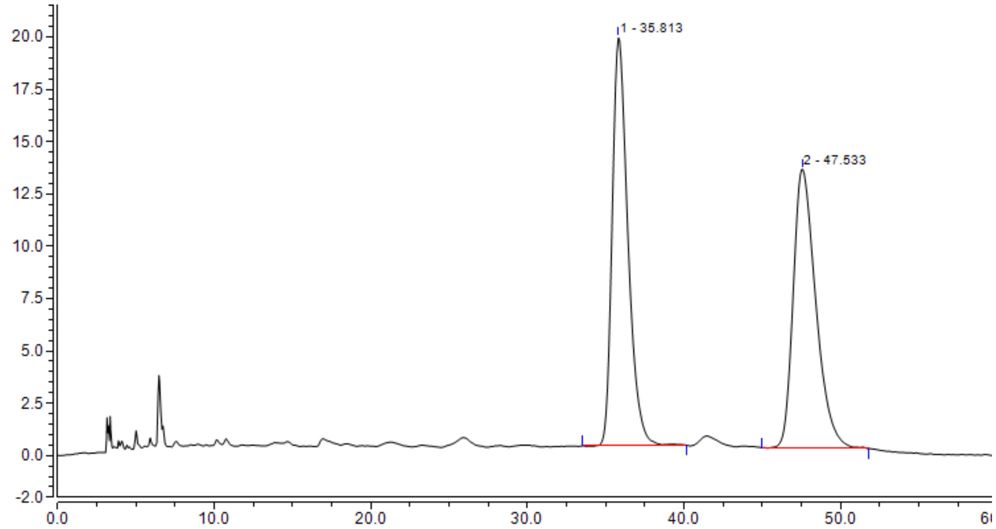

Peak	Ret. Time (min)	Area (mAu*min)	Height (mAu)	Area %	Height %
1	35.813	22.689	19.495	50.41	59.39
2	47.533	22.316	13.330	49.59	40.61

Supplementary Fig. 265. HPLC spectrum of racemic (*P*, *E*)-5

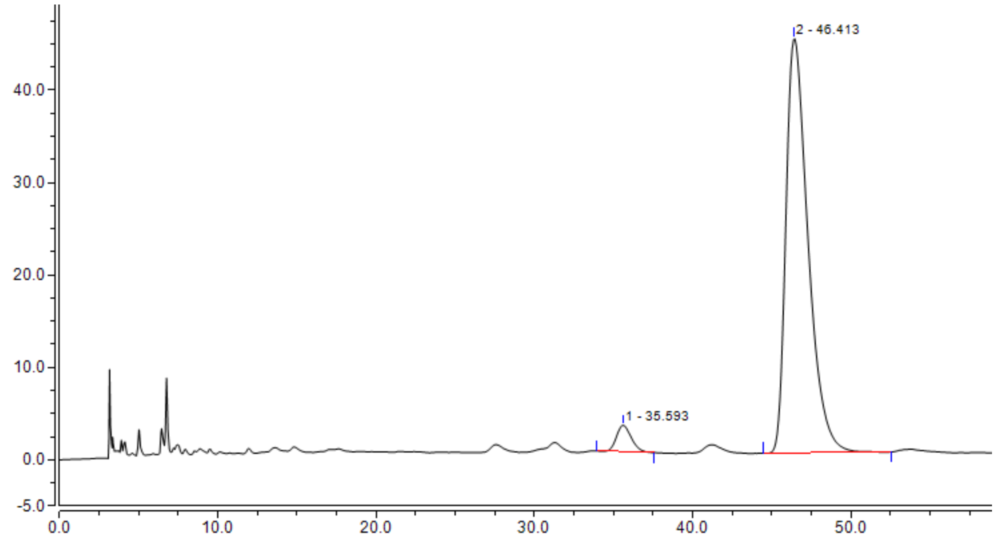

Peak	Ret. Time (min)	Area (mAu*min)	Height (mAu)	Area %	Height %
1	35.593	3.048	2.856	4.04	5.99
2	46.413	72.440	44.850	95.96	94.01

Supplementary Fig. 266. HPLC spectrum of chiral (*P*, *E*)-5

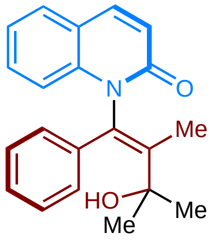

(*M*, *E*)-**5**

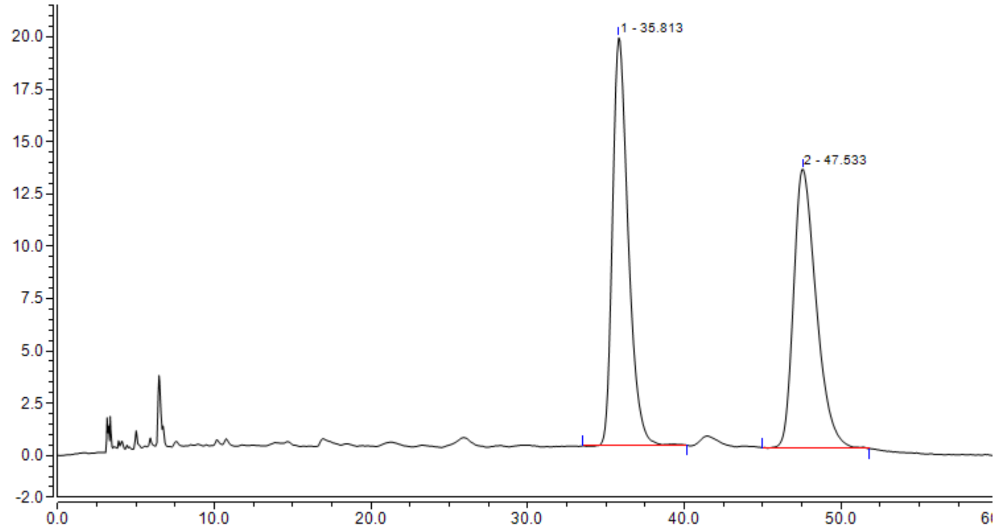

Peak	Ret. Time (min)	Area (mAu*min)	Height (mAu)	Area %	Height %
1	35.813	22.689	19.495	50.41	59.39
2	47.533	22.316	13.330	49.59	40.61

Supplementary Fig. 267. HPLC spectrum of racemic (*M*, *E*)-5

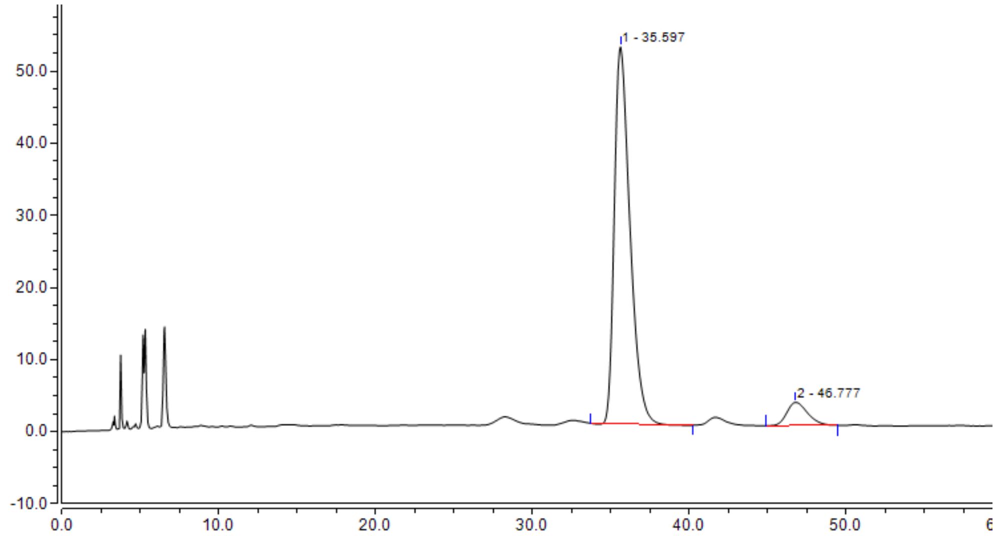

Peak	Ret. Time (min)	Area (mAu*min)	Height (mAu)	Area %	Height %
1	35.597	60.536	52.306	92.46	94.20
2	46.777	4.938	3.219	7.54	5.80

Supplementary Fig. 268. HPLC spectrum of chiral (*M*, *E*)-5

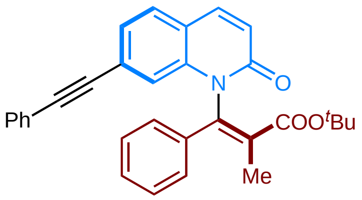

(P, Z)-**6**

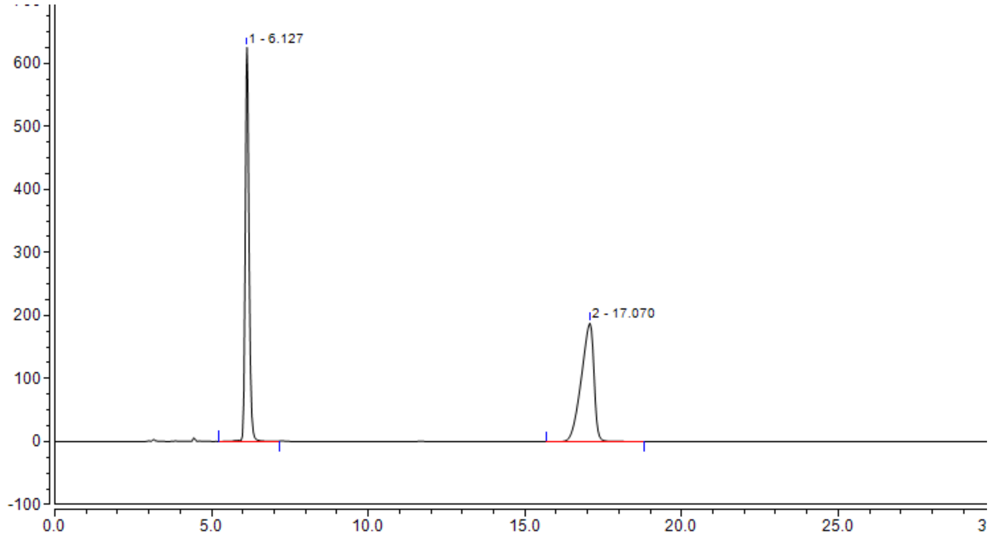

Peak	Ret. Time (min)	Area (mAu*min)	Height (mAu)	Area %	Height %
1	6.127	87.612	624.960	49.96	76.88
2	17.070	87.755	187.981	50.04	23.12

Supplementary Fig. 269. HPLC spectrum of racemic (*P*, *Z*)-6

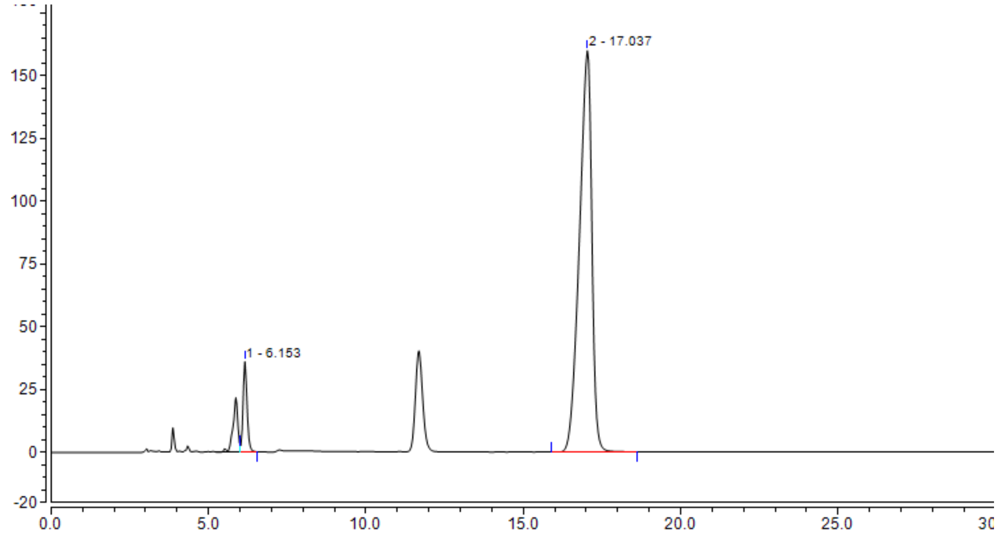

Peak	Ret. Time (min)	Area (mAu*min)	Height (mAu)	Area %	Height %
1	6.153	5.219	35.924	6.67	18.35
2	17.037	72.975	159.814	93.33	81.65

Supplementary Fig. 270. HPLC spectrum of chiral (*P*, *Z*)-6

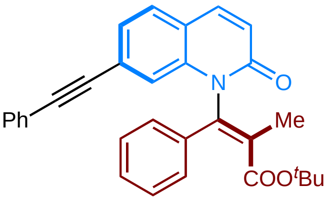

(*P*, *E*)-**6**

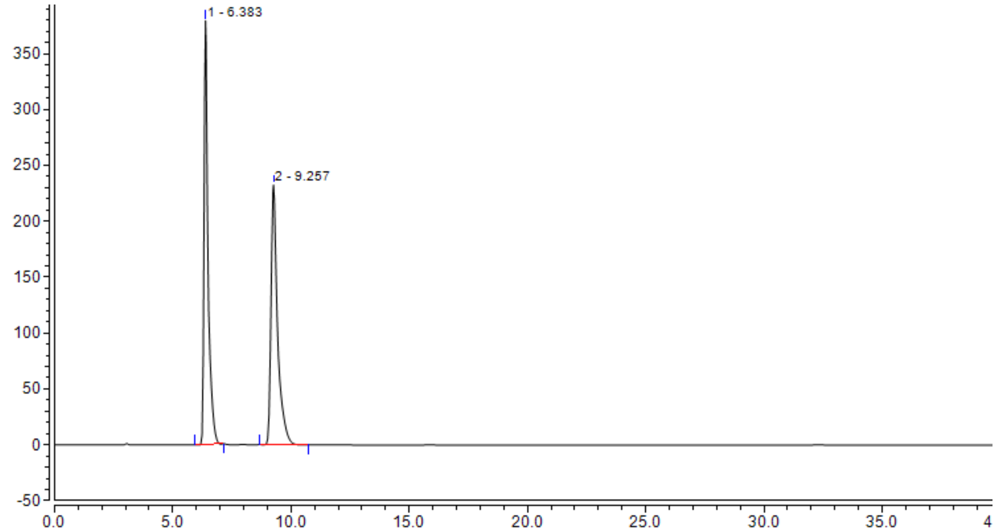

Peak	Ret. Time (min)	Area (mAu*min)	Height (mAu)	Area %	Height %
1	6.383	73.523	379.077	49.87	61.99
2	9.257	73.910	232.393	50.13	38.01

Supplementary Fig. 271. HPLC spectrum of racemic (*P*, *E*)-6

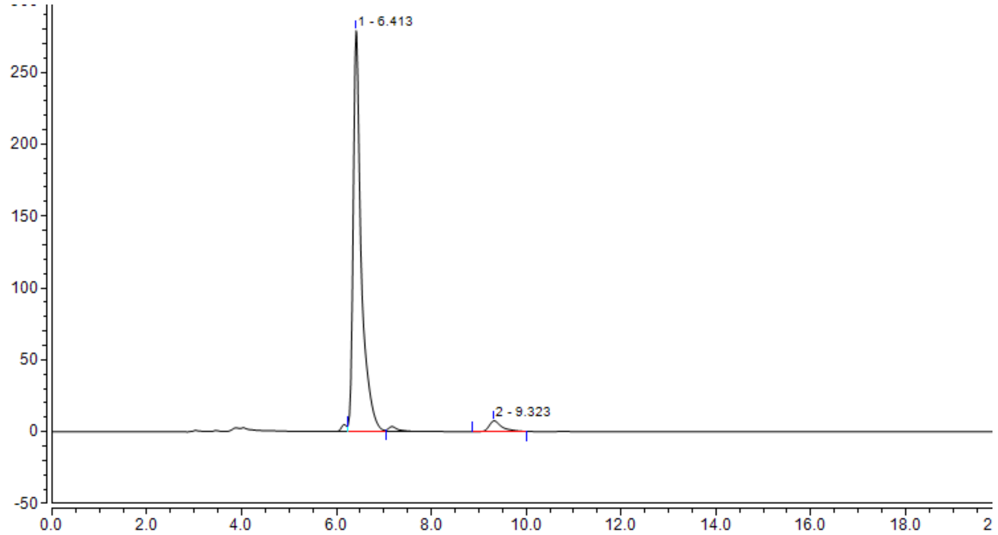

Peak	Ret. Time (min)	Area (mAu*min)	Height (mAu)	Area %	Height %
1	6.413	54.735	278.833	95.94	97.38
2	9.323	2.315	7.515	4.06	2.62

Supplementary Fig. 272. HPLC spectrum of chiral (*P*, *E*)-6

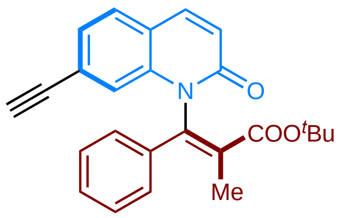

(P, Z)-**7**

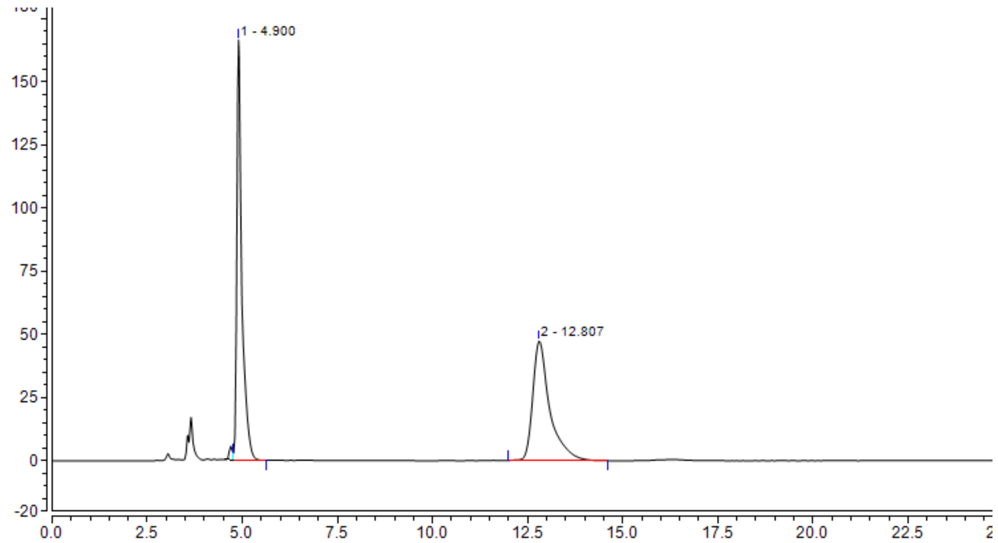

Peak	Ret. Time (min)	Area (mAu*min)	Height (mAu)	Area %	Height %
1	4.900	25.228	166.207	50.71	77.81
2	12.807	24.520	47.404	49.29	22.19

Supplementary Fig. 273. HPLC spectrum of racemic (*P*, *Z*)-7

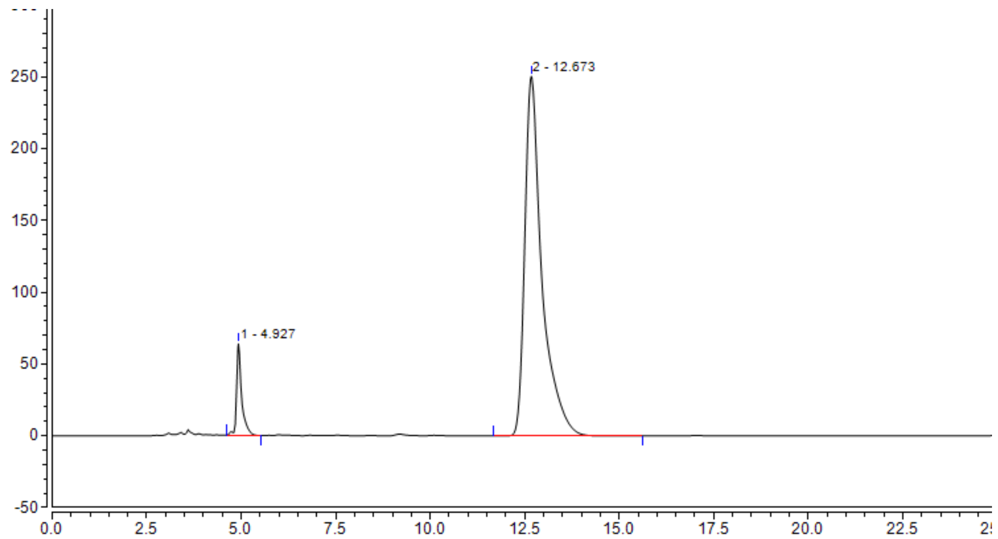

Peak	Ret. Time (min)	Area (mAu*min)	Height (mAu)	Area %	Height %
1	4.927	9.838	63.740	6.95	20.29
2	12.675	131.754	250.461	93.05	79.71

Supplementary Fig. 274. HPLC spectrum of chiral (*P*, *Z*)-7

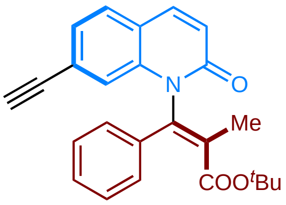

(*P*, *E*)-**7**

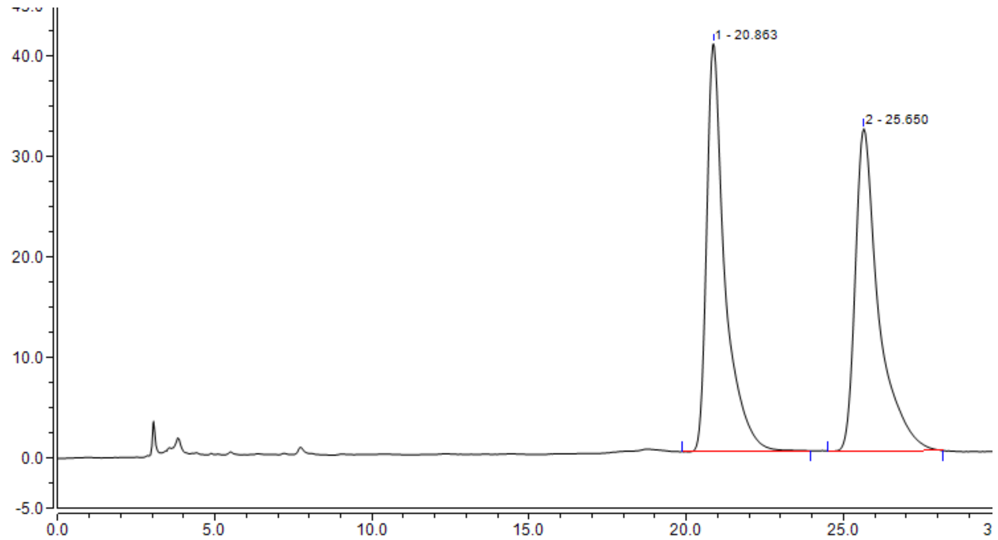

Peak	Ret. Time (min)	Area (mAu*min)	Height (mAu)	Area %	Height %
1	20.863	27.270	40.567	49.69	55.86
2	25.650	27.614	32.060	50.31	44.14

Supplementary Fig. 275. HPLC spectrum of racemic (*P*, *E*)-7

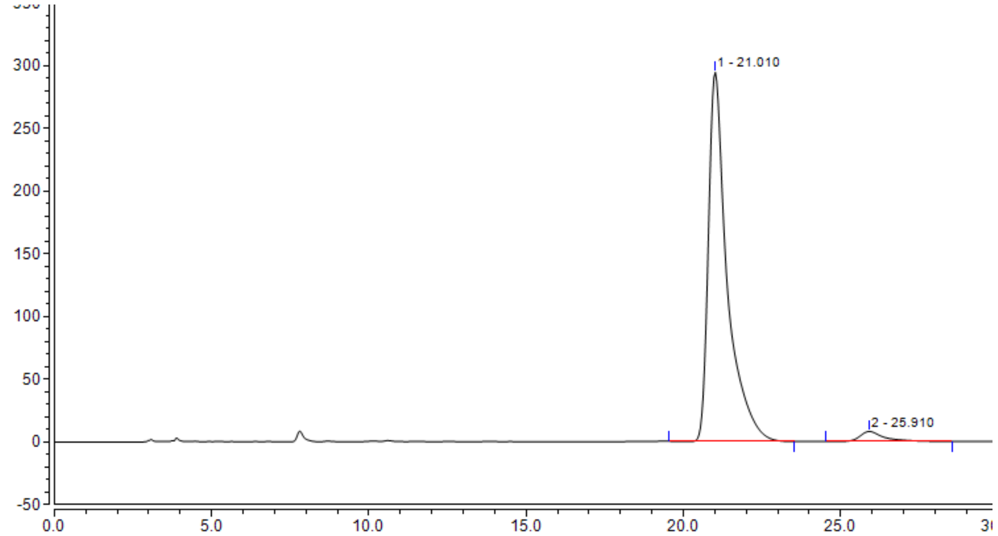

Peak	Ret. Time (min)	Area (mAu*min)	Height (mAu)	Area %	Height %
1	21.010	203.840	294.455	96.52	97.32
2	25.910	7.351	8.098	3.48	2.68

Supplementary Fig. 276. HPLC spectrum of chiral (*P*, *E*)-7

References

[1] Frisch, M. J.; Trucks, G. W.; Schlegel, H. B.; Scuseria, G. E.; Robb, M. A.; Cheeseman, J. R.; Scalmani, G.; Barone, V.; Petersson, G. A.; Nakatsuji, H.; Li, X.; Caricato, M.; Marenich, A.; Bloino,; Janesko, J. B.; Gomperts, G. R.; Mennucci, B.; Hratchian, H. P.; Ortiz, J. V.; Izmaylov, A. F.; Sonnenberg, J. L.; Williams-Young, D.; Ding, F.; Lipparini, F.; Egidi, F.; Goings, J.; Peng, B.; Petrone, A.; Henderson, T.; Ranasinghe, D.; Zakrzewski, V. G.; Gao, J.; Rega, N.; Zheng, G.; Liang, W.; Hada, M.; Ehara, M.; Toyota, K.; Fukuda, R.; Hasegawa, J.; Ishida, M.; Nakajima, T.; Honda, Y.; Kitao, O.; Nakai, H.; Vreven, T.; Throssell, K.; Montgomery, J. A.; Jr.; Peralta,J. E.; Ogliaro, F.; Bearpark, M.; Heyd, J. J.; Brothers, E. K.; Kudin, N.; Staroverov, V. N.; Keith, T.; Kobayashi, R.; Normand, J.; Raghavachari, K.; Rendell, A.; Burant, J. C.; Iyengar, S. S.; Tomasi, J.; Cossi, M.; Millam, J. M.; Klene, M.; Adamo, C.; Cammi, R.; Ochterski, J. W.; Martin, R. L.; Morokuma, K.; Farkas, O.; Foresman, J. B. & Fox, D. J. Gaussian, Inc., Wallingford CT, 2016.

[2] Zhao, Y. & Truhlar, D. G. The M06 suite of density functionals for main group thermochemistry, thermochemical kinetics, noncovalent interactions, excited states, and transition elements: two new functionals and systematic testing of four M06-class functionals and 12 other functionals. *Theor. Chem. Acc.* **120**, 215-241 (2007).

[3] Weigend, F. & Ahlrichs, R. Balanced basis sets of split valence, triple zeta valence and quadruple zeta valence quality for H to Rn: Design and assessment of accuracy. *Phys. Chem. Chem. Phys.* **7**, 3297-3305 (2005).

[4] Marenich, A. V.; Cramer, C. J. & Truhlar, D. G. Universal solvation model based on solute electron density and on a continuum model of the solvent defined by the bulk dielectric constant and atomic surface tensions. *J. Phys. Chem. B.* **2009**, *113*, 6378-6396.

[5] Lu, T. “sobMECP program”; http://sobereva.com/286.(10-27,2023)

[6] Lu, T. & Chen, F. Multiwfn: A multifunctional wavefunction analyzer, *J. Comput. Chem.* **33**, 580-592 (2012).
